# Phylogenetic Analysis, Lineage-Specific Expansion and Functional Divergence of *seed dormancy 4*-Like Genes in Plants

**DOI:** 10.1371/journal.pone.0153717

**Published:** 2016-06-14

**Authors:** Saminathan Subburaj, Shuanghe Cao, Xianchun Xia, Zhonghu He

**Affiliations:** 1 Institute of Crop Science, National Wheat Improvement Center, Chinese Academy of Agricultural Sciences, 12 Zhongguancun South Street, Beijing, 100081, China; 2 International Maize and Wheat Improvement Center (CIMMYT) China Office, 12 Zhongguancun South Street, Beijing, 100081, China; East Carolina University, UNITED STATES

## Abstract

The rice gene *seed dormancy 4* (*OsSdr4*) functions in seed dormancy and is a major factor associated with pre-harvest sprouting (PHS). Although previous studies of this protein family were reported for rice and other species, knowledge of the evolution of genes homologous to *OsSdr4* in plants remains inadequate. Fifty four *Sdr4-*like (hereafter designated *Sdr4L*) genes were identified in nine plant lineages including 36 species. Phylogenetic analysis placed these genes in eight subfamilies (I-VIII). Genes from the same lineage clustered together, supported by analysis of conserved motifs and exon-intron patterns. Segmental duplications were present in both dicot and monocot clusters, while tandemly duplicated genes occurred only in monocot clusters indicating that both tandem and segmental duplications contributed to expansion of the grass I and II subfamilies. Estimation of the approximate ages of the duplication events indicated that ancestral *Sdr4* genes evolved from a common angiosperm ancestor, about 160 million years ago (MYA). Moreover, diversification of *Sdr4L* genes in mono and dicot plants was mainly associated with genome-wide duplication and speciation events. Functional divergence was observed in all subfamily pairs, except IV/VIIIa. Further analysis indicated that functional constraints between subfamily pairs I/II, I/VIIIb, II/VI, II/VIIIb, II/IV, and VI/VIIIb were statistically significant. Site and branch-site model analyses of positive selection suggested that these genes were under strong adaptive selection pressure. Critical amino acids detected for both functional divergence and positive selection were mostly located in the loops, pointing to functional importance of these regions in this protein family. In addition, differential expression studies by transcriptome atlas of 11 *Sdr4L* genes showed that the duplicated genes may have undergone divergence in expression between plant species. Our findings showed that *Sdr4L* genes are functionally divergent and positively selected. These may contribute to further functional analysis and molecular evolution of *Sdr4L* gene families in land plants.

## Introduction

Seed dormancy can be defined as the process by which viable seeds lack the capacity to germinate even in the presence of favorable environmental conditions [1]. It is considered to be a way of regulating the distribution of seed germination in time, and to enable them to adapt in a diversity of habitats or in unfavorable conditions. Therefore, seed dormancy is an important component of plant fitness and an agronomically significant physiological trait [2, 3]. Pre-harvest sprouting (PHS) is a phenomenon in which seeds germinate within the spike caused by inappropriate level of dormancy under the prevailing wet weather conditions. PHS occurs in various cereal crops such as wheat, maize, rice and barley in most of grain production region of the world. Seed dormancy is been considered a main component of PHS, and is therefore an important quality trait. PHS affects grain yield and quality, and reduces the market value of the grains, thus the improvement of PHS is an important objective for the cereal breeding programs across the world [4].

Seed dormancy is a complex trait, influenced by various genetic factors with a substantial environmental influence. The induction and release of dormancy controlled by different kinds of regulators, which can be classified into seed maturation, hormonal, epigenetic and dormancy factors, has been reviewed [5, 6]. Several quantitative genetic studies have investigated quantitative trait loci (QTL) underlying seed dormancy. Molecular cloning of one of the first QTL in Arabidopsis, *DELAY OF GERMINATION* 1 (*DOG1*), identified novel genes involved in dormancy [7]. Recently, QTL mapping studies on dormancy/PHS traits in crop species led to the identification of two genes named *Seed dormancy 4* (*Sdr4*) in rice and wheat [8, 9] and *MOTHER OF FT AND TFL1* (*MFT*) in wheat [10]. The mechanism of action of most of these genes on dormancy in cereals remains poorly understood, particularly *Sdr4* because of the novel protein with unknown function [8]. Functional polymorphisms in genes those involves in various domestication traits has been reviewed [11]. In general, the natural variations in genes of particular trait of interest or homolog genes from other plant species suggested not causing any phenotypic variations during molecular assisted breeding [12]. Therefore in crop breeding attempts are to improve dormancy by using genes that are present in natural variations [12]. The current study focused on plant genes encoding amino acid sequences similar to that of *O*. *sativa Sdr4* (hereafter, *OsSdr4*), that is, *OsSdr4*-like genes. The discovery of dormancy-specific homologues of *Sdr4*-like (*Sdr4L*) genes in other plant species could also be useful in breeding strategies for improvement of PHS. Apart from a critical role in dormancy, these genes are also able to provide useful information for understanding evolutionary relationships among land plant lineages.

*Sdr4* were identified as intronless genes, encoding proteins without homology to other known proteins. A putative bipartite nuclear localization signal (NLS) motif (RKR_64-66_ KRKR_82-85_) is present in the N-terminal region of OsSdr4 proteins. A pentapeptide motif GQPEC_44-48_ was preserved near the C-terminal in OsSdr4 proteins and in several homologous dicot Sdr4 proteins [8]. Rice Sdr4 proteins were located in the nucleus and were more highly expressed in seeds. It was suggested that the global seed regulator *VP1* regulates transcriptional expression of *Sdr4* genes. Functional nucleotide variation in the coding region of *OsSdr4* and promoter causes differences in the dormancy levels of individual rice and wheat cultivars [8, 9]. *Sdr4* possibly interacts with the expression of *DOG1*-like genes [8]. However, there is no clear evidence for a mechanism of action of Sdr4 proteins in the seed dormancy pathway.

A multi-species approach to the *Sdr4* gene family in all plant lineages has not been reported [8, 9]. A comprehensive evolutionary study of the *Sdr4* gene family would therefore facilitate an understanding of its function and evolution in land plant species. Recent whole genome sequencing projects enable us to survey and characterize *Sdr4* homologs that encode similar structural features across plant species. We identified *Sdr4*-like (*Sdr4L*) gene families in many mono- and eudicotyledonous plants. The identified candidate genes were used to perform a comprehensive analysis of evolutionary relationships among *Sdr4L* genes. Our results show that the *Sdr4L* gene family recently expanded by duplication events in both mono- and dicot plant species. Conserved motifs/sub-domains and expression profiles of *Sdr4L* genes in various tissues revealed wide functional divergence within the gene family. So far, PHS resistance attributable to a *Sdr4* gene has been demonstrated only in the monocotyledon species rice and wheat. In order to investigate evolutionary differences between dicot and monocot *Sdr4L* genes, we analyzed functional divergence and adaptive evolution at the amino acid level. Our results indicate that selective constraints and amino acid properties may have driven the molecular evolution of Sdr4-like (Sdr4L) proteins.

## Methods

### Identification of *Sdr4* gene family members from different plant species

*Sdr4* gene sequences from previous reports [8, 9] were used to blast (BLASTP and TBLASTN) the Phytozome database (http://www.phytozome.net). Genes showing similarities in structure to *Sdr4* with predicted BLAST values ≤ 1e–5 were collected as candidate genes. Unique *Sdr4L* genes were filtered by excluding partial and redundant sequences. We identified non-redundant *Sdr4L* sequences from all angiosperm land plants. Conserved domain analysis of Sdr4L proteins was conducted using the ProDom (http://prodom.prabi.fr/prodom/current/html/home.php) and the Pfam (http://pfam.sanger.ac.uk/) databases. Following the ProDom search, genes without typical domains (PD319905 and PDB0AWP) of OsSdr4 proteins were deleted from further analysis. Mw and pI were predicted by submitting Sdr4L proteins to the ExPASY database. The location and signal peptides of Sdr4 or Sdr4L proteins were determined using the Target P1.1 and Signal P4.1 servers (http://www.cbs.dtu.dk/services). The assembled information including accession numbers, nomenclature, chromosome and genomic positions, predicted domains of *Sdr4L* genes, and encoding proteins are shown in S1 Table and S2 Table.

### Multiple alignment, phylogenetic analysis, and gene structure prediction

Sequence alignment and phylogenetic analyses of the Sdr4L proteins were conducted using the Molecular Evolutionary Genetics Analysis (MEGA) 5.0 program [13]. an unrooted phylogenetic tree was built by the neighbor-joining (NJ) method [14] with the following parameters: pairwise deletion option, 1,000 replicates of bootstrap and Jones-Taylor-Thornton (JTT) model [15]. The topology of the tree was further validated by the maximum-likelihood and minimum-evolution methods, and results revealed similar topology with only minor changes at lower nodes (data not shown). The numbers of exons and introns in *Sdr4L* genes were manually calculated during retrieval of Sdr4L genomic sequences from the Phytozome database (http://www.phytozome.net) in Blast analysis. Conserved motifs between Sdr4L proteins were identified using the MEME program (http://meme.sdsc.edu) [16] with the following parameters: number of repetitions = zero or one, maximum number of motifs = 6, and optimum motif width constrained between 6 and 50 residues.

### Calculation of divergence time among *Sdr4*/*Sdr4L* genes

The DNA coding sequences of *Sdr4L* genes were aligned using the MUSCLE program [17] with the default parameters integrated in MEGA5. The divergence times between the *Sdr4L* members were calculated using calibration nodes from mono and dicot species *Oryza sativa–Zea mays* (31 ± 6 MYA) and *Malus*. *domestica–Citrus*. *sinensis* (106 ± 4 MYA) [18]. A sequence from *Chlamydomonas reinhardtti* used as an outgroup showed weak similarity to OsSdr4 protein in Blast analysis. Divergence time estimates were obtained using Bayesian Markov Chain Monte Carlo (MCMC) analyze is implemented in Beast 1.5.4 [19]. The analysis was carried out with the following parameters: relaxed molecular clock with an uncorrected log-normal distribution model for rate of variation, the HKY substitution model, four gamma categories and a Yule model of speciation. Three independent runs were carried out, each with 20 million MCMC generations and sampled every 1000^th^ generation. The rest of analysis was performed following previous reports [18]. The final tree was graphically visualized and produced using FigTree v1.3.1 software [19].

### Estimation of functional divergence

In order to investigate the functional divergence between *Sdr4L* gene clusters, the software DIVERGE2 [20, 21] was employed. The analysis was based on a maximum likelihood test, in which Type I (changes in site-specific shifts in evolutionary rate) and Type II (changes in site-specific shifts in amino acid physiochemical properties) functional divergence, θI and θII, between the Sdr4L subfamilies were estimated. θI or θII values significantly greater than 0 means that both Type-I and II functions could occur after gene duplication and/or speciation. In this analysis, critical amino acid site (CAAS) residues responsible for functional divergence are predicted using a site-specific profile based on posterior probability (Qk). Large Qk values indicate a high probability that the evolutionary rate and/or a radical change in amino acid properties at a site are different between any two clusters [20, 21].

### Tests of positive selection

Tests of positive selection were carried out by employing the CODEML program implemented in the PAML v4.4 software package [22]. Nucleotide and associated multiple protein sequence alignments of the *Sdr4L* genes were firstly obtained by PAL2NAL [23], and the resulting codon alignments and NJ tree were then used to estimate the non-synonymous substitution rate (dN)/ synonymous rate (dS) (or ω) ratio for each site and to determine various evolutionary models [24]. Two pairs of models were chosen to determine positively selected sites using the likelihood ratio test (LRT).

In the site-specific model, M0 (one ratio) and M3 (discrete) were compared, using a test for heterogeneity between codon sites in the dN/dS ratio value, ω. A second comparison was M7 (beta) *vs* M8 (beta+ω >1); this is the most stringent test for positive selection [25]. When the LRT predicted positive selection, the Bayes empirical Bayes (BEB) method was used to calculate the posterior probability that each codon is from the site class of positive selection under models M3 and M8 [26]. The branch-site model, assumes that the ω ratio varies between codon sites over a small number of branches in the phylogenetic tree. The tree divided into foreground (interest of gene cluster) and background branches (the remaining gene clusters). The ω values in these two branches were then compared by assignment to four predefined site classes. The first class of sites is highly conserved throughout the tree with 0 < ω0 < 1, the second class ω1 = 1 (codons that neutrally evolved throughout the tree), the third and fourth classes ω2a>1 (positive selection only on the foreground but is constrained to be under purifying selection on the background). When constructing the LRTs, the null hypothesis fixes ω2 = 1, allowing sites to evolve under negative selection of the background lineages being released from constraint, and to evolve neutrally on the foreground lineage. The alternative hypothesis constrains ω2 ≥ 1 [27, 28]. Based on the BEB method [27], the posterior probabilities of specific codons falling into a site class affected by positive selection are estimated.

### Homology model building and microarray data/ RNA-Seq atlas extraction

The homology models of Sdr4L subgroup proteins were generated with i-TASSER (http://zhanglab.ccmb.med.umich.edu/I-TASSER/) [29], in which structural templates are first identified from the PDB database by the multiple-threading program LOMETS; then the full-length models are built by iterative template fragment assembly simulations; and 3D model functions are predicted by comparing them with the protein function database BioLiP. The resulting models were prepared and visualized with Swiss-PdbViewer v4.1.0 (http://spdbv.vital-it.ch/), and predicted critical amino acid residues were marked onto the corresponding positions. The eFP Browser (http://www.bar.utoronto.ca/efprice/cgi-bin/efpWeb.cgi) tool was used to search the microarray data for available monocot (maize, rice and barley) and dicot (poplar, *M*. *truncatula*, soybean, potato, tomato, *Eutrema salsugineum*) species. A table of expression values from different tissues and development stages were extracted according to the species data source. These values were then used to build heat maps using Cluster v3.0 and Treeview [30].

## Results

### Identification of *Sdr4* genes and their homologues in plants

To investigate the origin and evolutionary history of the *Sdr4* gene family, we first retrieved the available *Sdr4* or *Sdr4L* sequences from currently sequenced and unfinished genomes; 54 *Sdr4* homologues were identified from 38 plant species representing both monocotyledonous and dicotyledonous plants using the Phytozome, JGI, TAIR, RAP, and BRAD databases (S1 Table). Two *Sdr4L* gene copies were identified in monocots *Sorghum bicolor* and *Brachypodium distachyon* and dicot species *Populous trichocarpa*, *Linum usitatissimum*, *Gossypium raimondii*, *Brassica rapa*, *Glycine max*, and *Malus domestica*. In monocot species, *Panicum virgatum* had the highest number of 7 *Sdr4L* members, followed by *Zea mays* with three. The predicted Sdr4L protein sequences from all species queried ranged from 280 to 449 amino acids. All *Sdr4* and *Sdr4L* gene sequences began with an initiation codon and ended with a stop codon. Most *Sdr4L* genes had no intron, except that monocots *Panicum*. *virgatum*, *Sorghum bicolor* and *Brachypodium distachyon*, and the dicot *Vitis vinifera* did contain introns in their coding sequences. Most Sdr4L proteins in monocots lacked N-terminal transit peptides with exceptions of PvSdr4L1-3, SiSdr4L, SbSdr4L2, and ZmSdr4L1. However, in dicots, except for AcSdr4L, the proteins seemed to lack N-terminal transit peptides. The pI of the Sdr4L proteins varied from 5.15 to 10.7, with the majority (79.6%) being alkaline. The molecular weights of Sdr4L proteins ranged from 28.5 to 47.9 kD. These large variations in amino acid sequence length and physicochemical properties of Sdr4L proteins suggest that they may have changed to fulfill different functions.

The searches were further expanded using the SMART, PFAM and ProDom programs to identify conserved domains. Since OsSdr4 proteins have no homology with protein sequences of known function, the SMART and PFAM failed to identify any conserved domains. The search in ProDom using OsSdr4 as a query showed that this protein contained two protein domains: PD319905 and PDB0A0W9 (S2 Table). The same pair of domains was identified when we used Sdr4 and Sdr4L proteins from other species. These domains were defined as ''repeat finger zinc'' (zinc finger repeats). In contrast, domain searches with selected Sdr4L proteins (for example, those from *Arabidopsis lyrata* and *Mimulus guttatus*) as queries showed various sets of protein domains along with the above two; we identified a total of six domains in ProDom (S2 Table). The final set of Sdr4L proteins was determined based on the presence of PD319905 and PDB0A0W9 observed in OsSdr4 as well as their corresponding score and expected e-values regardless of the presence of other domains. In this way, we filtered candidate Sdr4L proteins for further analysis (S2 Table).

### Phylogenetic relationships among Sdr4 and Sdr4L proteins

**Fig 1:**
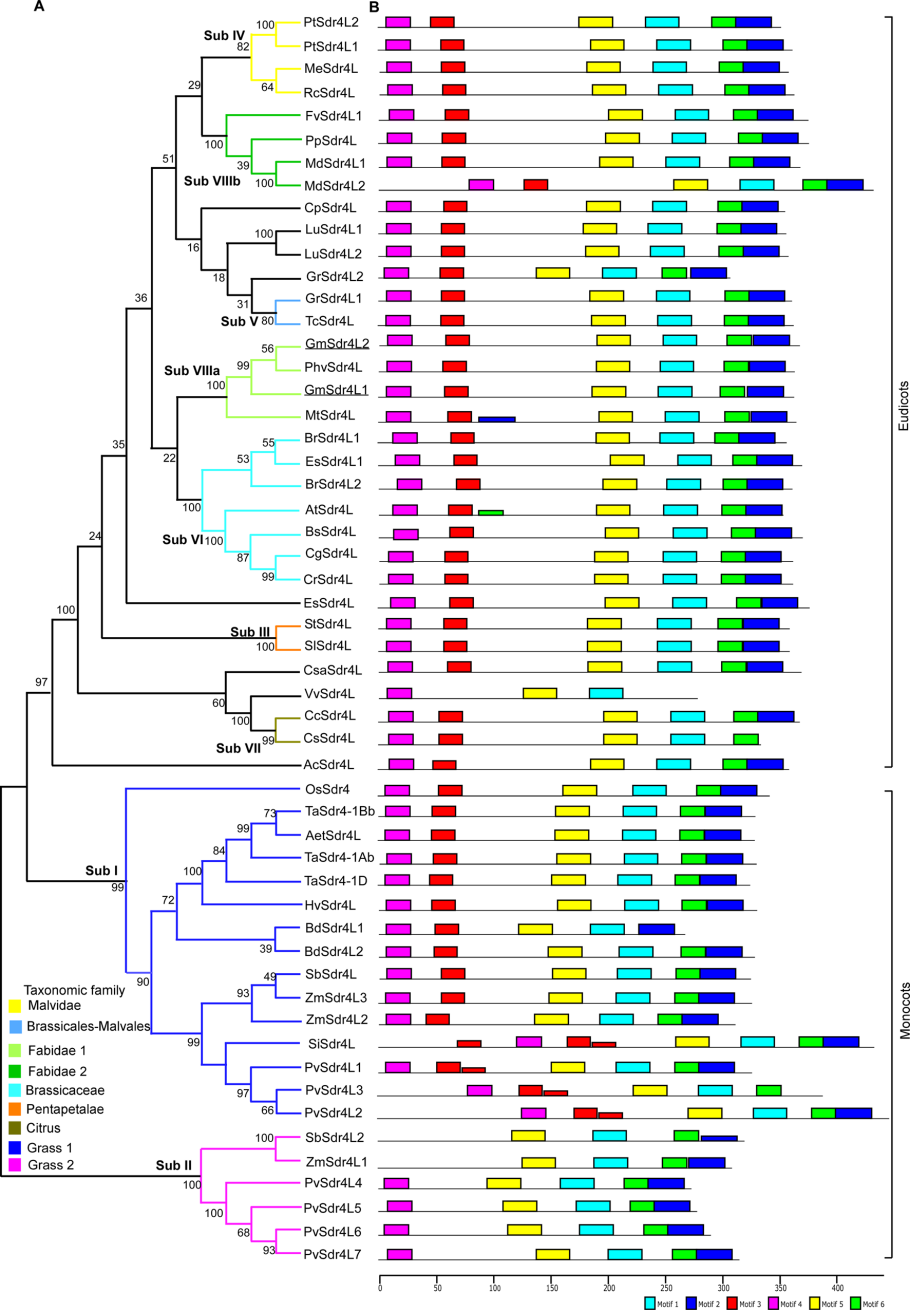
Phylogenetic tree showing relationships between Sdr4L protein sequences. (A) The unrooted tree was built with MEGA5 by using sequence alignments of 54 Sdr4L members. The branch support bootstrap values were obtained by 1000 replicates using the NJ method. (B) Proteins showing conserved motifs obtained by MEME analysis are indicated in numbered colored boxes.

To investigate evolutionary relationships among Sdr4 and Sdr4L proteins, we ran sequence alignment of 54 full-length amino acid sequences and built an unrooted phylogenetic tree using the neighbor-joining (NJ) algorithm (Fig 1A and S1 File).

The topology of the tree was further confirmed by constructing a Minimum evolution (ME)/Maximum likelihood (ML) tree. Both trees showed similar topologies, with clearly distinguished monocot- and dicot-specific clades (Fig 1A). Based on topology and duplication nodes of the Sdr4L paralogs in the NJ tree, the monocot-specific clade divided into two subclades based on bootstrap support values of 90 and 100; the Grass 1 (I) and Grass 2 (II) subclades (Fig 1A). The dicot-specific clade was divided into six subclades, based on bootstrap support values from 80 to 100. We designated these subclades as Sub III (*Pentapetalae*), Sub IV (*Malvidae*), Sub V (*Brasicales-Malvales*), Sub VI (*Brasicaceae*), Sub VII (*Citrus*), and Sub VIII a-b (*Fabidae* 1–2) in the phylogenetic tree. Some Sdr4L proteins, that did not fall into clusters or within acceptable bootstrap value ranges were left as unresolved. Our analysis suggested that *Sdr4* or *Sdr4L* genes from the same lineage tended to cluster together in the phylogenetic tree.

A MEME (Multiple Em for Motif Elicitation) search [16] for conserved protein motifs flanking the Sdr4L proteins was conducted (Fig 1B) to determine possible mechanisms for structural evolution of *Sdr4L* genes. As shown in Fig 1B, six different types of motifs were found (S1 Fig). The type, order and motif numbers were similar in proteins of the same subfamily, but differed between subfamilies. In the monocot specific-clade, the distribution of Motif 3 corresponded closely to phylogenetic relationships. Motif 3 was absent from Sub II members, but was present in all members of Sub I where a few proteins had two copies of Motif 3 (SiSdr4L1 and PvSdr4L1-3). Motif 4 was missing in some cases within Sub II (SbSdr4L2 and ZmSdr4L1). Motif 3 was predicted to be putative nucleotide localization signal (RARKR) region by cNLS mapper [31]. Within the dicot-specific clade, almost all subfamily members conserved all motifs, except for Sub VII, where Motif 2 (CsSdr4L) was missing. Motifs 2, 3 and 6 were missing in VvSdr4L causing a different gene structure in regard to intron-exon relationships and explaining why they formed an undefined cluster in the tree. Motifs 2 and 6 occurred twice in AtSdr4L (sub VI) and MtSdr4L (sub VIIIa). From these analyses, differences in motif distribution in different groups or subgroups of Sdr4L protein indicated that the functions of these genes might have diverged during evolution.

Analyses of exon-intron structures in gene families often helps in understanding their evolutionary histories. We investigated the exon-intron structure of individual *Sdr4L* genes in all lineages using the online Gene Structure Display Server [32]. The predicted numbers of exons and introns in the *Sdr4* gene family is shown in S1 Table. Among 54 *Sdr4* or *Sdr4L* genes, 47 had no intron according with previous reports [8], and suggesting that these *Sdr4* genes were conserved. However, several monocot species (for example, *B*. *distachyon* and *P*. *virgatum*) and one dicot (*V*. *vinifera*) contained introns. Of two genes from *B*. *distachyon*, *BdSdr4L1* possessed a single intron. While the *Sdr4L* genes from *P*. *virgatum* were very different from those in other monocot species, five of seven genes had more than one intron, including one *PvSd4r4L* gene with three introns, two *PvSd4r4L* genes with two introns, and two *PvSd4r4L* genes with one intron (S1 Table). In addition, two *PvSdr4L* genes contained no intron, indicating that the ancient *PvSdr4L* genes may have had three introns, but gradually lost them during evolution. Finally, some *PvSdr4L* genes lacked introns (*PvSdr4L1* and *L7*) or retained a single intron (*PvSdr4L4*). Similarly, *V*. *vinifera* (*VvSdr4L*) seemed to have a complex gene structure with four introns, a possible reason that VvSdr4L protein was not in the clade with their lineage cluster (Sub III of *Pentapetalae*). Together, these results showed that Sdr4L proteins can be classified into eight subgroups (Sub I-VIII), and this classification was supported by the position and presence or absence of conserved motifs. Most *Sdr4L* genes had a similar intronless structures, except for *P*. *virgatum* and *V*. *vinifera*, indicating that the conserved intron structures in these species were necessary for the regulation of *Sdr4L* expression.

### Expansion and timeline of *Sdr4L* evolution in higher plants

**Fig 2:**
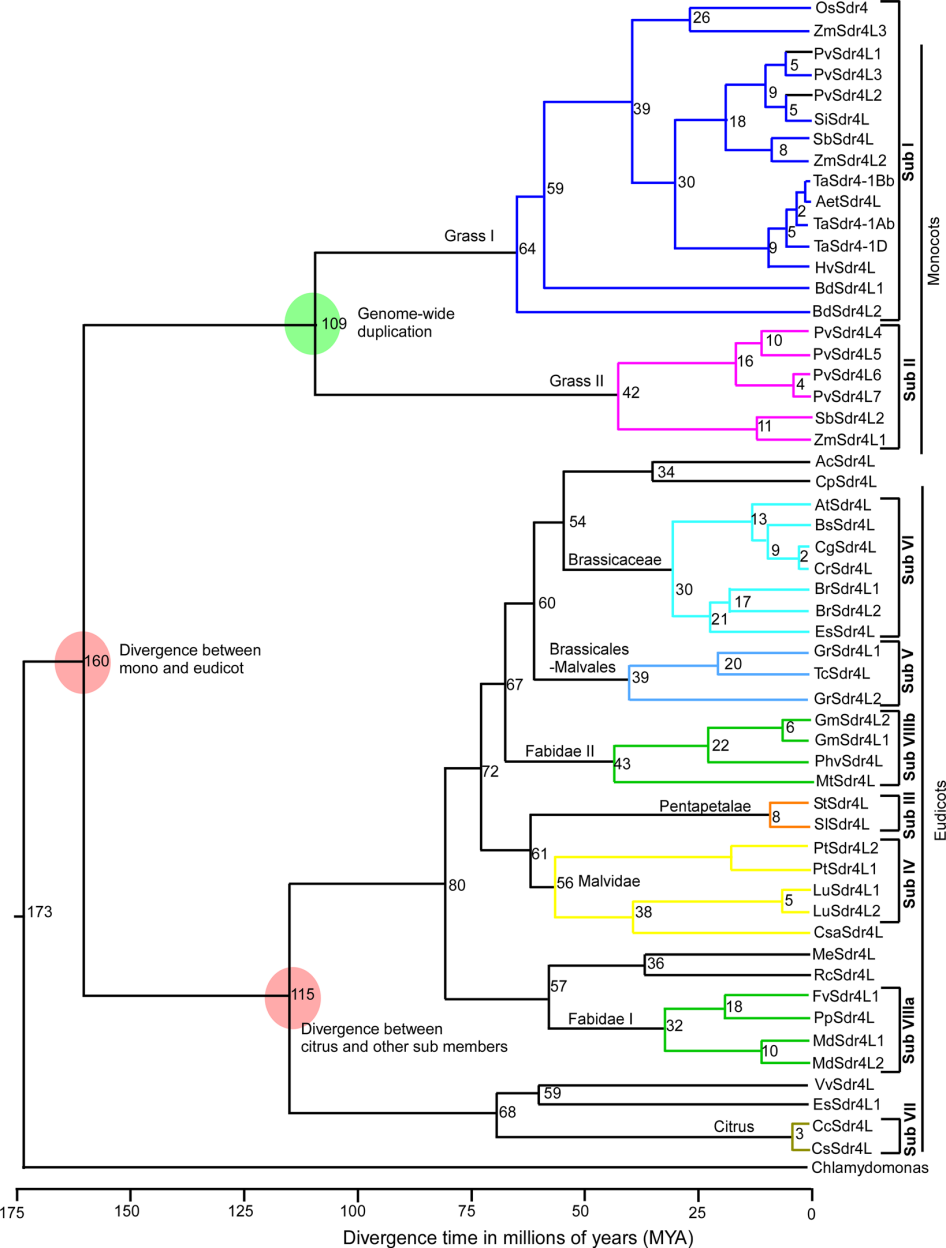
Divergence time estimations between *Sdr4L* genes in plants based on aligned nucleotide sequences using Bayesian MCMC analysis. A relaxed molecular clock approach with 20 million MCMC steps was used. Tree was drawn using the divergence of *O*. *sativa* and *B*. *distachyon* and *M*. *domestica* and *C*. *sinensis* as anchor points for monocot and dicot lineages, respectively. Number at each node represent estimated time in million years (MYA). The light pick and green circle shows the events of divergence between mono and dicot lineages and duplication in grass genomes, respectively. The major clusters of orthologous genes are shown in different colors and defined in different subgroups (I to VIII).

Gene and genome duplications that play a role in evolution of novel gene function are widespread among gene families [33]. Our results indicated that *Sdr4* or *Sdr4L* genes from different species did not share a common expansion model. Tandem duplications were common in the monocots *B*. *distachyon* and *P*. *virgatum*, but not in *Z*. *mays* and *S*. *bicolor*. Only segmental duplications were identified in the dicots *P*. *tirchocarpa*, *G*. *max* and *G*. *raimondii* (Fig 1A and S1 Table), suggesting that different subfamilies underwent different expansion patterns. To estimate the approximate ages of the duplication events, a Bayesian Markov chain Monte Carlo MCMC analysis with a relaxed molecular clock approach based on aligned nucleotide sequences of *Sdr4L* genes was undertaken. The evolutionary history of *Sdr4L* genes was calculated across monocots and eudicots, showing multiple duplication events (Fig 2).

The ancestral *Sdr4L* gene in early monocots and dicots must have undergone divergence, about 160 million years ago (MYA), to generate the gene families of *Sdr4L* in monocots and dicots. As dicots did not undergo any major events until speciation of Sub VII (*citrus*) and other dicot family members from ~ 115 MYA, this must have contributed to produce various orthologs of *Sdr4L* genes. Afterwards, *Sdr4L* must have undergone a duplication event ~ 109 MYA to produce two isoforms of *Sdr4L* genes in the monocot-specific clade. As shown in Fig 2, further divergence corresponded with speciation. Our analysis further demonstrated that both in monocots (*PvSdr4L*, *ZmSdr4L*, and *SbSdr4L*) and dicots (*PtSdr4L*, *MdSdr4L*, *GmSdr4L* and *LuSdr4L*), duplications (either tandem or segmental) in each *Sdr4L* subfamily were mostly conserved once they occurred. Moreover, these duplicated genes belonged to the same subfamilies, suggesting that they did not undergo further divergence.

### Functional divergence in the *Sdr4* gene family

Significant changes in Type-I (shifted evolutionary rate) and Type-II (altered amino acid physiochemical properties) functional divergence after the emergence of paralogous sequences were estimated using statistical methods [20] implemented in DIVERGE2 [21]. Posterior probability (Qk) of divergence at each site was calculated to predict the location of certain critical amino acid sites (CAAS) [34] that are highly relevant to functional divergence. The advantage of these methods is that they use amino acid sequences, and consequently are not sensitive to saturation of synonymous sites [34]. The analysis was based on the NJ tree built from a multiple clustal alignment of 54 Sdr4L protein sequences. In this analysis subfamilies with less than four sequences (Sub III, Sub V and Sub VII) were excluded since they cannot be analyzed. We performed pairwise comparisons among the remaining subfamilies (Sub I, II, IV, VI, VIIIa, and VIIIb) to estimate the rate of amino acid evolution at each sequence position.

As shown in Table 1, the coefficient of Type I functional divergence (θI) between any two relevant clusters was significantly greater than 0. The functional divergences between subfamily pairs I/II, I/VIIIb, II/VI, II/VIIIb, II/IV, VI/VIIIb were statistically significant (θ > 0; likelihood ratio test statistic > 6.147; *P* < 0.01), indicating that significantly different site-specific shifts in evolutionary rate may have taken place at certain amino acid sites between these pairs. Type II functional divergence (θII) between all subfamilies, except Sub IV and VIIIa, were noted, was indicative of a radical shift in amino acid properties (Table 1). These results demonstrated that the functional evolution of *Sdr4L* gene subfamilies in both monocots and dicots may have adopted varying degrees of Type I and Type II functional divergence. In addition, critical amino acid sites responsible for functional divergence between the subfamilies were identified based on appropriate posterior probability (Qk) values derived from each comparison. To avoid too many residues, Qk > 0.9 was used as the cutoff for identifying both Type I and Type II functional divergence-related residues in all comparisons between the Sdr4L subfamilies (Table 1, S3 Table). A total of 56 and 64 CAAS were predicted in PD319905_1-240_ (domain I) and PDB0A0W9_241-325_ (domain II) (referring to the rice OsSdr4 protein) by Type I and Type II functional divergence analyses, respectively. The results further suggested distinct variation in the number and dispersion of sites for functional divergence within each pair. For example, for Type I functional divergences, 27, 13, 33, 36, and 5 CAAS were predicted for subfamily pairs I/II, II/VI, II/IV, and II/VIIIa, respectively, whereas only one site was identified for pairs I/VIIIb and VI/VIIIb. The rest of the subfamilies had no CAAS as indicated by statistical non-significance. The identified number of CAAS for Type II was higher than Type I. The number of CAAS for each subfamily pair ranged from 0 to 50 (Table 1, S3 Table). Moreover, most of the Type II CAAS sites for each subfamily group belonged to corresponding Type I sites. Some of the subfamily comparisons (I/VI, I/VIIIb, I/VI, I/VIIIa, VI/VIIIb, VI/IV, VI/VIIIa, VIIIb/IV, and VIIIb/VIIIa) indicated zero or one CAAS for Type I functional divergence, but there were more than 10 CAAS for Type II functional divergence, particularly I/VI, I/IV, and I/VIIIa, I/VIIIb, VI/VIIIb and VIIIb/VIIIa containing 32, 38, 30, 25, 21, and 10 CAAS, respectively. This suggested that these subfamilies were undergoing functional divergence as well as shifts in evolutionary rate. It should be noted that several pairs left did not follow the model, indicating that site-specific shifts in evolutionary rate and changes in amino acid properties did not act uniformly on the Sdr4 subfamily members over evolutionary time.

**Table 1:** Functional divergence between Sdr4L subfamilies.

Group 1	Group 2	Type-I			Type-II	
		Θ±SE	LRT	Qk > 0.9	Θ±SE	Qk > 0.9
I	II	0.759±0.148	26.346**	27	0.158±0.146	32
I	VI	0.334±0.193	0.003	0	0.195±0.105	32
I	VIIIb	0.459±0.185	6.147*	1	0.281±0.094	38
I	IV	0.064±0.164	0.153	0	0.188±0.108	30
I	VIIIa	0.214±0.248	0.746	0	0.148±0.110	25
II	VI	0.787±0.254	9.590**	13	0.272±0.129	50
II	VIIIb	0.839±0.207	16.420**	33	0.289±0.115	43
II	IV	0.944±0.238	15.718**	36	0.163±0.138	47
II	VIIIa	0.615±0.336	3.356	5	0.151±0.137	45
VI	VIIIb	0.642±0.211	9.257**	1	0.188±0.072	21
VI	IV	0.001±0.022	0.000	0	0.014±0.083	4
VI	VIIIa	0.178±0.313	0.325	0	0.009±0.081	5
VIIIb	IV	0.174±0.146	1.416	0	0.006±0.071	2
VIIIb	VIIIa	0.383±0.205	3.499	0	0.033±0.067	10
IV	VIIIa	0.001±0.022	0.000	0	-0.076±0.078	0

Note: θI and θII, the coefficients of Type I and Type II functional divergence between two gene clusters; LRT, likelihood ratio Statistic

*, *P* < 0.05

**, *P* < 0.01

Qk, posterior probability. Large Qk values indicate a high probability of functional constraint (or the evolutionary rate) or that physicochemical properties of a given amino acid site differs between two clusters.

Compared with Sub I/IV, I/ VI, I/VIIIa and I/VIIIb, Sub II/IV, II/ VI, II/VIIIa and II/VIIIb had relatively larger coefficients of functional divergence (θI & θII) as well as more CAAS related to functional divergence. This suggests that Sub II/IV, II/ VI, II/VIIIa and II/VIIIb are relatively more significant than sub I/IV, I/ VI, I/VIIIa and I/VIIIb. The motif analysis also showed that the Sub II of Grass 2 has a clearly different motif organization compared to the other subfamilies in both monocot- (Sub I of Grass 1) and dicot-specific clades (Fig 1A). Most of the CAAS (78 of 110) were present in C-terminal domains, that is, domain II (PDB0A0W9_241-325_) of OsSdr4L protein, suggesting that these sites were important for subgroup-specific functional evolution of *Sdr4L* genes after the split of monocots and dicots. However, there was no functional characterization of any of the Sdr4 or Sdr4L proteins. The analysis suggest that, due to different evolutionary rates at some amino acid sites, *Sdr4L* genes in both monocots and dicots have significantly diverged in functions.

### Positive selection in the *Sdr4*/*Sdr4L* gene family

To test for positive selection in the Sdr4L subgroups, likelihood ratio tests were implemented in PAML v4.4 software using a site-specific model [22]. Estimation was performed based on NJ tree topology (Fig 1A) using two pairs of models, M0/M3, and M7/M8. Comparison between models yields the variation in ω (non-synonymous (dN or Ka) *versus* synonymous (dS or Ks) mutation ratios among amino acid sites. Based on the Ka/Ks ratio, genes subjected to neutral (1), negative (<1) and positive (>1) selection are determined [24]. The results of parameter estimates and log-likelihood values for selected models are shown in Table 2.

**Table 2:** Tests for positive selection among Sdr4L codons of using site models.

Model	InL	2ΔL	Estimates of parameters^a^	Positively selected sites^b^
M0 (one ratio)	-8461.241		ω = 0.16456	None
M3 (discrete)	-8244.734	433.014 (M0 vs M3)	p0 = 0.55168, p1 = 0.33358, p2 = 0.11474, ω0: 0.05766, ω1:0.23719, ω2: 0.66151	None
M7 (beta)	-8240.96		p = 0.73100, q = 2.96832	Not allowed
M8 (beta & ω)	-9418.204	1177.244 (M7 vs M8)	p0 = 0.99999, p = 0.36186, q = 1.15287, p1 = 0.00001, ω = 2.06669	**5Q**, 74 P**, 77 P*, 78 P, 158V**, 174 E*, **186 H**, 193 D**, 194 V*, **195 G**, 203 A*, 204A**, 205A**, 206 P*, **249 P**, 302 S**, 303 S*

Note

^a^ Number of parameters in the ω distribution (ratios of non-synonymous (dN or Ka) *versus* synonymous (dS or Ks) mutations).

^b^ Positively selected sites are inferred from posterior probabilities > 95% (*) or 99% (**).

Sites implicated in Type I and Type II divergence are shown in bold. Codon (amino acid) positions are based on rice OsSDR4 protein.

In the first model pair (M0/M3), M0 is the one-ratio model that assumes one ω ratio at all sites. Under this model, the estimated ω value is 0.16456 for *Sdr4L* with the log-likelihood score ℓ = -8461.241, whereas in the discrete model (M3), the probabilities (p0, p1, and p2) of each site were submitted to purifying, neutral, and positive selection, respectively, and their corresponding ω ratios (ω0, ω1, and ω2) were inferred from the data. In contrast to M0, M3 was better, in which likelihood-rate test statistic 2Δℓ = 433.014 indicated a statistically significant result (*P* < 0.01), reflecting higher selective pressure on the *Sdr4L* gene family. This suggested that the *Sdr4L* sequence has undergone strong positive evolutionary selection. None of the codon sites were identified during comparison of models M3 and M0. Thus the additional M7 (Beta model, a null test assuming a Beta distribution with ω between 0 and 1) and M8 (Beta & ω model, add one extra class with the same ratio ω >1) tests were performed. The comparison M7 *versus* M8 revealed 17 different codon sites that appeared to be under the influence of positive selection, for which the likelihood rate test statistic 2Δℓ = 1177.244 greatly exceeded the critical value, indicative of strong positive selection. Based on Bayes Empirical Bayes (BEB) analysis of the M8 model, 17 candidate amino acid sites (5 Q, 74 P, 77 P, 78 P, 158 V, 174 E, 186 H, 193 D, 194 V, 195 G, 203 A, 204 A, 205 A, 206 P, 249 P, 302 S, and 303S) under positive selection were identified (Table 2). Among these amino acids, 15 were implicated in both Type I and Type II functional divergence, the exceptions being 78 P and 158 V.

The branch-site model was also performed to investigate the adaptive evolution of Sdr4L subfamilies. Based on the Sdr4L phylogenetic tree (Fig 1), nine branches were independently considered as the foreground branch, whereas the rest of the branches were considered to be backgrounds branches. The results of estimates and log-likelihood values under the branch-site models are listed in S4 Table. A few notably significant codon sites were identified in five of nine Sdr4L subfamilies (S4 Table). Although, significant positive selection was indicated when Sub I and VIIIb were defined as foreground branches. In Sub II, 11 sites were predicted as positively selected sites when branch I was considered to be the foreground branch and six of them (3 M, 13 A, 16 I, 20 F, 59 R, and 186 H) had a posterior probability higher than 0.95. In Sub VIIIa, three sites (184 T, 210 E, 218 S) were detected at P > 0.70 significance level. When Sub I and VI were chosen as foreground branches, only one site (241 R and 210 E) in each case was identified at >90 and >95 significance, respectively. Finally, for Sub VII two sites (57 Q, 63 A) were found at a posterior probability >50. These results suggested that Sub II might have been under stronger positive selection than other subfamilies as the highest number of statistically significant positive sites was predicted in this subfamily. In addition, all the amino sites from the branch sites analysis were also responsible for Type I and Type II functional divergence and only two sites were implicated in the site model of positive selection (74 P and 186 H) (Table 2 and S4 Table).

**Fig 3:**
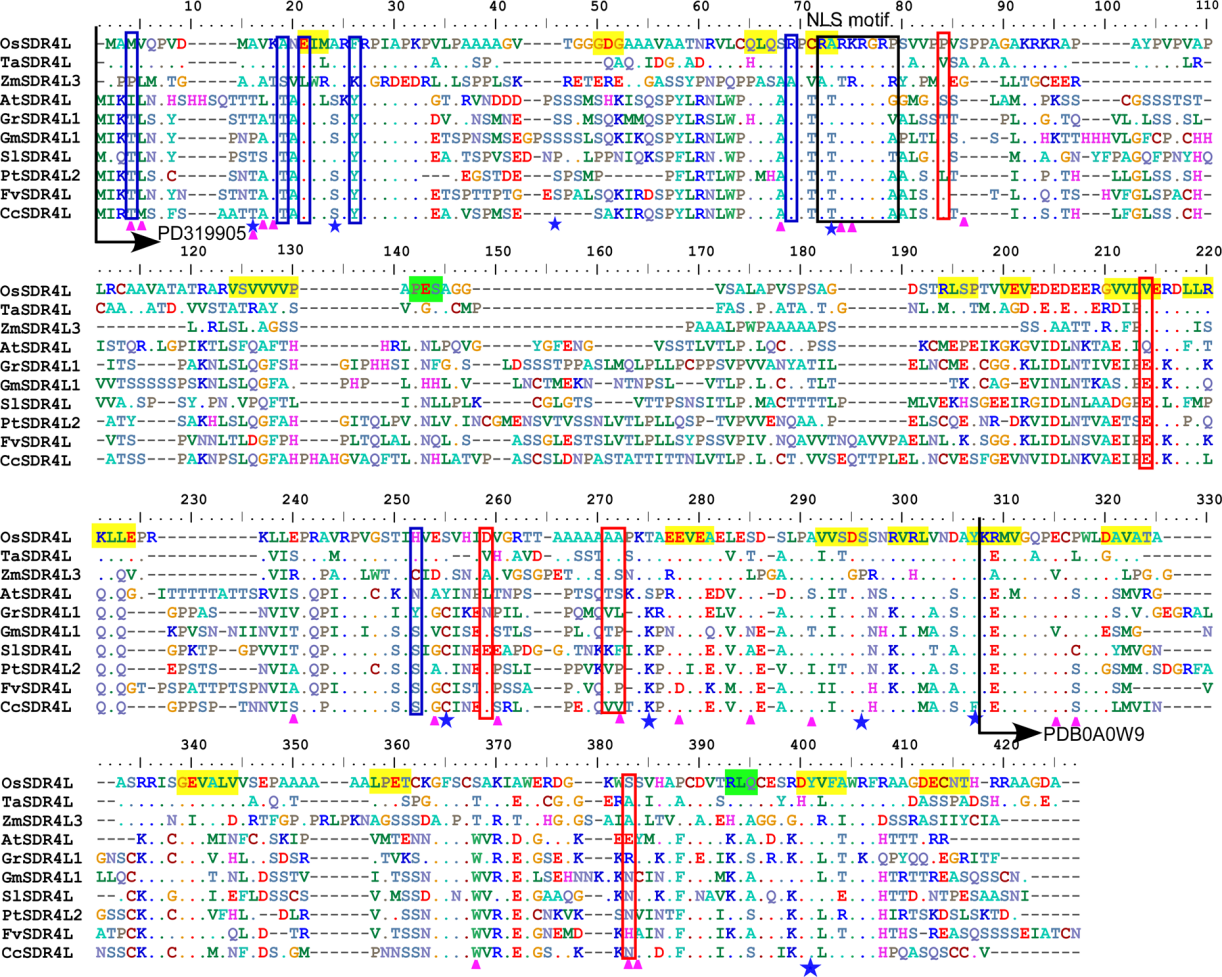
Sequence comparison by multiple clustal alignment of typical Sdr4L protein sequences from monocot and eudicot plant species. Black arrows indicate predicted protein domains (PD319905 and PDB0A0W9). Critical amino acid sites (CAAS) responsible for functional divergence (Qk > 0.9, Type I and Type II) and phosphorylation sites are marked by pink triangles and blue stars, respectively. Adaptive selection sites for the site and branch site model are indicated by red and blue boxes, respectively. Putative nuclear localization signal (NLS) motifs are boxed.

The number of amino acid sites responsible for both positive selection and functional divergence, in which 27 CAAS had an influence on both positive selection as well as Type I and Type II functional divergence in the evolution of *Sdr4L* genes was estimated (S2 Fig). All 27 sites were located in multiple sequence alignments (Fig 3 and S3 Fig).

**Fig 4:**
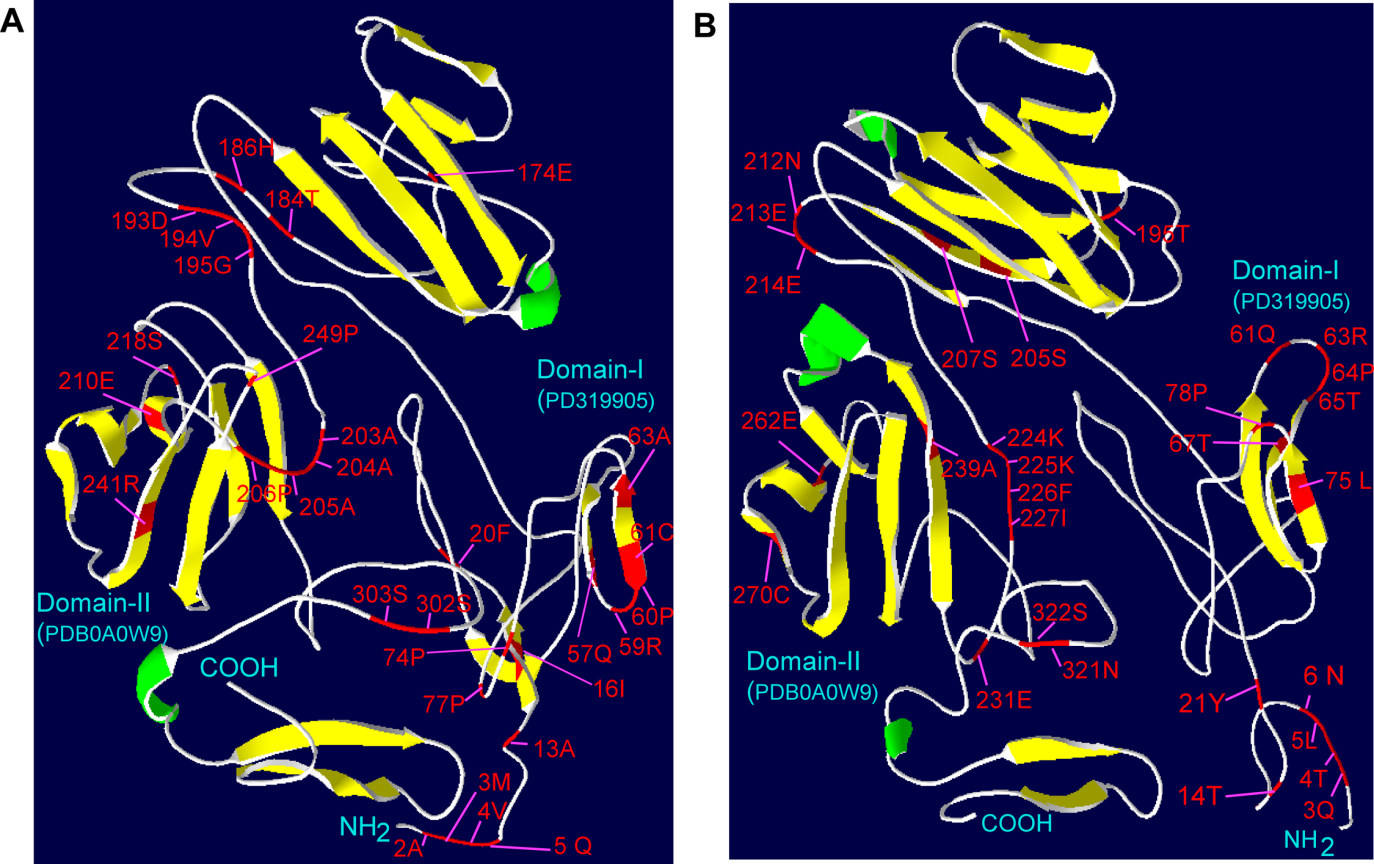
**Three dimensional structures of rice OsSdr4L (a) and tomato SlSdr4L (b) proteins. The corresponding structures were obtained using i-TASSER** [22]. The α-helices and β-strands are shown in green and yellow, respectively. Critical amino acid sites responsible for both positive selection and functional divergence are labeled in red. The edges of amino (N) and carboxyl (COOH) terminals, and random view of predicted domains of I and II (PD319905 and PDB0A0W9) are indicated in light blue.

Among these, one (249 P), two (302 S, 303 S), five (55 Q, 59 R, 60 P, 61 C, 63 A), three (13 A, 16 I, 20 F), three (174 E, 184 T, 186 H) and zero amino acids were located in motifs 1, 2, 3, 4, 5 and 6, respectively (S3 Fig). Further we labeled these 27 CAAS on a three dimensional (3D) structure of OsSdr4 (rice) and SlSdr4L (tomato) built using i-TAASER [29] through homology modeling (Fig 4).

The predicted 3D models suggested that the basic structure of Sdr4L proteins was almost conserved among plant species with some minor variations in the number of β-sheets, α-helices and loops. The 3D model of OsSdr4L revealed that there were 18 sheets, 21 loops and two helices, in which most of the amino acid sites were distributed on the loops and only several sites (13 A, 16 I, 57 Q, 61 C, 63, 241) were distributed on the sheets (Fig 4). However in the case of SlSdr4L, it was found 20 sheets, 24 loops and three helices. Similar to OsSdr4L, SlSdr4L also possessed critical amino acid sites mostly in loops except several sites (67 T, 75L, 205 S, 207 S, 239 A, and 270 C) which were present on the sheets. The minor changes in number of helices, sheets and loops indicates the possible existence of structural divergences among Sdr4L proteins. The observed critical amino acid sites in the loops and sheets might also act as a major evolutionary force driving the divergence of Sdr4-conserved motifs and further indicating the functional importance of these regions for this protein family. However in the present study, 3D structures of Sdr4L was built based on homology model. In order to understand the functional variations and to evaluate the importance of the critical amino acid sites, a detailed experimental validations are required further.

Phosphorylation sites play an important role in a variety of cellular processes such as the cell cycle and signaling transduction in addition to the structural and functional state of plant proteins [35, 36]. We predicted that any possible serine, threonine and tyrosine phosphorylation sites in Sdr4L proteins and calculated the average number of phosphorylation sites in Sdr4L proteins from the subgroups noted in the phylogenetic analysis. The number of serine (S) phosphorylation sites in dicot-specific Sdr4L proteins was significantly higher than in the monocot proteins (S2 File). In Sdr4L proteins, some of the conserved and variable amino acid sites suspected to be under phosphorylation, were marked in the multiple alignments (Fig 3). For example, site 24 is located in the N-terminal loop (Fig 3) in dicot Sdr4L proteins (AtSdr4L, GrSdr4L, GmSdr4L, PtSdr4L, FvSdr4L, and CcSdr4L), and the residue at the corresponding site is serine (S), predicted to be a conserved phosphorylation site with high probability (score = 0.9; S2 File), whereas in monocot (OsSdr4, TaSdr4 and ZmSdrL), the site is occupied by alanine (A) with different physiochemical properties (Fig 3). Similarly, a threonine (T_63_) phosphorylation site in the NLS motif was conserved with high probability in dicots, whereas the corresponding site was alanine (A_63_) in monocots. Therefore, positive selection in these regions may have acted as a major determinant driving the functional divergence of Sdr4L proteins between monocot and dicot plant species.

### Expression patterns of *Sdr4L* genes in higher plants

The expression profiles of homologous *Sdr4L* family genes in subgroups were investigated using the available microarray data and RNA-Seq atlas of several plant species (rice, maize, barrel medic, Arabidopsis, soy bean, tomato and poplar) as shown in S4 Fig. All the probeset IDs of species were present in the data source except for *Z*. *mays* GRMZM2G038991 which lacked expression information in the Sekhon Atlas [37]. Since the gene copies of *Sdr4L* were one or two, we made expression profiles for 11 *Sdr4L* genes by heat map construction. According to our results, the levels of *Sdr4L* expression differed in different tissues or organs. Expression of most of the *Sdr4L* genes in reproductive parts indicated that these genes contribute to plant developmental processes. However, *Sdr4L* genes in certain species showed preferential expression, and their transcript abundances were observed only in seeds. For example, *O*. *sativa*, *Z*. *mays*, *G*. *max*, *M*. *truncatula* and *A*. *thaliana* showed much higher expression in seeds, or during the seed developmental stages, than in other organs, indicating that these *Sdr4L* genes function as dormancy-specific proteins, and are limited to seed organs as noted for rice in a previous study [8]. For species *S*. *lycopersicum*, *S*. *tuberosum* and *P*. *trichocarpa*, there was no expression data for seed tissue, but there were data for fruits, flowers and tubers along with vegetative parts such as root and leaves. In *S*. *lycopersicum* and *S*. *tuberosum*, expression was mostly in fruit tissues, and the mesocarp, and in *P*. *trichocarpa* expression was highest in seedlings and flowers (catkins). Further, among three segmentally duplicated gene pairs, the *Z*. *mays* (GRMZM2G396402 [ZmSdr4L3] and GRMZM2G038991 [ZmSdr4L1]) and *G*. *max* (Glyma17g35260 [GmSdr4L1]) and Glyma14g09910 [GmSdr4L2]) pairs retained the same seed-specific expression patterns, whereas the *P*. *trichocarpa* (Potri.014G015300 [*PtSdr4L2*] and Potri.002G117700 [*PtSdr4L1*]) pair showed the most abundant expression in seedlings and flowers, respectively, indicating a divergence in expression after gene duplication.

Changes in expression patterns are often influenced by the presence or absence of *cis*-regulatory elements in the promoter regions [8]. We performed a motif search using the PlantCARE database [38] to identify putative *cis*-elements in 1500 bp promoter sequences upstream of the initiation codon of all *Sdr4L* genes. The occurrence of *cis*-elements in all *Sdr4L* genes is shown in S5 Table. We identified potential regulatory elements associated with transcription factor-binding sites, including ABA-response elements [ABRE; ACGTGG/T(C)], RY repeats (CATGCA, core binding sites of the ABI3/VP1 family transcription factor) and an ABRE-related coupling element (CE).

The presence of these elements in promoter regions of seed maturation-related genes is very common [8, 39]. Apart from these elements, other *cis*-acting motifs such as light responsive (ACE, AE-box, CE3 and G-Box), endosperm-specific (Skn, GCN) and flowering (Circadian) responsive were also identified. Most of the *Sdr4L* genes contained the seed- or endosperm-specific element of the RY repeat and an ABRE hormonal responsive element. Both are considered important elements for induction of seed dormancy by maturation-related genes [8]. The RY element was present in both monocot and dicot specific *Sdr4L* genes, averaging 75% and 62.5%, respectively, whereas the average numbers of other elements were 18.8% (CE), 100% (ABRE), 62.5% (ACE), 37% (AE), 93.8% (G-box), 93% (methyl-jasmonate), 37.5% (GCN), 87.5% (SKN), 56.3% (circadian) for monocot species, and 0% (CE), 90.63% (ABRE), 53% (ACE), 28% (AE), 90.63% (G-box), 68.8% (methyl-jasmonate), 34.4% (GCN), 78.13% (SKN), 56.13% (circadian) for dicot species-specific *Sdr4L* genes (S5 Table). In short, the monocot *Sdr4L* genes seem to possess slightly higher numbers of *cis*-regulatory motifs in the promoter regions than the dicot species, particularly RY, ABRE and CE. However, we found no clear evidence of a correlation between the presence or absence of these elements and *in*-*silico* differential expression profiles of 11 *Sdr4L* genes.

## Discussion

### Comparative genomic analysis of *Sdr4L* gene families

Genome sequences of *Sdr4* and *Sdr4L* genes from various plant species provide a large amount of data that can be used to explore functional diversity from multiple perspectives. The current study identified 54 *Sdr4*/*Sdr4L* genes in 38 plant species, and there was a minimum of one *Sdr4L* gene in each species (S1 Table). An unrooted phylogeny analysis based on rice *OsSdr4* and homologous genes from several dicots, identified separate mono- and dicot-specific clades, similar to a previous phylogenetic analysis [8]. The phylogenetic tree (Fig 1A and S1 File) of *Sdr4L* genes again showed distinct monocot- and dicot-specific clades, which sub-divided into eight major sub families, sub I-VIII, supported by high boot strap branch values (80–100) and highly conserved motif positions, although several species that lacked branch support values were left as unresolved or undefined. Similar constraints were reported in phylogenetic analyses of large numbers of substitutable residue variations among gene sequences and conserved motifs [40]. Although a minimum of one *Sdr4L* gene was present in each species, and the highest number of gene copies occurred in grasses with seven and three copies in *P*. *virgatum* and *Z*. *mays*, respectively. This might be due to polyploidy [41].

Previous reports suggested there were several copies of *Sdr4* genes in Arabidopsis [8, 9]. However, we detected only a single copy of *Sdr4L* in that species based on domain and sequences characteristics as some Arabidopsis sequences did not contain expected domains (S1 and S2 Tables). Similarly, we omitted an *Sdr4L* gene from *B*. *distachyon* (Bradi4g39520). However, we found an extra copy of *Sdr4L* in *Z*. *mays* (GRMZM2G038991) and *S*. *bicolor* (Sobic.001G326000). Our analysis of motif composition of Sdr4L proteins also revealed interesting features of this family in different plant species. Sdr4L members from closely related lineages are clustered together in the phylogenetic tree and have common conserved motif compositions, indicating functional similarities among Sdr4L proteins within the same subfamily. Sub II in grass family members showed entirely different motif compositions compared to other family members (Fig 1). Therefore, Sub II members may have different functions than to OsSdr4 in Sub I grass family members. These results will require confirmation in future studies. Gene structure is considered to be important for structural evolution of genes. A large proportion of the *Sdr4L* genes (48 of 54, or 88.9%) were intronless, as was the *OsSdr4* gene [8]. Intronless genes are generally characteristic of prokaryotes and single exonic-gene families, suggesting to be evolved by gene duplication [42, 43]. Four *Sdr4L* genes from *P*. *virgatum* had different numbers of introns, suggesting that evolution of introns in *PvSdr4L* genes was a diverse and complex.

### Expansion pattern of the *Sdr4L* gene family

Gene duplication events (segmental, tandem, and transposition) are important in the evolution of gene families and leads to new members with functional variation [44, 45]. Tandem and segmental duplication events were responsible for expansion of the *Sdr4L* gene family in both monocots and dicots, respectively. Similar kinds of duplication events underlying gene clusters in angiosperm species were reported recently [46]. The phylogenetic tree constructed from the Bayesian MCMC analysis clearly showed two distinct clades as in the NJ tree corresponding to the monocots and dicots (Fig 2). According to previous reports, a whole-genome duplication (WGD) event must have occurred in an angiosperm ancestor ~150–270 MYA [18, 47]. This suggested that the earliest proliferation of *Sdr4L* genes in angiosperms occurred after the monocot-dicot split approximately ~160 MYA (Fig 2). We also found that a genome-wide duplication event ~109 MYA to produce several *Sdr4L* isoforms in the Sub I and Sub II clades in grasses. Divergence of these isoforms in monocots (*P*. *virgatum*, *S*. *bicolor*, and *Z*. *mays*) occurred ~42 to 64 MYA. This is in accordance with earlier studies suggesting that modern grasses emerged ~50 to 70 MYA [48]. We identified seven copies of *Sdr4L* genes in *P*. *virgatum*. The pairs *PvSdr4L1*-*L2*/*PvSdr4L3*, *PvSdr4L4*/*PvSdr4L5*, and *PvSdr4L6*/*PvSdr4L7* were located on chromosomes 2a/2b, 9b/9b and 9a/9a, respectively. The duplicated gene pairs *PvSdr4L1*-*L2*/*PvSdr4L3* probably resulted from whole-genome duplication (WGD) followed by chromosome translocation, while the remaining pairs possibly resulted from whole-genome duplication. These gene pairs may be due to a recent second genome-wide duplication event that may have occurred ~16 MYA. *Z*. *mays* and *S*. *bicolor* contained duplicated gene copies in Sub I and Sub II, in which they clustered together. This indicated a WGD event that occurred in a common ancestor [49, 50] followed by the divergence of these species ~12 MYA [51]. Ancestral eudicots undergone WGD, ~ 125 MYA [47]. According to this study, most of the Sdr4L genes were from core eudicots of rosids (Sub IV-VIII). When dating the WGD in eudicot of rosids, it is concentrated around 115MYA [52]. Ancestral eudicot undergone WGD, ~ 125 MYA [47], according to this study after the major WGD events, the *Sdr4L* genes in eudicots must have undergone diversification/speciation ~ 115 MYA. We identified a single copy of an *Sdr4L* gene in Arabidopsis, although it had undergone WGD [53]. This suggests that Arabidopsis may have lost some gene copies during speciation [18]. We noted several duplicated gene pairs in eudicot-specific family members. For example, soybean *GmSdr4L* genes in sub VIIIb (Fabidae, ~43 MYA) underwent segmental duplication about 6 MYA, corresponding to reports of two rounds of large-scale genome and/or segmental duplication of the soybean genome at about 14 and 42 MYA [54, 55]. In poplar, two gene copies (*PtSdr4L*) may have arisen from separate rounds of WGD [56]. Similar duplication events may have occurred in *L*. *usitatissimum* (*LuSdr4L*) and *G*. *raimondii* (*GrSdr4L*) [57].

### Functional divergence and positive selection in the *Sdr4L* gene family

Altered functional constraints may have resulted from duplication events between the gene clusters of a single gene family [58–61]. Based on the WGD, tandem and segmental duplication events observed among different *Sdr4*/*Sdr4L* subfamilies suggested that these paralogues may have a wide range of physiological functions. Evolutionary rate differences at some critical amino acid sites were investigated through functional divergence analysis (Table 1 and S3 Table) [20, 21]. Estimates of θI and θII (type I and II coefficients) were significantly greater than zero, suggesting that it is possible to attribute differences among *Sdr4L* genes to specific amino acid changes. These statistical results revealed that *Sdr4L* genes in some subfamilies (I/II, I/VIIIb, II/VI, II/VIIIb, II/IV, VI/VIIIb) should be functionally divergent from each other. Subfamily II (monocot) in particular seems to have diverged in function from IV, VI and VIIIb (dicots), indicating that monocot *Sdr4L* genes are likely to have functional differences compared to dicot counterparts. Rice *Sdr4* homologues were identified in Arabidopsis based on sequence similarity, but none correlation with a dormancy function [8]. In another study, dormancy-conferring paralogous of structurally distinct *AtDOG*-like genes in cereal plants showed dissimilarity in function to *AtDOG* [12]. This partially explained why these *Sdr4L* homologues in different plant species may have functionally diverged during evolution. Furthermore, compared with the number of CAAS for Type I functional divergence, higher numbers of Type II-related CAAS were identified for all subfamily pairs, strongly suggesting that the physiochemical properties of some ancient amino acids may have changed between subfamilies during functional divergence of Sdr4L proteins (Table 1). Significant differences in Type I and Type II functional divergence appear to reflect the effects of long-term selective pressures.

We also investigated the amino acid sites that have undergone strong positive selection. Site-specific profile analysis predicted that 17 candidate amino acid sites (Table 2) were under selection pressure, and 15 were implicated in functional divergence (S2 Fig), hence indicating that these sites were important in the evolutionary history of *Sdr4* genes. When branch-site model was used, no or few significant sites were found in *Sdr4* subfamilies except for subfamily II (S4 Table). Whereas subfamily II genes with 11 positive selection sites may have experienced higher selection pressure, other subfamilies seemed to be more conserved and no positive selection sites were identified. Moreover, subfamily II had a variable number of conserved motifs and introns compared to other *Sdr4* subfamily members. CAAS identified from branch sites implicated in functional divergence (Type I and II), but only two (74 P and 186 H) were implicated in positive site model selection. In total, 27 CAAS were responsible for both functional divergence and positive selection (S2 Fig). Conservation and variation of an amino acid residue site among duplicated genes has been reported [62]. These CAAS are assumed to be part of the coding region of the *Sdr4*/*Sdr4L* gene family and may play important roles in functional divergence of *Sdr4L* genes. We also predicted the potential phosphorylation sites in Sdr4L proteins. The results indicated that dicot Sdr4L proteins may have more phosphorylation sites than monocots (S2 File). A threonine (T) phosphorylation at site 73 was detected in the NLS motif of all dicot-specific Sdr4L proteins (Fig 3 and S2 File). According to Harreman et al. [63] phosphorylation sites regulate the transport of nuclear cargo proteins if they are within or adjacent to the NLS motifs of regulatory proteins. We found no phosphorylation sites in NLS regions of monocot Sdr4L proteins, which also possessed less phosphorylation sites overall than dicot proteins. These results thus indicated there might be functional divergence between monocot- and dicot-specific Sdr4L proteins.

We used i-TAASER [29] to build 3D structures of rice (OsSdr4) and tomato (SlSd4L) proteins through homology modeling, with labeled CAAS responsible for both positive selection and functional divergence analysis. Most amino acid sites were distributed on the loops and only a few sites (13 A, 16 I, 57 Q, 61 C, 63, 241) were on the sheets in OsSdr4L (Fig 4). Functional nucleotide polymorphism at amino acid residue position 174 in genes *Sdr4k* and *Sdr4n* showed differences in the levels of seed dormancy in rice cultivars Kasalath and Nipponbare, respectively [8]. This 174 (E) acid residue position was one of the 27 CAAS and was located on the 14th sheet of the rice 3D model (Fig 4), suggesting that positive selection in such regions might act as major drivers of functional divergence of *Sdr4L* genes, and thereby contributing to variation of function in different plant species. The general structure of all the Sdr4L proteins might have conserved fold since almost the same number of structural (sheets, loops and helices) regions were noted between the 3D models of OsSdr4L and SlSdr4L. Yet, SlSdr4L had one or two higher in number of both the sheets, loops and helices than OsSdr4L which might causes the possible structural divergences among Sdr4L proteins, in particularly between monocot and dicots. Structural divergences in Sdr4L can be explained by the presence of duplication events from evolutionary forces that noted only in monocots specific Sdr4L members (Fig 2). This duplication events could have produced variations such as insertions and deletions (Indels) of amino acid residues in the genomic content of Sdr4L. As shown in the Fig 3, more number of indels were observed between monocot and dicots specific Sdr4L members in throughout alignment. This is also could be a possible explanation for the structural divergences between Sdr4L members. There were six CAAS were found to be distributed on β sheets of both OsSdr4L and SlSdr4L. Moreover, the number of amino acid sites in domain I_1-240_ responsible for both positive selection and functional divergence was considerably greater than that in domain II_241-325_ (Fig 3 and S2 Fig). This difference might be associated with functional adaptiveness. Yet, unavailability of functional studies on *Sdr4* or *Sdr4L* genes, the present study just mapped some of the CAAS in the identified domain regions. These results suggesting that both structural divergence and CAAS those associated with positive selection and functional divergence may have acts a major evolutionary forces to create functional variation of Sdr4L proteins among plants. Therefore a future study on functional characterization of these Sdr4L proteins may required to understand their possible roles in different plant species.

### Expression and function analysis of *Sdr4L* genes

Differential expression patterns partly reflect gene function. The seed dormancy gene *OsSdr4* in rice acts as an intermediate regulator of dormancy during seed maturation [8]. In order to investigate how *Sdr4L* genes function in dicot and monocot species, we analyzed the expression profiles of 11 *Sdr4L* genes from different monocot and dicot species using available microarray and RNA-Seq atlas data. Previous studies showed that rice *OsSdr4* was specifically expressed in the embryo during seed development, suggesting functions in seed dormancy [8]. In our expression profiles, *Sdr4L* was abundantly expressed in seeds (rice, maize, soybean, barrel medic, and Arabidopsis) as reported by Sugimoto et al. [8], whereas other *Sdr4L* genes (tomato, cassava and poplar) were highly expressed level in both fruits and flower parts (S4 Fig). Rice *OsSdr4L* (LOC_Os07g39700) also showed preferential expression in inflorescence/flowers, as did tomato (*SlSdr4L*), cassava (*StSdr4L*), and poplar (*PtSdr4L1*). However, in poplar the *PtSdr4L2* (Potri.014G015300) was preferentially expressed in etiolated seedlings. Both copies of *Sdr4L* from soybean (*GmSdr4L1-2*) showed similar expression patterns and abundances during seed development suggesting that the functions of some *Sdr4L* genes might be relatively conserved.

Functional diversity among duplicated gene copies is obvious and could be caused by subfunctionalization, where deleterious mutations or changes in protein regulatory sequences are expected to occur in the promoter regions [64, 65]. We therefore examined potential *cis*-regulatory elements in the promoter regions of *Sdr4L* genes (S5 Table). All *Sdr4L* genes harbored variable numbers of *cis* elements including those related to hormonal, light and flowering responses. Functions of *OsSdr4* are related to abscisic acid (ABA) response along with the seed dormancy induction process [8]. Our search showed that almost all *Sdr4L* genes contain candidate sequences of ABREs (ABA-responsive elements). The calculated average numbers of *cis* element types in monocot *Sdr4L* members were slightly higher than in dicot *Sdr4L* members. We found no correlation between the presence or absence of *cis* elements and differential or similar expression characters of *Sdr4L* members from different plant species. These findings will require an additional exploration in the detailed differential expression characters and functions of *Sdr4L* members in different plant species.

So far, about thirty different types of seed dormancy related genes have been found in plants [5]. Most of them from Arabidopsis and only a few genes such as *Sdr4*, *VP8*, and *MFT* were reported from monocots such as rice, wheat, and maize [5]. Sugimoto et al. [8] suggested that *Sdr4* in rice has a regulatory role in dormancy induction and germination inhibition, but there was no indication of how *Sdr4* regulates these processes. Similar to this, wheat *TaSdr4* gene was also found to be associated with seed dormancy and PHS tolerance in different wheat cultivars [9]. In Arabidopsis, a transcription factor regulator called *ABI3* have been reported to implicates in seed development and maturation. These *ABI3* was found to regulates more number of genes as their targets in which AtSdr4L (AT1G27461) was also identified as one of a target [66]. This suggests that AtSdr4L might have indirectly involved in the process of seed dormancy in Arabidopsis. However in an another study, *AtSdr4L* gene (AT1G27461) was reported as nuclear encoding protein called drought responsive gene (*DRG*) which confers drought and freezing stress tolerance in *drg* mutants of Arabidopsis [67]. Altogether demonstrates that Sdr4 genes in monocots such as rice and wheat might have conserved functions, but it could have functionally diverged during the evolution process in dicots such as Arabidopsis. We predicted the structures of Sdr4L proteins in rice and tomato using i-TASSER [29] based on the homology model as shown in Fig 4. The 3D structure of rice OsSdr4 protein indicated similarity to methylamine dehydrogenase (Protein Data Bank [PDB] code: 2gc4E). The predicted gene ontology terms (GO:0005515) indicated that this protein might participate in methylamine metabolism (S3 File). Thus Sdr4 proteins may have a role in chromatin remodeling through histone methylation [68] with positive effects on transcriptional elongation of seed dormancy-related genes such as *DOG1*, *NCED9*, and *ABI4* [6]. We identified dormancy-conferring *Sdr4L* genes from dicot and monocot species. In order to determine the complete functions of these *Sdr4L* genes in plants, we aim to develop of transgenic lines containing different *Sdr4L* genes and to understand their effects on seed dormancy. Any positive effects should be useful for improving PHS tolerance in cereals.

## Conclusions

We identified 54 *Sdr4L* genes in 36 plant species representing land plant lineages of monocotyledonous and dicotyledonous angiosperms. Phylogenetic analysis revealed that genes from same lineages tended to cluster together and were classified into eight well-conserved subfamilies supported by exon/intron pattern and motif analyses. *Sdr4L* genes in various plant species and subfamilies showed different evolutionary expansion patterns. Segmental duplication was the probable expansion pattern of *Sdr4L* genes in some dicot-specific subfamilies. Tandem and segmental duplication events played important roles in expansion of *Sdr4L* genes in monocot-specific subfamilies grass I and grass II, the latter subfamily possessed structurally distinct *Sdr4L* genes. *Sdr4*/*Sdr4L* genes in ancestral angiosperms underwent major divergence ~160 MYA to generate subfamilies in the land plants. Functional divergence analyses showed that changes in functional constraints occurred in all pair comparisons, but were more significant in subfamily pairs of I/II, I/VIIIb, II/VI, II/VIIIb, II/IV, and VI/VIIIb, indicating the changed evolutionary rates. Additional analyses revealed that *Sdr4L* genes were under positive selection during evolution. The sites of functional divergence and positive selection identified by CAAS were mainly distributed in protein loop regions, indicating the importance of these regions in driving the functional divergence of Sdr4L proteins. Finally, differential expression profiles showed functional diversity among duplicated copies of *Sdr4L* in various species. These data may provide solid foundation for further functional dissection of *Sdr4L* genes in plants.

## Supporting Information

S1 Fig Motifs in Sdr4L proteins from different plant species identified by MEME analysis. (DOCX)

S2 Fig Relationships between Type I and Type II amino acid sites related to functional divergence, and positively selected sites. All sites are positioned on the reference sequence (OsSdr4) based on multiple sequence alignment.
(TIF)

S3 Fig Multiple sequence alignment of *Sdr4L* genes in monocot and dicot plants. The amino acid sequences of plant Sdr4L proteins were aligned using CLUSTAL X (1.81). In the alignment, the residues are displayed in the “Difference Mode” with the “Diff/Consensus Line” style. Dots indicate conserved residues with the first protein OsSdr4, and “-” indicates gaps on the alignment. Black arrowheads indicated the predicted protein domains (PD319905 and PDB0A0W9). Critical amino acid sites responsible for functional divergence are shaded in red. Residues identified from the tests of positive selection are shown with green arrows. Motifs (1–6) identified from MEME analysis are represented in parentheses in various colors.
(RTF)

S4 Fig Differential expression profiles of *Sdr4L* genes. In-silico expression patterns for rice (A), maize (B), soybean (C), barrel medic (D), Arabidopsis (E), tomato (F), cassava (G), and poplar (H) *Sdr4L* genes based on microarray data and the RNA-Seq atlas. Fold changes in expression level are indicated by the intensity of red (for up-regulation) or green (for down-regulation) Color. The color scale shows variation in gene expression.
(PDF)

S1 File Neighbor-joining (NJ) phylogenetic tree of the *Sdr4* gene family. (MTS)

S2 File Predicted average numbers of phosphorylation sites in *Sdr4L* subgroup proteins obtained from phylogenetic analysis. (DOCX)

S3 File Three dimensional protein structure prediction data using i-TASSER server. (PDF)

S1 Table Predicted *Sdr4* and *Sdr4L* gene models and related information. (DOC)

S2 Table Number of identified protein domains in Sdr4L proteins produced by ProDom search. (XLSX)

S3 Table Amino acid sites of functional divergence between subfamilies of the Sdr4L proteins. (DOCX)

S4 Table Parameter estimation and likelihood ratio tests for branch-site models. (DOCX)

S5 Table Functions and numbers of identified *cis* elements in the 5’ flanking region of *Sdr4L* genes from different plants species. (DOCX)
