# Supplementary material for: Phylogenetic Analysis, Lineage-Specific Expansion and Functional Divergence of seed dormancy 4-Like Genes in Plants
Source: PLoS One. 2016 Jun 14;11(6):e0153717. doi: 10.1371/journal.pone.0153717 (PMC4907471; doi:10.1371/journal.pone.0153717)

**S1 Fig.** Motifs found in Sdr4L proteins from different plant species by MEME analysis.

**
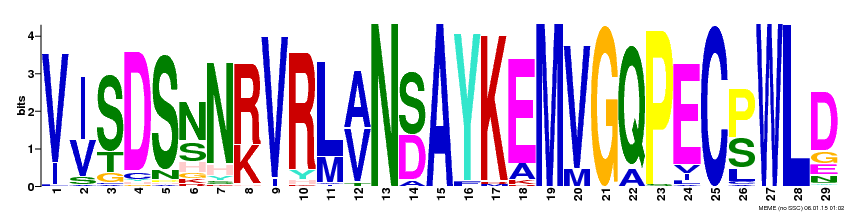
Motif-1**

**Motif-2
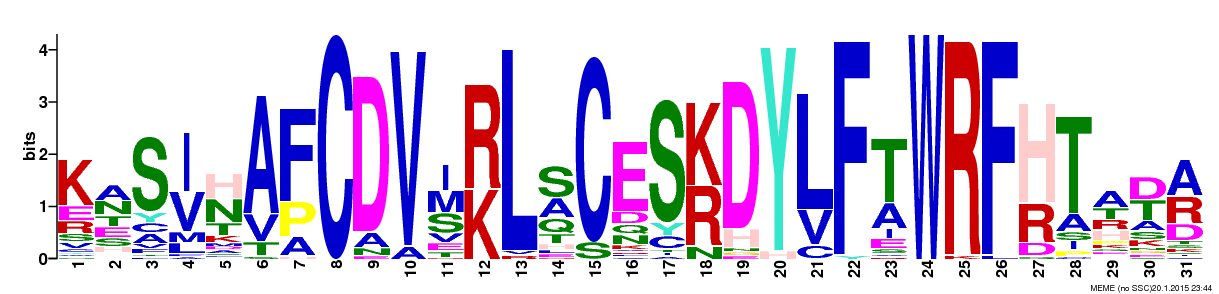
**

**Motif-3**

**
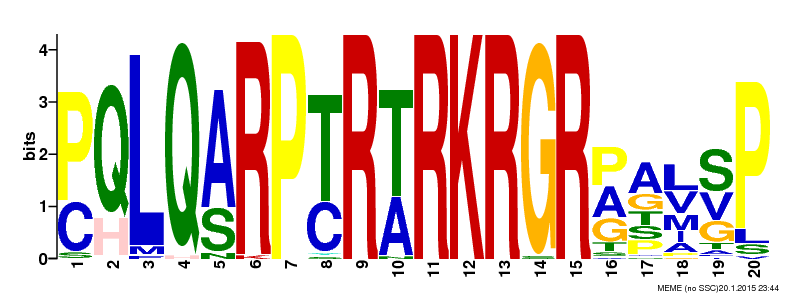
**

**Motif-4**

**
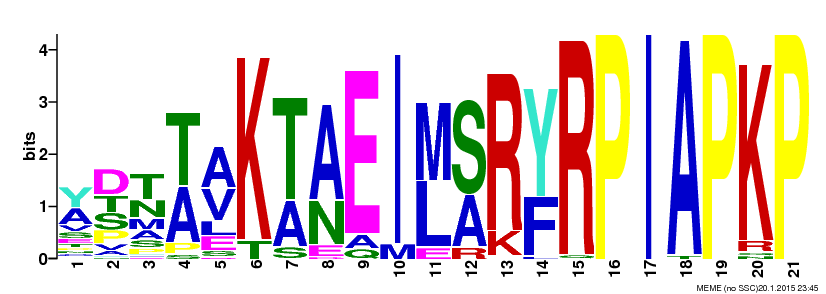
**

**Motif-5**
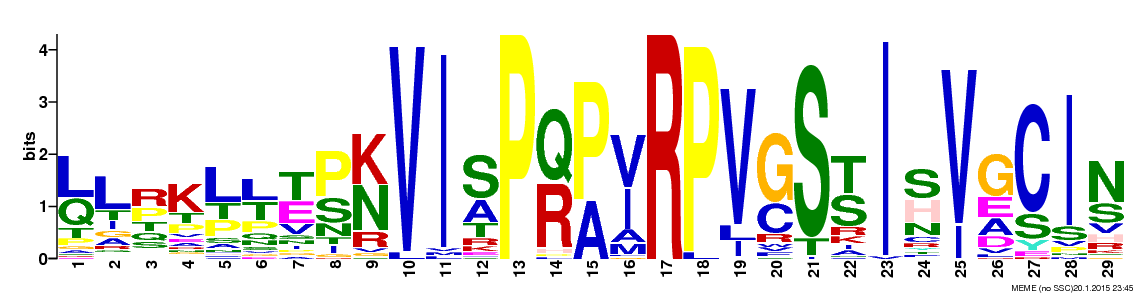


**Motif-6**


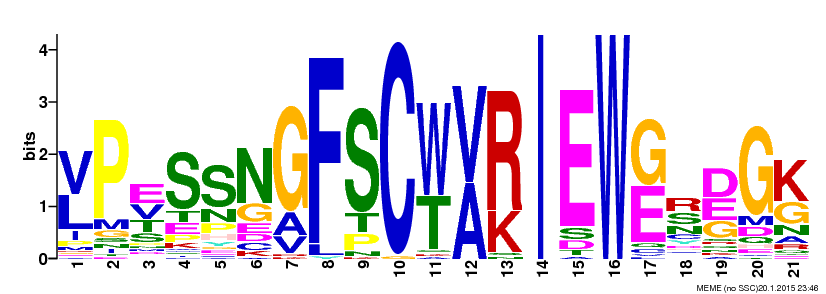

Supplement: S1 Fig — (DOCX) [file pone.0153717.s001.docx]
