# Supplementary figures and images for: Phylogenetic Analysis, Lineage-Specific Expansion and Functional Divergence of seed dormancy 4-Like Genes in Plants
Source: PLoS One. 2016 Jun 14;11(6):e0153717. doi: 10.1371/journal.pone.0153717 (PMC4907471; doi:10.1371/journal.pone.0153717)

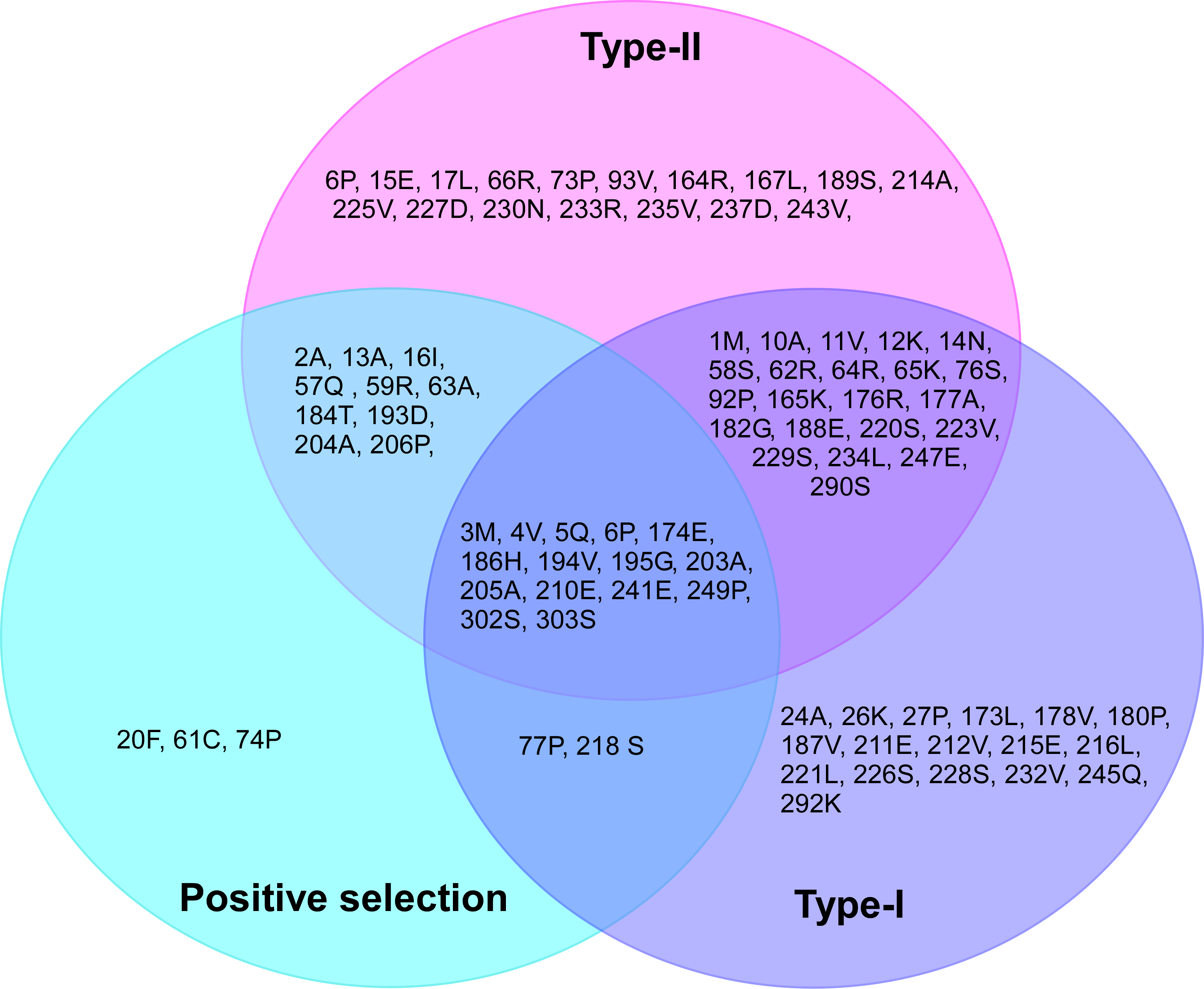

Supplement: S2 Fig — All sites are positioned on the reference sequence (OsSdr4) based on multiple sequence alignment. (TIF) [file pone.0153717.s002.tif]

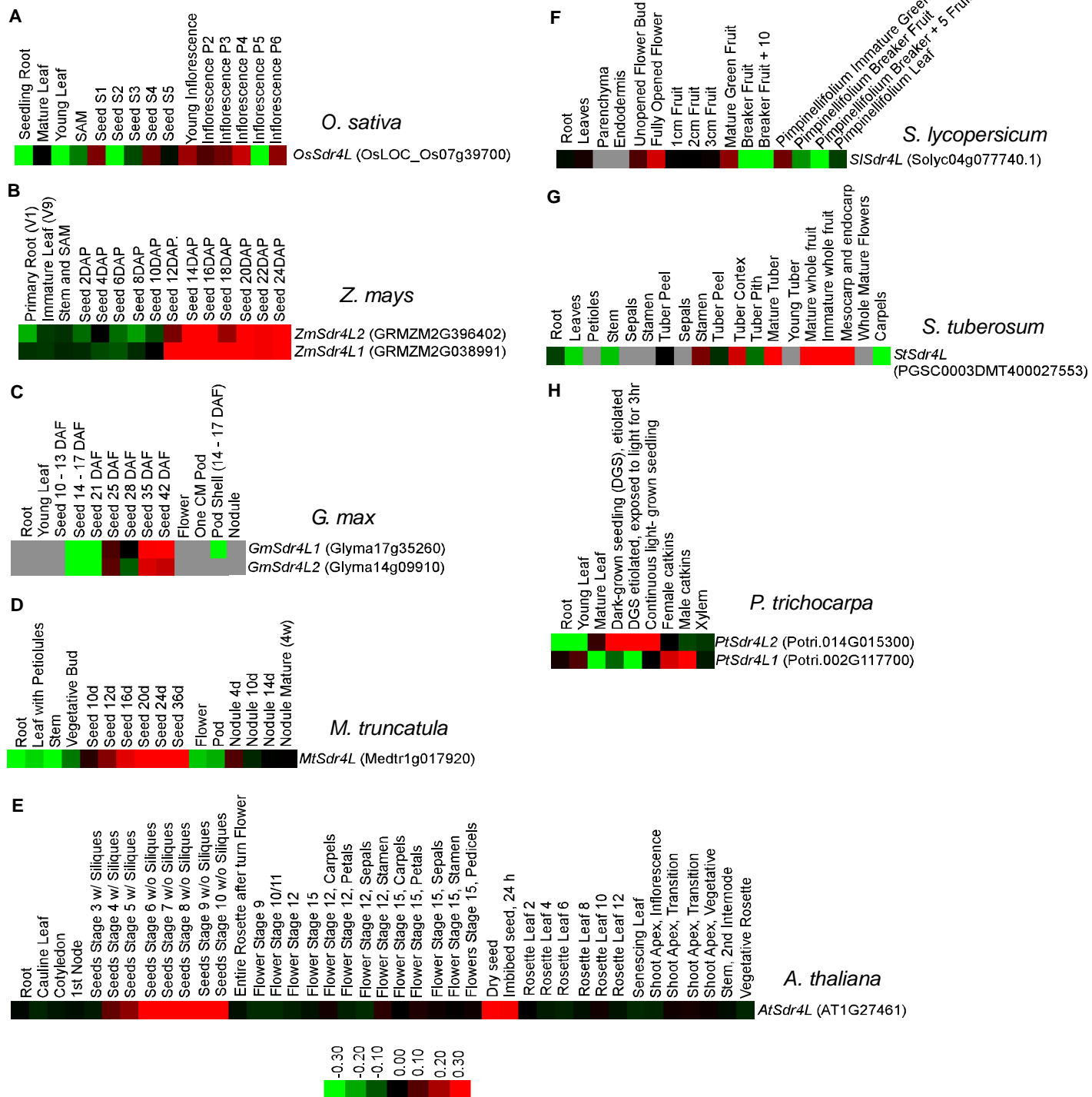

Supplement: S4 Fig — In-silico expression patterns for rice (A), maize (B), soybean (C), barrel medic (D), Arabidopsis (E), tomato (F), cassava (G), and poplar (H) Sdr4L genes based on microarray data and the RNA-Seq atlas. Fold changes in expression level are indicated by the intensity of red (for up-regulation) or green (for down-regulation) Color. The color scale shows variation in gene expression. (PDF) [file pone.0153717.s004.pdf]
