# Supplementary material for: Phylogenetic Analysis, Lineage-Specific Expansion and Functional Divergence of seed dormancy 4-Like Genes in Plants
Source: PLoS One. 2016 Jun 14;11(6):e0153717. doi: 10.1371/journal.pone.0153717 (PMC4907471; doi:10.1371/journal.pone.0153717)
Supplement: S3 Fig — The amino acid sequences of plant Sdr4L proteins were aligned using CLUSTAL X (1.81). In the alignment, the residues are displayed in the “Difference Mode” with the “Diff/Consensus Line” style. Dots indicate conserved residues with the first protein OsSdr4, and “-” indicates gaps on the alignment. Black arrowheads indicated the predicted protein domains (PD319905 and PDB0A0W9). Critical amino acid sites responsible for functional divergence are shaded in red. Residues identified from the tests of positive selection are shown with green arrows. Motifs (1–6) identified from MEME analysis are represented in parentheses in various colors. (RTF) [file pone.0153717.s003.rtf]

S3 Fig. Multiple sequence alignment of Sdr4L proteins in monocot and dicot plants.
 10	20	30	40	50	60	70	80	90	100	110	120	130	140	150	160
....|....|....|....|....|....|....|....|....|....|....|....|....|....|....|....|....|....|....|....|....|....|....|....|....|....|....|....|....|....|....|....|       |           |             |    |     |
OsSdr4	-------------------------------------------------------------------------------------------------------------------------------MAMVQPVD---------MAVKANEIMARFRPIA
PvSdr4L1	-------------------------------------------------------------------------------------------------------------------------------......A.---------P.......L.......
PvSdr4L2	MANVTGSRSGQATRMQTPRLPPSPHSILAASSHWNPPPPNPSAP-------SKARAPATRLR---------------------LRVGRVPARSPTWPP--------------------------LFA...A..A.---------P.......L.......
PvSdr4L3	MQRKTIKEIARRAAILANETPSGAHATTPSMARAPASQPVPAARPAERSTGDEARAPARRLLPLEPTQPKR-------APPPRLGSAYVSAASPRGRPRSPFSLEWSSPLTTDLDPEPREPAAALLA......A.---------P.......L.......
PvSdr4L4	-------------------------------------------------------------------------------------------------------------------------------..HQKE.A---------AETSEAMER-.....T
PvSdr4L5	----------------------------------------------------------------------------------------------------------------------------MAFR.HQKE.A---------AETSEAMER-......
PvSdr4L6	-------------------------------------------------------------------------------------------------------------------------------..HEKE.A---------AETSEAMER-..Q...
PvSdr4L7	----------------------------------------------------------------------------------------------------------------------------MAF..HEKE.A---------AETSEAMER-......
SiSdr4L	MARTPASQCPRPSRRNAPRGTRRPRQRGTAKARGKPKRP-SDPHAGPRRHLTRPDALLSSVLPLEPTQPKPPLQSARPATRLRLRVGRVPARSPTVSLEWSSTLTTDP-----------DPEPPLLA......A.---------P.....Q.L.......
SbSdr4L1	-------------------------------------------------------------------------------------------------------------------------------......A.---------T.......L.......
SbSdr4L2	-----------------------------------------------------------------------------------------------------------------------MERRLRAIAPKPL.PP---------APPRSLLWRGHK.GRD
BdSdr4L1	-------------------------------------------------------------------------------------------------------------------------------......A.---------V.......L..L....
BdSdr4L2	-------------------------------------------------------------------------------------------------------------------------------..L...A.---------........L.......
ZmSdr4L3	-------------------------------------------------------------------------------------------------------------------------------.....AA.---------A.......L.......
ZmSdr4L2	-------------------------------------------------------------------------------------------------------------------------------......A.---------T.......L.......
ZmSdr4L1	-------------------------------------------------------------------------------------------------------------------------------.PPLM.TG---------A.ATSVLWR..K.GRD
TaSdr41b	-------------------------------------------------------------------------------------------------------------------------------........---------........L.......
TaSDr41d	-------------------------------------------------------------------------------------------------------------------------------......A.---------........L.......
TaSdr41a	-------------------------------------------------------------------------------------------------------------------------------........---------........L.......
AetSdrL	-------------------------------------------------------------------------------------------------------------------------------........---------........L.......
HvSdr4L	-------------------------------------------------------------------------------------------------------------------------------........---------......Q.L.......
AcSdr4L	------------------------------------------------------------------------------------------------------------------------------M.NSMNVYSS------SSTSS.TDQ.LS.Y....
StSdr4L	------------------------------------------------------------------------------------------------------------------------------M.QTLN.YP---------STS.TA.....Y....
SlSdr4L	------------------------------------------------------------------------------------------------------------------------------M.QTLN.YP---------STS.TA.....Y....
VvSdr4L	------------------------------------------------------------------------------------------------------------------------------MIRTLN.YN---------STA.TA...S.Y....
EgSdr4L	------------------------------------------------------------------------------------------------------------------------------MIKTLS.YSTTP-----AATA.TA...S.Y....
PtSdr4L2	------------------------------------------------------------------------------------------------------------------------------MIKTLS.CS---------NTA.TA...S.Y....
PtSdr4L1	------------------------------------------------------------------------------------------------------------------------------MIKTLS.CS---------NTA.TA...S.Y....
LuSdr4L1	------------------------------------------------------------------------------------------------------------------------------MIKTLT.Y---------SNTA.TA...S.Y....
LuSdr4L2	------------------------------------------------------------------------------------------------------------------------------MIKTLT.Y---------SNTA.TA...S.Y....
MeSdr4L	------------------------------------------------------------------------------------------------------------------------------MIKTLN.YS----------TA.TA...S.Y....
RcSdr4L	------------------------------------------------------------------------------------------------------------------------------MIKTLS.YS---------TTA.TA...S.Y....
CpSdr4L	------------------------------------------------------------------------------------------------------------------------------MIKTLS.YS---------TTA.TA...S.Y....
GrSdr4L1	------------------------------------------------------------------------------------------------------------------------------MIKTLS.YS---------TTATTA...S.Y....
GrSdr4L2	------------------------------------------------------------------------------------------------------------------------------MIKTLN.Y-----------SA.TA...S.Y....
TcSdr4L	------------------------------------------------------------------------------------------------------------------------------MIKTLN.YS---------TTA.TA...S.Y....
AtSdr4L	------------------------------------------------------------------------------------------------------------------------------MIKILN.HS-HH---SQTTTL.TA..LSKY....
BsSdr4L1	------------------------------------------------------------------------------------------------------------------------------MIKILN.HSHHS---HSTTTL.TA..LSKY....
BrSdr4L1	------------------------------------------------------------------------------------------------------------------------------MIKILS.HNSHS---TTTTTL.TA..LSKY....
BrSdr4L2	------------------------------------------------------------------------------------------------------------------------------MIKILSSHNSHHSHSTTTTTL.TA..LSKY....
CgSdr4L1	------------------------------------------------------------------------------------------------------------------------------MIKILN.--------HSTTTL.TA..LSKY....
CrSdr4L1	------------------------------------------------------------------------------------------------------------------------------MIKILN.--------HSTTTL.TA..LSKY....
EsSdr4L	------------------------------------------------------------------------------------------------------------------------------MIKILN.HQSHHSH-STTTTL.TA..LSKY....
CcSdr4L	------------------------------------------------------------------------------------------------------------------------------MIRTMS.FSA-------ATTA.TA...S.Y....
CsSdr4L	------------------------------------------------------------------------------------------------------------------------------MIRTMS.FSA-------ATTA.TA...S.Y....
CSaSdr4L	------------------------------------------------------------------------------------------------------------------------------MIKTLP.PFP--------STD.TA...S.Y....
FvSdr4L	------------------------------------------------------------------------------------------------------------------------------MIKTLN.YNS-------TNTA.TA...S.Y....
GmSdr4L1	------------------------------------------------------------------------------------------------------------------------------MIKTLN.YP---------NPA.TA...S.Y....
GmSdr4L2	------------------------------------------------------------------------------------------------------------------------------MIKTLN.YP---------NPA.TA...S.Y....
MdSdr4L1	------------------------------------------------------------------------------------------------------------------------------MIKTLN.Y---------TNTE.TA...S.Y....
MdSdr4L1	------------------------------------------------------MLPFSDWSSLTLKNSNPSLNPPPHQPPXKSLPPSXTCLPALSLLPPYRRRHHRHYRTAGRPALAKRTAYRHNMIKTLN.Y---------TD.E.TA...SXY....
MtSdr4L	------------------------------------------------------------------------------------------------------------------------------MIKTLN.YP---------NPA.TA...S.Y....
PhvSdr4L	------------------------------------------------------------------------------------------------------------------------------MIKTLN.YP---------NPA.TA...S.Y....
PpSdr4L	------------------------------------------------------------------------------------------------------------------------------MIKTLN.YN--------SNTE.TA...S.Y....
	                                                                                                                                                            :   
170	180	190	200	210	220	230	240	250	260	270          280	290	300	310	320
....|....|....|....|....|....|....|....|....|....|....|....|....|....|....|....|....|....|....|....|....|....|....|....|....|....|....|....|....|....|....|....|       |            |           |            |           
OsSdr4L	PKPVLPAAAAG------------------------VTGGGDGAAAVAATNRVLCQLQSRPCRARKRGRPS--VVPPVSP-------PAGAKRKR-------------------APAYPVPVAPLRCAAV-AT------------ATRARVSVVVVPAPES
PvSdr4L1	...A.A...S----P-------------------VAQAAAE.VV---.A....................--T...VSPK---SPA-QPA.....--------------------A.A.Y.PLRCAA.TDAVA------------TATRAHVS....GSAC
PvSdr4L2	...A.A...S----P-------------------VTQAAAE.VV---.A....................--T...VSPK---SPA-QPA.....--------------------A.A.Y.PLRCAA.TDVVA------------TAARAHVS....DSAC
PvSdr4L3	...A.A....S---P-------------------VAQAAAE.VV---.A....................--N...VSPK---SPA-QPA.N...--------------------A.A.Y.PLRCAATTDAVA------------TATRAHVS....GSAC
PvSdr4L4	...LPESPPPMPIGTT----------------------------------------ATNDGEVPVVMGAWCMPESFLPG-----------------------------------CEEHLQGLS.EGSSISLWA----------------------SSQDA
PvSdr4L5	...LPASPPSMPIGTTWL-----------------------------SYPPPPIRWATNDGEVPVVT.AWCMPESFLPG-----------------------------------CEEHLRGLS.EGSLISPWA----------------------.S.DT
PvSdr4L6	...LPAPPPPMPISTTVSSILLGAHKRNRQDYLVPSPVSKRERD.LSYPPPPPVRWATNDGEVPVVM.AWCMPESFLPS-----------------------------------CEEHFQGLS.EG----------------------------------
PvSdr4L7	...LPAPPPPMPISTTVSSILLGAHKRNRQDYLVPSPVSKRERD.LSYPPPPPVRWATNDGEVPVVM.AWCMPESFLPS-----------------------------------CEEHFQGLS.EGSS.SWRA----------------------.S.DA
SiSdr4L	...A.A..SP------------------------VAQAAAE.VV---.A........N...........--T...VSPK---SPA-QPA.....--------------------AEA.Y.PLRCAA.T-----------------ATRAHVS....DSAC
SbSdr4L1	...T.AT...AAS-P-------------------VAQAAAE.VV---.A.....H..............--T...VSP.KSGSGA-QSP.....--------------------A.T.Y.PLRCAA.TA-------------------CSASA...VSAR
SbSdr4L2	-DHL.LSPPVSKREREATS-------------------SSSSSSSYPYPYPYPPPPLLPAAGVGLG-.YMSMPEGVLAG-----------------------------------CEERLRGLS.VAGS---------------------------..AAA
BdSdr4L1	...PAALMTTS------------------------PAQTIG..----.AS...S...............-----.VSPL-------A.RR..PA--------------------AP..A.QLRCAA.TDG.V------------VST.TRAR.S.DG---
BdSdr4L2	...---PS.MP------------------------TQQPIG..G--.G.S...SH..A..Y.........--.A...P.P---PH-A.A.R...TA-----------------VVP..A.P-LGCA.PTD.V------------VST.MRA-----GSAC
ZmSdr4L3	.N.T.A---------------------------------AE.VV---.A.....H...K..........GPA..A.SPE---SGL-QPP.....--------------------ATT.Y.PLRC.GPRA----------------------SAA..GSAG
ZmSdr4L2	...T.A....AAAAP-------------------VAQAAAE.VV---.A.....H..............--T...VSPK---SGA-QPP...R.--------------------AST.Y.PLRCAA.TTG------------------AHVSA...GSAR
ZmSdr4L1	EDRL.LSPPLSKRETEREA-------------------A.ASS----YP.PQPPASAAPVAAT.RGR.YVPMPEGLLTG-----------------------------------CEERLRRLS.VAGSSP.AAL-------------------PW..AAA
TaSdr41b	...-ALP.SPA------------------------QAQAI..-----.AD....H...............--A..VSA.-------A.A.....--------------------A.....LRCAAA..TD.V------------VST.TRAY.S..GSAC
TaSdr41d	...-ALP.SP--------------------------VQAI..-----.AD....H..N............--A..VSA.-------A.A.....--------------------A.....LRCAAA..TD.V------------VST.TRAY.S..GSAC
TaSdr41a	...-ALP.SPA------------------------QAQAI..-----.AD....H...............--A..VSA.-------A.A.....--------------------A.....LRCAAA..TD.V------------VST.TRAY.S..GSAC
AetSdrL	...-ALP.SPA------------------------QAQAI..-----.AD....H...............--A..VSA.-------A.A.....--------------------A.....LRCAAA..TD.V------------VST.TRAY.S..GSAC
HvSdr4L	...AALP.SP--------------------------AQAI..-----.AS....H...............--A..VSAQ-------..A.....--------------------A.....LRCAAA..TD.V------------VST.TRAH.S..GSAC
AcSdr4L	....VEGPPTPENTQ-----------------------------QSPFSK----Y.LT..S.N....K-----SGCLPS-----------..A.TT--QSAAFSTPCG--VASLAKNTQLGLS.QGYGHGFP-----------SQLPIPSFDLSSSLEKP
StSdr4L	...EA.TSPVSEDNP---------------------..LPPNIQKSPFLRN.WP...A..T.T.....-----TALGP.S---------M..A.ANYFPAGQFPTYQQ------VMAAS.SYRPNVLPQFT----------LIPNLLPLKCGLGTSVTTP
SlSdr4L	...EA.TSPVSEDNP---------------------..LPPNIQKSPFLRN.WP...A..T.T.....-----TALGP.S---------M..A.GNYFPAGQFPNYHQ------VVAAS.SYRPNVVPQFT----------LIPNLLPLKCGLGTSVTTP
VvSdr4L	...EP.VNPIPG-------------------------------FSP..LK.PRTH.VGFSTPSHVTS------PAKNLSM-------H.FTH---------------------------SPLQ.P-------------VPAPSF.PLNGGFERAISTTAD
EgSdr4L	...ETVPS..SSAAESP-------------------.SMSPKIRQSPYLRNLWP...A..T.T.....-----TAISP.A---------I..PKTAATAAGAPAGVPSP----CALP.TKSLS.QVFTHGLP---HIPLAGLMESPPVALAGSAAA.SSP
PtSdr4L2	...EG----STDESP----------------------SMP------PFLRTLWP.MHA..T.T.....-----AAVSPLT---------I..P.T---HLLGLSSPSH------AT.SAKHLS.QGF.HGI.-------QLPVPNLVGINCGMENSVTV.
PtSdr4L1	.R.EGS-.SSMDESS----------------------SMSQKIRESPYLRTLWP.M.A..T.T.....-----AVVSP.N---------I..P.T---HLLGLSSPSH------VTS.AKHLS.QGFVHGIP-------QLPVPNLVGVN.GLENSVTM.
LuSdr4L1	...DC.-SNSEDESS----------------------SMSQKIIQSPYLRNLWP...A..T.T.....-----GALSP.---------VV..P.TH--QMFSISPPAE------HLG-MQGFYPTGFSQLP.-------VQN-PGAIQSN..IAS----P
LuSdr4L2	...DC.-SNSEDENS----------------------SMSQKIRQSPYLRNLWP...A..T.T.....-----GALSP.---------VV..L.TH--QMFSISPPAE------HLG-MQGFYPTGFSQLP.-------VQN-PGAIQSN..IAS----P
MeSdr4L	...E..-SNSLGDTP----------------------SMSQKIRQSPYLRNLWP...A..T.T.....-----ATMSP.T---------I..P.T---HLLGFSSPNH------VLS.ARHLS.QGFVHGVS-------QLPISNLAGVS.RSEN.VTAA
RcSdr4L	...EG.-SNSFGESS----------------------SMSQKISQSPYLRNLWP...A..T.T.....-----AAISP.T---------I..P.T---HVLGLSSTSH------VIS.ARHLS.QGF.HGLS-------QLSVPSLVGVS.SLNN.VTTN
CpSdr4L	...EV.-MDPISDGAGS--------------------SMSQKIRQSPYLRNLWP...A..T.T.....-----A.LSP.S---------L..A.TH--HVLGLSVPSH------VTS.AKALSSQAFTH-----------LPAPNLAPVTCNLEN..TQ.
GrSdr4L1	...DV.-.NSMNESS----------------------.MSQKMMQSPYLRSLW.H..A..T.......-----.ALS.TTP--------L..G.T---QVLALSSPSL------ITS.AKNLS.QGFSHGIP-------HHSIPNFG---GSLDSSSTPP
GrSdr4L2	...EVLPENSIDESS----------------------AMSQKMRQSPYLRNLWP...A..S.N.....G----TGLSP.PP----TTTAM..A.TQ--YFLGLSPPPP------.PP.P.SS-------------------------------------T
TcSdr4L	...EV.-.NSLNENS----------------------AMSQKIRQSPYLRNLWP...A..T.T.....-----AALSP.T---------L..A.T---HVLGLSSPSP------VTS.AKNLS.QGFSHGIP-------QLSVPNFVNTGGGLEISCAPP
AtSdr4L	...GT.RVNDDDPS----------------------SSMSHKISQSPYLRNLWP...A..T.T.....-----GGMGPSS-------LAM..PKSSCGSS--STS-TISTQR--VLG.IKTLSFQAFTHHR----------LPNLP-QVGYGFEN--GV.
BsSdr4L1	.R.GT.RVNDDDPS----------------------SSMSHKITQSPYLRNLWP...A..T.T.....-----GGMGPSS-------LAM..PKSSCASSPTSSTTTTTTQR--VIG.IKTLSFQGFTHHG----------LPNL.-QAGYALEN--GG.
BrSdr4L1	...GTTP-QVNDNDS-------------------SSSSMSHKISQSPYLRNLWP...A..T.T.....-----GGMGPTSP------LSL..HKPSSS----ASTTTTTPQR--VFG.IKTLSFQAFSHAG----------IPNLA-QVGYALEN--GG.
BrSdr4L2	.R.GTTQVNDNDSSS-------------------SSSYMSHKISQSPYLRHLWP...A..T.T.....-----GGMGPTSH------LSL..PKSLAT------STKTPTQR--VFG.IKTLAFQAFSHAG----------LPNL.TQVGYALEN--GG.
CgSdr4L1	...GT.RLNDDDPS----------------------SSMSHKISQSPYLRNLWP...A..T.T.....-----GGMGPSS-------LAM..PKSSCVSSPTSSS-TTTTQR--VIG.IKTLSFQAFTHHG----------LPSL.-QVGYALEN--GA.
CrSdr4L1	...GT.RVNDDDPS----------------------SSMSHKISQSPYLRNLWP...A..T.T.....-----GGMGPSS-------LAM..PKSSCVSSPTSSS-TTTTQR--VIG.IKTLSFQAFTHHG----------LPSL.-QVGYALEN--GA.
EsSdr4L	...GTTSSQVNDND--------------------TSSSMSHKISQSPYLRNLWP...A..T.T.....-----GGMGPTSP------LAL..PKSSSPSATISSTTTTTTPR--VFG.IKTLSFQAFPHG-----------LPSLA-QVGYTLENNGGG.
CcSdr4L	...EA.VSPMSESAKIRQSPYLRNLWPQLQARPTRTRKR.RA.ISPPTIK.SRTH.FGLSSPCHATSS-----PAKNPSL-------Q.FAHPHAH---------------------G.AQFT.PNH----------LATVPASCSLDNPASTATTITTN
CsSdr4L	...EA.VSPMSESAKIRQSPYLRNLWPQLQARPTRTRKR.RA.ISPPTIK.SRTH.FGLSSPCHATSS-----PAKNPSL-------Q.FAHPHAH---------------------G.AQFT.PNH----------LATVPASCSLDNPASTATTITTN
CSaSdr4L	...ES.FPTSDHSLN-------------------NIPHSSSSSSSSSFLRN.WP...A..T.T...S.-----P..-ISP-------HSL..T.IT---------------------.S.NFS.HHHPFSSS-----LLPHLSLPSINSGFRDS-SSNSN
FvSdr4L	...ETSPTTPTGESP----------------------ALSQKIRDSPYLRNLWP...A..T.T.....-----AAISP.---------TL..Q.TS--HVFGLSPACH------VTS..NNLT.DGFPHPL.-------QLALPNQLGSA.SGLE----.
GmSdr4L1	...ETS-PNS-MSEGP------------------SSSSLSQKIKQSPYLRNLWP...A..T.T.....-----A.LTL.S-------SSL..HKTTHHHVLGFCPPCHHVVTSSSSS.SKNLS.QG-FAPP-----HPLPHH-LGVLNCTMEKNNT---N
GmSdr4L2	...ETS-PNSSMSEG-------------------SCSSLSQKINQSPYLRNLWP...A..T.T.....-----A.LTL.S-------SSL..HKTT--HILGFCPPCH-VVTSS---.AKNLSFQG-FAP--------LPNHGLGVLNCTMENNNTLTAN
MdSdr4L1	....T.-SNPAGESP----------------------SMSQKIRDSPYLRNLWP...A..T.T.....-----SALSPT---------TF..Q.SH--QMLGFATPCH------VQS..KNPR.DGF.RVLP-------KLPIPXSLDAANN-------.
MdSdr4L2	.Q.EIT-.NPAGXXP----------------------SMSQKIRDSSYLRNLWPXF.A..T.T.....-----TALSPI---------TF..Q.SH--HMLGFSTXCH------VQS..KNLR.DGF.RAL------------YGSFDTAN--------T
MtSdr4L	...ETCSSNNSTSDGSS-----------------SSNSLSQKIKQSPYLRNLWP...A..T.T.....-----A.ISL.--------SSL..QKTH---VLGFCQPLH--VTSP----IKNLT.QGNF.PPSSLPQLPLPNHGVGVLNCN--KNSTT--N
PhvSdr4L	...ETS-PNS-MTEG--------------------SSSLSHKIKQSPYLRNLWP...A..T.T.....-----A.LTL.P-------SSF..HKTH---ILGFCPPCH--VTSP----GNNLSFQG-FAPPP--LPLPHPNHGLGMLN.SIEKNSLM--N
PpSdr4L	...ET.-.NSAGENP----------------------SLSQKIRESPYLRNLWP...A..T.T.....-----SALSPT---------TF..Q.TH--HVFGFSTPCH------VTS.AKNLT.DGF.HALS-------QLPIPTSFDA.K--------.
.
 330	340	350	360	370	380	390	400	410	420	430	440	450	460	470	480
....|....|....|....|....|....|....|....|....|....|....|....|....|....|....|....|....|.... |....|....|....|....|.... |....|....|....|....|....|....|....|....|....|
OsSdr4	AGGVS--ALAPVSPS---------------AGDSTRLSPTVVEVE---------DEDEERGVVLVERDLLRKLLEPR--------------KLLEPRAVRPVGSTIHVESVHIDVGR--------TTAAAAAAAPKTAEEVEAELES-DSLPAVVSDSS-
PvSdr4L1	LPLA.--LPPAATV----------------.E.LVKVAAE-------------------ERD.P............---------------.VIS.......C......C--.RRT---------GATCT-D.G....A.......A-.A........G-
PvSdr4L2	LPLA.--LPPAATV----------------.E.LVKVAAE-------------------ERD.P............---------------.VVS.P.G...C......C--.RRT---------DATCT-D.I....A.......A-.A........G-
PvSdr4L3	LPLA.--LPPADTV----------------.E.LVKVAAE-------------------ERD.P............---------------.VIS.......F......C--.RRT---------DATCT-D.VS...A.......A-.A........G-
PvSdr4L4	G-----------------------------------------------------------RLFP.....IS..QV.---------------.VIR..PA...RII.CIDCSN.V.DATT-------SVLEV.MSN..PR...V...LP.A...I.AGYNN
PvSdr4L5	G-----------------------------------------------------------RLFP.....IS..QV.---------------.VIR..PA...RTI.CIDCNN.VIDATT-------S.VEV.MSN..PR...V...LP.A...I..GCNN
PvSdr4L6	---------------------------------------------------------------------VFRFTV.---------------.VIR..PA...RT..FIDCSN-I..ATT-------SVVEV.VSN..PR...V...LP.T...I..GCNN
PvSdr4L7	G-----------------------------------------------------------RLFP.....ISN.QV.---------------.VIK..PA...RT..FIDCSN-VI.ATT-------SEVEV.VSN..PR...V...LP.A...I..GCNN
SiSdr4L	LPLA.--LPPATTV----------------...LVKVAAE-------------------ERD.P............---------------.VIS.......C...Y..R--.HRT---------DATCT-.VVS...A...V...A-.A.........-
SbSdr4L1	LPLA--SLPPASAG----------------.E.LAKVAAA------------------.GRD.P............---------------.VIS.......C.A...GC--.HRT---------DATCT-...S...AQ......V-.A.........-
SbSdr4L2	.------------------------------------------------------------A.P.....IS..QV.---------------.VIK..PA..LCT..CID.SN.ADAVDGGGVAYPE.TSTVSVSS...R...T...LPGA......GHHH
BdSdr4L1	-----------------------------------M.E--------------------DDRD.P............---------------.VIS.......S.....LEPIVVP.AGT------DNIHVGNV.S...........A-EA.....A...-
BdSdr4L2	LSLAP--------A.---------------..NL....TEMPAP-----------.EDDNRD.P............---------------.VIS......L...V.ILEP-VCAVAGT------NNDNTY..SS...Q....A..A-.A...L.....-
ZmSdr4L3	LPLA.ASLPPAGAGT---------------.E.LAKVAAE-------------------GRD.P............---------------.VIS.......C.A...GC--.HRA---------DATCT-..VS...VR......V-.A.......A.-
ZmSdr4L2	-------LPPASAG----------------VE.IAKAAAAAATE--------------.GRD.P............---------------RVIS.......W.A...GC--.HRTD--------DA.CTD..VS...VQ......V-.A........G-
ZmSdr4L1	.PSS.-------------------------------------------------.AATT.R.FP.....IS..QV.---------------.VIR..PA..LWT..CID.SN.A.VGSG-----PE.--..S.SN...R.......LPGA......GPR-
TaSdr41b	MPFA.--LPPATAST---------------G.NL.M..-.M.AG--------DEE.E..ERDIP............---------------.VIS...M...........--.VH.A--------VD..SST..S.........V.T-.A...I.T...-
TaSdr41d	MPFA.--LPPATAST---------------G.NL.M..T.M.AG--------DDE.E--ERD.P............---------------.VIS...M...........--.VP.A--------VD.TS-T..S.........V.T-.A.....T...-
TaSdr41a	MPFA.--LPPATAST---------------G.NL.M..-.M.AG--------DEE.E..ERDIP............---------------.VIS...M...........--.VH.A--------VD..SST..S.........V.T-.A.....T...-
AetSdr4L	MPFA.--LPPATAST---------------G.NL.M..-.M.AG--------DEE.E..ERDIP............---------------.VIS...M...........--.VH.A--------VD..SST..S.........V.T-.A...I.T...-
HVSdr4L	MPFA.--LPPATASA---------------S.NL....-.MLVGVG------DEE.E..ERD.P............---------------.VIS...M...........--.VH.A--------VD..SSTT.S.........V.T-.A.....T...-
AcSdr4L	VNL.TLPF.PYP.S-------------SVPEVPK.VPQMHGINMYS--QLQDVP.LN-SNSAIPQ.M...LN.HPS---NSATLNCGN--G.VIT.Q.......S.T..C--.QEDHNS------NS---.SSVL..PKD....I.L-.A........R-
StSdr4L	SNSITLPLM.CTTT----------------TLPMLVEKNSGE.IRG-------I.LN-LAADGPE.L.FMPQ.QG-----PKTPGP-----GVIT.QP......S.SIGC--.NEEEAP------EGGGTNKKFM.KP....E.V.A-EA....I...N-
SlSdr4L	SNSITLPLM.CTTT----------------TLPMLVEKHSGE.IRG-------I.LN-LAADGPE.L.FMPQ.QG-----PKTPGP-----VVIT.QP......S.SIGC--.NEEEAP------DGG.TNKKFI.KP....E.V.A-EA........N-
VvSdr4L	G-LMTLPL.PCPP-----------------------------------------------PQLP..K...QQ.QR-----APANTT-----NVIS.QP...I..S.S.RC--MRDDLSP------IP--EVQILK.-P....E.M..-ET....I...N-
EgSdr4L	RSL.TLPL.PCSPELN-------------CMNTVRVEEAAA.D----------LNVNSAVETIPK.K...LQ.QG-----PASGSG-----NVIS..P......R.S.GC--.SED.RP------DSP--.PR.RQKP....E.V..-EA....I...K-
PtSdr4L2	SNL.TLPL.QSP-------------TVPVVENQAAAPELSCQ.PNR----DKVI.LN-TVAETSE...P.QQ.Q.-----PST--S-----NVIA.QP....C.S.S.A.--.NEDPSL------IP---PVKV..KP..I.E.V..-EV..I.IT..N-
PtSdr4L1	SNL.TLPL.QSPT------------TVTVV.NQAAVPELSCM.PNR----DKVI.LN-TVAEFPE.K...QQ.QV-----PPT--N-----NVIA.QPL.L...S.SIAC--.SEDPSF------IP---LVRV..KP....E.V..-EV..T.I...N-
LuSdr4L1	SSL.TLPL.S-----------------PSA.FQGMQPHELMRCMAP--SQERDI.LNTVV-EIPE.K....Q.Q.R----HTTTN-------VIA.QP....V...R.GC--.NETPG-------VP---PVQYQ.MP.Q..E.V..-.V....I...N-
LuSdr4L2	SSL.TLPL.S-----------------PSA.FQGMQPHELMRCMAP--SQERDI.LNTVV-EIPE.K....Q.Q.R----HATTTN-----VVIA.QP....V...R.GC--.NETPG-------VP---PVQYQ.KP.Q..E.V..-.V....I...N-
MeSdr4L	SDL.TLPL.PCQP------------SVPAV-----APEISCM.PRK----EVVI.LNTTVAEIPE.K...QQ.Q.-----TLT--T-----NVIA.LPI.......S.GC--.SEDPSS------IP---PLQL..KP....Q.V.C-E.....I...K-
RcSdr4L	SDL.TLSL.PCQP------------PVPVVDNQV.APEISCM.ARR----QVII.LN-TVAEIPE.K.F.QQ.Q.-----PPT--N-----NVIA.QPI.....S.S.GC--VSEDTNS------.P---QVQV..KP....E.I.C-EA..T.IC..N-
CpSdr4L	PSL.TLPL.PCSP------------SVPVILNQA.TGQ.SCM.T---------I.LN-KVAEIPE.K...QQ.QT-----PPTTIS-----NVMK.QPI.....R.S.GC--.NEDPNL------FS---TVQI.RK.....D.M..-EA....I...N-
GrSdr4L1	.SLMQLPL.PCPP------------SVPVV.NYA.I.ELNCM.PCGG---EKLI.LN-TIVEIPE.K...KQ.QG-----PPA--S-----NVIV.QPI.....I.Y.GC--.KENPIL------.P---QMQVL.KR....ELV..-.A....I...N-
GrSdr4L2	TSL..LPL.P--------------------------------------------CLKVAAHEIPE.K.F.KQ.QGL----PVLPTS-----S.IT.QPI.......I.GC--.NEAPAP------AA---PLQ...KP....DDI..-E.M.TII...N-
TcSdr4L	.SL.TLPL.PCPP------------SGPIA.NKA.LPALNCM.PCGG---EKVI.LN-TVAEIPE.K...KQ.QG-----PVT--S-----GVIA.QPI.....S.S.GC--.SEDPSL------.P---PMQV..KS....E.V..-EA....I...N-
AtSdr4L	STL.TLPL.QCSP.---------------S-SKCMEPEIKGKG---------VI.LN-KTAE.IQ...F.TQ.QG.--ITTTTTATT---SRVIS.QPI...C.K.N.AY--.NPLTNP------SPT-S-QTSK.SPR...EDV..-.D..S.IT..N-
BsSdr4L1	SAL.NLPL.QCSP.---------------LPSKCMEPEIKGKG---------VI.LN-KTAE.IQ...F.KQ.QG.--ITTTTTTTTTTTSRVIS.QPI...F.K.N.AY--.NPLINP------SPL-P-QTSN.SPS...E.V..-.D..S.IT..N-
BrSdr4L1	PSL.TLPL.QCSPS---------------PPSKCMEPEIK.K.---------AI.LN-KTAE.IQ...F.KQ.Q..--ITTTT------TS.VIA.Q.I...C.R.N.AC--.NPLTNS------S-----Q.IK.SPQD..E.F..-.DV..II...N-
BrSdr4L2	PAL.TLPL.QCSP----------------LSSKCMEPEIK.KG---------LI.LN-KSAETIQ...F.KQ.QG.--ITTTT--AAE-ASRVIT.QPI...C.R.N.AC--.NPLSNP------S-----QISK.SPQ...E.V..-.V...II...N-
CgSdr4L1	SAL.TLPL.QCSP.---------------LPSKCMEPEIKGKG---------VI.LN-KTAE.IQ.I.F.KQ.QG.--ITTTTTTT----SRVIS.QPI..LC.K.N.AY--.NPLTNP------SPL-PNQTSK.SPS...E.V..-.D..S.IT...-
CrSdr4L1	SAL.TLPL.QCSP.---------------LPSKCMEPEIKGKG---------VI.LN-KTAE.IQ.I.F.KQ.QG.--ITTTTTTT----SRVIS.QPI..LC.K.N.AY--.NPLTNP------SPL-PNQTSK.SPS...E.V..-.D..S.IT...-
EsSdr4L	SAL.TLPL.QCSP.---------------PPSKCMEPEVKGKV---------VI.LN-KTAE.IQ...F.KQ.QG.--TVTTTATDTN-TSRVIA.Q.I...C.R.N.AC--.NPLTNP------SPP-Y-QISK.LPQ...E.V..-.D..TII...K-
CcSdr4L	--L.TLPL.PCTPV----------VSEQTTPLELSNCVESFG..N-------VI.LN-KVAEIPE.K...LQ.QG-----PPSPTN-----NVIS..P......S.S.GC--.NEDSRL------.P--E.QVVT.KP....E.V..-EA....I...H-
CsSdr4L	--L.TLPL.PCTPV----------VSEQTTPLELSNCVESFG..N-------VI.LN-KVAEIPE.K...LQ.QG-----PPSPTN-----NVIS..P......S.S.GC--.NEDSRL------.P--E.QVVT.KP....E.V..-EA..T.I...H-
CSaSdr4L	--L.TLPL.PS..D----------ETETTTPVSEINFIKSFD.EKG---VEFSV.SS-VVSEIPQ.K...QQ.Q------CPVSIS-----NVIT.HP......S.R.GC--.NEAQNP------VHSNNTPQL..KPD...K.V..-EV....I...N-
FvSdr4L	TSL.TLPL.SYP.SVP------IVPNQAVVTNQAVVPAELNLLKPS--GGEKLI.LNSVA-EIPE.K...KQ.QGTPSPATTPTSP-----NVIA.QPI.....S.S.GC--.STDPSS------AP---.VQ...KP.D..K.M..-EA...II...H-
GmSdr4L1	PSL.TLPL.PCSP-----------TLT-----------TKPCAG.-------VINLN-TKAS.PE.K...QQ.QK-----PVSNNI----INVIT.QPI..I..S.S.VC--.SEDSTL------SP---L.QT..KPN...Q.V.N-EA..T.I...N-
GmSdr4L2	PSL.TLPL.PCSP-----------CPAPKLE--.NSTLTKPCVG.-------VI.LN-TKVS.PE.K...QQ.QK-----PVSSN-----INVIT.QP...I..S.S.VC--.SEDLTL------PP---LSQT..RPN...Q.V.N-EP....I...N-
MdSdr4L1	T.XMTLPL.PYIP.----------ASAPIVTNPAVVPPEFELIKPCRGGEEELI.LNTVAAEIPE.K...KQ.QGN-SPAPPPTPT-----NVIA.QPI.....S.S.GC--.SADPSL------.L---..QV..KP.D..E.M..-EA...II...H-
MdSdr4L2	.ALMTLPL.PYIP.----------SSVPIATNQAVVPAELELMKPC-GGEENLI.LNIVAAEIPE.K...KQ.QGN-SPAPPPTNN-----NVI..QPI.LX..N.S.GC--.SADPSL------AP---..Q...KPDD..E.M..-KA....I...H-
MtSdr4L	PNL.TLPL.PCSPN----------SPN--------NNNALKF.L.-------VI.LNNTKVE.PQ.....QQ.QK-----PASSTNNV--INVIS.QPI..I..C.N.GC--.SEVSAI------PC---VTKT..KP..L.Q.V..-EE....I...N-
PhvSdr4L	PSL.TLPL.PCSPG----------APAPKLDSITATTTTKPCGGG-------VI.LN-TKAS.PE.....QQ.QK-----PVSNN-------VIQ.HP...I..S.T.VC--.GEDSTL------P-----SQT..RSQ.....V..-E...V.I...N-
PpSdr4L	TSLMTLPL.QYPP.PPSPPPPPPTSSVPVVTNQAMVPAEFELIKPC-EGEEKLI.LNTVA-EIPE.K...QQ.QG---SAPTPTTI-----NVIA.QP......S.S.GC--.KTDPSL------AP---.EQ...KP.D..D.V..-EA...II...H-
.	                                                                     : .:                   :: * . *     : :                           :    :*  .*    :* :: .   
490	500	510	520	530	540	550	560	570	580	590	600	610	620	630
....|....|....|....|....|....|....|....|....|....|....|....|....|....|....|....|....|....|....|....|....|....|....|....|....|....|....|....|....|....|....|.....|.....|............|     |         |
OsSdr4	NRVRLVNDAYKRMVGQPECPWLDAVAT---------AASRRISGEVALVVSEPAAA-------AAALPETC-KGFSCSAKIAWERD-GKW-------------SSVHAPCDVTRLQCE-SRDYVFAWRFRAAGDECNTH--RRAAGDA----
PvSdr4L1	...........E..............A----------...........Q.DV-----------..L...PH-EVLT.T.R.E..YG-..Y-------------T.IM.....S..L..-....L.T....T--ADADVSAG..SGDGEAIDS
PvSdr4L2	...........E..............A----------.........V.LLA.-----------..L...PH-EVLT.T.R.E..YG-.RH-------------LHLGT-----------...NL.T....T--TDADASVG..SGDGEASDS
PvSdr4L3	...........E..............A----------.........V.Q.A.-----------..L...PH-EVLT.T.R.E..YG-.RC-------------TCIMV....R..L..-....L.T....T--ADADVSVGC.SGDGEASDS
PvSdr4L4	...Y.A.....A.....V.....SLPG-AGA-------....N...V.S.GAFST.LP---------LSSTGCA.PGT.R.S----------WERE----EASA.LTV..A.E..TSN-CN..CYI...DSKKASIMYCIT-----------
PvSdr4L5	.H.Y.A.....A.....I.....SLPG-AGP-------..W.N...V.S.G------------------DIGCA.P.T.R.S----------WERE----DAKA.LTV..A.E..TSN-CNG.C.I...DSKKASIMYCIT-----------
PvSdr4L6	H..Y.A.....A.....I.....SLPG-AGA-------....N...V.S.G.FSNILH---------LPSTECA.P.T.R.S----------WERE----DASA.LTV..A.E..TSN-CN..C.I...DCEKASIMYCIA-----------
PvSdr4L7	H..Y.A.....A.....I.....SLPG-AGA-------....N...V.S.G.FSNILH---------LPSTECA.P.T.R.S----------WERE----DASA.LTV..A.E..TSN-CN..C.I...DSEKASIMYCIT-----------
SiSdr4L	...........E....S.........A----------.............G.-----------P.S...PH-GV.T.T.R.E..YG-..C-------------T.IL.....S.....-....L.T....T--VDADASVG..SG--ETSDS
SbSdr4L1	...........E..........Y...A----------T............AD-----------QSS....Y-GV.T.T...E..D.-..V-------------T.IAV....S..H..-....L.T....T--ADADASVGHSSE--EISES
SbSdr4L2	...H.......A.....V.....YLPGGAGAG--VSTT....N.I.V.D.RKFGP.AAPPRRPPDVVGGSVDAA.P.T.R.T----------WEQGGG--NAIA.LTV..T.EH.IGSR.G..RYI...DSSRASIIYCIT-----------
BdSdr4L1	S..........E...A.A....G----------------------------------------------------..A...E...G-.ER-------------A..N.A...I.....-....I...S..T.DASSSVS-HH..V-------
BdSdr4L2	...........K...A...A..S.L.AA--------..............PA-----------..MM..SQNG....A...E..LRG.ER-------------A....A......H..-A...L....L.T.D.ASPSSVSH..DK------
ZmSdr4L3	...........E..............A----------T............AN-----------QSS...SY-GV.T.T...E..D.-..V-------------A.IDV....S.....-..E.L.V....T.DADADASVGCSSE--EISES
ZmSdr4L2	...........E..............A----------T............AD-----------RSS..DSY-GA.T.T...E..D.-..V-------------T.IA.....S.....-....L......T.AADADASVGHSSE--EISES
ZmSdr4L1	...H.......A.....V......LPG-AGA-------....N.I...D.RTFGP.PRLPKN-----AGSSSDA.P.T.R.T----------WEHGGG--SAIA.LTV..A.EH.AGG-.G..R.I...DSSRASIIYCIA-----------
TaSdr41b	...........E...A...L..G...A-----------............A.-----------Q.T...SP-G....T...E..CGG.ER-------------A.I..A...S.....-Y.H.L........DASSPAD-SH..G.E.----
TaSdr41b	...........E...A...L..G...A-----------............A.-----------Q.T...SP-G....T...E..CRG.ER-------------A.F..A...S.....-Y.H.L......T.DASSSGS-SH..G...----
TaSdr41a	...........E...A...L..G...A-----------............A.-----------Q.T...SP-G....T...E..CGG.ER-------------A.I..A...S.....-Y.H.L........DASSPAD-SH..G.E.----
AetSdrL	...........E...A...L..G...A-----------............A.-----------Q.T...SP-G....T...E..CGG.ER-------------A.I..A...S.....-Y.H.L........DASSPAD-SH..G.E.----
HvSdr4L	...........E...A...L..G...A-----------............A.-----------K.....SP-E....T...E..CGT.ER-------------T.IQ.A...S.VH..-..H.L.....HT.DASSPAS-NH..D...----
AcSdr4L	.....A.S..ME.......L..NSM.NFDGQAR--SS.CK..G.K.M.DLA-------------DSQIPVFPDA.K.K.R.----------EWGSNEE----K..INVH...VK.S..-.KNHL.T...HTKECKF.SQS------------
StSdr4L	.K...T.A...E.......C...YMVGN---------.CK..G...I.EFLD-----------SSCSVPMSSD..N.WV..----------EWGAAQG---KKN..K.F.NAVK.A.Q-.K....E...HTTD.DAPASAASNN--------
SlSdr4L	.K...T.A...E.......C...YMVGN---------.CK..G...I.EFLD-----------SSCSVPMSSD..N.WV..----------EWGAAQG---KKN..K.F.NAVK.A.Q-.K....E...HTTD.NTPESAASNI--------
VvSdr4L	.KI.MA.S...E..........NSMV.C---I----VL.TFVDALLTIIYEG-----------RFCFLVQMINNLP.ELMFG--------GKWAIV----FKRTICR------.WDVQ-GH..DTE--------------------------
EgSdr4L	....V..S...E..........E.M.RLGAAG------G...G....IRMCD-----------EE.KVPVWAE....W.R.----------EWVGSGG---EK...K.F..AI..R.D-.K..L.T...HTNAARPSSEFSSS.V-------
PtSdr4L2	.K...A.S...E.......S..GSMM.SDGRF--AGSSCK..C...VFHL.-------------DLRVPESSN....WVR.----------EWCNKVK----SNVINTF...I..S..-.K..L.R...HIRTSKDSLSKTDA---------
PtSdr4L1	.K...A.S...E.......S...SMM.GDGSF--AGRSCK..C...E.HL.-------------DLRVPASSN....WVR.----------EWCNKGT----KNVIITF...I..S.A-....L.S...HTR.RKDFQSKTNA---------
LuSdr4L1	.K...A.S...E.......S..NLMV.GNDRLGFGSSCCQ........HL.-------------DSKVPVSSN....WVR.----------EWGSQGK----K...N.F...I..S.K-.K..L.T...HTPARQGSRISSNV---------
LuSdr4L2	.K...A.S...E.......S..NLMV.GNDRLGFGSSCCQ........HL.-------------DSKVPVSSN....WVS.----------EWGSQGK----K...N.F...I..S.K-.K..L.T...HTPARQGSQISSNV---------
MeSdr4L	Y....A.S...E.......S...LMV.GDGRI--GGSSCK..C.....HLP-------------DSKVP.SSN....WVR.----------EWGEEGK----KK.INSF...I..S..-.K..L.T...HTHNR.GSQSSTTA---------
RcSdr4L	YK...A.S...E...........SMVAGDGRF--GGNSCK..C...I.HLA-------------DSRVPSSSNK...WVN.----------EWGHEGK----KTAAT.F...I..S..-.K..L.....HTHSR.GSQSSTKA---------
CpSdr4L	.K...A.S..RK.......S..E.MV.S-GER--GSSSCK..C...G.HFC-------------DSRVPVSSN....WVR.----------DWGSDGK----NN.I..F...V..S.L-.K..L.T...HTREAARFRSNV-----------
GrSdr4L1	.K...A.S...E...........S.V.GEGRA--LGNSCK..C...V.HL..-----------SDSRLTVKS.....W.R.----------EWGSEGK----KR..K.F.E.IK.S.R-.K..L.T.K.QPYQQ.EGRITF-----------
GrSdr4L2	.K...A.S...A...........SMVKG--------SECK..C...M.NL.-------------NSRVPVKS.....WVR.----------EWGNEGNNSNNKG.IT.F...V..S.Q-.K..L.T...HIPTIG--KTS------------
TcSdr4L	.K...A.S...E...........SMV.VEGRA--GGNSCK..C...M.HL.-------------DSRVPVTSNR...WVR.----------DWGSDGK----K..IN.F...I..S.Q-.K..L.T...HTHNR.ATHSSCNV---------
AtSdr4L	S......S...E.M.....S...SMVRG-----------K..C...MINFC.-----------SKIPVM.ENN....WVR.----------DWGRDGK----EEYM..F....K.A.D-.K....T...HTTT.RR----------------
BsSdr4L1	.......S...E.M.....S...SMVRG-----------K..C.G.MIHFC.-----------SKIPVM.ENN....WVR.----------EWGRDGK----EEYM..F...MK.A..-.K.H..T...HTTT.RRE.CQS--SCNV-----
BrSdr4L1	.......S...E.M.....S...SMVRG-----------K..C...MIRCC.-----------.EIPE---NN....WVR.----------EWGRDGK----EEF...F...MK.E.D-.K....T...HTTT--RENLSTKLSCLV-----
BrSdr4L2	.K.K...S...E.M.....S...SMVKV-----------K..C...VIQFC.-----------SKISEK--NN.Y..WV..----------EWGRDGK----EEL...F...MKRE.D-.K....T...HITA--KE.CQP--SYNA-----
CgSdr4L1	.......S...E.M.....S...SMVRG-----------...C...MIHFC.-----------SKIPMM.ENN....WVR.----------EWGRDGK----EEYM..F...MK.A..-.K....T...H.TT.RRE.CQS--SCNV-----
CrSdr4L1	.......S...E.M.....S...SMVRG-----------...C...MIHFC.-----------SKIPMM.ENN....WVR.----------EWGRDGK----EEYM..F...MK.A..-.K....T...H.TT.RREACQS--SCNV-----
EsSdr4L	.......S...E.M.....S...SMVRG-----------K..C...MIHLC.-----------TKIPE---NN....WVR.----------EWGRDGK----EEF...F...MK.A.D-.K....T...HTRT--RE.CQS--SCNA-----
CcSdr4L	....MA.S.F.E.......S...LMVIN---N----SSCK..C...M.NF.D-----------S.GM.NNSSN....WVR.----------EWGSD----GKKN..D.F...I..S..-IK..L.....HPQASQSCC.V------------
CsSdr4L	....MA.S.F.E.......S...LMVIN---N----SSCK..C...M.NF.D-----------S.GM.NNSSN....WVR.----------EWGSD----GRRTR-----------------------------------------------
CSaSdr4L	....MA.S...E.......L...SMV.GDE-R----LKG...G...M.HL.D-----------...V.-HSSN....WVR.----------EWGNSD---GKKN..T.F...IK.S.V-....L.T...HTQTRNNHNNNNNAFNPICINV-
FvSdr4L	.K..MA.A...E.......S...SMVAT---------PCK........QL.-------------DTRVP.SSN....WVR.----------EWGNEMD----KHAIN.F...IK.A..-.K..L.T...HTHSR.ASQSSSSEI.TCN----
GmSdr4L1	H.I.MA.S...E.....V....ESMGN----L----LQC.......T.NL.D-----------SSTVIP.SSN....WVR.----------EWLSEHNN-KKKNCIN.F...MK.A..-....L.T...HTRTTREASQSSCN.--------
GmSdr4L2	H.I.MA.S...E.....L....ESMVNN-NGG----GECK......T.HL.D-----------S-TIVP.SSN....WVR.----------EWQSEHNN-KK-NC.N.F...MK.A..-....L.T...HTRTTREASQSSCN.--------
MdSdr4L1	.K..MA.S...E............MVAGGDGR-FGG---K..N...S.QL.-------------DSGVPVSSN....WVR.----------EWGKDMN----KHAINTF...MK.S..-.K..L.E...HTHSR.GCQSSSSS.--------
MdSdr4L1	KK..IA.S...E..........XXIVAG-DGR-FGG---K......S.QL.-------------GSGVPVSSN....WVR.----------EWGNEMN----KHTINTF...MK.S..-.K..L.E...HTHSR.GCQSSRSS.--------
2Medicag2	....MA.S...E..........EIQCGS---------SCK......T.QL.D-----------S-SNIPISSN....WVR.----------EWENNG---QKKNC.N.F...VK.C.DQ......T...HTR-SREASQSSCN.--------
PhvSdr4L	H...MA.S...E.....L....ESMVNAGAGN----LQCK........HL..-----------S--NIP.SSN....WVR.----------EWQSDQ---QKKCC.N.F...MK.T..-....L.T...HTR-TREACQSSCT.--------
PpSdr4L	.K..MA.S...E.......S...SMVAS-DGR-FGGSSCK......I.EL.-------------ESGVPVSSN....WVR.----------EWGNEMN----KHAIN.F...IK.S..-TK..L.S...HTHSK.GSQSSSSN.--------
.	 :: :.* *:  *:* . * **                                                          :                                                                       


Additional file 8. Multiple sequence alignment of SDR4-like genes in monocot and dicot plants. The amino acid sequences of plant SDR4 proteins were aligned using CLUSTAL X (1.81). In the alignment, the residues are displayed in the “Difference Mode” with the “Diff/Consensus Line” style. Dots indicate conserved residues with the first protein OsSdr4, and “-” indicates gaps on the alignment. The critical amino acid sites responsible for functional divergence are shaded in red. The residues identified from the tests of positive selection are designated with green arrows. Motifs (1-6)  identified from MEME analysis are represented in parenthesis by various colors.
