# Supplementary material for: Phylogenetic Analysis, Lineage-Specific Expansion and Functional Divergence of seed dormancy 4-Like Genes in Plants
Source: PLoS One. 2016 Jun 14;11(6):e0153717. doi: 10.1371/journal.pone.0153717 (PMC4907471; doi:10.1371/journal.pone.0153717)

**S2 File.** The predicted average number of phosphorylation sites in the subgroup of Sdr4L proteins those noted in the phylogenetic analysis.

| Subgroup | Taxonomic family | Serine (S) | Threonine  (T) | Tyrosine (Y) |
| --- | --- | --- | --- | --- |
| Sub I | Grass 1 | 11.9 | 3.2 | 1.6 |
| Sub II | Grass 2 | 11.8 | 5 | 2.1 |
| Sub III | Pentapetalae | 11 | 9.5 | 2.5 |
| Sub IV | Malvidae | 17.8 | 5 | 2.3 |
| Sub V | Brasiccales Malvales | 33.7 | 4.6 | 1.7 |
| Sub VI | Brasicaceae | 20.3 | 10.1 | 4.1 |
| Sub VII | Citrus | 14 | 8.5 | 1 |
| Sub VIIIa | Fabidae1 | 16.8 | 6.3 | 2.3 |
| Sub VIIIb | Fabidae2 | 18.5 | 5.5 | 2.3 |

**The predicted serine, threonine and tyrsine phosphorylation sites in plant Sdr4-like proteins**

**SbSdr4L1**

MAMVQPADTAVKANEILARFRPIAPKPTLATAAAAASPVAQAAAEGVVAANRVLCHLQSRPCRARKRGRPTVVPVSPPKS 80

GSGAQSPAKRKRAATPYPPLRCAAATACSASAVVPVSARLPLASLPPASAGAEDLAKVAAAEGRDVPVERDLLRKLLEPK 160

VISPRAVRPVCSAIHVGCIHRTDATCTAAASKTAAQVEAELEVDALPAVVSDSSNRVRLVNDAYKEMVGQPECPWLYAVA 240

ATSRRISGEVALVVADQSSLPETYGVFTCTAKIEWEDDGKVTSIAVPCDVSRLHCESRDYLFTWRFRTADADASVGHSSE 320

EISES 400

........T...........................S.................................T....S...S 80

.....S........T.................................S............................... 160

..S.................................................S..........Y................ 240

..S...S...........S................................................T.....S...SS. 320

..S.. 400

Phosphorylation sites predicted: Ser: 14 Thr: 4 Tyr: 1

Serine predictions

Name Pos Context Score Pred

_________________________v_________________

10Sb02g0377 37 AAAASPVAQ 0.804 *S*

10Sb02g0377 76 VVPVSPPKS 0.985 *S*

10Sb02g0377 80 SPPKSGSGA 0.896 *S*

10Sb02g0377 86 SGAQSPAKR 0.850 *S*

10Sb02g0377 129 LPPASAGAE 0.873 *S*

10Sb02g0377 163 PKVISPRAV 0.936 *S*

10Sb02g0377 213 VVSDSSNRV 0.606 *S*

10Sb02g0377 243 VAATSRRIS 0.746 *S*

10Sb02g0377 247 SRRISGEVA 0.998 *S*

10Sb02g0377 259 ADQSSLPET 0.816 *S*

10Sb02g0377 314 DADASVGHS 0.667 *S*

10Sb02g0377 318 SVGHSSEEI 0.996 *S*

10Sb02g0377 319 VGHSSEEIS 0.961 *S*

10Sb02g0377 323 SEEISES-- 0.655 *S*

_________________________^_________________

Threonine predictions

Name Pos Context Score Pred

_________________________v_________________

10Sb02g0377 9 QPADTAVKA 0.850 *T*

10Sb02g0377 71 RGRPTVVPV 0.970 *T*

10Sb02g0377 95 KRAATPYPP 0.987 *T*

10Sb02g0377 308 WRFRTADAD 0.760 *T*

_________________________^_________________

Tyrosine predictions

Name Pos Context Score Pred

_________________________v_________________

10Sb02g0377 224 VNDAYKEMV 0.931 *Y*

_________________________^_________________


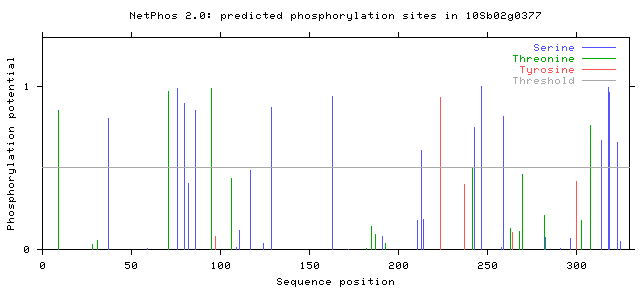


**SbSdr4L2**

MERRLRAIAPKPLPPPAPPRSLLWRGHKRGRDDHLLLSPPVSKREREATSSSSSSSSYPYPYPYPPPPLLPAAGVGLGRY 80

MSMPEGVLAGCEERLRGLSLVAGSPAAAAAAVPVERDLISKLQVPKVIKPRPARPLCTTICIDSSNIADAVDGGGVAYPE 160

TTSTVSVSSKTAREVETELELPGALPAVVSGHHHNRVHLVNDAYKAMVGQPVCPWLDYLPGGAGAGVSTTSRRINGIVVL 240

DVRKFGPAAAPPRRPPDVVGGSVDAAFPCTARITWEQGGGNAIASLTVPCTVEHLIGSRSGDYRYIWRFDSSRASIIYCI 320

T 400

.....................................S...S......TSSSSSSSSY...................... 80

.S................S....S........................................................ 160

.T...S.SS.T.....T..........................Y.........................TS......... 240

.....................S................................................S...S..... 320

. 400

Phosphorylation sites predicted: Ser: 20 Thr: 5 Tyr: 2

Serine predictions

Name Pos Context Score Pred

_________________________v_________________

11Sobic001G 38 HLLLSPPVS 0.620 *S*

11Sobic001G 42 SPPVSKRER 0.994 *S*

11Sobic001G 50 REATSSSSS 0.974 *S*

11Sobic001G 51 EATSSSSSS 0.936 *S*

11Sobic001G 52 ATSSSSSSS 0.753 *S*

11Sobic001G 53 TSSSSSSSS 0.972 *S*

11Sobic001G 54 SSSSSSSSY 0.993 *S*

11Sobic001G 55 SSSSSSSYP 0.948 *S*

11Sobic001G 56 SSSSSSYPY 0.982 *S*

11Sobic001G 57 SSSSSYPYP 0.850 *S*

11Sobic001G 82 GRYMSMPEG 0.986 *S*

11Sobic001G 99 LRGLSLVAG 0.956 *S*

11Sobic001G 104 LVAGSPAAA 0.700 *S*

11Sobic001G 166 TSTVSVSSK 0.957 *S*

11Sobic001G 168 TVSVSSKTA 0.992 *S*

11Sobic001G 169 VSVSSKTAR 0.967 *S*

11Sobic001G 231 VSTTSRRIN 0.757 *S*

11Sobic001G 262 VVGGSVDAA 0.527 *S*

11Sobic001G 311 WRFDSSRAS 0.960 *S*

11Sobic001G 315 SSRASIIYC 0.916 *S*

_________________________^_________________

Threonine predictions

Name Pos Context Score Pred

_________________________v_________________

11Sobic001G 49 EREATSSSS 0.927 *T*

11Sobic001G 162 YPETTSTVS 0.950 *T*

11Sobic001G 171 VSSKTAREV 0.953 *T*

11Sobic001G 177 REVETELEL 0.747 *T*

11Sobic001G 230 GVSTTSRRI 0.674 *T*

_________________________^_________________

Tyrosine predictions

Name Pos Context Score Pred

_________________________v_________________

11Sobic001G 58 SSSSYPYPY 0.792 *Y*

11Sobic001G 204 VNDAYKAMV 0.869 *Y*

_________________________^_________________


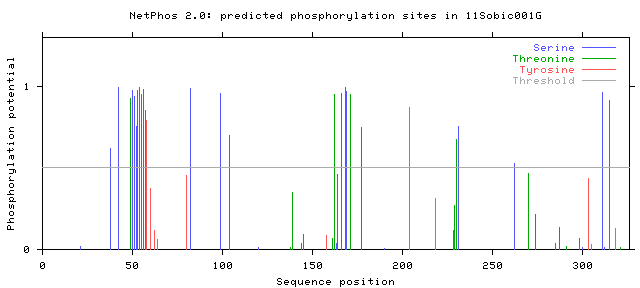


**BdSdr4L1**

MAMVQPADVAVKANEILARLRPIAPKPPAALMTTSPAQTIGGAAASRVLSQLQSRPCRARKRGRPPVSPLAARRKRPAAP 80

YPAPQLRCAAATDGAVVSTATRARVSVDGMLEDDRDVPVERDLLRKLLEPKVISPRAVRPVSSTIHVLEPIVVPGAGTDN 160

IHVGNVASKTAEEVEAELEAEALPAVVADSSSRVRLVNDAYKEMVGAPACPWLGSCAAKIEWERGGERASVNAACDVIRL 240

QCESRDYIFAWSFRTADASSSVSHHRAV 320

......................................T..........S.................S............ 80

.........................S...........................S.......S.................. 160

.........T...................S.S........Y............................S.......... 240

......Y...........SSS.S..... 320

Phosphorylation sites predicted: Ser: 12 Thr: 2 Tyr: 2

Serine predictions

Name Pos Context Score Pred

_________________________v_________________

12Bradi1g23 50 SRVLSQLQS 0.970 *S*

12Bradi1g23 68 RPPVSPLAA 0.971 *S*

12Bradi1g23 106 RARVSVDGM 0.991 *S*

12Bradi1g23 134 PKVISPRAV 0.936 *S*

12Bradi1g23 142 VRPVSSTIH 0.962 *S*

12Bradi1g23 190 VVADSSSRV 0.955 *S*

12Bradi1g23 192 ADSSSRVRL 0.507 *S*

12Bradi1g23 230 GERASVNAA 0.948 *S*

12Bradi1g23 259 TADASSSVS 0.824 *S*

12Bradi1g23 260 ADASSSVSH 0.898 *S*

12Bradi1g23 261 DASSSVSHH 0.952 *S*

12Bradi1g23 263 SSSVSHHRA 0.992 *S*

_________________________^_________________

Threonine predictions

Name Pos Context Score Pred

_________________________v_________________

12Bradi1g23 39 SPAQTIGGA 0.927 *T*

12Bradi1g23 170 VASKTAEEV 0.614 *T*

_________________________^_________________

Tyrosine predictions

Name Pos Context Score Pred

_________________________v_________________

12Bradi1g23 201 VNDAYKEMV 0.931 *Y*

12Bradi1g23 247 ESRDYIFAW 0.604 *Y*

_________________________^_________________


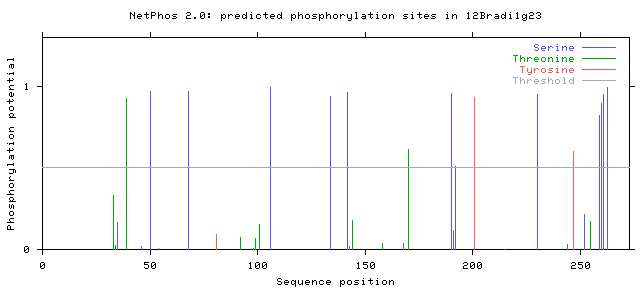


**BdSdr4L2**

MALVQPADMAVKANEILARFRPIAPKPPSAMPTQQPIGGAGAGTSRVLSHLQARPYRARKRGRPSVAPPVPPPPHAAAAR 80

RKRTAVVPYPAPPLGCAAPTDAVVSTAMRAGSACLSLAPASAGNLTRLSTEMPAPEEDDNRDVPVERDLLRKLLEPKVIS 160

PRAVRPLGSTVHILEPVCAVAGTNNDNTYAASSKTAQEVEAALEADALPALVSDSSNRVRLVNDAYKKMVGAPECAWLSA 240

LAAAAASRRISGEVALVVPAAAMMPESQNGGFSCAAKIEWELRGGERASVHAACDVTRLHCEARDYLFAWRLRTADDASP 320

SSVSHRADK 400

............................S..............T....S...............S............... 80

...T...........................S................ST.............................S 160

............................Y...S................................Y.............. 240

..........S.....................................S........................T....S. 320

SS.S..... 400

Phosphorylation sites predicted: Ser: 13 Thr: 4 Tyr: 2

Serine predictions

Name Pos Context Score Pred

_________________________v_________________

13Bradi1g23 29 PKPPSAMPT 0.503 *S*

13Bradi1g23 49 SRVLSHLQA 0.991 *S*

13Bradi1g23 65 RGRPSVAPP 0.984 *S*

13Bradi1g23 112 MRAGSACLS 0.501 *S*

13Bradi1g23 129 LTRLSTEMP 0.992 *S*

13Bradi1g23 160 PKVISPRAV 0.936 *S*

13Bradi1g23 193 YAASSKTAQ 0.991 *S*

13Bradi1g23 251 SRRISGEVA 0.997 *S*

13Bradi1g23 289 GERASVHAA 0.984 *S*

13Bradi1g23 319 ADDASPSSV 0.969 *S*

13Bradi1g23 321 DASPSSVSH 0.878 *S*

13Bradi1g23 322 ASPSSVSHR 0.799 *S*

13Bradi1g23 324 PSSVSHRAD 0.992 *S*

_________________________^_________________

Threonine predictions

Name Pos Context Score Pred

_________________________v_________________

13Bradi1g23 44 AGAGTSRVL 0.970 *T*

13Bradi1g23 84 RRKRTAVVP 0.783 *T*

13Bradi1g23 130 TRLSTEMPA 0.651 *T*

13Bradi1g23 314 WRLRTADDA 0.886 *T*

_________________________^_________________

Tyrosine predictions

Name Pos Context Score Pred

_________________________v_________________

13Bradi1g23 189 NDNTYAASS 0.840 *Y*

13Bradi1g23 226 VNDAYKKMV 0.888 *Y*

_________________________^_________________


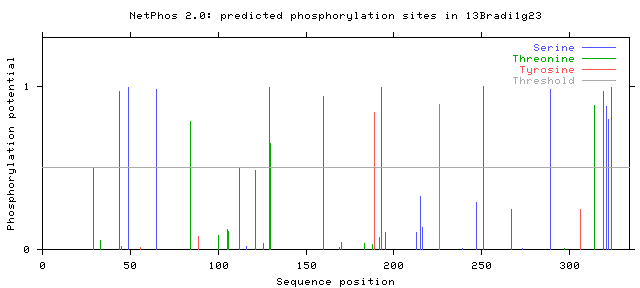


**ZmSdr4L3**

MAMVQAADAAVKANEILARFRPIAPNPTLAAEGVVAANRVLCHLQSKPCRARKRGRPGPAVVAPSPESGLQPPAKRKRAT 80

TPYPPLRCRGPRASAAVPGSAGLPLASASLPPAGAGTAEDLAKVAAEGRDVPVERDLLRKLLEPKVISPRAVRPVCSAIH 160

VGCIHRADATCTAAVSKTAVRVEAELEVDALPAVVSDASNRVRLVNDAYKEMVGQPECPWLDAVAATSRRISGEVALVVA 240

NQSSLPESYGVFTCTAKIEWEDDGKVASIDVPCDVSRLQCESREYLFVWRFRTADADADASVGCSSEEISES 320

................................................................S..S............ 80

T............S.....................................................S............ 160

................................................Y..................S...S........ 240

.......S.................................S..........T.......S...SS...S.. 320

Phosphorylation sites predicted: Ser: 12 Thr: 2 Tyr: 1

Serine predictions

Name Pos Context Score Pred

_________________________v_________________

14ZM2G10530 46 CHLQSKPCR 0.004 .

14ZM2G10530 65 VVAPSPESG 0.995 *S*

14ZM2G10530 68 PSPESGLQP 0.634 *S*

14ZM2G10530 94 GPRASAAVP 0.586 *S*

14ZM2G10530 100 AVPGSAGLP 0.025 .

14ZM2G10530 107 LPLASASLP 0.026 .

14ZM2G10530 109 LASASLPPA 0.018 .

14ZM2G10530 148 PKVISPRAV 0.936 *S*

14ZM2G10530 157 RPVCSAIHV 0.003 .

14ZM2G10530 176 TAAVSKTAV 0.090 .

14ZM2G10530 196 PAVVSDASN 0.022 .

14ZM2G10530 199 VSDASNRVR 0.110 .

14ZM2G10530 228 VAATSRRIS 0.746 *S*

14ZM2G10530 232 SRRISGEVA 0.998 *S*

14ZM2G10530 243 VANQSSLPE 0.007 .

14ZM2G10530 244 ANQSSLPES 0.220 .

14ZM2G10530 248 SLPESYGVF 0.797 *S*

14ZM2G10530 268 GKVASIDVP 0.140 .

14ZM2G10530 276 PCDVSRLQC 0.006 .

14ZM2G10530 282 LQCESREYL 0.510 *S*

14ZM2G10530 301 DADASVGCS 0.823 *S*

14ZM2G10530 305 SVGCSSEEI 0.981 *S*

14ZM2G10530 306 VGCSSEEIS 0.943 *S*

14ZM2G10530 310 SEEISES-- 0.655 *S*

_________________________^_________________

Threonine predictions

Name Pos Context Score Pred

_________________________v_________________

14ZM2G10530 81 KRATTPYPP 0.993 *T*

14ZM2G10530 293 WRFRTADAD 0.760 *T*

_________________________^_________________

Tyrosine predictions

Name Pos Context Score Pred

_________________________v_________________

14ZM2G10530 209 VNDAYKEMV 0.931 *Y*

_________________________^_________________


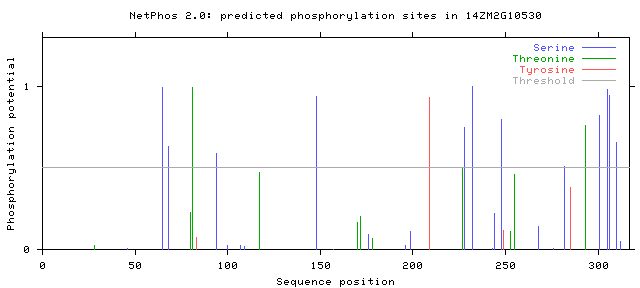


**ZmSdr4L2**

MAMVQPADTAVKANEILARFRPIAPKPTLAAAAAAAAAPVAQAAAEGVVAANRVLCHLQSRPCRARKRGRPTVVPVSPKS 80

GAQPPAKRRRASTPYPPLRCAAATTGAHVSAVVPGSARLPPASAGVEDIAKAAAAAATEEGRDVPVERDLLRKLLEPRVI 160

SPRAVRPVWSAIHVGCIHRTDDAACTDAAVSKTAVQVEAELEVDALPAVVSDSGNRVRLVNDAYKEMVGQPECPWLDAVA 240

ATSRRISGEVALVVADRSSLPDSYGAFTCTAKIEWEDDGKVTSIAAPCDVSRLQCESRDYLFAWRFRTAAADADASVGHS 320

SEEISES 400

........T..............................................................T....S..S 80

...........ST......................S......S..................................... 160

S...................................................S..........Y................ 240

..S...S...........S...S....................................................S...S 320

S...S.. 400

Phosphorylation sites predicted: Ser: 15 Thr: 3 Tyr: 1

Serine predictions

Name Pos Context Score Pred

_________________________v_________________

15ZM2G39640 77 VVPVSPKSG 0.998 *S*

15ZM2G39640 80 VSPKSGAQP 0.932 *S*

15ZM2G39640 92 RRRASTPYP 0.996 *S*

15ZM2G39640 116 VVPGSARLP 0.571 *S*

15ZM2G39640 123 LPPASAGVE 0.854 *S*

15ZM2G39640 161 PRVISPRAV 0.989 *S*

15ZM2G39640 213 VVSDSGNRV 0.723 *S*

15ZM2G39640 243 VAATSRRIS 0.746 *S*

15ZM2G39640 247 SRRISGEVA 0.998 *S*

15ZM2G39640 259 ADRSSLPDS 0.994 *S*

15ZM2G39640 263 SLPDSYGAF 0.954 *S*

15ZM2G39640 316 DADASVGHS 0.667 *S*

15ZM2G39640 320 SVGHSSEEI 0.996 *S*

15ZM2G39640 321 VGHSSEEIS 0.961 *S*

15ZM2G39640 325 SEEISES-- 0.655 *S*

_________________________^_________________

Threonine predictions

Name Pos Context Score Pred

_________________________v_________________

15ZM2G39640 9 QPADTAVKA 0.850 *T*

15ZM2G39640 72 RGRPTVVPV 0.970 *T*

15ZM2G39640 93 RRASTPYPP 0.993 *T*

_________________________^_________________

Tyrosine predictions

Name Pos Context Score Pred

_________________________v_________________

15ZM2G39640 224 VNDAYKEMV 0.931 *Y*

_________________________^_________________


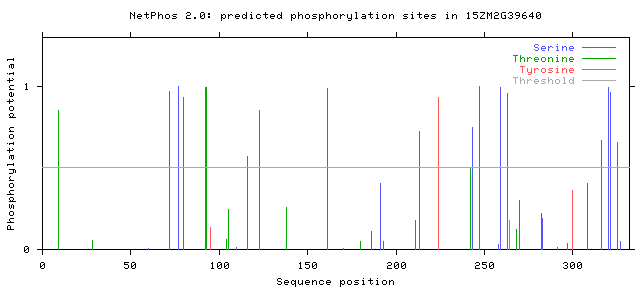


**ZmSdr4L1**

MPPLMPTGAAATSVLWRARKRGRDEDRLLLSPPLSKRETEREAAGASSYPNPQPPASAAPVAATRRGRRYVPMPEGLLTG 80

CEERLRRLSLVAGSSPAAALPWPAAAAAPSSSDAATTRRVFPVERDLISKLQVPKVIRPRPARPLWTTICIDSSNIAVVG 160

SGPETAASASNKTAREVEAELELPGALPAVVSGPRNRVHLVNDAYKAMVGQPVCPWLDALPGAGASRRINGIVALDVRTF 240

GPAPRLPKNAGSSSDAFPCTARITWEHGGGSAIASLTVPCAVEHLAGGSGDYRFIWRFDSSRASIIYCIA 320

..................................S...T.........Y..............T.....Y........T. 80

........S....................S.....TT..............................T............ 160

S......S.S..T...............................Y................................... 240

...........SS..............................................S...S...... 320

Phosphorylation sites predicted: Ser: 10 Thr: 7 Tyr: 3

Serine predictions

Name Pos Context Score Pred

_________________________v_________________

16ZM2G03899 35 SPPLSKRET 0.997 *S*

16ZM2G03899 89 LRRLSLVAG 0.996 *S*

16ZM2G03899 110 AAAPSSSDA 0.989 *S*

16ZM2G03899 161 AVVGSGPET 0.933 *S*

16ZM2G03899 168 ETAASASNK 0.648 *S*

16ZM2G03899 170 AASASNKTA 0.965 *S*

16ZM2G03899 252 KNAGSSSDA 0.994 *S*

16ZM2G03899 253 NAGSSSDAF 0.738 *S*

16ZM2G03899 300 WRFDSSRAS 0.960 *S*

16ZM2G03899 304 SSRASIIYC 0.916 *S*

_________________________^_________________

Threonine predictions

Name Pos Context Score Pred

_________________________v_________________

16ZM2G03899 39 SKRETEREA 0.995 *T*

16ZM2G03899 64 PVAATRRGR 0.829 *T*

16ZM2G03899 79 EGLLTGCEE 0.898 *T*

16ZM2G03899 116 SDAATTRRV 0.869 *T*

16ZM2G03899 117 DAATTRRVF 0.989 *T*

16ZM2G03899 148 PLWTTICID 0.539 *T*

16ZM2G03899 173 ASNKTAREV 0.855 *T*

_________________________^_________________

Tyrosine predictions

Name Pos Context Score Pred

_________________________v_________________

16ZM2G03899 49 GASSYPNPQ 0.554 *Y*

16ZM2G03899 70 RGRRYVPMP 0.666 *Y*

16ZM2G03899 205 VNDAYKAMV 0.869 *Y*

_________________________^_________________


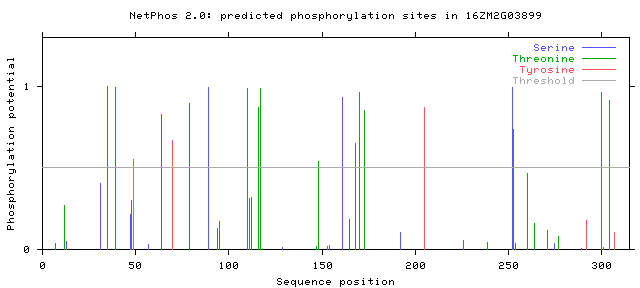


**KF021988**

MAMVQPVDMAVKANEILARFRPIAPKPALPASPAQAQAIDGAADRVLCHLQSRPCRARKRGRPSAVPVSAPAAAAKRKRA 80

AYPVPLRCAAAAATDAVVSTATRAYVSVPGSACMPFASLPPATASTGGNLTMLSTMVAGDEEEEEEERDIPVERDLLRKL 160

LEPKVISPRAMRPVGSTIHVESIVHGAVDAASSTAASKTAEEVEAEVETDALPAIVTDSSNRVRLVNDAYKEMVGAPECL 240

WLGAVAASRRISGEVALVVAEQATLPESPGGFSCTAKIEWECGGGERASIHAACDVSRLQCEYRHYLFAWRFRAADASSP 320

ADSHRAGGEA 400

...............................................................S................ 80

........................Y.S..................................................... 160

......S........................S....S................................Y.......... 240

.......S...S...............S....................S.............................S. 320

..S....... 400

Phosphorylation sites predicted: Ser: 11 Thr: 0 Tyr: 2

Serine predictions

Name Pos Context Score Pred

_________________________v_________________

17TaSdr4_1A 64 RGRPSAVPV 0.868 *S*

17TaSdr4_1A 107 RAYVSVPGS 0.819 *S*

17TaSdr4_1A 167 PKVISPRAM 0.980 *S*

17TaSdr4_1A 192 VDAASSTAA 0.738 *S*

17TaSdr4_1A 197 STAASKTAE 0.978 *S*

17TaSdr4_1A 248 AVAASRRIS 0.547 *S*

17TaSdr4_1A 252 SRRISGEVA 0.997 *S*

17TaSdr4_1A 268 TLPESPGGF 0.970 *S*

17TaSdr4_1A 289 GERASIHAA 0.971 *S*

17TaSdr4_1A 319 ADASSPADS 0.996 *S*

17TaSdr4_1A 323 SPADSHRAG 0.993 *S*

_________________________^_________________

Threonine predictions

Name Pos Context Score Pred

_________________________v_________________

_________________________^_________________

Tyrosine predictions

Name Pos Context Score Pred

_________________________v_________________

17TaSdr4_1A 105 ATRAYVSVP 0.862 *Y*

17TaSdr4_1A 230 VNDAYKEMV 0.931 *Y*

_________________________^_________________


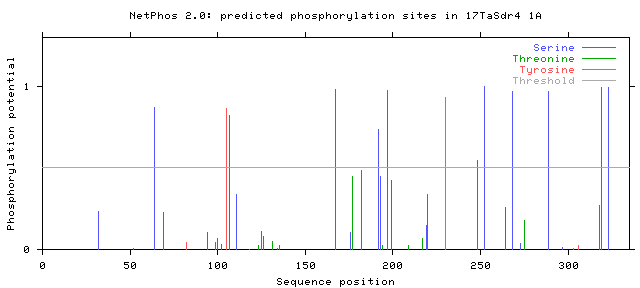


**KF021989**

MAMVQPADMAVKANEILARFRPIAPKPALPASPVQAIDGAADRVLCHLQNRPCRARKRGRPSAVPVSAPAAAAKRKRAAY 80

PVPLRCAAAAATDAVVSTATRAYVSVPGSACMPFASLPPATASTGGNLTMLSTTMVAGDDEEEERDVPVERDLLRKLLEP 160

KVISPRAMRPVGSTIHVESIVPGAVDATSTAASKTAEEVEAEVETDALPAVVTDSSNRVRLVNDAYKEMVGAPECLWLGA 240

VAASRRISGEVALVVAEQATLPESPGGFSCTAKIEWECRGGERASFHAACDVSRLQCEYRHYLFAWRFRTADASSSGSSH 320

RAGGDA 400

...............................S.............................S.................. 80

......................Y.S....................................................... 160

...S..............S.........S...S................................Y.............. 240

...S...S...............S....................S........................T...SSS.SS. 320

...... 400

Phosphorylation sites predicted: Ser: 16 Thr: 1 Tyr: 2

Serine predictions

Name Pos Context Score Pred

_________________________v_________________

18TaSDr41BK 32 ALPASPVQA 0.668 *S*

18TaSDr41BK 62 RGRPSAVPV 0.868 *S*

18TaSDr41BK 105 RAYVSVPGS 0.819 *S*

18TaSDr41BK 164 PKVISPRAM 0.980 *S*

18TaSDr41BK 179 IHVESIVPG 0.582 *S*

18TaSDr41BK 189 VDATSTAAS 0.886 *S*

18TaSDr41BK 193 STAASKTAE 0.989 *S*

18TaSDr41BK 244 AVAASRRIS 0.547 *S*

18TaSDr41BK 248 SRRISGEVA 0.997 *S*

18TaSDr41BK 264 TLPESPGGF 0.970 *S*

18TaSDr41BK 285 GERASFHAA 0.952 *S*

18TaSDr41BK 314 TADASSSGS 0.963 *S*

18TaSDr41BK 315 ADASSSGSS 0.961 *S*

18TaSDr41BK 316 DASSSGSSH 0.973 *S*

18TaSDr41BK 318 SSSGSSHRA 0.956 *S*

18TaSDr41BK 319 SSGSSHRAG 0.993 *S*

_________________________^_________________

Threonine predictions

Name Pos Context Score Pred

_________________________v_________________

18TaSDr41BK 310 WRFRTADAS 0.683 *T*

_________________________^_________________

Tyrosine predictions

Name Pos Context Score Pred

_________________________v_________________

18TaSDr41BK 103 ATRAYVSVP 0.862 *Y*

18TaSDr41BK 226 VNDAYKEMV 0.931 *Y*

_________________________^_________________


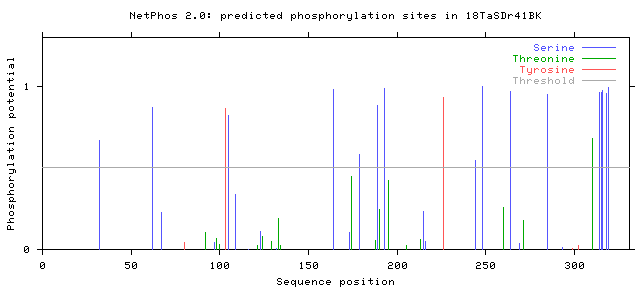


**KF021991**

MAMVQPVDMAVKANEILARFRPIAPKPALPASPAQAQAIDGAADRVLCHLQSRPCRARKRGRPSAVPVSAPAAAAKRKRA 80

AYPVPLRCAAAAATDAVVSTATRAYVSVPGSACMPFASLPPATASTGGNLTMLSTMVAGDEEEEEEERDIPVERDLLRKL 160

LEPKVISPRAMRPVGSTIHVESIVHGAVDAASSTAASKTAEEVEAEVETDALPAVVTDSSNRVRLVNDAYKEMVGAPECL 240

WLGAVAASRRISGEVALVVAEQATLPESPGGFSCTAKIEWECGGGERASIHAACDVSRLQCEYRHYLFAWRFRAADASSP 320

ADSHRAGGEA 400

...............................................................S................ 80

........................Y.S..................................................... 160

......S........................S....S................................Y.......... 240

.......S...S...............S....................S.............................S. 320

..S....... 400

Phosphorylation sites predicted: Ser: 11 Thr: 0 Tyr: 2

Serine predictions

Name Pos Context Score Pred

_________________________v_________________

19TaSdr_1AK 64 RGRPSAVPV 0.868 *S*

19TaSdr_1AK 107 RAYVSVPGS 0.819 *S*

19TaSdr_1AK 167 PKVISPRAM 0.980 *S*

.

19TaSdr_1AK 192 VDAASSTAA 0.738 *S*

19TaSdr_1AK 197 STAASKTAE 0.978 *S*

19TaSdr_1AK 248 AVAASRRIS 0.547 *S*

19TaSdr_1AK 252 SRRISGEVA 0.997 *S*

19TaSdr_1AK 268 TLPESPGGF 0.970 *S*

19TaSdr_1AK 289 GERASIHAA 0.971 *S*

19TaSdr_1AK 319 ADASSPADS 0.996 *S*

19TaSdr_1AK 323 SPADSHRAG 0.993 *S*

_________________________^_________________

Threonine predictions

Name Pos Context Score Pred

_________________________v_________________

_________________________^_________________

Tyrosine predictions

Name Pos Context Score Pred

_________________________v_________________

19TaSdr_1AK 105 ATRAYVSVP 0.862 *Y*

19TaSdr_1AK 230 VNDAYKEMV 0.931 *Y*

_________________________^_________________


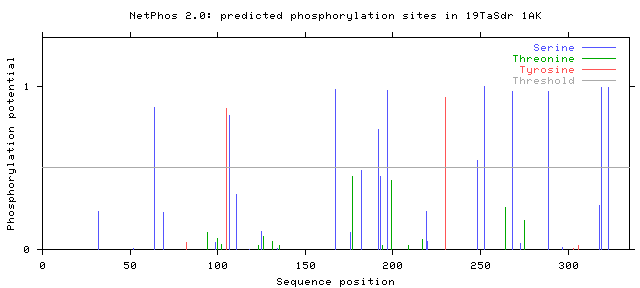


**OsSdr4L**

MAMVQPVDMAVKANEIMARFRPIAPKPVLPAAAAGVTGGGDGAAAVAATNRVLCQLQSRPCRARKRGRPSVVPPVSPPAG 80

AKRKRAPAYPVPVAPLRCAAVATATRARVSVVVVPAPESAGGVSALAPVSPSAGDSTRLSPTVVEVEDEDEERGVVLVER 160

DLLRKLLEPRKLLEPRAVRPVGSTIHVESVHIDVGRTTAAAAAAAPKTAEEVEAELESDSLPAVVSDSSNRVRLVNDAYK 240

RMVGQPECPWLDAVATAASRRISGEVALVVSEPAAAAAALPETCKGFSCSAKIAWERDGKWSSVHAPCDVTRLQCESRDY 320

VFAWRFRAAGDECNTHRRAAGDA 400

.....................................................................S.....S.... 80

.............................S...................S.S...S...S.................... 160

............................S........T.........T...................S..........Y. 240

......................S......................................S.................Y 320

..............T........ 400

Phosphorylation sites predicted: Ser: 11 Thr: 3 Tyr: 2

Serine predictions

Name Pos Context Score Pred

_________________________v_________________

1Rice 70 RGRPSVVPP 0.991 *S*

1Rice 76 VPPVSPPAG 0.801 *S*

1Rice 110 RARVSVVVV 0.963 *S*

1Rice 130 LAPVSPSAG 0.988 *S*

1Rice 132 PVSPSAGDS 0.988 *S*

1Rice 136 SAGDSTRLS 0.950 *S*

1Rice 140 STRLSPTVV 0.994 *S*

1Rice 189 IHVESVHID 0.551 *S*

1Rice 228 VVSDSSNRV 0.606 *S*

1Rice 263 SRRISGEVA 0.997 *S*

1Rice 302 DGKWSSVHA 0.507 *S*

_________________________^_________________

Threonine predictions

Name Pos Context Score Pred

_________________________v_________________

1Rice 198 VGRTTAAAA 0.836 *T*

1Rice 208 AAPKTAEEV 0.656 *T*

1Rice 335 DECNTHRRA 0.963 *T*

_________________________^_________________

Tyrosine predictions

Name Pos Context Score Pred

_________________________v_________________

1Rice 239 VNDAYKRMV 0.922 *Y*

1Rice 320 ESRDYVFAW 0.840 *Y*

_________________________^_________________


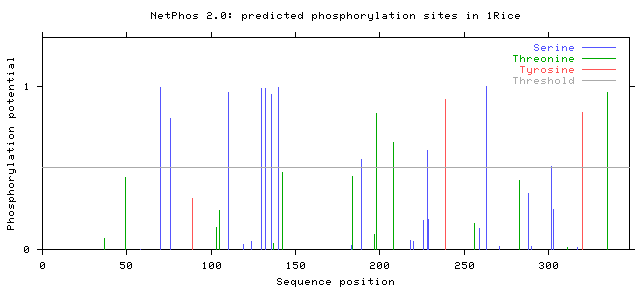


**AeSdr4L**

MAMVQPVDMAVKANEILARFRPIAPKPALPASPAQAQAIDGAADRVLCHLQSRPCRARKRGRPSAVPVSAPAAAAKRKRA 80

AYPVPLRCAAAAATDAVVSTATRAYVSVPGSACMPFASLPPATASTGGNLTMLSTMVAGDEEEEEEERDIPVERDLLRKL 160

LEPKVISPRAMRPVGSTIHVESIVHGAVDAASSTAASKTAEEVEAEVETDALPAIVTDSSNRVRLVNDAYKEMVGAPECL 240

WLGAVAASRRISGEVALVVAEQATLPESPGGFSCTAKIEWECGGGERASIHAACDVSRLQCEYRHYLFAWRFRAADASSP 320

ADSHRAGGEA 400

...............................................................S................ 80

........................Y.S..................................................... 160

......S........................S....S................................Y.......... 240

.......S...S...............S....................S.............................S. 320

..S....... 400

Phosphorylation sites predicted: Ser: 11 Thr: 0 Tyr: 2

Serine predictions

Name Pos Context Score Pred

_________________________v_________________

20Aegilops 64 RGRPSAVPV 0.868 *S*

20Aegilops 107 RAYVSVPGS 0.819 *S*

20Aegilops 167 PKVISPRAM 0.980 *S*

20Aegilops 192 VDAASSTAA 0.738 *S*

20Aegilops 197 STAASKTAE 0.978 *S*

20Aegilops 248 AVAASRRIS 0.547 *S*

20Aegilops 252 SRRISGEVA 0.997 *S*

20Aegilops 268 TLPESPGGF 0.970 *S*

20Aegilops 289 GERASIHAA 0.971 *S*

20Aegilops 319 ADASSPADS 0.996 *S*

20Aegilops 323 SPADSHRAG 0.993 *S*

_________________________^_________________

Threonine predictions

Name Pos Context Score Pred

_________________________v_________________

_________________________^_________________

Tyrosine predictions

Name Pos Context Score Pred

_________________________v_________________

20Aegilops 105 ATRAYVSVP 0.862 *Y*

20Aegilops 230 VNDAYKEMV 0.931 *Y*

_________________________^_________________


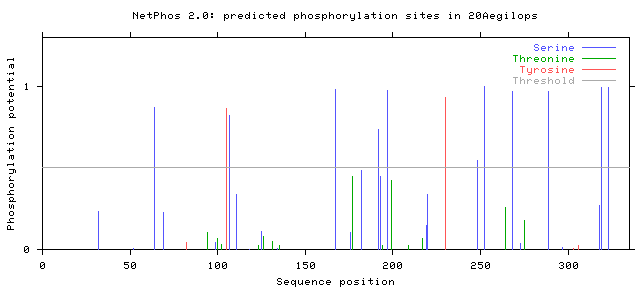


**HvSdr4L**

MAMVQPVDMAVKANQILARFRPIAPKPAALPASPAQAIDGAASRVLCHLQSRPCRARKRGRPSAVPVSAQPAAAKRKRAA 80

YPVPLRCAAAAATDAVVSTATRAHVSVPGSACMPFASLPPATASASGNLTRLSTMLVGVGDEEEEEEERDVPVERDLLRK 160

LLEPKVISPRAMRPVGSTIHVESIVHGAVDAASSTTASKTAEEVEAEVETDALPAVVTDSSNRVRLVNDAYKEMVGAPEC 240

LWLGAVAASRRISGEVALVVAEKAALPESPEGFSCTAKIEWECGTGERTSIQAACDVSRVHCESRHYLFAWRFHTADASS 320

PASNHRADGDA 400

..............................................................S................. 80

................................................................................ 160

.......S.........................S...S................................Y......... 240

........S...S...............S...............T....S........................T....S 320

..S........ 400

Phosphorylation sites predicted: Ser: 10 Thr: 2 Tyr: 1

Serine predictions

Name Pos Context Score Pred

_________________________v_________________

21Hordeuvul 63 RGRPSAVPV 0.868 *S*

21Hordeuvul 168 PKVISPRAM 0.980 *S*

21Hordeuvul 194 DAASSTTAS 0.868 *S*

21Hordeuvul 198 STTASKTAE 0.917 *S*

21Hordeuvul 249 AVAASRRIS 0.547 *S*

21Hordeuvul 253 SRRISGEVA 0.997 *S*

21Hordeuvul 269 ALPESPEGF 0.990 *S*

21Hordeuvul 290 GERTSIQAA 0.981 *S*

21Hordeuvul 320 ADASSPASN 0.974 *S*

21Hordeuvul 323 SSPASNHRA 0.988 *S*

_________________________^_________________

Threonine predictions

Name Pos Context Score Pred

_________________________v_________________

21Hordeuvul 285 WECGTGERT 0.531 *T*

21Hordeuvul 315 WRFHTADAS 0.516 *T*

_________________________^_________________

Tyrosine predictions

Name Pos Context Score Pred

_________________________v_________________

21Hordeuvul 231 VNDAYKEMV 0.931 *Y*

_________________________^_________________


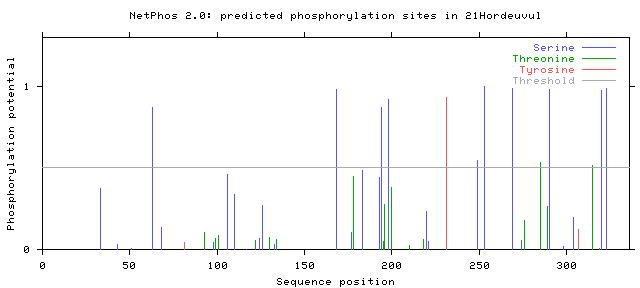


**AcSdr4L**

MMNSMNVYSSSSTSSKTDQILSRYRPIAPKPVVEGPPTPENTQQSPFSKYLLTRPSRNRKRGKSGCLPSKRARTTQSAAF 80

STPCGVASLAKNTQLGLSLQGYGHGFPSQLPIPSFDLSSSLEKPVNLVTLPFLPYPSSSVPEVPKTVPQMHGINMYSQLQ 160

DVPDLNSNSAIPQEMDLLLNLHPSNSATLNCGNGKVITPQAVRPVGSSITVECIQEDHNSNSASSVLKTPKDVEAEIELD 240

ALPAVVSDSRNRVRLANSAYMEMVGQPECLWLNSMANFDGQARSSACKRIGGKVMLDLADSQIPVFPDAFKCKARIEWGS 320

NEEKSSINVHCDVVKLSCESKNHLFTWRFHTKECKFNSQS 400

.......Y.S.STSS......S...............T......S..S.......S.......S.........TT..... 80

.T...................................S.S..................S..................... 160

...............................................S................S...T........... 240

......S.S..........Y........................S..................................S 320

....S...........S.............T......... 400

Phosphorylation sites predicted: Ser: 20 Thr: 7 Tyr: 2

Serine predictions

Name Pos Context Score Pred

_________________________v_________________

22Aquilegia 10 NVYSSSSTS 0.872 *S*

22Aquilegia 12 YSSSSTSSK 0.831 *S*

22Aquilegia 14 SSSTSSKTD 0.991 *S*

22Aquilegia 15 SSTSSKTDQ 0.997 *S*

22Aquilegia 22 DQILSRYRP 0.860 *S*

22Aquilegia 45 NTQQSPFSK 0.516 *S*

22Aquilegia 48 QSPFSKYLL 0.555 *S*

22Aquilegia 56 LTRPSRNRK 0.875 *S*

22Aquilegia 64 KRGKSGCLP 0.990 *S*

22Aquilegia 118 SFDLSSSLE 0.533 *S*

22Aquilegia 120 DLSSSLEKP 0.969 *S*

22Aquilegia 139 YPSSSVPEV 0.931 *S*

22Aquilegia 208 PVGSSITVE 0.540 *S*

22Aquilegia 225 NSASSVLKT 0.987 *S*

22Aquilegia 247 PAVVSDSRN 0.511 *S*

22Aquilegia 249 VVSDSRNRV 0.722 *S*

22Aquilegia 285 QARSSACKR 0.943 *S*

22Aquilegia 320 IEWGSNEEK 0.947 *S*

22Aquilegia 325 NEEKSSINV 0.778 *S*

22Aquilegia 337 VVKLSCESK 0.559 *S*

_________________________^_________________

Threonine predictions

Name Pos Context Score Pred

_________________________v_________________

22Aquilegia 13 SSSSTSSKT 0.519 *T*

22Aquilegia 38 EGPPTPENT 0.888 *T*

22Aquilegia 74 KRARTTQSA 0.583 *T*

22Aquilegia 75 RARTTQSAA 0.972 *T*

22Aquilegia 82 AAFSTPCGV 0.941 *T*

22Aquilegia 229 SVLKTPKDV 0.974 *T*

22Aquilegia 351 WRFHTKECK 0.537 *T*

_________________________^_________________

Tyrosine predictions

Name Pos Context Score Pred

_________________________v_________________

22Aquilegia 8 SMNVYSSSS 0.834 *Y*

22Aquilegia 260 ANSAYMEMV 0.802 *Y*

_________________________^_________________


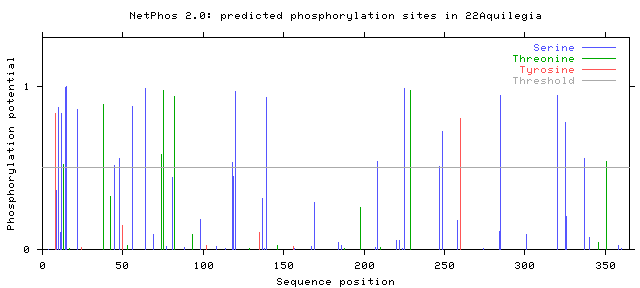


**StSdr4L**

MMQTLNPYPSTSKTAEIMARYRPIAPKPEAPTSPVSEDNPTGLPPNIQKSPFLRNVWPQLQARPTRTRKRGRTALGPPSM 80

KRARANYFPAGQFPTYQQVMAASPSYRPNVLPQFTLIPNLLPLKCGLGTSVTTPSNSITLPLMACTTTTLPMLVEKNSGE 160

EIRGIDLNLAADGPEELDFMPQLQGPKTPGPGVITPQPVRPVGSSISIGCINEEEAPEGGGTNKKFMKKPEEVEEEVEAE 240

ALPAVISDSNNKVRLTNAAYKEMVGQPECCWLDYMVGNACKRIGGEVILEFLDSSCSVPMSSDGFNCWVKIEWGAAQGKK 320

NSVKAFCNAVKLACQSKDYVFEWRFHTTDDDAPASAASNN 400

...T.....STS....................S..S..............................T...........S. 80

...............Y........S...........................T........................S.. 160

...........................T......T.........S................T.................. 240

...................Y.............Y.............................................. 320

.S........................TT............ 400

Phosphorylation sites predicted: Ser: 9 Thr: 9 Tyr: 3

Serine predictions

Name Pos Context Score Pred

_________________________v_________________

23Solnumtub 10 NPYPSTSKT 0.820 *S*

23Solnumtub 12 YPSTSKTAE 0.808 *S*

23Solnumtub 33 EAPTSPVSE 0.991 *S*

23Solnumtub 36 TSPVSEDNP 0.976 *S*

23Solnumtub 50 NIQKSPFLR 0.031 .

23Solnumtub 79 LGPPSMKRA 0.996 *S*

23Solnumtub 103 VMAASPSYR 0.186 .

23Solnumtub 105 AASPSYRPN 0.940 *S*

23Solnumtub 158 VEKNSGEEI 0.995 *S*

23Solnumtub 205 PVGSSISIG 0.512 *S*

23Solnumtub 322 GKKNSVKAF 0.909 *S*

_________________________^_________________

Threonine predictions

Name Pos Context Score Pred

_________________________v_________________

23Solnumtub 4 -MMQTLNPY 0.508 *T*

23Solnumtub 11 PYPSTSKTA 0.850 *T*

23Solnumtub 67 RPTRTRKRG 0.874 *T*

23Solnumtub 133 TSVTTPSNS 0.743 *T*

23Solnumtub 188 QGPKTPGPG 0.755 *T*

23Solnumtub 195 PGVITPQPV 0.857 *T*

23Solnumtub 222 EGGGTNKKF 0.813 *T*

23Solnumtub 347 WRFHTTDDD 0.952 *T*

23Solnumtub 348 RFHTTDDDA 0.919 *T*

_________________________^_________________

Tyrosine predictions

Name Pos Context Score Pred

_________________________v_________________

23Solnumtub 96 QFPTYQQVM 0.519 *Y*

23Solnumtub 260 TNAAYKEMV 0.548 *Y*

23Solnumtub 274 CWLDYMVGN 0.610 *Y*

_________________________^_________________


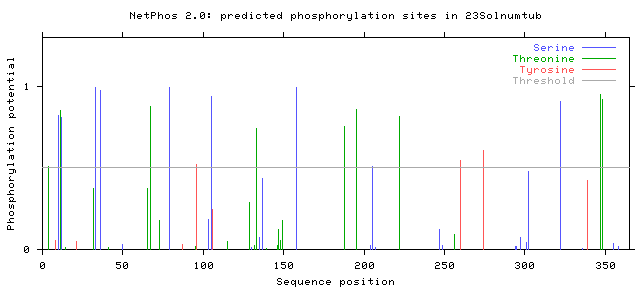


**SlSdr4L**

MMQTLNPYPSTSKTAEIMARYRPIAPKPEAPTSPVSEDNPTGLPPNIQKSPFLRNVWPQLQARPTRTRKRGRTALGPPSM 80

KRARGNYFPAGQFPNYHQVVAASPSYRPNVVPQFTLIPNLLPLKCGLGTSVTTPSNSITLPLMACTTTTLPMLVEKHSGE 160

EIRGIDLNLAADGPEELDFMPQLQGPKTPGPVVITPQPVRPVGSSISIGCINEEEAPDGGATNKKFIKKPEEVEEEVEAE 240

ALPAVVSDSNNKVRLTNAAYKEMVGQPECCWLDYMVGNACKRIGGEVILEFLDSSCSVPMSSDGFNCWVKIEWGAAQGKK 320

NSVKAFCNAVKLACQSKDYVFEWRFHTTDDNTPESAASNI 400

...T.....STS....................S..S..............................T...........S. 80

......................S.S...........................T........................S.. 160

...........................T......T.........S................T.................. 240

...................Y.............Y.............................................. 320

.S........................TT...T..S..... 400

Phosphorylation sites predicted: Ser: 11 Thr: 10 Tyr: 2

Serine predictions

Name Pos Context Score Pred

_________________________v_________________

24Solanumly 10 NPYPSTSKT 0.820 *S*

24Solanumly 12 YPSTSKTAE 0.808 *S*

24Solanumly 33 EAPTSPVSE 0.991 *S*

24Solanumly 36 TSPVSEDNP 0.976 *S*

24Solanumly 79 LGPPSMKRA 0.996 *S*

24Solanumly 103 VVAASPSYR 0.646 *S*

24Solanumly 105 AASPSYRPN 0.689 *S*

24Solanumly 158 VEKHSGEEI 0.997 *S*

24Solanumly 205 PVGSSISIG 0.512 *S*

24Solanumly 322 GKKNSVKAF 0.909 *S*

24Solanumly 355 NTPESAASN 0.556 *S*

_________________________^_________________

Threonine predictions

Name Pos Context Score Pred

_________________________v_________________

24Solanumly 4 -MMQTLNPY 0.508 *T*

24Solanumly 11 PYPSTSKTA 0.850 *T*

24Solanumly 67 RPTRTRKRG 0.874 *T*

24Solanumly 133 TSVTTPSNS 0.743 *T*

24Solanumly 188 QGPKTPGPV 0.900 *T*

24Solanumly 195 PVVITPQPV 0.644 *T*

24Solanumly 222 DGGATNKKF 0.802 *T*

24Solanumly 347 WRFHTTDDN 0.862 *T*

24Solanumly 348 RFHTTDDNT 0.825 *T*

24Solanumly 352 TDDNTPESA 0.531 *T*

_________________________^_________________

Tyrosine predictions

Name Pos Context Score Pred

_________________________v_________________

24Solanumly 260 TNAAYKEMV 0.548 *Y*

24Solanumly 274 CWLDYMVGN 0.610 *Y*

_________________________^_________________


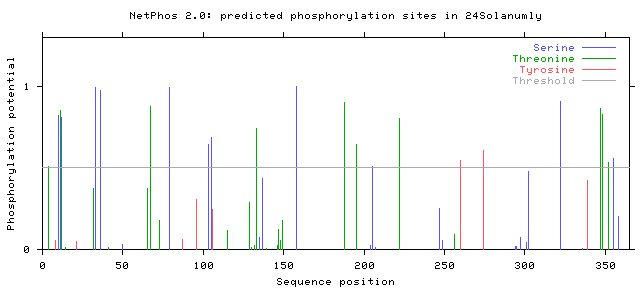


**VvSdr4L**

MIRTLNPYNSTAKTAEIMSRYRPIAPKPEPPVNPIPGFSPAALKRPRTHLVGFSTPSHVTSPAKNLSMHGFTHSPLQLPV 80

PAPSFAPLNGGFERAISTTADGLMTLPLLPCPPPQLPVEKDLLQQLQRAPANTTNVISPQPVRPIGSSISVRCMRDDLSP 160

IPEVQILKKPEEVEEEMESETLPAVISDSNNKIRMANSAYKEMVGQPECPWLNSMVTCIVLSTFVDALLTIIYEGRFCFL 240

VQMINNLPCELMFGGKWAIVFKRTICRRWDVQGHDYDTE 320

..................S...................S...............T.....S................... 80

................ST...................................................S........S. 160

.......................................Y........................................ 240

....................................... 320

Phosphorylation sites predicted: Ser: 6 Thr: 2 Tyr: 1

Serine predictions

Name Pos Context Score Pred

_________________________v_________________

25VitisVini 19 AEIMSRYRP 0.903 *S*

25VitisVini 39 IPGFSPAAL 0.565 *S*

25VitisVini 61 SHVTSPAKN 0.854 *S*

25VitisVini 97 ERAISTTAD 0.982 *S*

25VitisVini 150 GSSISVRCM 0.977 *S*

25VitisVini 159 RDDLSPIPE 0.986 *S*

_________________________^_________________

Threonine predictions

Name Pos Context Score Pred

_________________________v_________________

25VitisVini 55 VGFSTPSHV 0.817 *T*

25VitisVini 98 RAISTTADG 0.646 *T*

_________________________^_________________

Tyrosine predictions

Name Pos Context Score Pred

_________________________v_________________

25VitisVini 200 ANSAYKEMV 0.824 *Y*

_________________________^_________________


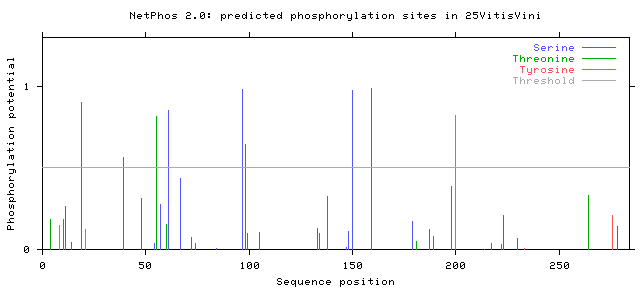


**PvSdr4L1** MAMVQPADPAVKANEILARFRPIAPKPALAAAASPVAQAAAEGVVAANRVLCQLQSRPCRARKRGRPTVVPVSPKSPAQP 80

AAKRKRAAAPYPPLRCAAATDAVATATRAHVSVVVPGSACLPLASLPPAATVAEDLVKVAAEERDVPVERDLLRKLLEPK 160

VISPRAVRPVCSTIHVECIRRTGATCTDAGPKTAAEVEAELEADALPAVVSDSGNRVRLVNDAYKEMVGQPECPWLDAVA 240

AASRRISGEVALQVDVAALLPEPHEVLTCTARIEWEYGGKYTSIMAPCDVSRLLCESRDYLFTWRFRTADADVSAGRRSG 320

DGEAIDS 400

...................................................................T....S..S.... 80

..................................................T............................. 160

..S.................................................S..........Y................ 240

..S...S.................................Y..........................T..........S. 320

....... 400

Phosphorylation sites predicted: Ser: 7 Thr: 3 Tyr: 2

Serine predictions

Name Pos Context Score Pred

_________________________v_________________

2Pavir.Ba01 73 VVPVSPKSP 0.998 *S*

2Pavir.Ba01 76 VSPKSPAQP 0.981 *S*

2Pavir.Ba01 163 PKVISPRAV 0.936 *S*

2Pavir.Ba01 213 VVSDSGNRV 0.723 *S*

2Pavir.Ba01 243 VAAASRRIS 0.543 *S*

2Pavir.Ba01 247 SRRISGEVA 0.997 *S*

2Pavir.Ba01 319 AGRRSGDGE 0.988 *S*

_________________________^_________________

Threonine predictions

Name Pos Context Score Pred

_________________________v_________________

2Pavir.Ba01 68 RGRPTVVPV 0.970 *T*

2Pavir.Ba01 131 PPAATVAED 0.882 *T*

2Pavir.Ba01 308 WRFRTADAD 0.760 *T*

_________________________^_________________

Tyrosine predictions

Name Pos Context Score Pred

_________________________v_________________

2Pavir.Ba01 281 YGGKYTSIM 0.824 *Y*

_________________________^_________________


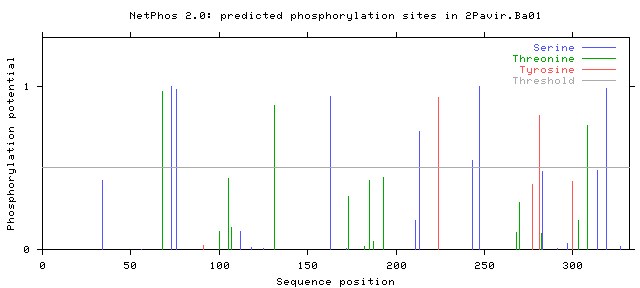


**PvSdr4L2**

MANVTGSRSGQATRMQTPRLPPSPHSILAASSHWNPPPPNPSAPSKARAPATRLRLRVGRVPARSPTWPPLFAMAMAQPA 80

DPAVKANEILARFRPIAPKPALAAAASPVTQAAAEGVVAANRVLCQLQSRPCRARKRGRPTVVPVSPKSPAQPAAKRKRA 160

AAPYPPLRCAAATDVVATAARAHVSVVVPDSACLPLASLPPAATVAEDLVKVAAEERDVPVERDLLRKLLEPKVVSPPAG 240

RPVCSTIHVECIRRTDATCTDAIPKTAAEVEAELEADALPAVVSDSGNRVRLVNDAYKEMVGQPECPWLDAVAAASRRIS 320

GEVVLLLAEAALLPEPHEVLTCTARIEWEYGGRHLHLGTSRDNLFTWRFRTTDADASVGRRSGDGEASDS 400

......S.S.......T.....S..S....SS............S...................S............... 80

............................................................T....S..S........... 160

...........................................T...............................S.... 240

.............................................S..........Y..................S...S 320

......................................T...........TT....S....S........ 400

Phosphorylation sites predicted: Ser: 16 Thr: 6 Tyr: 1

Serine predictions

Name Pos Context Score Pred

_________________________v_________________

3Pavir.Ba01 7 NVTGSRSGQ 0.745 *S*

3Pavir.Ba01 9 TGSRSGQAT 0.787 *S*

3Pavir.Ba01 23 RLPPSPHSI 0.946 *S*

3Pavir.Ba01 26 PSPHSILAA 0.727 *S*

3Pavir.Ba01 31 ILAASSHWN 0.771 *S*

3Pavir.Ba01 32 LAASSHWNP 0.660 *S*

3Pavir.Ba01 45 PSAPSKARA 0.800 *S*

3Pavir.Ba01 65 VPARSPTWP 0.995 *S*

3Pavir.Ba01 146 VVPVSPKSP 0.998 *S*

3Pavir.Ba01 149 VSPKSPAQP 0.981 *S*

3Pavir.Ba01 236 PKVVSPPAG 0.940 *S*

3Pavir.Ba01 286 VVSDSGNRV 0.723 *S*

3Pavir.Ba01 316 VAAASRRIS 0.543 *S*

3Pavir.Ba01 320 SRRISGEVV 0.996 *S*

3Pavir.Ba01 377 DADASVGRR 0.958 *S*

3Pavir.Ba01 382 VGRRSGDGE 0.993 *S*

_________________________^_________________

Threonine predictions

Name Pos Context Score Pred

_________________________v_________________

3Pavir.Ba01 17 TRMQTPRLP 0.993 *T*

3Pavir.Ba01 141 RGRPTVVPV 0.970 *T*

3Pavir.Ba01 204 PPAATVAED 0.882 *T*

3Pavir.Ba01 359 LHLGTSRDN 0.712 *T*

3Pavir.Ba01 371 WRFRTTDAD 0.887 *T*

3Pavir.Ba01 372 RFRTTDADA 0.937 *T*

_________________________^_________________

Tyrosine predictions

Name Pos Context Score Pred

_________________________v_________________

3Pavir.Ba01 297 VNDAYKEMV 0.931 *Y*

_________________________^_________________


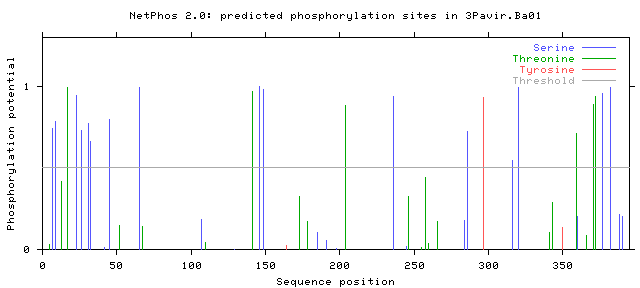


**PvSdr4L3** MQRKTIKEIARRAAILANETPSGAHATTPSMARAPASQPVPAARPAERSTGDEARAPARRLLPLEPTQPKRAPPPRLGSA 80

YVSAASPRGRPRSPFSLEWSSPLTTDLDPEPREPAAALLAMAMVQPADPAVKANEILARFRPIAPKPALAAAAASPVAQA 160

AAEGVVAANRVLCQLQSRPCRARKRGRPNVVPVSPKSPAQPAANRKRAAAPYPPLRCAATTDAVATATRAHVSVVVPGSA 240

CLPLASLPPADTVAEDLVKVAAEERDVPVERDLLRKLLEPKVISPRAVRPVFSTIHVECIRRTDATCTDAVSKTAAEVEA 320

ELEADALPAVVSDSGNRVRLVNDAYKEMVGQPECPWLDAVAAASRRISGEVVLQVAEAALLPEPHEVLTCTARIEWEYGG 400

RCTCIMVPCDVRRLLCESRDYLFTWRFRTADADVSVGCRSGDGEASDS 480

....T..............T.......T....................ST............................S. 80

Y....S......S..S....S.....................................................S..... 160

.................................S..S......................T.................... 240

...........T...............................S.........T.................S........ 320

.............S..........Y..................S...S................................ 400

............................T................... 480

Phosphorylation sites predicted: Ser: 14 Thr: 8 Tyr: 2

Serine predictions

Name Pos Context Score Pred

_________________________v_________________

4Pavir.Bb01 49 PAERSTGDE 0.994 *S*

4Pavir.Bb01 79 PRLGSAYVS 0.513 *S*

4Pavir.Bb01 86 VSAASPRGR 0.992 *S*

4Pavir.Bb01 93 GRPRSPFSL 0.996 *S*

4Pavir.Bb01 96 RSPFSLEWS 0.992 *S*

4Pavir.Bb01 101 LEWSSPLTT 0.677 *S*

4Pavir.Bb01 155 AAAASPVAQ 0.804 *S*

4Pavir.Bb01 194 VVPVSPKSP 0.994 *S*

4Pavir.Bb01 197 VSPKSPAQP 0.981 *S*

4Pavir.Bb01 284 PKVISPRAV 0.936 *S*

4Pavir.Bb01 312 TDAVSKTAA 0.780 *S*

4Pavir.Bb01 334 VVSDSGNRV 0.723 *S*

4Pavir.Bb01 364 VAAASRRIS 0.543 *S*

4Pavir.Bb01 368 SRRISGEVV 0.996 *S*

_________________________^_________________

Threonine predictions

Name Pos Context Score Pred

_________________________v_________________

4Pavir.Bb01 5 MQRKTIKEI 0.955 *T*

4Pavir.Bb01 20 LANETPSGA 0.560 *T*

4Pavir.Bb01 28 AHATTPSMA 0.844 *T*

4Pavir.Bb01 50 AERSTGDEA 0.965 *T*

4Pavir.Bb01 220 RCAATTDAV 0.511 *T*

4Pavir.Bb01 252 PPADTVAED 0.966 *T*

4Pavir.Bb01 294 PVFSTIHVE 0.500 *T*

4Pavir.Bb01 429 WRFRTADAD 0.760 *T*

_________________________^_________________

Tyrosine predictions

Name Pos Context Score Pred

_________________________v_________________

4Pavir.Bb01 81 LGSAYVSAA 0.717 *Y*

4Pavir.Bb01 345 VNDAYKEMV 0.931 *Y*

_________________________^_________________


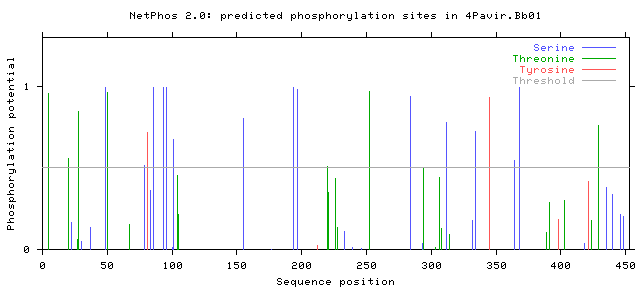


**PvSdr4L4**

MAHQKEVAAETSEAMERRFRPITPKPLPESPPPMPIGTTATNDGEVPVVMGAWCMPESFLPGCEEHLQGLSLEGSSISLW 80

ASSQDAGRLFPVERDLISKLQVPKVIRPRPARPVRIIICIDCSNIVVDATTSVLEVAMSNKTPREVEVELELPDALPAIV 160

AGYNNNRVYLANDAYKAMVGQPVCPWLDSLPGAGASRRINGEVVLSVGAFSTALPLSSTGCAFPGTARISWEREEASASL 240

TVPCAVERLTSNCNDYCYIWRFDSKKASIMYCIT 320

......................T......S.................................................. 80

.SS...............................................T.......S..T.................. 160

..............Y......................................................S.......... 240

..........S....Y.......S.......... 320

Phosphorylation sites predicted: Ser: 7 Thr: 3 Tyr: 2

Serine predictions

Name Pos Context Score Pred

_________________________v_________________

5Pavir.Ib02 30 PLPESPPPM 0.878 *S*

5Pavir.Ib02 82 SLWASSQDA 0.837 *S*

5Pavir.Ib02 83 LWASSQDAG 0.766 *S*

5Pavir.Ib02 139 EVAMSNKTP 0.906 *S*

5Pavir.Ib02 230 TARISWERE 0.998 *S*

5Pavir.Ib02 251 ERLTSNCND 0.769 *S*

5Pavir.Ib02 264 WRFDSKKAS 0.990 *S*

_________________________^_________________

Threonine predictions

Name Pos Context Score Pred

_________________________v_________________

5Pavir.Ib02 23 FRPITPKPL 0.988 *T*

5Pavir.Ib02 131 VDATTSVLE 0.646 *T*

5Pavir.Ib02 142 MSNKTPREV 0.987 *T*

_________________________^_________________

Tyrosine predictions

Name Pos Context Score Pred

_________________________v_________________

5Pavir.Ib02 175 ANDAYKAMV 0.931 *Y*

5Pavir.Ib02 256 NCNDYCYIW 0.680 *Y*

_________________________^_________________


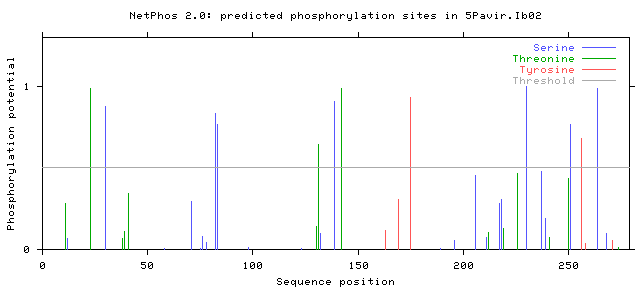


**PvSdr4L5**

MAFRAHQKEVAAETSEAMERRFRPIAPKPLPASPPSMPIGTTWLSYPPPPIRWATNDGEVPVVTRAWCMPESFLPGCEEH 80

LRGLSLEGSLISPWAPSPDTGRLFPVERDLISKLQVPKVIRPRPARPVRTIICIDCNNIVIDATTSAVEVAMSNKTPREV 160

EVELELPDALPAIVSGCNNNHVYLANDAYKAMVGQPICPWLDSLPGAGPSRWINGEVVLSVGDIGCAFPCTARISWERED 240

AKASLTVPCAVERLTSNCNGYCFIWRFDSKKASIMYCIT 320

................................S.....................T......................... 80

....S...........S..T............................................TS......S..T.... 160

......................Y.....Y..............................S..............S..... 240

...............S............S.......... 320

Phosphorylation sites predicted: Ser: 9 Thr: 4 Tyr: 2

Serine predictions

Name Pos Context Score Pred

_________________________v_________________

6Pavir.Ib02 33 PLPASPPSM 0.877 *S*

6Pavir.Ib02 85 LRGLSLEGS 0.958 *S*

6Pavir.Ib02 97 PWAPSPDTG 0.903 *S*

6Pavir.Ib02 146 DATTSAVEV 0.643 *S*

6Pavir.Ib02 153 EVAMSNKTP 0.933 *S*

6Pavir.Ib02 220 EVVLSVGDI 0.989 *S*

6Pavir.Ib02 235 TARISWERE 0.997 *S*

6Pavir.Ib02 256 ERLTSNCNG 0.829 *S*

6Pavir.Ib02 269 WRFDSKKAS 0.990 *S*

_________________________^_________________

Threonine predictions

Name Pos Context Score Pred

_________________________v_________________

6Pavir.Ib02 55 IRWATNDGE 0.777 *T*

6Pavir.Ib02 100 PSPDTGRLF 0.952 *T*

6Pavir.Ib02 145 IDATTSAVE 0.608 *T*

6Pavir.Ib02 156 MSNKTPREV 0.987 *T*

_________________________^_________________

Tyrosine predictions

Name Pos Context Score Pred

_________________________v_________________

6Pavir.Ib02 183 NNHVYLAND 0.546 *Y*

6Pavir.Ib02 189 ANDAYKAMV 0.931 *Y*

_________________________^_________________


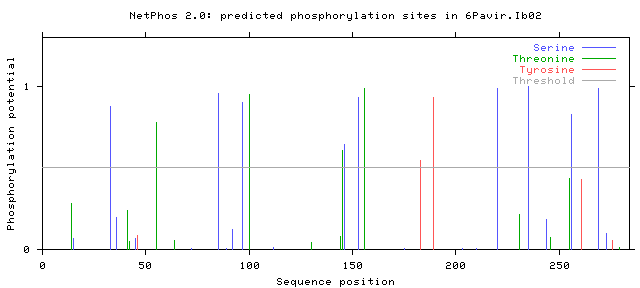


**PvSdr4L6**

MAHEKEVAAETSEAMERRFQPIAPKPLPAPPPPMPISTTVSSILLGAHKRNRQDYLVPSPVSKRERDALSYPPPPPVRWA 80

TNDGEVPVVMRAWCMPESFLPSCEEHFQGLSLEGVFRFTVPKVIRPRPARPVRTTIFIDCSNIVGATTSVVEVAVSNKTP 160

REVEVELELPDTLPAIVSGCNNHRVYLANDAYKAMVGQPICPWLDSLPGAGASRRINGEVVLSVGEFSNILHLPSTECAF 240

PCTARISWEREDASASLTVPCAVERLTSNCNDYCFIWRFDCEKASIMYCIA 320

.....................................T....................S..S.......S.......... 80

T....................S................T...............T............T.......S..T. 160

...............................Y..............................S...........S..... 240

......S......S.............S....Y.................. 320

Phosphorylation sites predicted: Ser: 10 Thr: 6 Tyr: 2

Serine predictions

Name Pos Context Score Pred

_________________________v_________________

7Pavir.Ia02 59 YLVPSPVSK 0.869 *S*

7Pavir.Ia02 62 PSPVSKRER 0.995 *S*

7Pavir.Ia02 70 RDALSYPPP 0.790 *S*

7Pavir.Ia02 102 SFLPSCEEH 0.990 *S*

7Pavir.Ia02 156 EVAVSNKTP 0.907 *S*

7Pavir.Ia02 223 EVVLSVGEF 0.974 *S*

7Pavir.Ia02 235 LHLPSTECA 0.966 *S*

7Pavir.Ia02 247 TARISWERE 0.997 *S*

7Pavir.Ia02 254 REDASASLT 0.726 *S*

7Pavir.Ia02 268 ERLTSNCND 0.769 *S*

_________________________^_________________

Threonine predictions

Name Pos Context Score Pred

_________________________v_________________

7Pavir.Ia02 38 MPISTTVSS 0.523 *T*

7Pavir.Ia02 81 VRWATNDGE 0.765 *T*

7Pavir.Ia02 119 VFRFTVPKV 0.627 *T*

7Pavir.Ia02 135 PVRTTIFID 0.878 *T*

7Pavir.Ia02 148 VGATTSVVE 0.957 *T*

7Pavir.Ia02 159 VSNKTPREV 0.990 *T*

_________________________^_________________

Tyrosine predictions

Name Pos Context Score Pred

_________________________v_________________

7Pavir.Ia02 192 ANDAYKAMV 0.931 *Y*

7Pavir.Ia02 273 NCNDYCFIW 0.656 *Y*

_________________________^_________________


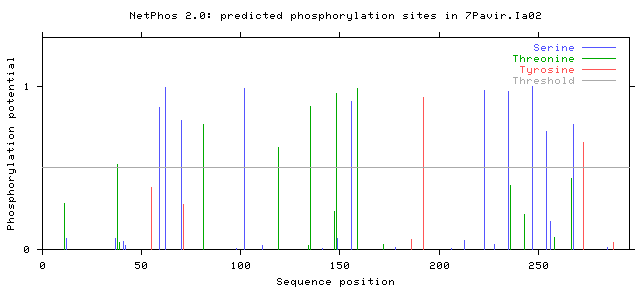


**PvSdr4L7** MAFMAHEKEVAAETSEAMERRFRPIAPKPLPAPPPPMPISTTVSSILLGAHKRNRQDYLVPSPVSKRERDALSYPPPPPV 80

RWATNDGEVPVVMRAWCMPESFLPSCEEHFQGLSLEGSSVSWRAPSPDAGRLFPVERDLISNLQVPKVIKPRPARPVRTT 160

IFIDCSNVIGATTSEVEVAVSNKTPREVEVELELPDALPAIVSGCNNHRVYLANDAYKAMVGQPICPWLDSLPGAGASRR 240

INGEVVLSVGEFSNILHLPSTECAFPCTARISWEREDASASLTVPCAVERLTSNCNDYCFIWRFDSEKASIMYCIT 320

........................................T....................S..S.......S....... 80

...T....................S.............S.S....S.................................T 160

............TS......S..T................................Y....................... 240

.......S...........S...........S......S.............S....Y.......S.......... 320

Phosphorylation sites predicted: Ser: 15 Thr: 5 Tyr: 2

Serine predictions

Name Pos Context Score Pred

_________________________v_________________

8Pavir.Ia03 62 YLVPSPVSK 0.869 *S*

8Pavir.Ia03 65 PSPVSKRER 0.995 *S*

8Pavir.Ia03 73 RDALSYPPP 0.790 *S*

8Pavir.Ia03 105 SFLPSCEEH 0.990 *S*

8Pavir.Ia03 119 LEGSSVSWR 0.612 *S*

8Pavir.Ia03 121 GSSVSWRAP 0.980 *S*

8Pavir.Ia03 126 WRAPSPDAG 0.995 *S*

8Pavir.Ia03 174 GATTSEVEV 0.534 *S*

8Pavir.Ia03 181 EVAVSNKTP 0.907 *S*

8Pavir.Ia03 248 EVVLSVGEF 0.974 *S*

8Pavir.Ia03 260 LHLPSTECA 0.966 *S*

8Pavir.Ia03 272 TARISWERE 0.997 *S*

8Pavir.Ia03 279 REDASASLT 0.726 *S*

8Pavir.Ia03 293 ERLTSNCND 0.769 *S*

8Pavir.Ia03 306 WRFDSEKAS 0.991 *S*

_________________________^_________________

Threonine predictions

Name Pos Context Score Pred

_________________________v_________________

8Pavir.Ia03 41 MPISTTVSS 0.523 *T*

8Pavir.Ia03 84 VRWATNDGE 0.765 *T*

8Pavir.Ia03 160 PVRTTIFID 0.878 *T*

8Pavir.Ia03 173 IGATTSEVE 0.942 *T*

8Pavir.Ia03 184 VSNKTPREV 0.990 *T*

_________________________^_________________

Tyrosine predictions

Name Pos Context Score Pred

_________________________v_________________

8Pavir.Ia03 217 ANDAYKAMV 0.931 *Y*

8Pavir.Ia03 298 NCNDYCFIW 0.656 *Y*

_________________________^_________________


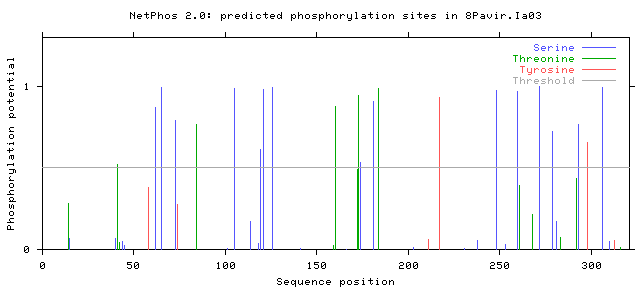


**SiSdr4L** MARTPASQCPRPSRRNAPRGTRRPRQRGTAKARGKPKRPSDPHAGPRRHLTRPDALLSSVLPLEPTQPKPPLQSARPATR 80

LRLRVGRVPARSPTVSLEWSSTLTTDPDPEPPLLAMAMVQPADPAVKANQILARFRPIAPKPALAAASPVAQAAAEGVVA 160

ANRVLCQLQNRPCRARKRGRPTVVPVSPKSPAQPAAKRKRAEAPYPPLRCAAATATRAHVSVVVPDSACLPLASLPPATT 240

VAGDLVKVAAEERDVPVERDLLRKLLEPKVISPRAVRPVCSTIYVERIHRTDATCTAVVSKTAAEVEVELEADALPAVVS 320

DSSNRVRLVNDAYKEMVGQSECPWLDAVAAASRRISGEVALVVGEPASLPEPHGVFTCTARIEWEYGGKCTSILAPCDVS 400

RLQCESRDYLFTWRFRTVDADASVGRRSGETSDS 480

............S.......T.......T..........S..........T............................. 80

...........S...S.......T...........................................S............ 160

.....................T....S..S..............................S..................T 240

...............................S.........T.Y...........T........................ 320

.S..........Y..................S...S......................T..................... 400

................T..........S..TS.. 480

Phosphorylation sites predicted: Ser: 14 Thr: 11 Tyr: 2

Serine predictions

Name Pos Context Score Pred

_________________________v_________________

9Si029889m 13 CPRPSRRNA 0.993 *S*

9Si029889m 40 PKRPSDPHA 0.973 *S*

9Si029889m 92 VPARSPTVS 0.989 *S*

9Si029889m 96 SPTVSLEWS 0.988 *S*

9Si029889m 148 LAAASPVAQ 0.796 *S*

9Si029889m 187 VVPVSPKSP 0.998 *S*

9Si029889m 190 VSPKSPAQP 0.981 *S*

9Si029889m 221 RAHVSVVVP 0.549 *S*

9Si029889m 272 PKVISPRAV 0.936 *S*

9Si029889m 322 VVSDSSNRV 0.606 *S*

9Si029889m 352 VAAASRRIS 0.543 *S*

9Si029889m 356 SRRISGEVA 0.997 *S*

9Si029889m 428 VGRRSGETS 0.997 *S*

9Si029889m 432 SGETSDS-- 0.868 *S*

_________________________^_________________

Threonine predictions

Name Pos Context Score Pred

_________________________v_________________

9Si029889m 21 APRGTRRPR 0.985 *T*

9Si029889m 29 RQRGTAKAR 0.929 *T*

9Si029889m 51 RRHLTRPDA 0.949 *T*

9Si029889m 104 SSTLTTDPD 0.840 *T*

9Si029889m 182 RGRPTVVPV 0.970 *T*

9Si029889m 240 PPATTVAGD 0.845 *T*

9Si029889m 282 PVCSTIYVE 0.560 *T*

9Si029889m 296 DATCTAVVS 0.515 *T*

9Si029889m 379 VFTCTARIE 0.577 *T*

9Si029889m 417 WRFRTVDAD 0.842 *T*

9Si029889m 431 RSGETSDS- 0.559 *T*

_________________________^_________________

Tyrosine predictions

Name Pos Context Score Pred

_________________________v_________________

9Si029889m 284 CSTIYVERI 0.766 *Y*

9Si029889m 333 VNDAYKEMV 0.931 *Y*

_________________________^_________________


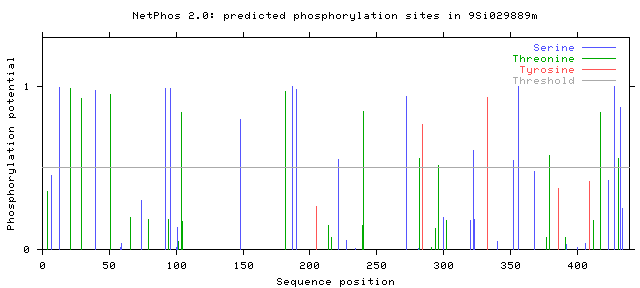


[**Explain**](http://www.cbs.dtu.dk/services/NetPhos-2.0/output.html) the output. Go [**back**](javascript:history.back()).

|  |  |  |
| --- | --- | --- |

**EgSdr4L** MIKTLSPYSTTPAATAKTAEIMSRYRPIAPKPETVPSAASSAAESPTSMSPKIRQSPYLRNLWPQLQARPTRTRKRGRTA 80

ISPPAIKRPKTAATAAGAPAGVPSPCALPPTKSLSLQVFTHGLPHIPLAGLMESPPVALAGSAAAASSPRSLVTLPLLPC 160

SPELNCMNTVRVEEAAAVDLNVNSAVETIPKEKDLLLQLQGPASGSGNVISPRPVRPVGSRISVGCISEDRRPDSPAPRA 240

RQKPEEVEEEVESEALPAVISDSKNRVRVVNSAYKEMVGQPECPWLEAMARLGAAGGRRIGGEVAIRMCDEEAKVPVWAE 320

GFSCWARIEWVGSGGEKSSVKAFCDAIRLRCDSKDYLFTWRFHTNAARPSSEFSSSAV 400

.....S..S.T...T.......S.................S...S..S.S.....S.Y..............T....... 80

.S.....................S..........................................SS..S......... 160

S.......T..............S...T...............S......S...........S...........S..... 240

.................................Y.............................................. 320

............S.....S.............S................SS....... 400

Phosphorylation sites predicted: Ser: 24 Thr: 5 Tyr: 2

Serine predictions

Name Pos Context Score Pred

_________________________v_________________

26Eucalyptu 6 IKTLSPYST 0.991 *S*

26Eucalyptu 9 LSPYSTTPA 0.942 *S*

26Eucalyptu 23 AEIMSRYRP 0.903 *S*

26Eucalyptu 41 SAASSAAES 0.983 *S*

26Eucalyptu 45 SAAESPTSM 0.987 *S*

26Eucalyptu 48 ESPTSMSPK 0.946 *S*

26Eucalyptu 50 PTSMSPKIR 0.916 *S*

26Eucalyptu 56 KIRQSPYLR 0.768 *S*

26Eucalyptu 82 RTAISPPAI 0.903 *S*

26Eucalyptu 104 AGVPSPCAL 0.729 *S*

26Eucalyptu 147 AAAASSPRS 0.561 *S*

26Eucalyptu 148 AAASSPRSL 0.987 *S*

26Eucalyptu 151 SSPRSLVTL 0.761 *S*

26Eucalyptu 161 LLPCSPELN 0.715 *S*

26Eucalyptu 184 LNVNSAVET 0.624 *S*

26Eucalyptu 204 QGPASGSGN 0.902 *S*

26Eucalyptu 211 GNVISPRPV 0.568 *S*

26Eucalyptu 223 GSRISVGCI 0.927 *S*

26Eucalyptu 235 RRPDSPAPR 0.991 *S*

26Eucalyptu 333 EWVGSGGEK 0.816 *S*

26Eucalyptu 339 GEKSSVKAF 0.979 *S*

26Eucalyptu 353 LRCDSKDYL 0.842 *S*

26Eucalyptu 370 AARPSSEFS 0.986 *S*

26Eucalyptu 371 ARPSSEFSS 0.980 *S*

_________________________^_________________

Threonine predictions

Name Pos Context Score Pred

_________________________v_________________

26Eucalyptu 11 PYSTTPAAT 0.834 *T*

26Eucalyptu 15 TPAATAKTA 0.537 *T*

26Eucalyptu 73 RPTRTRKRG 0.874 *T*

26Eucalyptu 169 NCMNTVRVE 0.917 *T*

26Eucalyptu 188 SAVETIPKE 0.567 *T*

_________________________^_________________

Tyrosine predictions

Name Pos Context Score Pred

_________________________v_________________

26Eucalyptu 58 RQSPYLRNL 0.551 *Y*

26Eucalyptu 274 VNSAYKEMV 0.678 *Y*

_________________________^_________________


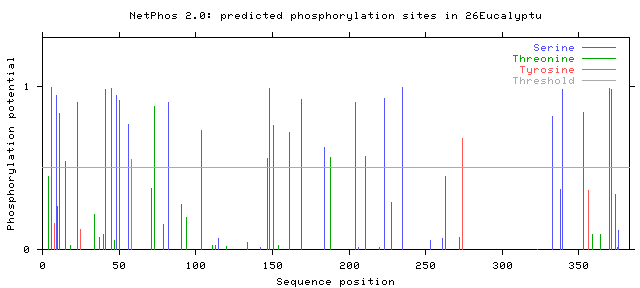


**PtSdr4L1** MIKTLSPCSNTAKTAEIMSRYRPIAPKPEGSTDESPSMPPFLRTLWPQMHARPTRTRKRGRAAVSPLTIKRPRTHLLGLS 80

SPSHATYSAKHLSLQGFAHGITQLPVPNLVGINCGMENSVTVSSNLVTLPLLQSPTVPVVENQAAAPELSCQEPNRDKVI 160

DLNTVAETSEERDPLQQLQEPSTSNVIAPQPVRPVCSSISVASINEDPSLIPPVKVPKKPEEIEEEVESEVLPIVITDSN 240

NKVRLANSAYKEMVGQPECSWLGSMMTSDGRFAGSSCKRICGEVVFHLSDLRVPESSNGFSCWVRIEWCNKVKSNVINTF 320

CDVIRLSCESKDYLFRWRFHIRTSKDSLSKTDA 400

.....S............S...........S...S.S................T.T........S..T............ 80

.......S........................................................................ 160

.......TS.................................S.....S............................... 240

.........Y................T........S....................S.....................T. 320

......S...............TS..S.S.... 400

Phosphorylation sites predicted: Ser: 16 Thr: 7 Tyr: 1

Serine predictions

Name Pos Context Score Pred

_________________________v_________________

27PopulusPo 6 IKTLSPCSN 0.965 *S*

27PopulusPo 19 AEIMSRYRP 0.903 *S*

27PopulusPo 31 KPEGSTDES 0.989 *S*

27PopulusPo 35 STDESPSMP 0.994 *S*

27PopulusPo 37 DESPSMPPF 0.753 *S*

27PopulusPo 65 RAAVSPLTI 0.683 *S*

27PopulusPo 88 HATYSAKHL 0.583 *S*

27PopulusPo 169 VAETSEERD 0.996 *S*

27PopulusPo 203 ISVASINED 0.969 *S*

27PopulusPo 209 NEDPSLIPP 0.932 *S*

27PopulusPo 276 FAGSSCKRI 0.959 *S*

27PopulusPo 297 VPESSNGFS 0.627 *S*

27PopulusPo 327 VIRLSCESK 0.993 *S*

27PopulusPo 344 HIRTSKDSL 0.972 *S*

27PopulusPo 347 TSKDSLSKT 0.996 *S*

27PopulusPo 349 KDSLSKTDA 0.997 *S*

_________________________^_________________

Threonine predictions

Name Pos Context Score Pred

_________________________v_________________

27PopulusPo 54 HARPTRTRK 0.697 *T*

27PopulusPo 56 RPTRTRKRG 0.874 *T*

27PopulusPo 68 VSPLTIKRP 0.894 *T*

27PopulusPo 168 TVAETSEER 0.641 *T*

27PopulusPo 267 GSMMTSDGR 0.624 *T*

27PopulusPo 319 NVINTFCDV 0.772 *T*

27PopulusPo 343 FHIRTSKDS 0.889 *T*

_________________________^_________________

Tyrosine predictions

Name Pos Context Score Pred

_________________________v_________________

27PopulusPo 250 ANSAYKEMV 0.824 *Y*

_________________________^_________________


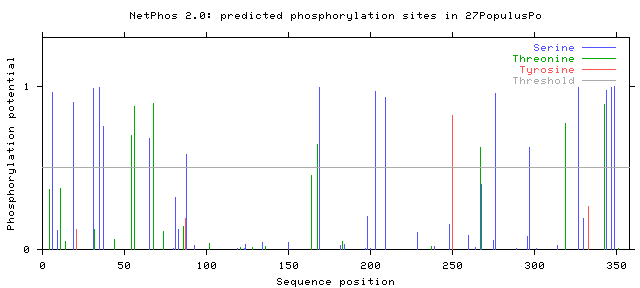


**PtSdr4L2**

MIKTLSPCSNTAKTAEIMSRYRPIAPRPEGSASSMDESSSMSQKIRESPYLRTLWPQMQARPTRTRKRGRAVVSPPNIKR 80

PRTHLLGLSSPSHVTSPAKHLSLQGFVHGIPQLPVPNLVGVNSGLENSVTMSSNLVTLPLLQSPTTVTVVANQAAVPELS 160

CMEPNRDKVIDLNTVAEFPEEKDLLQQLQVPPTNNVIAPQPLRLVGSSISIACISEDPSFIPLVRVPKKPEEVEEEVESE 240

VLPTVISDSNNKVRLANSAYKEMVGQPECSWLDSMMTGDGSFAGRSCKRICGEVELHLSDLRVPASSNGFSCWVRIEWCN 320

KGTKNVIITFCDVIRLSCASRDYLFSWRFHTRGRKDFQSKTNA 400

.....S............S.............SS...SSS.S.....S.Y..............T........S...... 80

...........S...S................................................................ 160

..........................................................S..................... 240

...................Y.........S......T........S....................S............. 320

................S........S............S.... 400

Phosphorylation sites predicted: Ser: 19 Thr: 2 Tyr: 2

Serine predictions

Name Pos Context Score Pred

_________________________v_________________

28PopulusPo 6 IKTLSPCSN 0.965 *S*

28PopulusPo 19 AEIMSRYRP 0.903 *S*

28PopulusPo 33 EGSASSMDE 0.991 *S*

28PopulusPo 34 GSASSMDES 0.991 *S*

28PopulusPo 38 SMDESSSMS 0.746 *S*

28PopulusPo 39 MDESSSMSQ 0.836 *S*

28PopulusPo 40 DESSSMSQK 0.873 *S*

28PopulusPo 42 SSSMSQKIR 0.988 *S*

28PopulusPo 48 KIRESPYLR 0.955 *S*

28PopulusPo 74 RAVVSPPNI 0.899 *S*

28PopulusPo 92 LSSPSHVTS 0.747 *S*

28PopulusPo 96 SHVTSPAKH 0.902 *S*

28PopulusPo 219 SEDPSFIPL 0.849 *S*

28PopulusPo 270 QPECSWLDS 0.940 *S*

28PopulusPo 286 FAGRSCKRI 0.928 *S*

28PopulusPo 307 VPASSNGFS 0.870 *S*

28PopulusPo 337 VIRLSCASR 0.722 *S*

28PopulusPo 346 DYLFSWRFH 0.688 *S*

28PopulusPo 359 KDFQSKTNA 0.776 *S*

_________________________^_________________

Threonine predictions

Name Pos Context Score Pred

_________________________v_________________

28PopulusPo 65 RPTRTRKRG 0.874 *T*

28PopulusPo 277 DSMMTGDGS 0.642 *T*

Tyrosine predictions

Name Pos Context Score Pred

_________________________v_________________

28PopulusPo 50 RESPYLRTL 0.927 *Y*

28PopulusPo 260 ANSAYKEMV 0.824 *Y*

_________________________^_________________


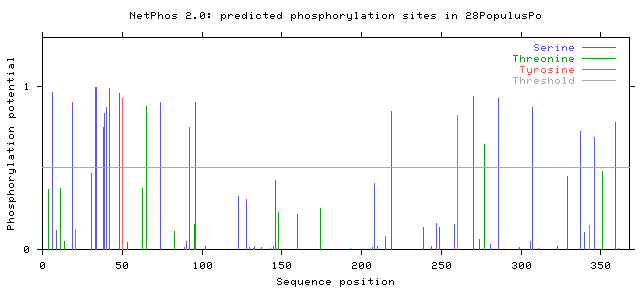


**LuSdr4L2** MIKTLTPYSNTAKTAEIMSRYRPIAPKPDCPSNSEDESSSMSQKIIQSPYLRNLWPQLQARPTRTRKRGRGALSPPVVKR 80

PRTHQMFSISPPAEHLGMQGFYPTGFSQLPTVQNPGAIQSNSVIASPSSLVTLPLLSPSAAFQGMQPHELMRCMAPSQER 160

DIDLNTVVEIPEEKDLLRQLQERHTTTNVIAPQPVRPVVSTIRVGCINETPGVPPVQYQKMPEQVEEEVESDVLPAVISD 240

SNNKVRLANSAYKEMVGQPECSWLNLMVTGNDRLGFGSSCCQRISGEVALHLSDSKVPVSSNGFSCWVRIEWGSQGKKSS 320

VNAFCDVIRLSCKSKDYLFTWRFHTPARQGSRISSNV 400

.....T.Y..........S............S.S...SSS.S......................T........S...... 80

.........................................S...S..S.......S...................S... 160

.........................T..............T................Y...................... 240

...........Y................................S..............S..................S. 320

..........S.............T.....S...... 400

Phosphorylation sites predicted: Ser: 18 Thr: 5 Tyr: 3

Serine predictions

Name Pos Context Score Pred

_________________________v_________________

29LinumLus1 9 LTPYSNTAK 0.128 .

29LinumLus1 19 AEIMSRYRP 0.903 *S*

29LinumLus1 32 PDCPSNSED 0.945 *S*

29LinumLus1 34 CPSNSEDES 0.977 *S*

29LinumLus1 38 SEDESSSMS 0.800 *S*

29LinumLus1 39 EDESSSMSQ 0.935 *S*

29LinumLus1 40 DESSSMSQK 0.785 *S*

29LinumLus1 42 SSSMSQKII 0.965 *S*

29LinumLus1 74 RGALSPPVV 0.629 *S*

29LinumLus1 122 IQSNSVIAS 0.631 *S*

29LinumLus1 126 SVIASPSSL 0.758 *S*

29LinumLus1 129 ASPSSLVTL 0.573 *S*

29LinumLus1 137 LPLLSPSAA 0.974 *S*

29LinumLus1 157 CMAPSQERD 0.983 *S*

29LinumLus1 285 CQRISGEVA 0.840 *S*

29LinumLus1 300 KVPVSSNGF 0.690 *S*

29LinumLus1 319 QGKKSSVNA 0.979 *S*

29LinumLus1 331 VIRLSCKSK 0.995 *S*

29LinumLus1 351 ARQGSRISS 0.730 *S*

Threonine predictions

Name Pos Context Score Pred

_________________________v_________________

29LinumLus1 6 IKTLTPYSN 0.809 *T*

29LinumLus1 65 RPTRTRKRG 0.874 *T*

29LinumLus1 186 ERHTTTNVI 0.749 *T*

29LinumLus1 201 PVVSTIRVG 0.916 *T*

29LinumLus1 345 WRFHTPARQ 0.723 *T*

_________________________^_________________

Tyrosine predictions

Name Pos Context Score Pred

_________________________v_________________

29LinumLus1 8 TLTPYSNTA 0.632 *Y*

29LinumLus1 218 PPVQYQKMP 0.540 *Y*

29LinumLus1 252 ANSAYKEMV 0.824 *Y*

________________________^_________________


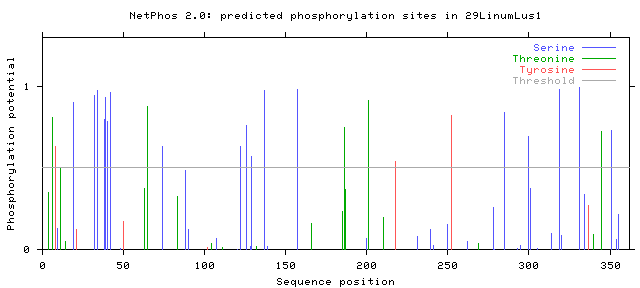


**LuSdr4L1**

MIKTLTPYSNTAKTAEIMSRYRPIAPKPDCPSNSEDENSSMSQKIRQSPYLRNLWPQLQARPTRTRKRGRGALSPPVVKR 80

LRTHQMFSISPPAEHLGMQGFYPTGFSQLPTVQNPGAIQSNSVIASPSSLVTLPLLSPSAAFQGMQPHELMRCMAPSQER 160

DIDLNTVVEIPEEKDLLRQLQERHATTTNVVIAPQPVRPVVSTIRVGCINETPGVPPVQYQKKPEQVEEEVESDVLPAVI 240

SDSNNKVRLANSAYKEMVGQPECSWLNLMVTGNDRLGFGSSCCQRISGEVALHLSDSKVPVSSNGFSCWVSIEWGSQGKK 320

SSVNAFCDVIRLSCKSKDYLFTWRFHTPARQGSQISSNV 400

.....T.Y..........S............S.S....SS.S.......Y..............T........S...... 80

.........................................S...S..S.......S...................S... 160

.........................TT...............T..................................... 240

.............Y................................S..............S........S......... 320

S...........S.............T.....S...... 400

Phosphorylation sites predicted: Ser: 18 Thr: 6 Tyr: 3

Serine predictions

Name Pos Context Score Pred

_________________________v_________________

30LinumLus1 19 AEIMSRYRP 0.903 *S*

30LinumLus1 32 PDCPSNSED 0.945 *S*

30LinumLus1 34 CPSNSEDEN 0.968 *S*

30LinumLus1 39 EDENSSMSQ 0.872 *S*

30LinumLus1 40 DENSSMSQK 0.595 *S*

30LinumLus1 42 NSSMSQKIR 0.929 *S*

30LinumLus1 74 RGALSPPVV 0.629 *S*

30LinumLus1 122 IQSNSVIAS 0.631 *S*

30LinumLus1 126 SVIASPSSL 0.758 *S*

30LinumLus1 129 ASPSSLVTL 0.573 *S*

30LinumLus1 137 LPLLSPSAA 0.974 *S*

30LinumLus1 157 CMAPSQERD 0.983 *S*

30LinumLus1 287 CQRISGEVA 0.840 *S*

30LinumLus1 302 KVPVSSNGF 0.690 *S*

30LinumLus1 311 SCWVSIEWG 0.689 *S*

30LinumLus1 321 QGKKSSVNA 0.979 *S*

30LinumLus1 333 VIRLSCKSK 0.995 *S*

30LinumLus1 353 ARQGSQISS 0.762 *S*

_________________________^_________________

Threonine predictions

Name Pos Context Score Pred

_________________________v_________________

30LinumLus1 6 IKTLTPYSN 0.809 *T*

30LinumLus1 65 RPTRTRKRG 0.874 *T*

30LinumLus1 186 ERHATTTNV 0.751 *T*

30LinumLus1 187 RHATTTNVV 0.747 *T*

30LinumLus1 203 PVVSTIRVG 0.916 *T*

30LinumLus1 347 WRFHTPARQ 0.723 *T*

_________________________^_________________

Tyrosine predictions

Name Pos Context Score Pred

_________________________v_________________

30LinumLus1 8 TLTPYSNTA 0.632 *Y*

30LinumLus1 50 RQSPYLRNL 0.551 *Y*

30LinumLus1 254 ANSAYKEMV 0.824 *Y*

_________________________^_________________


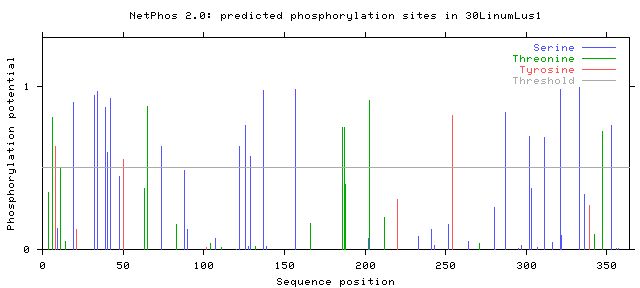


**MeSdr4L**

MIKTLNPYSTAKTAEIMSRYRPIAPKPELPSNSLGDTPSMSQKIRQSPYLRNLWPQLQARPTRTRKRGRATMSPPTIKRP 80

RTHLLGFSSPNHVLSPARHLSLQGFVHGVSQLPISNLAGVSSRSENPVTAASDLVTLPLLPCQPSVPAVAPEISCMEPRK 160

EVVIDLNTTVAEIPEEKDLLQQLQETLTTNVIAPLPIRPVGSTISVGCISEDPSSIPPLQLPKKPEEVEQEVECESLPAV 240

ISDSKYRVRLANSAYKEMVGQPECSWLDLMVTGDGRIGGSSCKRICGEVALHLPDSKVPTSSNGFSCWVRIEWGEEGKKK 320

SINSFCDVIRLSCESKDYLFTWRFHTHNREGSQSSTTA 400

.........T.......S..............S...T...S.......Y..............T......T.S..T.... 80

..............S..........................S...................................... 160

.....................................................S.......................... 240

...S.Y........Y.........S...............S..............S....S................... 320

...S.......S.....Y.......T.....S.S.... 400

Phosphorylation sites predicted: Ser: 16 Thr: 6 Tyr: 4

Serine predictions

Name Pos Context Score Pred

_________________________v_________________

31Manihot 18 AEIMSRYRP 0.903 *S*

31Manihot 33 LPSNSLGDT 0.993 *S*

31Manihot 41 TPSMSQKIR 0.739 *S*

31Manihot 73 RATMSPPTI 0.536 *S*

31Manihot 95 NHVLSPARH 0.964 *S*

31Manihot 122 AGVSSRSEN 0.946 *S*

31Manihot 214 SEDPSSIPP 0.911 *S*

31Manihot 244 VISDSKYRV 0.892 *S*

31Manihot 265 QPECSWLDL 0.851 *S*

31Manihot 281 IGGSSCKRI 0.988 *S*

31Manihot 296 HLPDSKVPT 0.577 *S*

31Manihot 301 KVPTSSNGF 0.824 *S*

31Manihot 324 KSINSFCDV 0.931 *S*

31Manihot 332 VIRLSCESK 0.993 *S*

31Manihot 352 NREGSQSST 0.974 *S*

31Manihot 354 EGSQSSTTA 0.559 *S*

_________________________^_________________

Threonine predictions

Name Pos Context Score Pred

_________________________v_________________

31Manihot 10 NPYSTAKTA 0.723 *T*

31Manihot 37 SLGDTPSMS 0.611 *T*

31Manihot 64 RPTRTRKRG 0.874 *T*

31Manihot 71 RGRATMSPP 0.851 *T*

31Manihot 76 MSPPTIKRP 0.658 *T*

31Manihot 346 WRFHTHNRE 0.544 *T*

Tyrosine predictions

Name Pos Context Score Pred

_________________________v_________________

31Manihot 49 RQSPYLRNL 0.551 *Y*

31Manihot 246 SDSKYRVRL 0.607 *Y*

31Manihot 255 ANSAYKEMV 0.824 *Y*

31Manihot 338 ESKDYLFTW 0.530 *Y*

_________________________^_________________


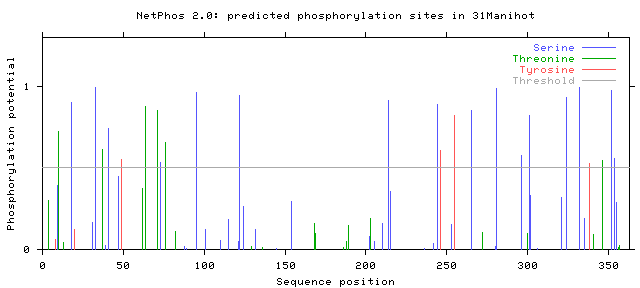


**RcSdr4L**

MIKTLSPYSTTAKTAEIMSRYRPIAPKPEGPSNSFGESSSMSQKISQSPYLRNLWPQLQARPTRTRKRGRAAISPPTIKR 80

PRTHVLGLSSTSHVISPARHLSLQGFAHGLSQLSVPSLVGVSSSLNNPVTTNSDLVTLSLLPCQPPVPVVDNQVTAPEIS 160

CMEARRQVIIDLNTVAEIPEEKDFLQQLQEPPTNNVIAPQPIRPVGSSISVGCVSEDTNSTPQVQVPKKPEEVEEEIECE 240

ALPTVICDSNYKVRLANSAYKEMVGQPECPWLDSMVAGDGRFGGNSCKRICGEVILHLADSRVPSSSNKFSCWVNIEWGH 320

EGKKTAATAFCDVIRLSCESKDYLFAWRFHTHSREGSQSSTKA 400

.....S..STT.......S............S.S....S..S...S...Y..............T........S..T... 80

...........S...S..................................T............................S 160

...............................................S................................ 240

...................Y.........................S..................S.S............. 320

................S.............T.S...S.SS... 400

Phosphorylation sites predicted: Ser: 21 Thr: 6 Tyr: 2

Serine predictions

Name Pos Context Score Pred

_________________________v_________________

32Ricinusco 6 IKTLSPYST 0.991 *S*

32Ricinusco 9 LSPYSTTAK 0.854 *S*

32Ricinusco 19 AEIMSRYRP 0.903 *S*

32Ricinusco 32 PEGPSNSFG 0.728 *S*

32Ricinusco 34 GPSNSFGES 0.897 *S*

32Ricinusco 39 FGESSSMSQ 0.508 *S*

32Ricinusco 42 SSSMSQKIS 0.985 *S*

32Ricinusco 46 SQKISQSPY 0.597 *S*

32Ricinusco 74 RAAISPPTI 0.675 *S*

32Ricinusco 92 LSSTSHVIS 0.658 *S*

32Ricinusco 96 SHVISPARH 0.985 *S*

32Ricinusco 160 APEISCMEA 0.946 *S*

32Ricinusco 208 PVGSSISVG 0.758 *S*

32Ricinusco 286 FGGNSCKRI 0.939 *S*

32Ricinusco 305 SRVPSSSNK 0.989 *S*

32Ricinusco 307 VPSSSNKFS 0.989 *S*

32Ricinusco 337 VIRLSCESK 0.993 *S*

32Ricinusco 353 FHTHSREGS 0.981 *S*

32Ricinusco 357 SREGSQSST 0.986 *S*

32Ricinusco 359 EGSQSSTKA 0.815 *S*

32Ricinusco 360 GSQSSTKA- 0.949 *S*

_________________________^_________________

Threonine predictions

Name Pos Context Score Pred

_________________________v_________________

32Ricinusco 10 SPYSTTAKT 0.659 *T*

32Ricinusco 11 PYSTTAKTA 0.623 *T*

32Ricinusco 65 RPTRTRKRG 0.874 *T*

32Ricinusco 77 ISPPTIKRP 0.786 *T*

32Ricinusco 131 NPVTTNSDL 0.696 *T*

32Ricinusco 351 WRFHTHSRE 0.698 *T*

_________________________^_________________

Tyrosine predictions

Name Pos Context Score Pred

_________________________v_________________

32Ricinusco 50 SQSPYLRNL 0.784 *Y*

32Ricinusco 260 ANSAYKEMV 0.824 *Y*

_________________________^_________________


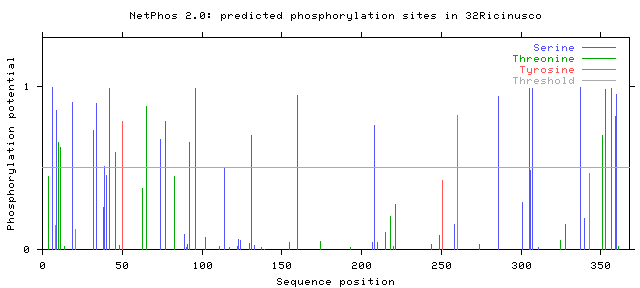


**CpSdr4L**

MIKTLSPYSTTAKTAEIMSRYRPIAPKPEVPMDPISDGAGSSMSQKIRQSPYLRNLWPQLQARPTRTRKRGRAPLSPPSL 80

KRARTHHVLGLSVPSHVTSPAKALSSQAFTHLPAPNLAPVTCNLENPATQSPSLVTLPLLPCSPSVPVILNQATTGQPSC 160

METIDLNKVAEIPEEKDLLQQLQTPPTTISNVMKPQPIRPVGSRISVGCINEDPNLFSTVQIPRKAEEVEDEMESEALPA 240

VISDSNNKVRLANSAYRKMVGQPECSWLEAMVTSGERGSSSCKRICGEVGLHFCDSRVPVSSNGFSCWVRIDWGSDGKNN 320

SIHAFCDVVRLSCLSKDYLFTWRFHTREAARFRSNV 400

.....S..STT.......S................S....S..........Y..............T........S..S. 80

....T.............S...........................................................S. 160

.............................................S.................................. 240

.........................S......TS....SSS....................S.................. 320

...........S.............T.......... 400

Phosphorylation sites predicted: Ser: 17 Thr: 6 Tyr: 1

Serine predictions

Name Pos Context Score Pred

_________________________v_________________

33Caricapap 6 IKTLSPYST 0.991 *S*

33Caricapap 9 LSPYSTTAK 0.854 *S*

33Caricapap 19 AEIMSRYRP 0.903 *S*

33Caricapap 36 MDPISDGAG 0.698 *S*

33Caricapap 41 DGAGSSMSQ 0.898 *S*

33Caricapap 76 RAPLSPPSL 0.962 *S*

33Caricapap 79 LSPPSLKRA 0.996 *S*

33Caricapap 99 SHVTSPAKA 0.864 *S*

33Caricapap 159 TGQPSCMET 0.990 *S*

33Caricapap 206 GSRISVGCI 0.563 *S*

33Caricapap 266 QPECSWLEA 0.698 *S*

33Caricapap 274 AMVTSGERG 0.950 *S*

33Caricapap 279 GERGSSSCK 0.982 *S*

33Caricapap 280 ERGSSSCKR 0.901 *S*

33Caricapap 281 RGSSSCKRI 0.988 *S*

33Caricapap 302 VPVSSNGFS 0.563 *S*

33Caricapap 332 VVRLSCLSK 0.907 *S*

_________________________^_________________

Threonine predictions

Name Pos Context Score Pred

_________________________v_________________

33Caricapap 10 SPYSTTAKT 0.659 *T*

33Caricapap 11 PYSTTAKTA 0.623 *T*

33Caricapap 67 RPTRTRKRG 0.874 *T*

33Caricapap 85 KRARTHHVL 0.762 *T*

33Caricapap 273 EAMVTSGER 0.676 *T*

33Caricapap 346 WRFHTREAA 0.542 *T*

_________________________^_________________

Tyrosine predictions

Name Pos Context Score Pred

_________________________v_________________

33Caricapap 52 RQSPYLRNL 0.551 *Y*

_________________________^_________________


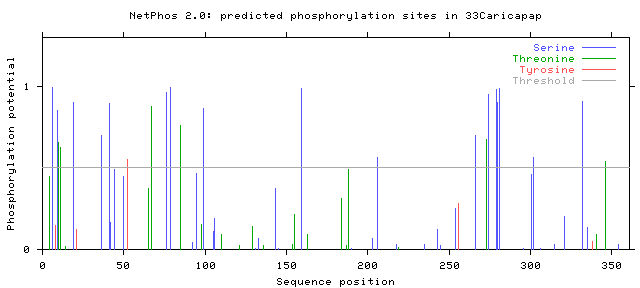


**GrSdr4L1**

MIKTLSPYSTTATTAEIMSRYRPIAPKPDVPANSMNESSGMSQKMMQSPYLRSLWCHLQARPTRARKRGRVALSSTTPLK 80

RGRTQVLALSSPSLITSPAKNLSLQGFSHGIPHHSIPNFGGSLDSSSTPPASLMQLPLLPCPPSVPVVANYATILELNCM 160

EPCGGEKLIDLNTIVEIPEEKDLLKQLQGPPASNVIVPQPIRPVGSIIYVGCIKENPILTPQMQVLKKREEVEELVESDA 240

LPAVISDSNNKVRLANSAYKEMVGQPECPWLDSVVTGEGRALGNSCKRICGEVVLHLSESDSRLTVKSKGFSCWARIEWG 320

SEGKKRSVKAFCEVIKLSCRSKDYLFTWKFQPYQQEEGRITF 400

.....S..S.........S..............S....S..S................................S..... 80

...T........................................SSS....S............................ 160

...........................................................T.................... 240

..................Y.........................S............S.S....T..S............ 320

......S..........S........................ 400

Phosphorylation sites predicted: Ser: 17 Thr: 3 Tyr: 1

Serine predictions

Name Pos Context Score Pred

_________________________v_________________

34Gossypium 6 IKTLSPYST 0.991 *S*

34Gossypium 9 LSPYSTTAT 0.982 *S*

34Gossypium 19 AEIMSRYRP 0.903 *S*

34Gossypium 34 VPANSMNES 0.961 *S*

34Gossypium 39 MNESSGMSQ 0.582 *S*

34Gossypium 42 SSGMSQKMM 0.985 *S*

34Gossypium 75 VALSSTTPL 0.754 *S*

34Gossypium 125 GSLDSSSTP 0.508 *S*

34Gossypium 126 SLDSSSTPP 0.718 *S*

34Gossypium 127 LDSSSTPPA 0.532 *S*

34Gossypium 132 TPPASLMQL 0.612 *S*

34Gossypium 285 ALGNSCKRI 0.944 *S*

34Gossypium 298 VLHLSESDS 0.986 *S*

34Gossypium 300 HLSESDSRL 0.631 *S*

34Gossypium 308 LTVKSKGFS 0.860 *S*

34Gossypium 327 GKKRSVKAF 0.984 *S*

34Gossypium 338 VIKLSCRSK 0.733 *S*

_________________________^_________________

Threonine predictions

Name Pos Context Score Pred

_________________________v_________________

34Gossypium 84 KRGRTQVLA 0.707 *T*

34Gossypium 220 NPILTPQMQ 0.519 *T*

34Gossypium 305 DSRLTVKSK 0.982 *T*

_________________________^_________________

Tyrosine predictions

Name Pos Context Score Pred

_________________________v_________________

34Gossypium 259 ANSAYKEMV 0.824 *Y*

_________________________^_________________


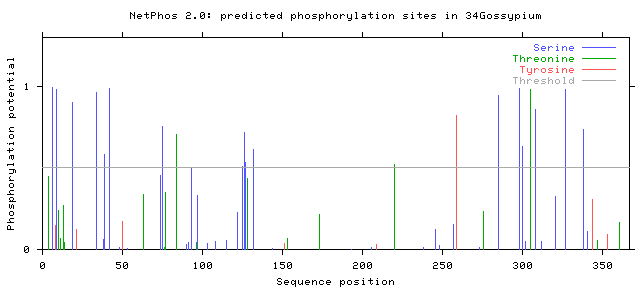


**GrSdr4L2**

MIKTLNPYSAKTAEIMSRYRPIAPKPEVLPENSIDESSAMSQKMRQSPYLRNLWPQLQARPSRNRKRGRGTGLSPPPPTT 80

TAMKRARTQYFLGLSPPPPPPPPPPSSTTSLVSLPLLPCLKVAAHEIPEEKDFLKQLQGLPVLPTSSLITPQPIRPVGST 160

IIVGCINEAPAPAAPLQAPKKPEEVEDDIESESMPTIISDSNNKVRLANSAYKAMVGQPECPWLDSMVKGSECKRICGEV 240

MLNLSNSRVPVKSKGFSCWVRIEWGNEGNNSNNKGSITAFCDVVRLSCQSKDYLFTWRFHIPTIGKTS 320

........S.......S...............S....S..S.......Y............S........T..S.....T 80

T......T..................S..S.................................................. 160

..............................S....................Y............................ 240

............S.................................S..................... 320

Phosphorylation sites predicted: Ser: 12 Thr: 4 Tyr: 2

Serine predictions

Name Pos Context Score Pred

_________________________v_________________

35Gossypium 9 LNPYSAKTA 0.959 *S*

35Gossypium 17 AEIMSRYRP 0.903 *S*

35Gossypium 33 LPENSIDES 0.653 *S*

35Gossypium 38 IDESSAMSQ 0.780 *S*

35Gossypium 41 SSAMSQKMR 0.989 *S*

35Gossypium 62 QARPSRNRK 0.972 *S*

35Gossypium 74 GTGLSPPPP 0.681 *S*

35Gossypium 107 PPPSSTTSL 0.773 *S*

35Gossypium 110 SSTTSLVSL 0.707 *S*

35Gossypium 191 DDIESESMP 0.556 *S*

35Gossypium 253 VPVKSKGFS 0.951 *S*

35Gossypium 287 VVRLSCQSK 0.935 *S*

_________________________^_________________

Threonine predictions

Name Pos Context Score Pred

_________________________v_________________

35Gossypium 71 RGRGTGLSP 0.725 *T*

35Gossypium 80 PPPTTTAMK 0.618 *T*

35Gossypium 81 PPTTTAMKR 0.741 *T*

35Gossypium 88 KRARTQYFL 0.511 *T*

_________________________^_________________

Tyrosine predictions

Name Pos Context Score Pred

_________________________v_________________

35Gossypium 49 RQSPYLRNL 0.551 *Y*

35Gossypium 212 ANSAYKAMV 0.667 *Y*

_________________________^_________________


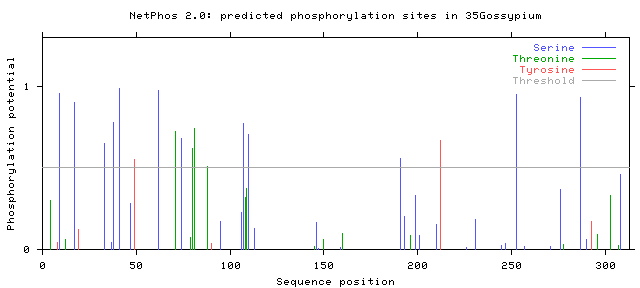


**TcSdr4L**

MIKTLNPYSTTAKTAEIMSRYRPIAPKPEVPANSLNENSAMSQKIRQSPYLRNLWPQLQARPTRTRKRGRAALSPPTLKR 80

ARTHVLGLSSPSPVTSPAKNLSLQGFSHGIPQLSVPNFVNTGGGLEISCAPPASLVTLPLLPCPPSGPIAANKATLPALN 160

CMEPCGGEKVIDLNTVAEIPEEKDLLKQLQGPVTSGVIAPQPIRPVGSSISVGCISEDPSLTPPMQVPKKSEEVEEEVES 240

EALPAVISDSNNKVRLANSAYKEMVGQPECPWLDSMVTVEGRAGGNSCKRICGEVMLHLSDSRVPVTSNRFSCWVRIDWG 320

SDGKKSSINAFCDVIRLSCQSKDYLFTWRFHTHNREATHSSCNV 400

.........TT.......S..............S.......S.......Y..............T........S..T... 80

..T........S...S................................................................ 160

................................................S..........S..........S......... 240

....................Y.........................S....................S............ 320

.....S...........S.............T.....T.S.... 400

Phosphorylation sites predicted: Ser: 14 Thr: 7 Tyr: 2

Serine predictions

Name Pos Context Score Pred

_________________________v_________________

36Theobroma 19 AEIMSRYRP 0.903 *S*

36Theobroma 34 VPANSLNEN 0.839 *S*

36Theobroma 42 NSAMSQKIR 0.945 *S*

36Theobroma 74 RAALSPPTL 0.844 *S*

36Theobroma 92 LSSPSPVTS 0.961 *S*

36Theobroma 96 SPVTSPAKN 0.810 *S*

36Theobroma 209 PVGSSISVG 0.758 *S*

36Theobroma 220 SEDPSLTPP 0.831 *S*

36Theobroma 231 VPKKSEEVE 0.983 *S*

36Theobroma 287 AGGNSCKRI 0.969 *S*

36Theobroma 308 VPVTSNRFS 0.927 *S*

36Theobroma 326 DGKKSSINA 0.980 *S*

36Theobroma 338 VIRLSCQSK 0.808 *S*

36Theobroma 360 EATHSSCNV 0.503 *S*

_________________________^_________________

Threonine predictions

Name Pos Context Score Pred

_________________________v_________________

36Theobroma 10 NPYSTTAKT 0.696 *T*

36Theobroma 11 PYSTTAKTA 0.623 *T*

36Theobroma 65 RPTRTRKRG 0.874 *T*

36Theobroma 77 LSPPTLKRA 0.648 *T*

36Theobroma 83 KRARTHVLG 0.571 *T*

36Theobroma 352 WRFHTHNRE 0.544 *T*

36Theobroma 358 NREATHSSC 0.754 *T*

_________________________^_________________

Tyrosine predictions

Name Pos Context Score Pred

_________________________v_________________

36Theobroma 50 RQSPYLRNL 0.551 *Y*

36Theobroma 261 ANSAYKEMV 0.824 *Y*

_________________________^_________________


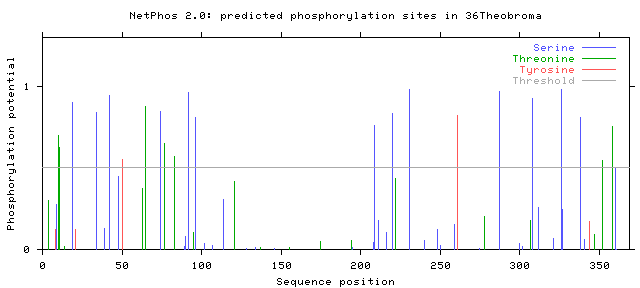


**AtSdr4L**

MIKILNPHSHHSQTTTLKTAEILSKYRPIAPKPGTPRVNDDDPSSSMSHKISQSPYLRNLWPQLQARPTRTRKRGRGGMG 80

PSSLAMKRPKSSCGSSSTSTISTQRVLGPIKTLSFQAFTHHRLPNLPQVGYGFENGVSSTLVTLPLLQCSPPSSKCMEPE 160

IKGKGVIDLNKTAEVIQERDFLTQLQGPITTTTTATTSRVISPQPIRPVCSKINVAYINPLTNPSPTSQTSKKSPREVEE 240

DVESDDLPSVITDSNSRVRLVNSAYKEMMGQPECSWLDSMVRGKRICGEVMINFCESKIPVMTENNGFSCWVRIDWGRDG 320

KEEYMHAFCDVTKLACDSKDYVFTWRFHTTTDRR 400

........S..S..TT.......S..........T........SSS.S...S...Y..............T......... 80

..........SS..SS..S...T.................................................SS...... 160

...............................TT...TS...S..............Y.......S..S.TS..S...... 240

...............S........Y.........S............................................. 320

...Y................Y.......TTT... 400

Phosphorylation sites predicted: Ser: 23 Thr: 12 Tyr: 5

Serine predictions

Name Pos Context Score Pred

_________________________v_________________

37ArabidopA 9 LNPHSHHSQ 0.942 *S*

37ArabidopA 12 HSHHSQTTT 0.831 *S*

37ArabidopA 24 AEILSKYRP 0.880 *S*

37ArabidopA 44 DDDPSSSMS 0.699 *S*

37ArabidopA 45 DDPSSSMSH 0.972 *S*

37ArabidopA 46 DPSSSMSHK 0.691 *S*

37ArabidopA 48 SSSMSHKIS 0.987 *S*

37ArabidopA 52 SHKISQSPY 0.781 *S*

37ArabidopA 91 KRPKSSCGS 0.996 *S*

37ArabidopA 92 RPKSSCGSS 0.894 *S*

37ArabidopA 95 SSCGSSSTS 0.510 *S*

37ArabidopA 96 SCGSSSTST 0.869 *S*

37ArabidopA 99 SSSTSTIST 0.986 *S*

37ArabidopA 153 CSPPSSKCM 0.972 *S*

37ArabidopA 154 SPPSSKCME 0.770 *S*

37ArabidopA 198 TATTSRVIS 0.523 *S*

37ArabidopA 202 SRVISPQPI 0.994 *S*

37ArabidopA 225 LTNPSPTSQ 0.874 *S*

37ArabidopA 228 PSPTSQTSK 0.855 *S*

37ArabidopA 231 TSQTSKKSP 0.997 *S*

37ArabidopA 234 TSKKSPREV 0.998 *S*

37ArabidopA 256 TDSNSRVRL 0.953 *S*

37ArabidopA 275 QPECSWLDS 0.940 *S*

_________________________^_________________

Threonine predictions

Name Pos Context Score Pred

_________________________v_________________

37ArabidopA 15 HSQTTTLKT 0.761 *T*

37ArabidopA 16 SQTTTLKTA 0.804 *T*

37ArabidopA 35 PKPGTPRVN 0.981 *T*

37ArabidopA 69 QARPTRTRK 0.377 .

37ArabidopA 71 RPTRTRKRG 0.874 *T*

37ArabidopA 103 STISTQRVL 0.957 *T*

37ArabidopA 192 PITTTTTAT 0.602 *T*

37ArabidopA 193 ITTTTTATT 0.520 *T*

37ArabidopA 197 TTATTSRVI 0.915 *T*

37ArabidopA 230 PTSQTSKKS 0.976 *T*

37ArabidopA 349 WRFHTTTDR 0.698 *T*

37ArabidopA 350 RFHTTTDRR 0.864 *T*

37ArabidopA 351 FHTTTDRR- 0.703 *T*

_________________________^_________________

Tyrosine predictions

Name Pos Context Score Pred

_________________________v_________________

37ArabidopA 56 SQSPYLRNL 0.784 *Y*

37ArabidopA 217 INVAYINPL 0.712 *Y*

37ArabidopA 265 VNSAYKEMM 0.707 *Y*

37ArabidopA 324 GKEEYMHAF 0.649 *Y*

37ArabidopA 341 DSKDYVFTW 0.837 *Y*

_________________________^_________________


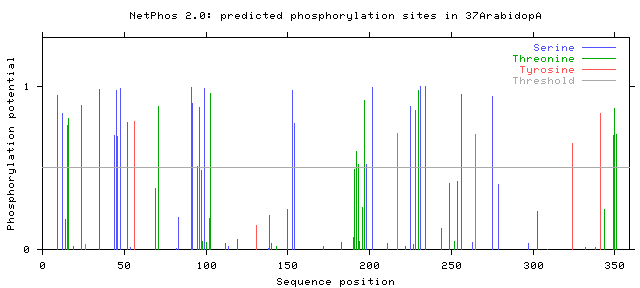


**BsSdr4L**

MIKILNPHSHHSHSTTTLKTAEILSKYRPIAPRPGTPRVNDDDPSSSMSHKITQSPYLRNLWPQLQARPTRTRKRGRGGM 80

GPSSLAMKRPKSSCASSPTSSTTTTTTQRVIGPIKTLSFQGFTHHGLPNLTQAGYALENGGSSALVNLPLLQCSPPLPSK 160

CMEPEIKGKGVIDLNKTAEVIQERDFLKQLQGPITTTTTTTTTTTSRVISPQPIRPVFSKINVAYINPLINPSPLPQTSN 240

KSPSEVEEEVESDDLPSVITDSNNRVRLVNSAYKEMMGQPECSWLDSMVRGKRICGGVMIHFCESKIPVMTENNGFSCWV 320

RIEWGRDGKEEYMHAFCDVMKLACESKDHVFTWRFHTTTDRRETCQSSCNV 400

........S..S.S..T.......S..........T........SSS.S......................T........ 80

...........SS...S..SS.....T..................................................... 160

...................................T.T.....TTS...S..............Y.............S. 240

.S.S............................Y.........S..................................... 320

...........Y........................TTT....T....... 400

Phosphorylation sites predicted: Ser: 19 Thr: 12 Tyr: 3

Serine predictions

Name Pos Context Score Pred

_________________________v_________________

38Boecheras 9 LNPHSHHSH 0.952 *S*

38Boecheras 12 HSHHSHSTT 0.909 *S*

38Boecheras 14 HHSHSTTTL 0.956 *S*

38Boecheras 25 AEILSKYRP 0.880 *S*

38Boecheras 45 DDDPSSSMS 0.699 *S*

38Boecheras 46 DDPSSSMSH 0.972 *S*

38Boecheras 47 DPSSSMSHK 0.691 *S*

38Boecheras 49 SSSMSHKIT 0.992 *S*

38Boecheras 92 KRPKSSCAS 0.995 *S*

38Boecheras 93 RPKSSCASS 0.675 *S*

38Boecheras 97 SCASSPTSS 0.990 *S*

38Boecheras 100 SSPTSSTTT 0.974 *S*

38Boecheras 101 SPTSSTTTT 0.874 *S*

38Boecheras 206 TTTTSRVIS 0.591 *S*

38Boecheras 210 SRVISPQPI 0.994 *S*

38Boecheras 239 LPQTSNKSP 0.904 *S*

38Boecheras 242 TSNKSPSEV 0.996 *S*

38Boecheras 244 NKSPSEVEE 0.997 *S*

38Boecheras 283 QPECSWLDS 0.940 *S*

_________________________^_________________

Threonine predictions

Name Pos Context Score Pred

_________________________v_________________

38Boecheras 17 HSTTTLKTA 0.943 *T*

38Boecheras 36 PRPGTPRVN 0.995 *T*

38Boecheras 72 RPTRTRKRG 0.874 *T*

38Boecheras 107 TTTTTQRVI 0.841 *T*

38Boecheras 196 GPITTTTTT 0.646 *T*

38Boecheras 198 ITTTTTTTT 0.556 *T*

38Boecheras 204 TTTTTTSRV 0.617 *T*

38Boecheras 205 TTTTTSRVI 0.905 *T*

38Boecheras 357 WRFHTTTDR 0.698 *T*

38Boecheras 358 RFHTTTDRR 0.864 *T*

38Boecheras 359 FHTTTDRRE 0.898 *T*

38Boecheras 364 DRRETCQSS 0.928 *T*

_________________________^_________________

Tyrosine predictions

Name Pos Context Score Pred

_________________________v_________________

38Boecheras 225 INVAYINPL 0.712 *Y*

38Boecheras 273 VNSAYKEMM 0.707 *Y*

38Boecheras 332 GKEEYMHAF 0.649 *Y*

_________________________^_________________


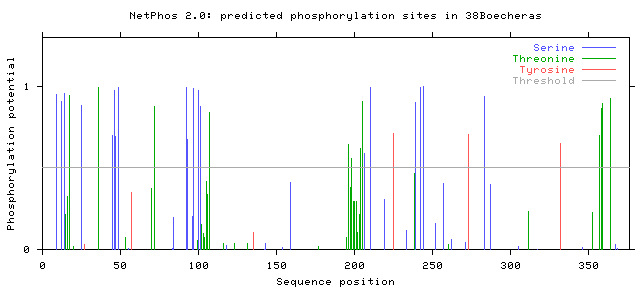


**BrSdr4L1**

MIKILSPHNSHSTTTTTLKTAEILSKYRPIAPKPGTTPQVNDNDSSSSSMSHKISQSPYLRNLWPQLQARPTRTRKRGRG 80

GMGPTSPLSLKRHKPSSSASTTTTTPQRVFGPIKTLSFQAFSHAGIPNLAQVGYALENGGSPSLVTLPLLQCSPSPPSKC 160

MEPEIKVKEAIDLNKTAEVIQERDFLKQLQEPITTTTTSKVIAPQAIRPVCSRINVACINPLTNSSQAIKKSPQDVEEEF 240

ESDDVPAIISDSNNRVRLVNSAYKEMMGQPECSWLDSMVRGKRICGEVMIRCCEAEIPENNGFSCWVRIEWGRDGKEEFV 320

HAFCDVMKLECDSKDYVFTWRFHTTTRENLSTKLSCLV 400

.....S.....S...TT.......S...........T.......S.SSS.S...S...Y..............T...... 80

.....S..S......SS..S....T....................................................S.. 160

....................................TTS................................S........ 240

......................Y.........S............................................... 320

...............Y.......TT.....S....... 400

Phosphorylation sites predicted: Ser: 19 Thr: 9 Tyr: 3

Serine predictions

Name Pos Context Score Pred

_________________________v_________________

39BrassicaB 6 IKILSPHNS 0.960 *S*

39BrassicaB 12 HNSHSTTTT 0.812 *S*

39BrassicaB 25 AEILSKYRP 0.880 *S*

39BrassicaB 45 NDNDSSSSS 0.786 *S*

39BrassicaB 47 NDSSSSSMS 0.974 *S*

39BrassicaB 48 DSSSSSMSH 0.916 *S*

39BrassicaB 49 SSSSSMSHK 0.919 *S*

39BrassicaB 51 SSSMSHKIS 0.992 *S*

39BrassicaB 55 SHKISQSPY 0.781 *S*

39BrassicaB 86 MGPTSPLSL 0.945 *S*

39BrassicaB 89 TSPLSLKRH 0.997 *S*

39BrassicaB 96 RHKPSSSAS 0.956 *S*

39BrassicaB 97 HKPSSSAST 0.979 *S*

39BrassicaB 100 SSSASTTTT 0.961 *S*

39BrassicaB 158 PSPPSKCME 0.970 *S*

39BrassicaB 199 TTTTSKVIA 0.514 *S*

39BrassicaB 232 AIKKSPQDV 0.972 *S*

39BrassicaB 273 QPECSWLDS 0.940 *S*

39BrassicaB 351 RENLSTKLS 0.991 *S*

_________________________^_________________

Threonine predictions

Name Pos Context Score Pred

_________________________v_________________

39BrassicaB 16 STTTTTLKT 0.771 *T*

39BrassicaB 17 TTTTTLKTA 0.812 *T*

39BrassicaB 37 KPGTTPQVN 0.789 *T*

39BrassicaB 74 RPTRTRKRG 0.874 *T*

39BrassicaB 105 TTTTTPQRV 0.831 *T*

39BrassicaB 197 ITTTTTSKV 0.844 *T*

39BrassicaB 198 TTTTTSKVI 0.842 *T*

39BrassicaB 344 WRFHTTTRE 0.665 *T*

39BrassicaB 345 RFHTTTREN 0.991 *T*

_________________________^_________________

Tyrosine predictions

Name Pos Context Score Pred

_________________________v_________________

39BrassicaB 59 SQSPYLRNL 0.784 *Y*

39BrassicaB 263 VNSAYKEMM 0.707 *Y*

39BrassicaB 336 DSKDYVFTW 0.837 *Y*

_________________________^_________________


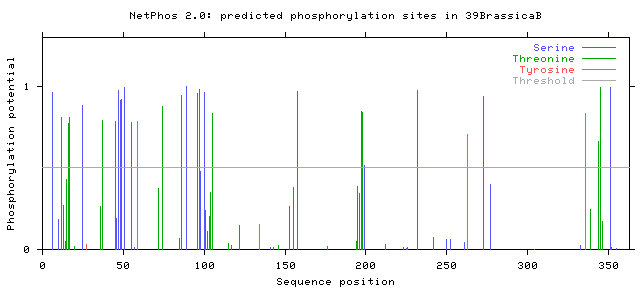


**BrSdr4L2**

MIKILSSHNSHHSHSTTTTTLKTAEILSKYRPIAPRPGTTQVNDNDSSSSSSYMSHKISQSPYLRHLWPQLQARPTRTRK 80

RGRGGMGPTSHLSLKRPKSLATSTKTPTQRVFGPIKTLAFQAFSHAGLPNLTTQVGYALENGGSPALVTLPLLQCSPLSS 160

KCMEPEIKVKGLIDLNKSAETIQERDFLKQLQGPITTTTAAEASRVITPQPIRPVCSRINVACINPLSNPSQISKKSPQE 240

VEEEVESDVLPAIISDSNNKVKLVNSAYKEMMGQPECSWLDSMVKVKRICGEVVIQFCESKISEKNNGYSCWVKIEWGRD 320

GKEELVHAFCDVMKRECDSKDYVFTWRFHITAKETCQPSYNA 400

.....S...S..S.S...TT.......S..........TT......S.S.SSY.S...S...Y..............T.. 80

.........S..S.....S...ST...T..................................................S. 160

.................S..T...............T.T........T......................S..S..S... 240

...........................Y.........S.....................S..S................. 320

.....................Y........T........... 400

Phosphorylation sites predicted: Ser: 23 Thr: 12 Tyr: 4

Serine predictions

Name Pos Context Score Pred

_________________________v_________________

40BraraH020 6 IKILSSHNS 0.548 *S*

40BraraH020 10 SSHNSHHSH 0.507 *S*

40BraraH020 13 NSHHSHSTT 0.788 *S*

40BraraH020 15 HHSHSTTTT 0.975 *S*

40BraraH020 28 AEILSKYRP 0.880 *S*

40BraraH020 47 NDNDSSSSS 0.897 *S*

40BraraH020 49 NDSSSSSSY 0.915 *S*

40BraraH020 51 SSSSSSYMS 0.984 *S*

40BraraH020 52 SSSSSYMSH 0.992 *S*

40BraraH020 55 SSYMSHKIS 0.988 *S*

40BraraH020 59 SHKISQSPY 0.781 *S*

40BraraH020 90 MGPTSHLSL 0.645 *S*

40BraraH020 93 TSHLSLKRP 0.992 *S*

40BraraH020 99 KRPKSLATS 0.897 *S*

40BraraH020 103 SLATSTKTP 0.992 *S*

40BraraH020 159 CSPLSSKCM 0.970 *S*

40BraraH020 178 DLNKSAETI 0.783 *S*

40BraraH020 231 LSNPSQISK 0.605 *S*

40BraraH020 234 PSQISKKSP 0.884 *S*

40BraraH020 237 ISKKSPQEV 0.989 *S*

40BraraH020 278 QPECSWLDS 0.940 *S*

40BraraH020 300 QFCESKISE 0.805 *S*

40BraraH020 303 ESKISEKNN 0.982 *S*

_________________________^_________________

Threonine predictions

Name Pos Context Score Pred

_________________________v_________________

40BraraH020 19 STTTTTLKT 0.771 *T*

40BraraH020 20 TTTTTLKTA 0.812 *T*

40BraraH020 39 PRPGTTQVN 0.682 *T*

40BraraH020 40 RPGTTQVND 0.886 *T*

40BraraH020 78 RPTRTRKRG 0.874 *T*

40BraraH020 104 LATSTKTPT 0.640 *T*

40BraraH020 108 TKTPTQRVF 0.758 *T*

40BraraH020 181 KSAETIQER 0.579 *T*

40BraraH020 197 GPITTTTAA 0.702 *T*

40BraraH020 199 ITTTTAAEA 0.848 *T*

40BraraH020 208 SRVITPQPI 0.689 *T*

40BraraH020 351 RFHITAKET 0.980 *T*

_________________________^_________________

Tyrosine predictions

Name Pos Context Score Pred

_________________________v_________________

40BraraH020 53 SSSSYMSHK 0.958 *Y*

40BraraH020 63 SQSPYLRHL 0.638 *Y*

40BraraH020 268 VNSAYKEMM 0.707 *Y*

40BraraH020 342 DSKDYVFTW 0.837 *Y*

_________________________^_________________


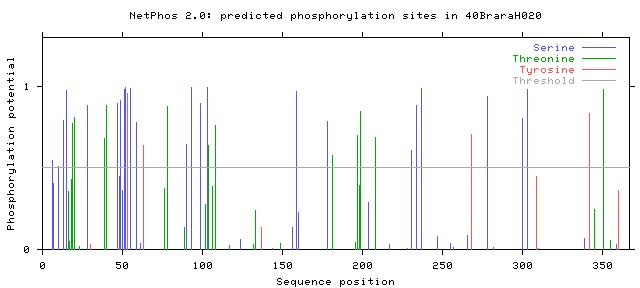


**CgSdr4L**

MIKILNPHSTTTLKTAEILSKYRPIAPKPGTPRLNDDDPSSSMSHKISQSPYLRNLWPQLQARPTRTRKRGRGGMGPSSL 80

AMKRPKSSCVSSPTSSSTTTTQRVIGPIKTLSFQAFTHHGLPSLTQVGYALENGASSALVTLPLLQCSPPLPSKCMEPEI 160

KGKGVIDLNKTAEVIQEIDFLKQLQGPITTTTTTTSRVISPQPIRPLCSKINVAYINPLTNPSPLPNQTSKKSPSEVEEE 240

VESDDLPSVITDSSNRVRLVNSAYKEMMGQPECSWLDSMVRGRRICGEVMIHFCESKIPMMTENNGFSCWVRIEWGRDGK 320

EEYMHAFCDVMKLACESKDYVFTWRFHATTDRRETCQSSCNV 400

........S..T.......S..........T........SSS.S...S...Y..............T............. 80

......SS...S..SSS..TT........................................................... 160

.............................T...TTS...S..............Y.............TS..S.S..... 240

.......................Y.........S.............................................. 320

..Y................Y........TT....T....... 400

Phosphorylation sites predicted: Ser: 19 Thr: 12 Tyr: 5

Serine predictions

Name Pos Context Score Pred

_________________________v_________________

41CapselCag 9 LNPHSTTTL 0.779 *S*

41CapselCag 20 AEILSKYRP 0.880 *S*

41CapselCag 40 DDDPSSSMS 0.699 *S*

41CapselCag 41 DDPSSSMSH 0.972 *S*

41CapselCag 42 DPSSSMSHK 0.691 *S*

41CapselCag 44 SSSMSHKIS 0.987 *S*

41CapselCag 48 SHKISQSPY 0.781 *S*

41CapselCag 87 KRPKSSCVS 0.994 *S*

41CapselCag 88 RPKSSCVSS 0.852 *S*

41CapselCag 92 SCVSSPTSS 0.979 *S*

41CapselCag 95 SSPTSSSTT 0.955 *S*

41CapselCag 96 SPTSSSTTT 0.777 *S*

41CapselCag 97 PTSSSTTTT 0.580 *S*

41CapselCag 196 TTTTSRVIS 0.591 *S*

41CapselCag 200 SRVISPQPI 0.994 *S*

41CapselCag 230 PNQTSKKSP 0.831 *S*

41CapselCag 233 TSKKSPSEV 0.997 *S*

41CapselCag 235 KKSPSEVEE 0.997 *S*

41CapselCag 274 QPECSWLDS 0.940 *S*

_________________________^_________________

Threonine predictions

Name Pos Context Score Pred

_________________________v_________________

41CapselCag 12 HSTTTLKTA 0.943 *T*

41CapselCag 31 PKPGTPRLN 0.941 *T*

41CapselCag 67 RPTRTRKRG 0.874 *T*

41CapselCag 100 SSTTTTQRV 0.514 *T*

41CapselCag 101 STTTTQRVI 0.931 *T*

41CapselCag 190 GPITTTTTT 0.646 *T*

41CapselCag 194 TTTTTTSRV 0.617 *T*

41CapselCag 195 TTTTTSRVI 0.905 *T*

41CapselCag 229 LPNQTSKKS 0.929 *T*

41CapselCag 349 RFHATTDRR 0.657 *T*

41CapselCag 350 FHATTDRRE 0.907 *T*

41CapselCag 355 DRRETCQSS 0.928 *T*

_________________________^_________________

Tyrosine predictions

Name Pos Context Score Pred

_________________________v_________________

41CapselCag 52 SQSPYLRNL 0.784 *Y*

41CapselCag 215 INVAYINPL 0.712 *Y*

41CapselCag 264 VNSAYKEMM 0.707 *Y*

41CapselCag 323 GKEEYMHAF 0.649 *Y*

41CapselCag 340 ESKDYVFTW 0.908 *Y*

_________________________^_________________


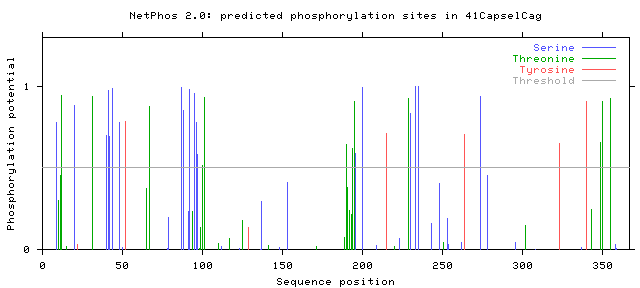


**CrSdr4L**

MIKILNPHSTTTLKTAEILSKYRPIAPKPGTPRVNDDDPSSSMSHKISQSPYLRNLWPQLQARPTRTRKRGRGGMGPSSL 80

AMKRPKSSCVSSPTSSSTTTTQRVIGPIKTLSFQAFTHHGLPSLTQVGYALENGASSALVTLPLLQCSPPLPSKCMEPEI 160

KGKGVIDLNKTAEVIQEIDFLKQLQGPITTTTTTTSRVISPQPIRPLCSKINVAYINPLTNPSPLPNQTSKKSPSEVEEE 240

VESDDLPSVITDSSNRVRLVNSAYKEMMGQPECSWLDSMVRGRRICGEVMIHFCESKIPMMTENNGFSCWVRIEWGRDGK 320

EEYMHAFCDVMKLACESKDYVFTWRFHATTDRREACQSSCNV 400

........S..T.......S..........T........SSS.S...S...Y..............T............. 80

......SS...S..SSS..TT........................................................... 160

.............................T...TTS...S..............Y.............TS..S.S..... 240

.......................Y.........S.............................................. 320

..Y................Y........TT............ 400

Phosphorylation sites predicted: Ser: 19 Thr: 11 Tyr: 5

Serine predictions

Name Pos Context Score Pred

_________________________v_________________

42CapselCar 9 LNPHSTTTL 0.779 *S*

42CapselCar 20 AEILSKYRP 0.880 *S*

42CapselCar 40 DDDPSSSMS 0.699 *S*

42CapselCar 41 DDPSSSMSH 0.972 *S*

42CapselCar 42 DPSSSMSHK 0.691 *S*

42CapselCar 44 SSSMSHKIS 0.987 *S*

42CapselCar 48 SHKISQSPY 0.781 *S*

42CapselCar 87 KRPKSSCVS 0.994 *S*

42CapselCar 88 RPKSSCVSS 0.852 *S*

42CapselCar 92 SCVSSPTSS 0.979 *S*

42CapselCar 95 SSPTSSSTT 0.955 *S*

42CapselCar 96 SPTSSSTTT 0.777 *S*

42CapselCar 97 PTSSSTTTT 0.580 *S*

42CapselCar 196 TTTTSRVIS 0.591 *S*

42CapselCar 200 SRVISPQPI 0.994 *S*

42CapselCar 230 PNQTSKKSP 0.831 *S*

42CapselCar 233 TSKKSPSEV 0.997 *S*

42CapselCar 235 KKSPSEVEE 0.997 *S*

42CapselCar 274 QPECSWLDS 0.940 *S*

.

_________________________^_________________

Threonine predictions

Name Pos Context Score Pred

_________________________v_________________

42CapselCar 12 HSTTTLKTA 0.943 *T*

42CapselCar 31 PKPGTPRVN 0.981 *T*

42CapselCar 67 RPTRTRKRG 0.874 *T*

42CapselCar 100 SSTTTTQRV 0.514 *T*

42CapselCar 101 STTTTQRVI 0.931 *T*

42CapselCar 190 GPITTTTTT 0.646 *T*

42CapselCar 194 TTTTTTSRV 0.617 *T*

42CapselCar 195 TTTTTSRVI 0.905 *T*

42CapselCar 229 LPNQTSKKS 0.929 *T*

42CapselCar 349 RFHATTDRR 0.657 *T*

42CapselCar 350 FHATTDRRE 0.907 *T*

_________________________^_________________

Tyrosine predictions

Name Pos Context Score Pred

_________________________v_________________

42CapselCar 52 SQSPYLRNL 0.784 *Y*

42CapselCar 215 INVAYINPL 0.712 *Y*

42CapselCar 264 VNSAYKEMM 0.707 *Y*

42CapselCar 323 GKEEYMHAF 0.649 *Y*

42CapselCar 340 ESKDYVFTW 0.908 *Y*

_________________________^_________________


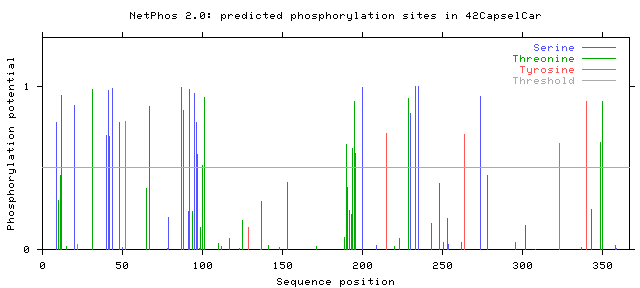


**EsSdr4L**

MIKILNPHQSHHSHSTTTTLKTAEILSKYRPIAPKPGTTSSQVNDNDTSSSMSHKISQSPYLRNLWPQLQARPTRTRKRG 80

RGGMGPTSPLALKRPKSSSPSATISSTTTTTTPRVFGPIKTLSFQAFPHGLPSLAQVGYTLENNGGGSSALVTLPLLQCS 160

PPPPSKCMEPEVKGKVVIDLNKTAEVIQERDFLKQLQGPTVTTTATDTNTSRVIAPQAIRPVCSRINVACINPLTNPSPP 240

YQISKKLPQEVEEEVESDDLPTIISDSKNRVRLVNSAYKEMMGQPECSWLDSMVRGKRICGEVMIHLCETKIPENNGFSC 320

WVRIEWGRDGKEEFVHAFCDVMKLACDSKDYVFTWRFHTRTRETCQSSCNA 400

............S.S..TT.......S...........T..........S..S...S...Y..............T.... 80

.......S........SSS.S........T.T................................................ 160

....S............................................TS............................. 240

Y.........................S..........Y.........S................................ 320

..............................Y.......T....T....... 400

Phosphorylation sites predicted: Ser: 15 Thr: 9 Tyr: 4

Serine predictions

Name Pos Context Score Pred

_________________________v_________________

43Eutremasa 13 QSHHSHSTT 0.809 *S*

43Eutremasa 15 HHSHSTTTT 0.971 *S*

43Eutremasa 27 AEILSKYRP 0.880 *S*

43Eutremasa 50 NDTSSSMSH 0.922 *S*

43Eutremasa 53 SSSMSHKIS 0.995 *S*

43Eutremasa 57 SHKISQSPY 0.781 *S*

43Eutremasa 88 MGPTSPLAL 0.927 *S*

43Eutremasa 97 KRPKSSSPS 0.992 *S*

43Eutremasa 98 RPKSSSPSA 0.744 *S*

43Eutremasa 99 PKSSSPSAT 0.996 *S*

43Eutremasa 101 SSSPSATIS 0.867 *S*

43Eutremasa 165 PPPPSKCME 0.896 *S*

43Eutremasa 211 DTNTSRVIA 0.591 *S*

43Eutremasa 267 IISDSKNRV 0.817 *S*

43Eutremasa 288 QPECSWLDS 0.940 *S*

_________________________^_________________

Threonine predictions

Name Pos Context Score Pred

_________________________v_________________

43Eutremasa 18 HSTTTTLKT 0.797 *T*

43Eutremasa 19 STTTTLKTA 0.916 *T*

43Eutremasa 39 KPGTTSSQV 0.793 *T*

43Eutremasa 76 RPTRTRKRG 0.874 *T*

43Eutremasa 110 STTTTTTPR 0.662 *T*

43Eutremasa 112 TTTTTPRVF 0.991 *T*

43Eutremasa 210 TDTNTSRVI 0.594 *T*

43Eutremasa 359 WRFHTRTRE 0.526 *T*

43Eutremasa 364 RTRETCQSS 0.583 *T*

_________________________^_________________

Tyrosine predictions

Name Pos Context Score Pred

_________________________v_________________

43Eutremasa 61 SQSPYLRNL 0.784 *Y*

43Eutremasa 241 PSPPYQISK 0.891 *Y*

43Eutremasa 278 VNSAYKEMM 0.707 *Y*

43Eutremasa 351 DSKDYVFTW 0.837 *Y*

_________________________^_________________


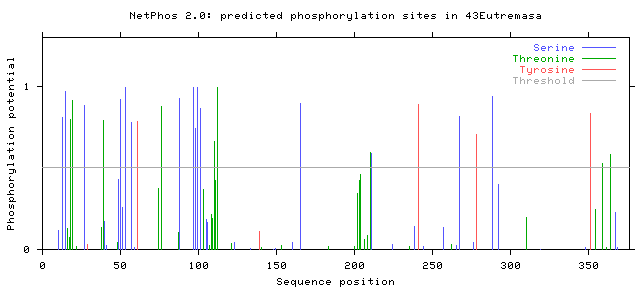


**CcSdr4L**

MIRTMSPFSAATTAKTAEIMSRYRPIAPKPEAPVSPMSESAKIRQSPYLRNLWPQLQARPTRTRKRGRAAISPPTIKRSR 80

THLFGLSSPCHATSSPAKNPSLQGFAHPHAHGVAQFTLPNHLATVPASCSLDNPASTATTITTNLVTLPLLPCTPVVSEQ 160

TTPLELSNCVESFGEVNVIDLNKVAEIPEEKDLLLQLQGPPSPTNNVISPRPVRPVGSSISVGCINEDSRLTPEAQVVTK 240

KPEEVEEEVESEALPAVISDSHNRVRMANSAFKEMVGQPECSWLDLMVINNSSCKRICGEVMLNFSDSAGMPNNSSNGFS 320

CWVRIEWGSDGKKNSVDAFCDVIRLSCEIKDYLFAWRFHPQASQSCCHV 400

.....S..S...T.......S.............S..S.......S.Y..............T........S..T..... 80

............T................................................................... 160

TT.......................................S......S.........S............T......T. 240

....................S....................S..........S........................... 320

..............S..........S....................... 400

Phosphorylation sites predicted: Ser: 15 Thr: 8 Tyr: 1

Serine predictions

Name Pos Context Score Pred

_________________________v_________________

44Citruscle 6 IRTMSPFSA 0.988 *S*

44Citruscle 9 MSPFSAATT 0.805 *S*

44Citruscle 21 AEIMSRYRP 0.903 *S*

44Citruscle 35 EAPVSPMSE 0.968 *S*

44Citruscle 38 VSPMSESAK 0.940 *S*

44Citruscle 46 KIRQSPYLR 0.753 *S*

44Citruscle 72 RAAISPPTI 0.675 *S*

44Citruscle 202 QGPPSPTNN 0.988 *S*

44Citruscle 209 NNVISPRPV 0.978 *S*

44Citruscle 219 PVGSSISVG 0.758 *S*

44Citruscle 261 VISDSHNRV 0.508 *S*

44Citruscle 282 QPECSWLDL 0.851 *S*

44Citruscle 293 INNSSCKRI 0.613 *S*

44Citruscle 335 GKKNSVDAF 0.550 *S*

44Citruscle 346 VIRLSCEIK 0.974 *S*

_________________________^_________________

Threonine predictions

Name Pos Context Score Pred

_________________________v_________________

44Citruscle 13 SAATTAKTA 0.899 *T*

44Citruscle 63 RPTRTRKRG 0.874 *T*

44Citruscle 75 ISPPTIKRS 0.780 *T*

44Citruscle 93 PCHATSSPA 0.620 *T*

44Citruscle 161 VSEQTTPLE 0.699 *T*

44Citruscle 162 SEQTTPLEL 0.957 *T*

44Citruscle 232 DSRLTPEAQ 0.953 *T*

44Citruscle 239 AQVVTKKPE 0.715 *T*

_________________________^_________________

Tyrosine predictions

Name Pos Context Score Pred

_________________________v_________________

44Citruscle 48 RQSPYLRNL 0.551 *Y*

_________________________^_________________


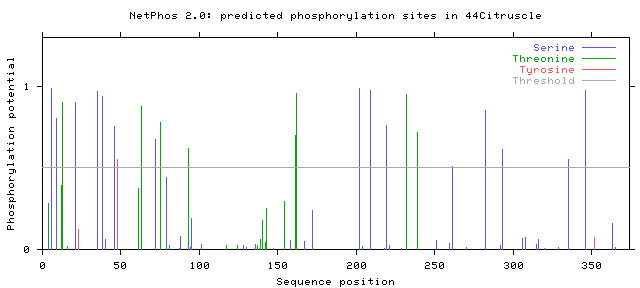


**CsSdr4L** MIRTMSPFSAATTAKTAEIMSRYRPIAPKPEAPVSPMSESAKIRQSPYLRNLWPQLQARPTRTRKRGRAAISPPTIKRSR 80

THLFGLSSPCHATSSPAKNPSLQGFAHPHAHGVAQFTLPNHLATVPASCSLDNPASTATTITTNLVTLPLLPCTPVVSEQ 160

TTPLELSNCVESFGEVNVIDLNKVAEIPEEKDLLLQLQGPPSPTNNVISPRPVRPVGSSISVGCINEDSRLTPEAQVVTK 240

KPEEVEEEVESEALPTVISDSHNRVRMANSAFKEMVGQPECSWLDLMVINNSSCKRICGEVMLNFSDSAGMPNNSSNGFS 320

CWVRIEWGSDGRRTR 400

.....S..S...T.......S.............S..S.......S.Y..............T........S..T..... 80

............T................................................................... 160

TT.......................................S......S.........S............T......T. 240

....................S....................S..........S........................... 320

.............T. 400

Phosphorylation sites predicted: Ser: 13 Thr: 9 Tyr: 1

Serine predictions

Name Pos Context Score Pred

_________________________v_________________

45Citrussin 6 IRTMSPFSA 0.988 *S*

45Citrussin 9 MSPFSAATT 0.805 *S*

45Citrussin 21 AEIMSRYRP 0.903 *S*

45Citrussin 35 EAPVSPMSE 0.968 *S*

45Citrussin 38 VSPMSESAK 0.940 *S*

45Citrussin 46 KIRQSPYLR 0.753 *S*

45Citrussin 72 RAAISPPTI 0.675 *S*

45Citrussin 202 QGPPSPTNN 0.988 *S*

45Citrussin 209 NNVISPRPV 0.978 *S*

45Citrussin 219 PVGSSISVG 0.758 *S*

45Citrussin 261 VISDSHNRV 0.913 *S*

45Citrussin 282 QPECSWLDL 0.851 *S*

45Citrussin 293 INNSSCKRI 0.613 *S*

_________________________^_________________

Threonine predictions

Name Pos Context Score Pred

_________________________v_________________

45Citrussin 13 SAATTAKTA 0.899 *T*

45Citrussin 63 RPTRTRKRG 0.874 *T*

45Citrussin 75 ISPPTIKRS 0.780 *T*

45Citrussin 93 PCHATSSPA 0.620 *T*

45Citrussin 161 VSEQTTPLE 0.699 *T*

45Citrussin 162 SEQTTPLEL 0.957 *T*

45Citrussin 232 DSRLTPEAQ 0.953 *T*

45Citrussin 239 AQVVTKKPE 0.715 *T*

45Citrussin 334 DGRRTR--- 0.521 *T*

_________________________^_________________

Tyrosine predictions

Name Pos Context Score Pred

_________________________v_________________

45Citrussin 48 RQSPYLRNL 0.551 *Y*

_________________________^_________________


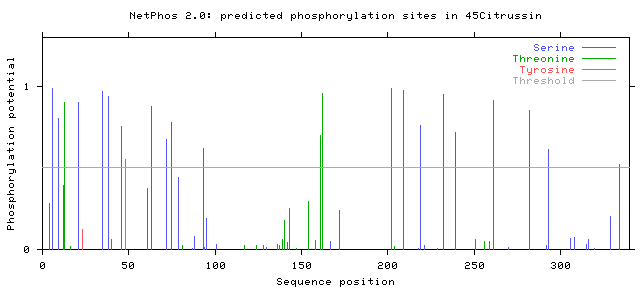


**CSaSdr4L** MIKTLPPPFPSTDKTAEIMSRYRPIAPKPESPFPTSDHSLNNIPHSSSSSSSSSFLRNVWPQLQARPTRTRKRSRPPPIS 80

PHSLKRTRITPSPNFSLHHHPFSSSLLPHLSLPSINSGFRDSSSNSNLVTLPLLPSVSDETETTTPVSEINFIKSFDEEK 160

GVEFSVDSSVVSEIPQEKDLLQQLQCPVSISNVITPHPVRPVGSSIRVGCINEAQNPVHSNNTPQLPKKPDEVEKEVESE 240

VLPAVISDSNNRVRMANSAYKEMVGQPECLWLDSMVTGDERLKGRRIGGEVMLHLSDAAAVPHSSNGFSCWVRIEWGNSD 320

GKKNSVTAFCDVIKLSCVSRDYLFTWRFHTQTRNNHNNNNNAFNPICINV 400

...........T.......S..........S.......S......S.SSSSS.S...............T...S.....S 80

..S......T.S.............................SSS...........S......TTT..S......S..... 160

....S...S..S......................T.........S................................... 240

...................Y................T........................................... 320

....S............................................. 400

Phosphorylation sites predicted: Ser: 25 Thr: 8 Tyr: 1

Serine predictions

Name Pos Context Score Pred

_________________________v_________________

46Cucumissa 20 AEIMSRYRP 0.903 *S*

46Cucumissa 31 PKPESPFPT 0.982 *S*

46Cucumissa 39 TSDHSLNNI 0.926 *S*

46Cucumissa 46 NIPHSSSSS 0.937 *S*

46Cucumissa 48 PHSSSSSSS 0.946 *S*

46Cucumissa 49 HSSSSSSSS 0.973 *S*

46Cucumissa 50 SSSSSSSSS 0.940 *S*

46Cucumissa 51 SSSSSSSSF 0.971 *S*

46Cucumissa 52 SSSSSSSFL 0.985 *S*

46Cucumissa 54 SSSSSFLRN 0.960 *S*

46Cucumissa 74 TRKRSRPPP 0.989 *S*

46Cucumissa 80 PPPISPHSL 0.985 *S*

46Cucumissa 83 ISPHSLKRT 0.998 *S*

46Cucumissa 92 RITPSPNFS 0.777 *S*

46Cucumissa 122 GFRDSSSNS 0.976 *S*

46Cucumissa 123 FRDSSSNSN 0.814 *S*

46Cucumissa 124 RDSSSNSNL 0.842 *S*

46Cucumissa 136 PLLPSVSDE 0.889 *S*

46Cucumissa 148 TTPVSEINF 0.963 *S*

46Cucumissa 155 NFIKSFDEE 0.975 *S*

46Cucumissa 165 GVEFSVDSS 0.645 *S*

46Cucumissa 169 SVDSSVVSE 0.830 *S*

46Cucumissa 172 SSVVSEIPQ 0.982 *S*

46Cucumissa 205 PVGSSIRVG 0.930 *S*

46Cucumissa 325 GKKNSVTAF 0.634 *S*

_________________________^_________________

Threonine predictions

Name Pos Context Score Pred

_________________________v_________________

46Cucumissa 12 PFPSTDKTA 0.561 *T*

46Cucumissa 70 RPTRTRKRS 0.918 *T*

46Cucumissa 90 RTRITPSPN 0.960 *T*

46Cucumissa 143 DETETTTPV 0.766 *T*

46Cucumissa 144 ETETTTPVS 0.804 *T*

46Cucumissa 145 TETTTPVSE 0.945 *T*

46Cucumissa 195 SNVITPHPV 0.661 *T*

46Cucumissa 277 DSMVTGDER 0.707 *T*

Tyrosine predictions

Name Pos Context Score Pred

_________________________v_________________

46Cucumissa 260 ANSAYKEMV 0.824 *Y*

_________________________^_________________


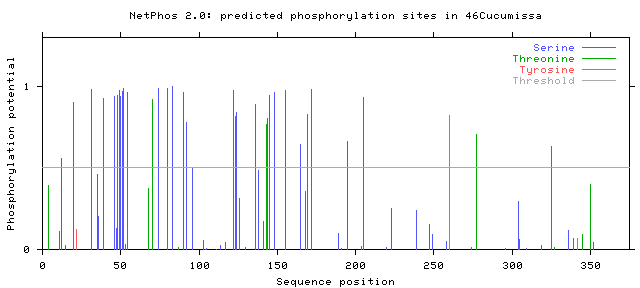


**FvSdr4L** MIKTLNPYNSTNTAKTAEIMSRYRPIAPKPETSPTTPTGESPALSQKIRDSPYLRNLWPQLQARPTRTRKRGRAAISPPT 80

LKRQRTSHVFGLSPACHVTSPVNNLTLDGFPHPLTQLALPNQLGSASSGLESTSLVTLPLLSYPSSVPIVPNQAVVTNQA 160

VVPAELNLLKPSGGEKLIDLNSVAEIPEEKDLLKQLQGTPSPATTPTSPNVIAPQPIRPVGSSISVGCISTDPSSAPAVQ 240

APKKPEDVEKEMESEALPAIISDSHNKVRMANAAYKEMVGQPECSWLDSMVATPCKRISGEVALQLSDTRVPTSSNGFSC 320

WVRIEWGNEMDKHAINAFCDVIKLACESKDYLFTWRFHTHSREASQSSSSEIATCN 400

............T.......S...........S..T........S.....S.Y..............T........S... 80

.....TS............S...........................S.............S.................. 160

...........S..........................T.S...T..S..............S..........S...... 240

..................................Y.........S.......T.....S..............SS..... 320

..............................Y.......T.S...S.SSSS...... 400

Phosphorylation sites predicted: Ser: 24 Thr: 8 Tyr: 3

Serine predictions

Name Pos Context Score Pred

_________________________v_________________

47Fragaria 21 AEIMSRYRP 0.903 *S*

47Fragaria 33 KPETSPTTP 0.946 *S*

47Fragaria 45 SPALSQKIR 0.969 *S*

47Fragaria 51 KIRDSPYLR 0.846 *S*

47Fragaria 77 RAAISPPTL 0.683 *S*

47Fragaria 87 RQRTSHVFG 0.988 *S*

47Fragaria 100 CHVTSPVNN 0.789 *S*

47Fragaria 128 GSASSGLES 0.869 *S*

47Fragaria 142 LPLLSYPSS 0.548 *S*

47Fragaria 172 LLKPSGGEK 0.632 *S*

47Fragaria 201 QGTPSPATT 0.619 *S*

47Fragaria 208 TTPTSPNVI 0.740 *S*

47Fragaria 223 PVGSSISVG 0.758 *S*

47Fragaria 234 STDPSSAPA 0.629 *S*

47Fragaria 285 QPECSWLDS 0.940 *S*

47Fragaria 299 CKRISGEVA 0.992 *S*

47Fragaria 314 RVPTSSNGF 0.868 *S*

47Fragaria 315 VPTSSNGFS 0.511 *S*

47Fragaria 361 FHTHSREAS 0.976 *S*

47Fragaria 365 SREASQSSS 0.971 *S*

47Fragaria 367 EASQSSSSE 0.877 *S*

47Fragaria 368 ASQSSSSEI 0.985 *S*

47Fragaria 369 SQSSSSEIA 0.889 *S*

47Fragaria 370 QSSSSEIAT 0.994 *S*

_________________________^_________________

Threonine predictions

Name Pos Context Score Pred

_________________________v_________________

47Fragaria 13 NSTNTAKTA 0.764 *T*

47Fragaria 36 TSPTTPTGE 0.839 *T*

47Fragaria 68 RPTRTRKRG 0.874 *T*

47Fragaria 86 KRQRTSHVF 0.903 *T*

47Fragaria 199 QLQGTPSPA 0.694 *T*

47Fragaria 205 SPATTPTSP 0.945 *T*

47Fragaria 293 SMVATPCKR 0.931 *T*

47Fragaria 359 WRFHTHSRE 0.698 *T*

_________________________^_________________

Tyrosine predictions

Name Pos Context Score Pred

_________________________v_________________

47Fragaria 53 RDSPYLRNL 0.877 *Y*

47Fragaria 275 ANAAYKEMV 0.616 *Y*

47Fragaria 351 ESKDYLFTW 0.530 *Y*

_________________________^_________________


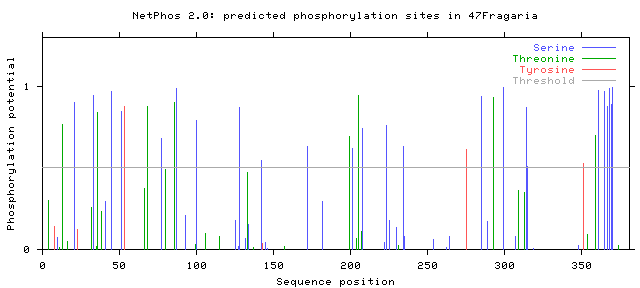


**GmSdr4L1** MIKTLNPYPNPAKTAEIMSRYRPIAPKPETSPNSMSEGPSSSSLSQKIKQSPYLRNLWPQLQARPTRTRKRGRAPLTLPS 80

SSLKRHKTTHHHVLGFCPPCHHVVTSSSSSPSKNLSLQGFAPPHPLPHHLGVLNCTMEKNNTNPSLVTLPLLPCSPTLTT 160

KPCAGEVINLNTKASVPEEKDLLQQLQKPVSNNIINVITPQPIRPIGSSISVVCISEDSTLSPLAQTPKKPNEVEQEVEN 240

EALPTVISDSNHRIRMANSAYKEMVGQPVCPWLESMGNLLQCRRISGEVTLNLSDSSTVIPTSSNGFSCWVRIEWLSEHN 320

NKKKNCINAFCDVMKLACESRDYLFTWRFHTRTTREASQSSCNA 400

..................S...........S..S.S...SS...S.......Y..............T............ 80

.S.....T...................S.S.S..............................................T. 160

..............S...................................S..........S....T............. 240

.........S..........Y........................S.......S........S.............S... 320

................................TT...S.S.... 400

Phosphorylation sites predicted: Ser: 21 Thr: 6 Tyr: 2

Serine predictions

Name Pos Context Score Pred

_________________________v_________________

48Glyma17g3 19 AEIMSRYRP 0.903 *S*

48Glyma17g3 31 KPETSPNSM 0.866 *S*

48Glyma17g3 34 TSPNSMSEG 0.997 *S*

48Glyma17g3 36 PNSMSEGPS 0.711 *S*

48Glyma17g3 40 SEGPSSSSL 0.906 *S*

48Glyma17g3 41 EGPSSSSLS 0.639 *S*

48Glyma17g3 45 SSSLSQKIK 0.969 *S*

48Glyma17g3 82 LPSSSLKRH 0.997 *S*

48Glyma17g3 108 VTSSSSSPS 0.773 *S*

48Glyma17g3 110 SSSSSPSKN 0.997 *S*

48Glyma17g3 112 SSSPSKNLS 0.621 *S*

48Glyma17g3 175 NTKASVPEE 0.969 *S*

48Glyma17g3 211 GSSISVVCI 0.502 *S*

48Glyma17g3 222 DSTLSPLAQ 0.859 *S*

48Glyma17g3 250 VISDSNHRI 0.962 *S*

48Glyma17g3 286 CRRISGEVT 0.996 *S*

48Glyma17g3 294 TLNLSDSST 0.714 *S*

48Glyma17g3 303 VIPTSSNGF 0.554 *S*

48Glyma17g3 317 IEWLSEHNN 0.888 *S*

48Glyma17g3 358 TREASQSSC 0.927 *S*

48Glyma17g3 360 EASQSSCNA 0.817 *S*

_________________________^_________________

Threonine predictions

Name Pos Context Score Pred

_________________________v_________________

48Glyma17g3 68 RPTRTRKRG 0.874 *T*

48Glyma17g3 88 KRHKTTHHH 0.537 *T*

48Glyma17g3 159 SPTLTTKPC 0.941 *T*

48Glyma17g3 227 PLAQTPKKP 0.989 *T*

48Glyma17g3 353 FHTRTTREA 0.967 *T*

48Glyma17g3 354 HTRTTREAS 0.909 *T*

_________________________^_________________

Tyrosine predictions

Name Pos Context Score Pred

_________________________v_________________

48Glyma17g3 53 KQSPYLRNL 0.508 *Y*

48Glyma17g3 261 ANSAYKEMV 0.824 *Y*

_________________________^_________________


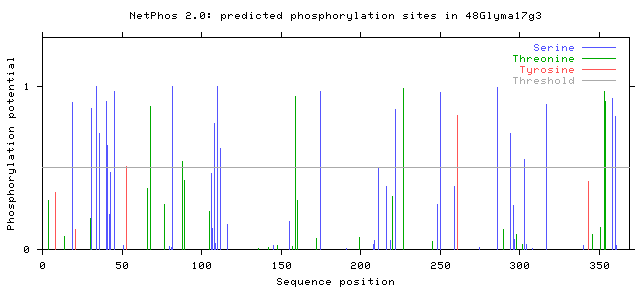


**GmSdr4L2** MIKTLNPYPNPAKTAEIMSRYRPIAPKPETSPNSSMSEGSCSSLSQKINQSPYLRNLWPQLQARPTRTRKRGRAPLTLPS 80

SSLKRHKTTHILGFCPPCHVVTSSPAKNLSFQGFAPLPNHGLGVLNCTMENNNTLTANPSLVTLPLLPCSPCPAPKLESN 160

STLTKPCVGEVIDLNTKVSVPEEKDLLQQLQKPVSSNINVITPQPVRPIGSSISVVCISEDLTLPPLSQTPKRPNEVEQE 240

VENEPLPAVISDSNHRIRMANSAYKEMVGQPLCPWLESMVNNNGGGECKRISGEVTLHLSDSTIVPTSSNGFSCWVRIEW 320

QSEHNNKKNCVNAFCDVMKLACESRDYLFTWRFHTRTTREASQSSCNA 400

..................S...........S..SS.S.......S.......Y..............T............ 80

.S.....T........................................................................ 160

..................S..................................S.............S.T.......... 240

............S..........Y...........................S..........T....S............ 320

.S..................................TT...S.S.... 400

Phosphorylation sites predicted: Ser: 16 Thr: 6 Tyr: 2

Serine predictions

Name Pos Context Score Pred

_________________________v_________________

49Glyma14g0 19 AEIMSRYRP 0.903 *S*

49Glyma14g0 31 KPETSPNSS 0.931 *S*

49Glyma14g0 34 TSPNSSMSE 0.772 *S*

49Glyma14g0 35 SPNSSMSEG 0.996 *S*

49Glyma14g0 37 NSSMSEGSC 0.905 *S*

49Glyma14g0 45 CSSLSQKIN 0.922 *S*

49Glyma14g0 82 LPSSSLKRH 0.997 *S*

49Glyma14g0 179 NTKVSVPEE 0.974 *S*

49Glyma14g0 214 GSSISVVCI 0.502 *S*

49Glyma14g0 228 LPPLSQTPK 0.922 *S*

49Glyma14g0 253 VISDSNHRI 0.758 *S*

49Glyma14g0 292 CKRISGEVT 0.995 *S*

49Glyma14g0 308 IVPTSSNGF 0.722 *S*

49Glyma14g0 322 IEWQSEHNN 0.767 *S*

49Glyma14g0 362 TREASQSSC 0.927 *S*

49Glyma14g0 364 EASQSSCNA 0.817 *S*

________________________^_________________

Threonine predictions

Name Pos Context Score Pred

_________________________v_________________

49Glyma14g0 68 RPTRTRKRG 0.874 *T*

49Glyma14g0 88 KRHKTTHIL 0.691 *T*

49Glyma14g0 230 PLSQTPKRP 0.972 *T*

49Glyma14g0 303 LSDSTIVPT 0.658 *T*

49Glyma14g0 357 FHTRTTREA 0.967 *T*

49Glyma14g0 358 HTRTTREAS 0.909 *T*

_________________________^_________________

Tyrosine predictions

Name Pos Context Score Pred

_________________________v_________________

49Glyma14g0 53 NQSPYLRNL 0.527 *Y*

49Glyma14g0 264 ANSAYKEMV 0.824 *Y*

_________________________^_________________


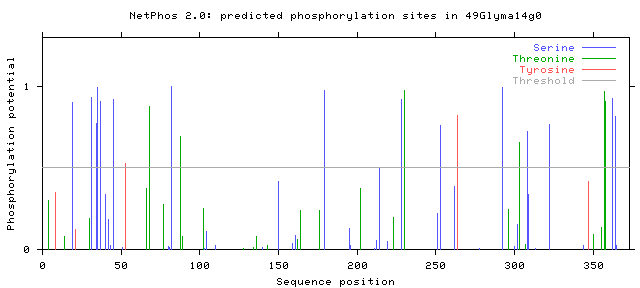


**MdSdr4L1** MIKTLNPYTNTEKTAEIMSRYRPIAPKPVTPSNPAGESPSMSQKIRDSPYLRNLWPQLQARPTRTRKRGRSALSPTTFKR 80

QRSHQMLGFATPCHVQSPVKNPRLDGFARVLPKLPIPXSLDAANNSTGXMTLPLLPYIPPASAPIVTNPAVVPPEFELIK 160

PCRGGEEELIDLNTVAAEIPEEKDLLKQLQGNSPAPPPTPTNVIAPQPIRPVGSSISVGCISADPSLTLAAQVPKKPEDV 240

EEEMESEALPAIISDSHNKVRMANSAYKEMVGQPECPWLDAMVAGGDGRFGGKRINGEVSLQLSDSGVPVSSNGFSCWVR 320

IEWGKDMNKHAINTFCDVMKLSCESKDYLFEWRFHTHSREGCQSSSSSA 400

..........T.......S..................S...S.....S.Y..............T.....S..S...... 80

..S.......T.....S............................................................... 160

......................................................S......................... 240

..........................Y..................................................... 320

.............T.......S.............T.S.....S..... 400

Phosphorylation sites predicted: Ser: 12 Thr: 5 Tyr: 2

Serine predictions

Name Pos Context Score Pred

_________________________v_________________

50Malusdome 19 AEIMSRYRP 0.903 *S*

50Malusdome 38 PAGESPSMS 0.624 *S*

50Malusdome 42 SPSMSQKIR 0.973 *S*

50Malusdome 48 KIRDSPYLR 0.846 *S*

50Malusdome 71 KRGRSALSP 0.981 *S*

50Malusdome 74 RSALSPTTF 0.985 *S*

50Malusdome 83 KRQRSHQML 0.967 *S*

50Malusdome 97 CHVQSPVKN 0.502 *S*

50Malusdome 215 PVGSSISVG 0.758 *S*

50Malusdome 342 VMKLSCESK 0.888 *S*

50Malusdome 358 FHTHSREGC 0.821 *S*

50Malusdome 364 EGCQSSSSS 0.593 *S*

_________________________^_________________

Threonine predictions

Name Pos Context Score Pred

_________________________v_________________

50Malusdome 11 PYTNTEKTA 0.882 *T*

50Malusdome 65 RPTRTRKRG 0.874 *T*

50Malusdome 91 LGFATPCHV 0.831 *T*

50Malusdome 334 HAINTFCDV 0.932 *T*

50Malusdome 356 WRFHTHSRE 0.698 *T*

_________________________^_________________

Tyrosine predictions

Name Pos Context Score Pred

_________________________v_________________

50Malusdome 50 RDSPYLRNL 0.877 *Y*

50Malusdome 267 ANSAYKEMV 0.824 *Y*

_________________________^_________________


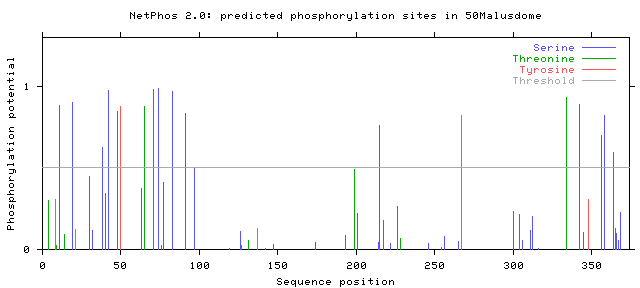


**MdSdr4L2** MLPFSDWSSLTLKNSNPSLNPPPHQPPXKSLPPSXTCLPALSLLPPYRRRHHRHYRTAGRPALAKRTAYRHNMIKTLNPY 80

TDAEKTAEIMSXYRPIAPQPEITANPAGXXPSMSQKIRDSSYLRNLWPXFQARPTRTRKRGRTALSPITFKRQRSHHMLG 160

FSTXCHVQSPVKNLRLDGFARALYGSFDTANTAALMTLPLLPYIPPSSVPIATNQAVVPAELELMKPCGGEENLIDLNIV 240

AAEIPEEKDLLKQLQGNSPAPPPTNNNVIEPQPIRLXGSNISVGCISADPSLAPAAQAPKKPDDVEEEMESKALPAVISD 320

SHKKVRIANSAYKEMVGQPECPWLXXIVAGDGRFGGKRISGEVSLQLSGSGVPVSSNGFSCWVRIEWGNEMNKHTINTFC 400

DVMKLSCESKDYLFEWRFHTHSREGCQSSRSSA 480

..........T......S...............S.............................................. 80

..........S......................S......SY..............T........S........S..... 160

................................................................................ 240

................................................................................ 320

S..........Y...........................S.....................................T.. 400

.....S.............T.S.....S...S. 480

Phosphorylation sites predicted: Ser: 13 Thr: 4 Tyr: 2

Serine predictions

Name Pos Context Score Pred

_________________________v_________________

51MalusdomM 18 NSNPSLNPP 0.752 *S*

51MalusdomM 34 SLPPSXTCL 0.953 *S*

51MalusdomM 91 AEIMSXYRP 0.896 *S*

51MalusdomM 114 XPSMSQKIR 0.908 *S*

51MalusdomM 121 IRDSSYLRN 0.989 *S*

51MalusdomM 146 RTALSPITF 0.944 *S*

51MalusdomM 155 KRQRSHHML 0.989 *S*

51MalusdomM 321 VISDSHKKV 0.974 *S*

51MalusdomM 360 GKRISGEVS 0.993 *S*

51MalusdomM 406 VMKLSCESK 0.888 *S*

51MalusdomM 422 FHTHSREGC 0.821 *S*

51MalusdomM 428 EGCQSSRSS 0.872 *S*

51MalusdomM 432 SSRSSA--- 0.936 *S*

_________________________^_________________

Threonine predictions

Name Pos Context Score Pred

_________________________v_________________

51MalusdomM 11 WSSLTLKNS 0.673 *T*

51MalusdomM 137 RPTRTRKRG 0.874 *T*

51MalusdomM 398 HTINTFCDV 0.919 *T*

51MalusdomM 420 WRFHTHSRE 0.698 *T*

_________________________^_________________

Tyrosine predictions

Name Pos Context Score Pred

_________________________v_________________

51MalusdomM 122 RDSSYLRNL 0.896 *Y*

51MalusdomM 332 ANSAYKEMV 0.824 *Y*

_________________________^_________________


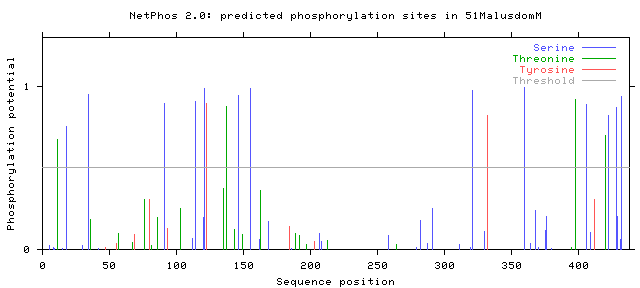


**MtSdr4L** MIKTLNPYPNPAKTAEIMSRYRPIAPKPETCSSNNSTSDGSSSSNSLSQKIKQSPYLRNLWPQLQARPTRTRKRGRAPIS 80

LPSSLKRQKTHVLGFCQPLHVTSPIKNLTLQGNFVPPSSLPQLPLPNHGVGVLNCNKNSTTNPNLVTLPLLPCSPNSPNN 160

NNALKFELEVIDLNNTKVEVPQERDLLQQLQKPASSTNNVINVISPQPIRPIGSCINVGCISEVSAIPCVTKTPKKPEEL 240

EQEVESEELPAVISDSNNRVRMANSAYKEMVGQPECPWLEIQCGSSCKRISGEVTLQLSDSSNIPISSNGFSCWVRIEWE 320

NNGQKKNCVNAFCDVVKLCCDQSRDYVFTWRFHTRSREASQSSCNA 400

..................S................S.S..SSS..S.S.......Y..............T......... 80

...S.....T............S.....................................................S... 160

...............T..................S.....................................T....... 240

.....S....................Y..................S....S............................. 320

.........................Y.......T.S...S.S.... 400

Phosphorylation sites predicted: Ser: 18 Thr: 5 Tyr: 3

Serine predictions

Name Pos Context Score Pred

_________________________v_________________

52Medicago 19 AEIMSRYRP 0.903 *S*

52Medicago 36 SSNNSTSDG 0.994 *S*

52Medicago 38 NNSTSDGSS 0.957 *S*

52Medicago 41 TSDGSSSSN 0.959 *S*

52Medicago 42 SDGSSSSNS 0.989 *S*

52Medicago 43 DGSSSSNSL 0.515 *S*

52Medicago 46 SSSNSLSQK 0.824 *S*

52Medicago 48 SNSLSQKIK 0.700 *S*

52Medicago 84 SLPSSLKRQ 0.997 *S*

52Medicago 103 LHVTSPIKN 0.825 *S*

52Medicago 157 CSPNSPNNN 0.670 *S*

52Medicago 195 QKPASSTNN 0.954 *S*

52Medicago 246 QEVESEELP 0.956 *S*

52Medicago 286 QCGSSCKRI 0.981 *S*

52Medicago 291 CKRISGEVT 0.996 *S*

52Medicago 356 FHTRSREAS 0.949 *S*

52Medicago 360 SREASQSSC 0.943 *S*

52Medicago 362 EASQSSCNA 0.817 *S*

_________________________^_________________

Threonine predictions

Name Pos Context Score Pred

_________________________v_________________

52Medicago 71 RPTRTRKRG 0.874 *T*

52Medicago 90 KRQKTHVLG 0.669 *T*

52Medicago 176 DLNNTKVEV 0.782 *T*

52Medicago 233 CVTKTPKKP 0.956 *T*

52Medicago 354 WRFHTRSRE 0.624 *T*

_________________________^_________________

Tyrosine predictions

Name Pos Context Score Pred

_________________________v_________________

52Medicago 56 KQSPYLRNL 0.508 *Y*

52Medicago 267 ANSAYKEMV 0.824 *Y*

52Medicago 346 QSRDYVFTW 0.549 *Y*

_________________________^_________________


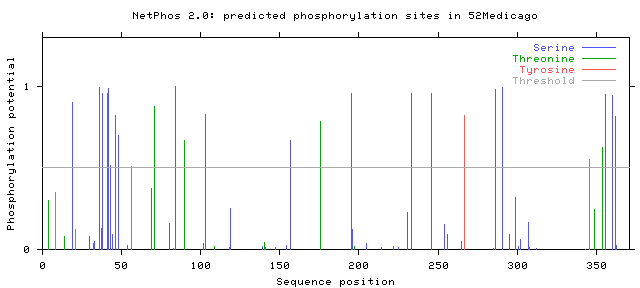


**PhvSdr4L** MIKTLNPYPNPAKTAEIMSRYRPIAPKPETSPNSMTEGSSSLSHKIKQSPYLRNLWPQLQARPTRTRKRGRAPLTLPPSS 80

FKRHKTHILGFCPPCHVTSPGNNLSFQGFAPPPLPLPHPNHGLGMLNRSIEKNSLMNPSLVTLPLLPCSPGAPAPKLDSI 160

TATTTTKPCGGGVIDLNTKASVPEERDLLQQLQKPVSNNVIQPHPVRPIGSSITVVCIGEDSTLPSQTPKRSQEVEAEVE 240

SESLPVVISDSNHRVRMANSAYKEMVGQPLCPWLESMVNAGAGNLQCKRISGEVALHLSESNIPTSSNGFSCWVRIEWQS 320

DQQKKCCVNAFCDVMKLTCESRDYLFTWRFHTRTREACQSSCTA 400

..................S...........S..S.....SS.S.......Y..............T.............S 80

..................S.............................S....S........................S. 160

...TT...............S............................................S.T...S........ 240

.....................Y............................S..............S.............. 320

...............................T............ 400

Phosphorylation sites predicted: Ser: 16 Thr: 5 Tyr: 2

Serine predictions

Name Pos Context Score Pred

_________________________v_________________

53Phaseolus 19 AEIMSRYRP 0.903 *S*

53Phaseolus 31 KPETSPNSM 0.853 *S*

53Phaseolus 34 TSPNSMTEG 0.996 *S*

53Phaseolus 40 TEGSSSLSH 0.777 *S*

53Phaseolus 41 EGSSSLSHK 0.783 *S*

53Phaseolus 43 SSSLSHKIK 0.956 *S*

53Phaseolus 80 LPPSSFKRH 0.997 *S*

53Phaseolus 99 CHVTSPGNN 0.922 *S*

53Phaseolus 129 MLNRSIEKN 0.911 *S*

53Phaseolus 134 IEKNSLMNP 0.673 *S*

53Phaseolus 159 PKLDSITAT 0.773 *S*

53Phaseolus 181 NTKASVPEE 0.974 *S*

53Phaseolus 226 STLPSQTPK 0.561 *S*

53Phaseolus 232 TPKRSQEVE 0.932 *S*

53Phaseolus 291 CKRISGEVA 0.984 *S*

53Phaseolus 306 NIPTSSNGF 0.508 *S*

_________________________^_________________

Threonine predictions

Name Pos Context Score Pred

_________________________v_________________

53Phaseolus 66 RPTRTRKRG 0.874 *T*

53Phaseolus 164 ITATTTTKP 0.716 *T*

53Phaseolus 165 TATTTTKPC 0.921 *T*

53Phaseolus 228 LPSQTPKRS 0.983 *T*

53Phaseolus 352 WRFHTRTRE 0.526 *T*

_________________________^_________________

Tyrosine predictions

Name Pos Context Score Pred

_________________________v_________________

53Phaseolus 51 KQSPYLRNL 0.508 *Y*

53Phaseolus 262 ANSAYKEMV 0.824 *Y*

_________________________^_________________


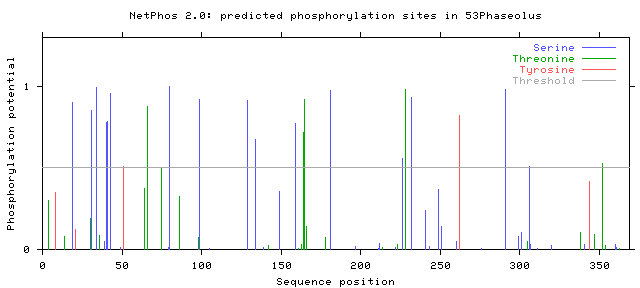


**PpSdr4L** MIKTLNPYNSNTEKTAEIMSRYRPIAPKPETPANSAGENPSLSQKIRESPYLRNLWPQLQARPTRTRKRGRSALSPTTFK 80

RQRTHHVFGFSTPCHVTSPAKNLTLDGFAHALSQLPIPTSFDAVKSTSLMTLPLLQYPPPPPSPPPPPPTSSVPVVTNQA 160

MVPAEFELIKPCEGEEKLIDLNTVAEIPEEKDLLQQLQGSAPTPTTINVIAPQPVRPVGSSISVGCIKTDPSLAPAEQAP 240

KKPEDVEDEVESEALPAIISDSHNKVRMANSAYKEMVGQPECSWLDSMVASDGRFGGSSCKRISGEVILELSESGVPVSS 320

NGFSCWVRIEWGNEMNKHAINAFCDVIKLSCETKDYLFSWRFHTHSKEGSQSSSSNA 400

...........T.......S..........T...S.......S.....S.Y..............T.....S..S..... 80

...T.......T..........................T.......................S......T.......... 160

............................................................S..........S........ 240

................................Y.........S...............S....S................ 320

.............................S........S....T.S...S.SS.... 400

Phosphorylation sites predicted: Ser: 18 Thr: 8 Tyr: 2

Serine predictions

Name Pos Context Score Pred

_________________________v_________________

54Prunusper 20 AEIMSRYRP 0.903 *S*

54Prunusper 35 TPANSAGEN 0.988 *S*

54Prunusper 43 NPSLSQKIR 0.957 *S*

54Prunusper 49 KIRESPYLR 0.756 *S*

54Prunusper 72 KRGRSALSP 0.981 *S*

54Prunusper 75 RSALSPTTF 0.985 *S*

54Prunusper 143 PPPPSPPPP 0.888 *S*

54Prunusper 221 PVGSSISVG 0.758 *S*

54Prunusper 232 KTDPSLAPA 0.747 *S*

54Prunusper 283 QPECSWLDS 0.940 *S*

54Prunusper 299 FGGSSCKRI 0.965 *S*

54Prunusper 304 CKRISGEVI 0.992 *S*

54Prunusper 350 VIKLSCETK 0.779 *S*

54Prunusper 359 DYLFSWRFH 0.509 *S*

54Prunusper 366 FHTHSKEGS 0.982 *S*

54Prunusper 370 SKEGSQSSS 0.671 *S*

54Prunusper 372 EGSQSSSSN 0.889 *S*

54Prunusper 373 GSQSSSSNA 0.826 *S*

_________________________^_________________

Threonine predictions

Name Pos Context Score Pred

_________________________v_________________

54Prunusper 12 YNSNTEKTA 0.517 *T*

54Prunusper 31 PKPETPANS 0.559 *T*

54Prunusper 66 RPTRTRKRG 0.874 *T*

54Prunusper 84 KRQRTHHVF 0.791 *T*

54Prunusper 92 FGFSTPCHV 0.919 *T*

54Prunusper 119 LPIPTSFDA 0.729 *T*

54Prunusper 150 PPPPTSSVP 0.651 *T*

54Prunusper 364 WRFHTHSKE 0.734 *T*

_________________________^_________________

Tyrosine predictions

Name Pos Context Score Pred

_________________________v_________________

54Prunusper 51 RESPYLRNL 0.951 *Y*

54Prunusper 273 ANSAYKEMV 0.824 *Y*

_________________________^_________________


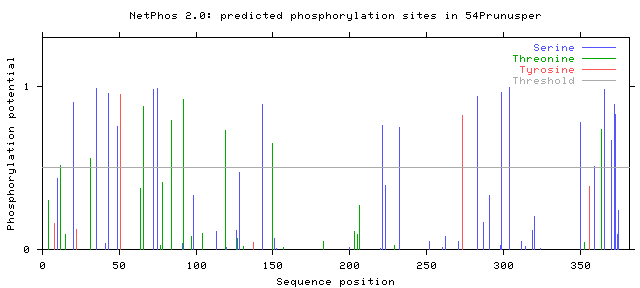

Supplement: S2 File — (DOCX) [file pone.0153717.s006.docx]
