# Supplementary material for: Phylogenetic Analysis, Lineage-Specific Expansion and Functional Divergence of seed dormancy 4-Like Genes in Plants
Source: PLoS One. 2016 Jun 14;11(6):e0153717. doi: 10.1371/journal.pone.0153717 (PMC4907471; doi:10.1371/journal.pone.0153717)
Supplement: S3 File — (PDF) [file pone.0153717.s007.pdf]

[Home] [Server] [Queue] [About] [Remove] [Statistics]

I-TASSER results for job id S205397

[Click on S205397_results.tar.bz2 to download the tarball file including all modeling results listed on this page]

Submitted Sequence in FASTA format

```
>Rice OsSdr4L protein
MAMVQPVDMAVKANEIMARFRPIAPKPVLPAAAAGVTGGGDGAAAVAATNRVLCQLQSRP
CRARKRGRPSVVPPVSPPAGAKRKRAPAYPVPVAPLRCAAVATATRARVSVVVVPAPESA
GGVSALAPVSPSAGDSTRLSPTVVEVEDEDEERGVVLVERDLLRKLLEPRKLLEPRAVRP
VGSTIHVESVHIDVGRTTAAAAAAAPKTAEEVEAELESDSLPAVVSDSSNRVRLVNDAYK
RMVGQPECPWLDAVATAASRRISGEVALVVSEPAAAAAALPETCKGFSCSAKIAWERDGK
WSSVHAPCDVTRLQCESRDYVFAWRFRAAGDECNTHRRAAGDA
```

Predicted Secondary Structure

```
                     20                  40                  60                  80                  100                 120                 140
                      |                   |                   |                   |                   |                   |                   |
Sequence   MAMVQPVDMAVKANEIMARFRPIAPKPVLPAAAAGVTGGGDGAAAVAATNRVLCQLQSRPCRARKRGRPSVVPPVSPPAGAKRKRAPAYPVPVAPLRCAAVATATRARVSVVVVPAPESAGGVSALAPVSPSAGDSTRLSPTVVEVEDEDEER
Prediction CCCCCCCCCHHHHHHHHHHCCCCCCCCCCCCCCCCCCCCCCCCCCCCCCCCCC SSCCCCCCCCCCCCCCCCCCCCCCCCCCCCCCCCCCCCCCCCCCCCCCCCCCCCCCCCCCCCCCCCCCCCCCCCCCCCCCCCCCCCCCCCCCCCCCCCCC
Conf.Score 9614667634889999985278889998998877766766554455300000210136666556577777787677876544454444577687864343223665564233457776655564454668887566544455665567544567
           H:Helix; S:Strand; C:Coil
```

Predicted Solvent Accessibility

```
                     20                  40                  60                  80                  100                 120                 140
                      |                   |                   |                   |                   |                   |                   |
Sequence   MAMVQPVDMAVKANEIMARFRPIAPKPVLPAAAAGVTGGGDGAAAVAATNRVLCQLQSRPCRARKRGRPSVVPPVSPPAGAKRKRAPAYPVPVAPLRCAAVATATRARVSVVVVPAPESAGGVSALAPVSPSAGDSTRLSPTVVEVEDEDEER
Prediction 6431422734230230034021102214334334444444554444333333244343333433444445344444465644444453463634434444344233532434524346355344423413144642642443344145345644
           Values range from 0 (buried residue) to 9 (highly exposed residue)
```

Predicted normalized B-factor

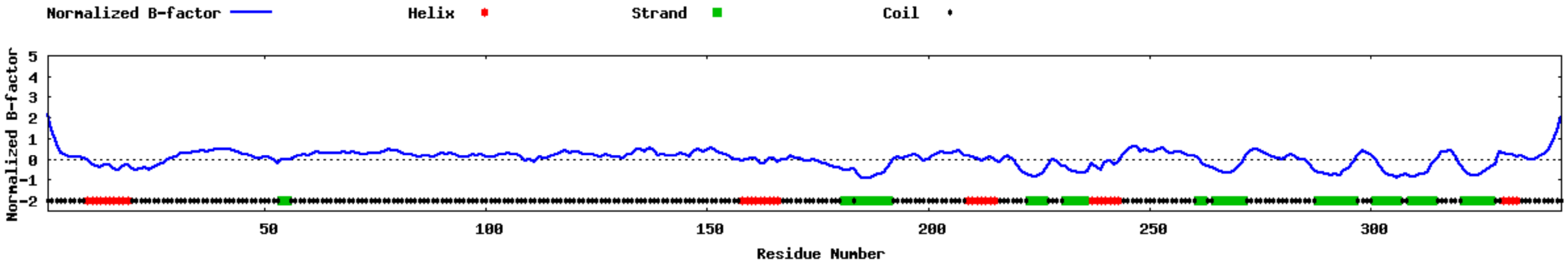

Read more about predicted normalized B-factor

Top 10 templates used by I-TASSER

Rank	PDB Hit	Iden1	Iden2	Cov.	Norm. Z-score	Download Align.	20 40 60 80 100 120
						Sec.Str	CCCCCCCCCHHHHHHHHHHCCCCCCCCCCCCCCCCCCCCCCCCCCCCCCCCCCCSSCCCCCCCCCCCCCCCCCCCCCCCCCCCCCCCCCCCCCCCCCCCCCCCCCCCCCCCCCCCCCCCCCCCCC
						Seq	MAMVQPVDMAVKANEIMARFRPIAPKPVLPAAAAGVTGGGDGAAAVAATNRVLCQLQSRPCRARKRGRPSVVPPVSPPAGAKRKRAPAYPVPVAPLRCAAVATATRARVSVVVVPAPESAGGVSALAPVS
1	4bmlA	0.06	0.22	0.87	1.62	Download	--ALKLFSGEV--------FTAFNNASIFKGLVRSYDLRGGKSKQFMFTGKLSAGYHTPGTPIVGDAGIKANEKTLVMDDLLVSSQFVYSLDEIFSQYSTRAEVSKQIGEALATHYDERIARVLAKASAE
2	1maeH	0.07	0.21	0.98	1.62	Download	----SSASAAAAAAAAALAAGAADGPTNDEAPGADGRRSYINLPAHHSAIIQQWVLDAGSGSILGHVNGGFLPNPVAAHSGSEFALASTSFSRIAKGKRTDYVEVFDPVTFLPIADIELPDAPRFDVGPY
3	1dgiR	0.09	0.16	0.87	1.55	Download	VVVQAPTQVPGFLDSVTLPCYLQVPNMEVTHVSQLTWARHGESGSMAVFHQTQGPSYSESKRLEFVAARLGAELRNASLR-------MFGLRVEDEGNYTCLFVTFPQGSRSVDIWLRVLAKPQNTAEVQ
4	1w0rA	0.08	0.19	0.96	1.45	Download	SVEDCCLNTAFAYQKRSGGLCQPCRSPRWSLVGWNGQCSGKVAPGTLEWQLQACEDQQCCPEMGGWSGWGPWEPCSVTCSKGTRTRRRACNHPAPKCGGHCPGQAQESEACDTQQVCPTHGAWATWGPWT
5	2ocwA	0.07	0.22	1.00	1.50	Download	SRGLSFDVSLEVSQGPGLLNDTKVYTVDLGRTVTINCPFKTENAQKQIGLYPVLVIDSSGYVNPNYTGRIRLDIQGTGGDDSNSNKKNADLQVLKPEPELVYEDLRGSVTFHCALGPEVANVAKFLCRQS
6	1mv3A	0.09	0.17	0.59	1.45	Download	DGSPAAT--------------PEIRVNHEPEPAGGATPGATLPKSPSQLRKGPPVPPPPKHTPSKEVKQEQILSLFEDTFVPEISVTTPSQPAEASEVAGGTQPAAGAQEPGETAASEAASSSLPAVVVE
7	3j65R	0.08	0.17	0.81	1.45	Download	--SSTQDSKAQTLNSNPEILLRKRRNADRTRIERQELAKKKREEQIKKKRSNKNKFVRAESIVAKTLATSREKE
8	3b7fA	0.09	0.20	0.95	1.44	Download	SAPESGPVLLVATIKGAWFLALRGPVFLGHTIHHIVQDPREPERLAARTLGPTVFRSDDGGGNWTEATRPPAFNKAPGRVVDHVFWLTPGHASEPGTWYAGTDHGASWEPVAGFNDHPRRAWTGGEPDGE
9	3cm9S	0.09	0.22	1.00	1.37	Download	-INSRGLSFDVSLEVSQGPGLLNDTKVYTVDLGRTVTINCPFKTENAQKRKSLYKQIGLYPVLVIDSSGYVNPNYTQAGDDSNSNKKNADLQVLKPEPELVYEDLRGSVTFHCALGPEVANVAKFLCRQS
10	1tzlA	0.09	0.21	1.00	1.42	Download	DIKYSGPIGCTYARELVGAGYKVAFDIGEIDSGLKIGAHKKNTVEYQKNIDKFVNVIQGQLSVSVPVNTLVVDTLSPTSWQASTFFVRNGSNPEQDPLRNLSGQAVTRVVGGSTHWTCATPRFDREQRPI

(a) All the residues are colored in black; however, those residues in template which are identical to the residue in the query sequence are highlighted in color. Coloring scheme is based on the property of amino acids, where polar are brightly coloured while non-polar residues are colored in dark shade. (more about the colors used)

(b) Rank of templates represents the top ten threading templates used by I-TASSER.

(c) Ident1 is the percentage sequence identity of the templates in the threading aligned region with the query sequence.

(d) Ident2 is the percentage sequence identity of the whole template chains with query sequence.

(e) Cov. represents the coverage of the threading alignment and is equal to the number of aligned residues divided by the length of query protein.

(f) Norm. Z-score is the normalized Z-score of the threading alignments. Alignment with a Normalized Z-score >1 mean a good alignment and vice versa.

(g) Download Align. provides the 3D structure of the aligned regions of the threading templates.

(h) The top 10 alignments reported above (in order of their ranking) are from the following threading programs:

1: Neff-PPAS 2: PROSPECT2 3: Neff-PPAS 4: PROSPECT2 5: Neff-PPAS 6: PROSPECT2 7: Neff-PPAS 8: PROSPECT2 9: Neff-PPAS 10: PROSPECT2

Top 5 Models predicted by I-TASSER

Generated 3D models Estimated local accuracy of models

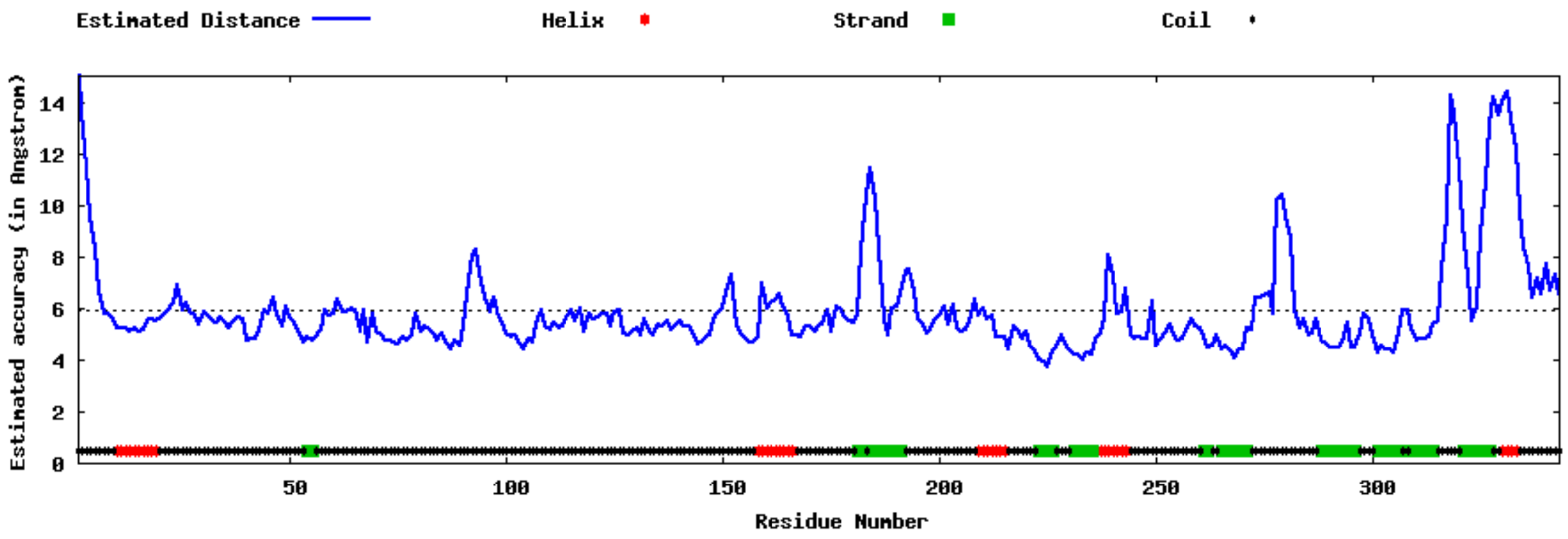

- Download Model 1
- C-score=-2.67 (Read more about C-score)
- Estimated TM-score = 0.41±0.14
- Estimated RMSD = 12.9±4.2Å

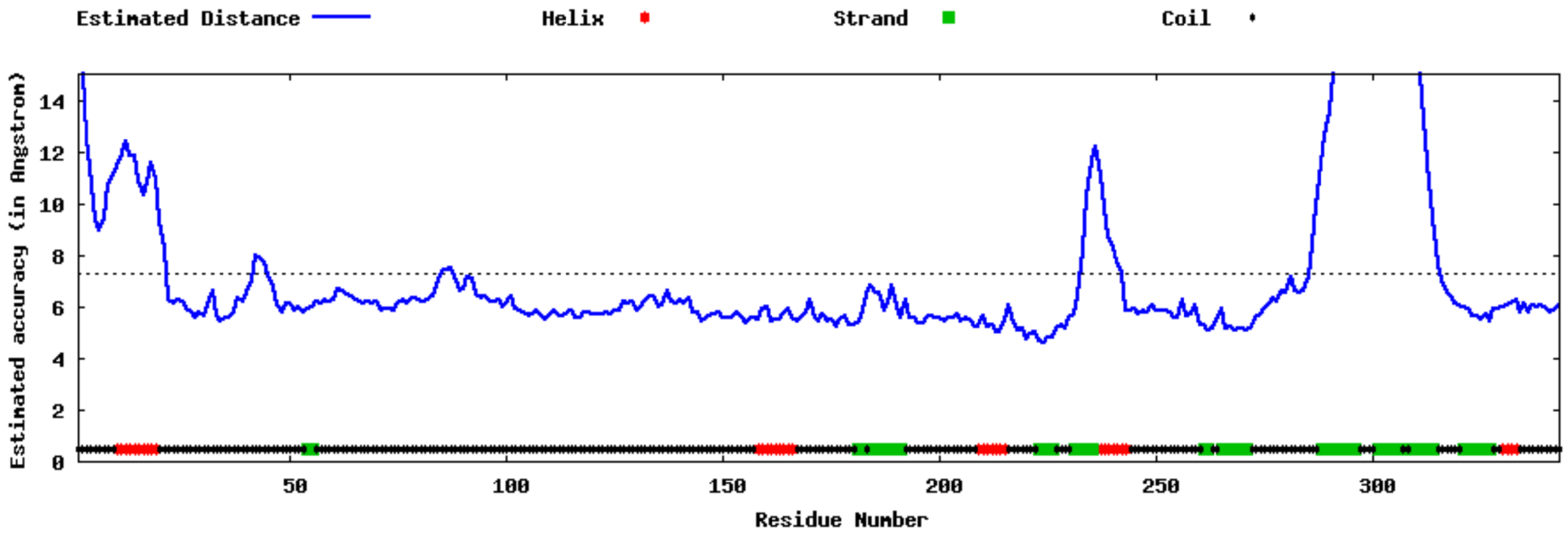

- Download Model 2
- C-score = -3.83

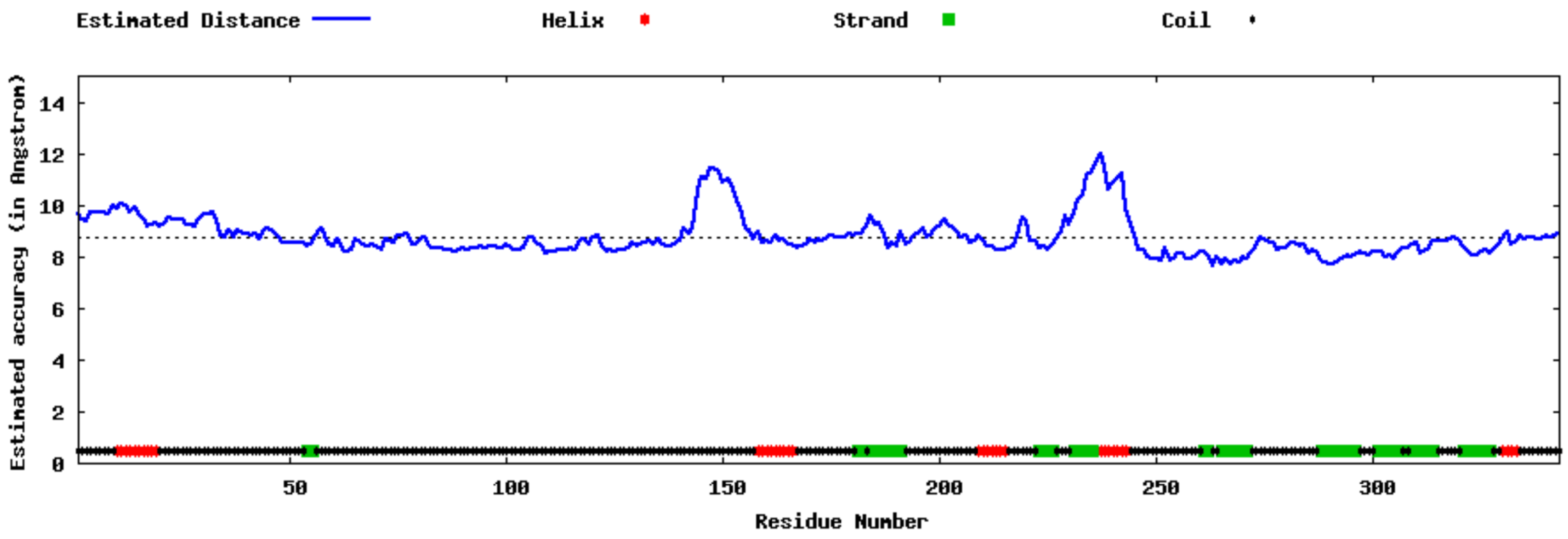

- Download Model 3
- C-score = -2.39

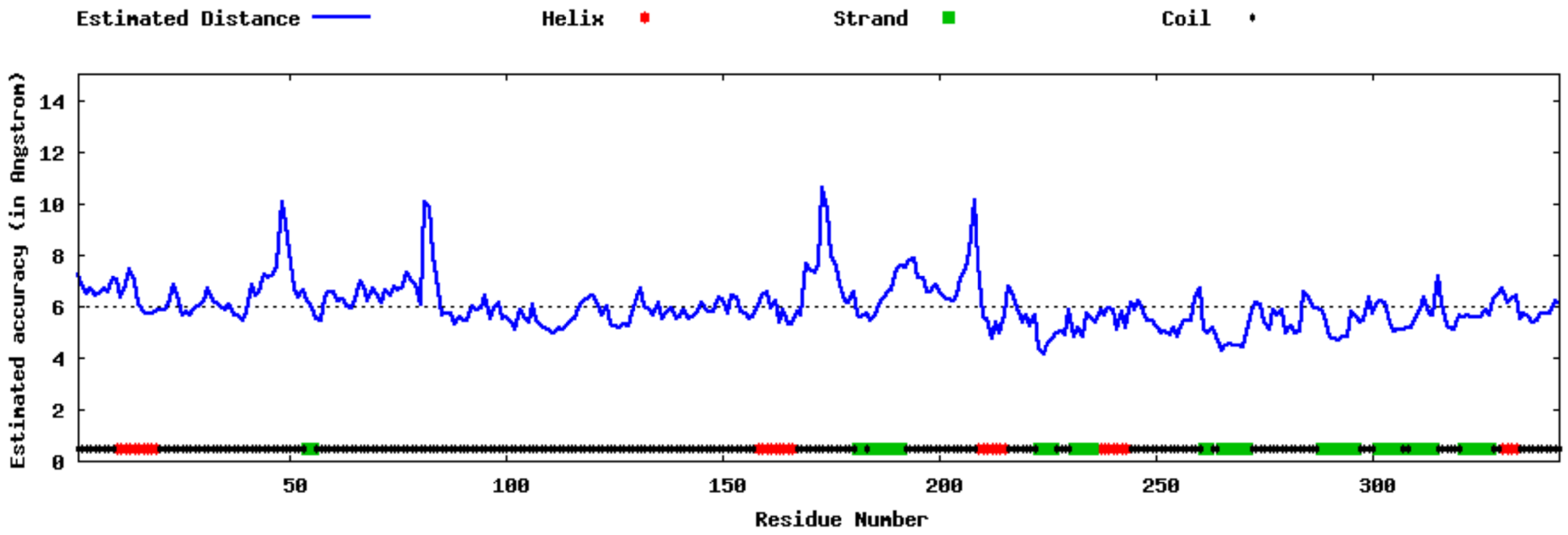

- Download Model 4
- C-score = -2.75

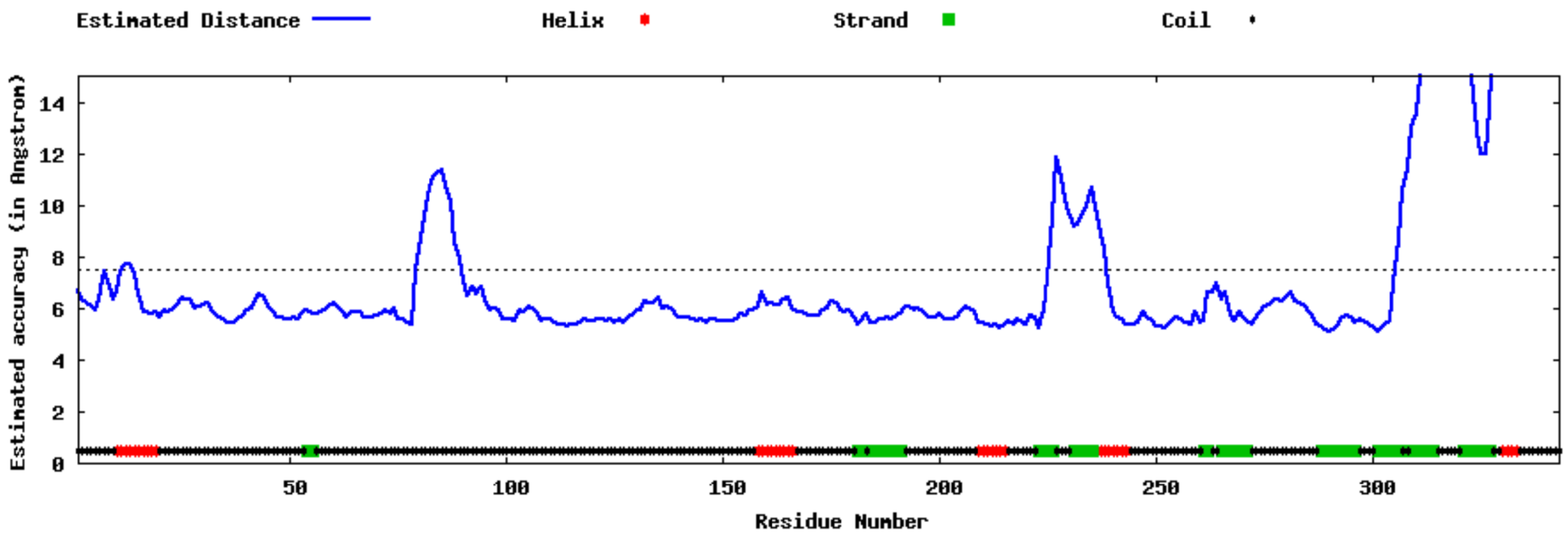

- Download Model 5
- C-score = -4.26

Proteins structurally close to the target in the PDB (as identified by TM-align)

Top 10 Identified stuctural analogs in PDB

Click to view	Rank	PDB Hit	TM-score	RMSD[a]	IDEN[a]	Cov.	Download Alignment
◉	1	2gc4E	0.947	1.73	0.073	0.994	Download
○	2	1maeH	0.940	1.64	0.071	0.983	Download
○	3	3c75J	0.937	1.74	0.062	0.985	Download
○	4	1mdaH	0.929	1.69	0.054	0.974	Download
○	5	1madH	0.928	1.70	0.072	0.974	Download
○	6	2hkmB	0.815	2.62	0.064	0.915	Download
○	7	1jjuB	0.661	3.23	0.086	0.781	Download
○	8	1qniF	0.659	3.70	0.072	0.813	Download
○	9	1jmzB	0.658	3.64	0.076	0.808	Download
○	10	2eceA	0.656	3.91	0.050	0.825	Download

☐ Spin On/Off

(a) Query structure is shown in cartoon, while the structural analog is displayed using backbone trace.
(b) Ranking of proteins is based on TM-score of the structural alignment between the query structure and known structures in the PDB library.
(c) RMSD[a] is the RMSD between residues that are structurally aligned by TM-align.
(d) IDEN[a] is the percentage sequence identity in the structurally aligned region.
(e) Cov. represents the coverage of the alignment by TM-align and is equal to the number of structurally aligned residues divided by length of the query protein.

Function Prediction using COFACTOR

Predicted EC Numbers

Top 5 enzyme homologs in PDB

Click to view	Rank	$Cscore^{EC}$	PDB Hit	TM-score	$RMSD^a$	$IDEN^a$	Cov.	EC Number	Predicted Active Site Residues
○	1	0.195	2gc7A	0.947	1.74	0.073	0.994	1.4.99.3	NA
○	2	0.192	1madH	0.928	1.70	0.072	0.974	1.4.99.3	NA
○	3	0.192	2madH	0.939	1.65	0.071	0.983	1.4.99.3	NA
○	4	0.189	3c75J	0.937	1.74	0.062	0.985	1.4.99.3	NA
○	5	0.167	2h47H	0.815	2.69	0.063	0.918	1.4.99.4	NA

Click on the radio buttons to visualize predicted active site residues.

(a) $Cscore^{EC}$ is the confidence score for the Enzyme Classification (EC) number prediction. $Cscore^{EC}$ values range in between [0-1]; where a higher score indicates a mc reliable EC number prediction.

(b) TM-score is a measure of global structural similarity between query and template protein.

(c) $RMSD^a$ is the RMSD between residues that are structurally aligned by TM-align.

(d) $IDEN^a$ is the percentage sequence identity in the structurally aligned region.

(e) Cov. represents the coverage of global structural alignment and is equal to the number of structurally aligned residues divided by length of the query protein.

☐ Spin On/Off

Predicted GO terms

Rank	$Cscore^{GO}$	TMscore	$RMSD^a$	$IDEN^a$	Cov.	PDB Hit	Associated GO Terms
1	0.19	0.9453	1.76	0.08	0.99	1mg2A	GO:0055114 GO:0006810 GO:0022900 GO:0016491 GO:0042597 GO:0030058 GO:0005515 GO:0030416
2	0.19	0.9408	1.63	0.07	0.98	1maeH	GO:0030416 GO:0042597 GO:0005515 GO:0055114 GO:0030058
3	0.19	0.9374	1.74	0.06	0.99	3c75H	GO:0055114 GO:0042597 GO:0030058 GO:0030416 GO:0005515
4	0.19	0.9286	1.69	0.05	0.97	1mdaH	GO:0005515 GO:0030058 GO:0030416 GO:0042597 GO:0055114
5	0.16	0.8159	2.60	0.06	0.92	2h47F	GO:0022900 GO:0006810 GO:0030058 GO:0030059 GO:0042597 GO:0005515 GO:0055114 GO:0030416 GO:0016491
6	0.14	0.6612	3.23	0.09	0.78	1jjuB	GO:0005515
7	0.14	0.6505	4.06	0.05	0.83	1ri6A	GO:0017057 GO:0005975 GO:0006006 GO:0016787 GO:0005515 GO:0006098
8	0.14	0.6390	4.36	0.09	0.84	1jofA	GO:0016853 GO:0047768 GO:0005515
9	0.14	0.6455	4.01	0.07	0.82	3hfqA	GO:0005515
10	0.14	0.6579	3.72	0.07	0.81	1qniA	GO:0046872 GO:0016020 GO:0005515 GO:0004129 GO:0005507

Consensus Prediction of Gene Ontology terms

Molecular Function		Biological Process		Cellular Location	
GO term	GO-Score	GO term	GO-Score	GO term	GO-Score
GO:0005515	0.64	GO:0030416	0.64	GO:0042597	0.64

GO:0030058	0.64	GO:0006810	0.33
		GO:0022900	0.33

(a) Cscore^{GO}, which is a combined measure for evaluating global and local similarity between query and template protein. Cscore^{GO} values range in between [0-1]; where a higher value indicates a better confidence in predicting the function using the templa

(b) TM-score is a measure of global structural similarity between query and template protein.

(c) RMSD^a is the RMSD between residues that are structurally aligned by TM-align.

(d) IDEN^a is the percentage sequence identity in the structurally aligned region.

(e) Cov. represents the coverage of global structural alignment and is equal to the number of structurally aligned residues divided by length of the query protein.

(f) The second table shows a consistence of function (GO terms) amongst top scoring templates. The GO-Score associated with each prediction is defined as the average weight o f the GO term, where the weights are assigned based on Cscore ^{GO} of template from which the GO term is derived.

Predicted Binding Site

Template proteins with similar binding site:

Click to view	Rank	Cscore^{LB}	PDB Hit	TM-score	RMSD^a	IDEN^a	Cov.	BS-score	Lig. Name	Download Complex	Predicted binding site residues
○	1	0.01	1jofH	0.649	4.32	0.079	0.846	0.22	PIN	Download	285,286,312,314,323
○	2	0.01	1gq10	0.633	4.19	0.065	0.810	0.26	PEPTIDE	Download	191,193,194,195,196,197,242,243

Click on the radio buttons to visualize predicted binding site and residues.

(a) Cscore^{LB} is the confidence score of predicted binding site. Cscore ^{LB} values range in between [0-1]; where a higher score indicates a more reliable ligand-binding site prediction.

(b) BS-score is a measure of local similarity (sequence & structure) between template binding site and predicted binding site in the query structure. Based on large scale benchmarking analysis, we have observed that a BS-score >1 reflects a significant local match between the predicted and template binding site.

(c) TM-score is a measure of global structural similarity between query and template protein.

(d) RMSD^a the RMSD between residues that are structurally aligned by TM-align.

(e) IDEN^a is the percentage sequence identity in the structurally aligned region.

(f) Cov. represents the coverage of global structural alignment and is equal to the number of structurally aligned residues divided by length of the query protein.

☐ Spin On/Off

[Click on S205397_results.tar.bz2 to download the tarball file including all modeling results listed on this page]

Please cite following articles when you use the I-TASSER server:

1. Yang Zhang. I-TASSER server for protein 3D structure prediction. BMC Bioinformatics, 9:40 (2008).
2. Ambrish Roy, Alper Kucukural, Yang Zhang. I-TASSER: a unified platform for automated protein structure and function prediction. Nature Protocols, vol 5, 725-738 (2010).

3. Ambrish Roy, Jianyi Yang, Yang Zhang. COFACTOR: an accurate comparative algorithm for structure-based protein function annotation. Nucleic Acids Research, vol 40, W471-W4 77 (2012).

[Home] [Server] [Queue] [About] [Remove] [Statistics]

I-TASSER results for job id S206754

[Click on S206754_results.tar.bz2 to download the tarball file including all modeling results listed on this page]

Submitted sequence in FASTA format

> SlSdr4L Protein
MMQTLNPYPSTSKTAEIMARYRPIAPKPEAPTSPVSEDNPTGLPPNIQKSPFLRNVWPQLQARPTRTRKRGRTALGPPSMKRARGNYFPAGQFPNYHQVVAASPSYRPNVVPQFTLIPNLLPLKCGLGTSVTTPSNSITLPLMACTTTTLPMLVEKHSGEEIRGI
DLNLAADGPEELDFMPQLQGPKTPGPVVITPQPVRPVGSSISIGCINEEEAPDGGATNKKFIKKPEEVEEEVEAEALPAVVSDSNNKVRLTNAAYKEMVGQPECCWLDYMVGNACKRIGGEVILEFLDSSCSVPMSSDGFNCWVKIEWGAAQGKKNSVKAFCNAV
KLACQSKDYVFEWRFHTTDDNTPESAASNI

Predicted secondary structure

```
                    20                  40                  60                  80                 100                 120                 140
                     |                   |                   |                   |                   |                   |                   |
Sequence   MMQTLNPYPSTSKTAEIMARYRPIAPKPEAPTSPVSEDNPTGLPPNIQKSPFLRNVWPQLQARPTRTRKRGRTALGPPSMKRARGNYFPAGQFPNYHQVVAASPSYRPNVVPQFTLIPNLLPLKCGLGTSVTTPSNSITLPLMACTTTTLPML
Prediction CCCCCCCCCCHHHHHHHHHHCCCCCCCCCCCCCCCCCCCCCCCCCCCCCC HHHHHCCCCCCCCCCCCCCCCCCCCCCCCCCCCCCCCCCCCCCCCCCCCCCCCCCCCCCCCCCCCCCCCCCCCCCCCCCCCCCCCCCCCC SSSCCCCCCCCCCCC
Conf.Score 9532368873378999997347889899999888777787766422101252332013011356654444677876764334566564567888865544456755455666666666666653445455655445611202687886667643
           H:Helix; S:Strand; C:Coil
```

Predicted solvent accessibility

```
                    20                  40                  60                  80                 100                 120                 140
                     |                   |                   |                   |                   |                   |                   |
Sequence   MMQTLNPYPSTSKTAEIMARYRPIAPKPEAPTSPVSEDNPTGLPPNIQKSPFLRNVWPQLQARPTRTRKRGRTALGPPSMKRARGNYFPAGQFPNYHQVVAASPSYRPNVVPQFTLIPNLLPLKCGLGTSVTTPSNSITLPLMACTTTTLPML
Prediction 623213425331203200331200012143444444444444443444344333333214324433443444344433464343444444354252454464445455444534444333342343634364444334301202113243651543
           Values range from 0 (buried residue) to 9 (highly exposed residue)
```

Predicted normalized B-factor

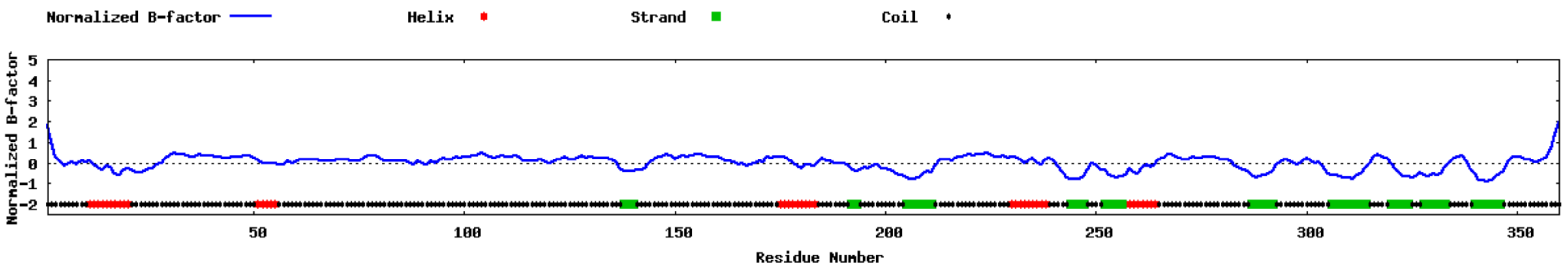

Read more about predicted normalized B-factor

Top 10 templates used by I-TASSER

Rank	PDB Hit	Iden1	Iden2	Cov	Norm. Z-score	Download Align.	20 40 60 80 100 120
						**Sec.Str**	CCCCCCCCCCHHHHHHHHHHCCCCCCCCCCCCCCCCCCCCCCCCCCCCCCHHHHHCCCCCCCCCCCCCCCCCCCCCCCCCCCCCCCCCCCCCCCCCCCCCCCCCCCCCCCCCCCCCCCCCCCCCCCCCC
						**Seq**	MMQTLNPYPSTSKTAEIMARYRPIAPKPEAPTSPVSEDNPTGLPPNIQKSPFLRNVWPQLQARPTRTRKRGRTALGPPSMKRARGNYFPAGQFPNYHQVVAASPSYRPNVVPQFTLIPNLLPLKCGLGTS
1	3j65R	0.06	0.17	0.87	1.87	Download	--------------------------------SSTQDSKAQTLNSNPEILLRKRRNADRTRIERQELAKKKREEQIKKKRSNKNKFVRAESIVAKTLATSREKERIKRVSILEDKKAKNETQHIASGKDF
2	1maeH	0.07	0.19	0.92	1.78	Download	-----SSASAAAAAAAAALAAGAADGPTNDEAPGADGRRSYINLPAHHSAIIQQWVLDAGSGSILGHVNGGFLPNPVAAHSGSEFALASTSFSRIAKGKRTDYDPVTFLPIADIELPDAPRFDVGPYSWM
3	4bmlA	0.05	0.16	0.84	1.84	Download	-TALKLFSGEVFTAFNNASIFKGLVRSYDLRGGKSKQFMFTGKLSAGYHTPGTPIVGDAGIKANEKTLVMDDLLVSSQFVYSLDEIFSQYSTRAEVSKQIGEALATHYDERIARVLAKASAEASPVTGEP
4	1w0rA	0.08	0.18	0.94	1.71	Download	VSVEDCCLNTAFAYQKRSGGLCQPCRSPRWSLWSVGWNGQCSGKVAPGTLEWQLQACEDQQCCPEMGGWSGWGPWEPCSVTACNHPAPKCGGHCPGQAQESEACDTQQVCPTHGAWATWGPWTPCSASCH
5	2ocwA	0.08	0.18	0.98	1.80	Download	---LGINSRGLSFDVSLEVSQGPGLLNDTKVYTVDLGRTVTINCPFKTENAQKRKSLYKQIGLYPVLVIDSSGYVNPNYTGRIRLDIQGTGQLDDSNSNKKNADLQVLKPEPELVYEDLRGSVTFHCALG
6	4btgA	0.10	0.17	0.93	1.53	Download	GFNL--------KVKDLNGSARGLTQLPLQFTRTFSASMTSELLWEAYVYRVGRTATYPFDANSPSTPKELDPSARLRNTNGIRGRAEVIFSDEELSSTIIPWFIEAMSEVSPFKLRPINETTSYIGQTS
7	3cm9S	0.10	0.18	0.98	1.69	Download	---LGINSRGLSFDVSLEVSQGPGLLNDTKVYTVDLGRTVTINCPFKTENAQKRKSLYKQIGLYPVLVIDSSGYVNPNYTGRIRLDIQGTGDDSNSNKKNADLQVLKPEPELVYEDLRGSVTFHCALGPE
8	4mguA	0.06	0.21	0.94	1.48	Download	IIPITRTVNDVTYVDNFVITSLGRLPNPNEFAQLPPGSCIEECNVRVTAFTPRIAFQATLNQNQFILHATGLNIKTQGVDVRPKEFQANEPMVVSSIDELGADENLFEDYVKEKSVPNHVPRHQFGIPYP
9	1kvpA	0.09	0.22	0.94	1.63	Download	--TEANPNELNQDDARYGFRCCPLPPETELSRQMTTSTGMAPVTTKFRDVPNLSGTPLIFRDNKGRTIKTGQLGIGPVDAGFLVAQNTAQAANGERAIPSNLWADLSNATSIDIMGLQAAYANLHTDQER
10	4jrfA	0.07	0.19	1.00	1.44	Download	YSVSVAPKSGELATTDYILNVGGANKAGYDGKAFKVPSPSDKFKTACVASASWSAINGAVEQTLDEVTGTSKNNFINAGDNANPTNGALVTANVKVVDGTTIPDVATAISEAQADRSIYTNPDGLKVPAG

(a) All the residues are colored in black; however, those residues in template which are identical to the residue in the query sequence are highlighted in color. Coloring scheme is based on the property of amino acids, where polar are brightly coloured while non-polar residues are colored in dark shade. (more about the colors used)

(b) Rank of templates represents the top ten threading templates used by I-TASSER.

(c) Ident1 is the percentage sequence identity of the templates in the threading aligned region with the query sequence.

(d) Ident2 is the percentage sequence identity of the whole template chains with query sequence.

(e) Cov represents the coverage of the threading alignment and is equal to the number of aligned residues divided by the length of query protein.

(f) Norm. Z-score is the normalized Z-score of the threading alignments. Alignment with a Normalized Z-score >1 mean a good alignment and vice versa.

(g) Download Align. provides the 3D structure of the aligned regions of the threading templates.

(h) The top 10 alignments reported above (in order of their ranking) are from the following threading programs:

1: Neff-PPAS 2: PROSPECT2 3: Neff-PPAS 4: PROSPECT2 5: Neff-PPAS 6: PROSPECT2 7: Neff-PPAS 8: PROSPECT2 9: Neff-PPAS 10: PROSPECT2

Top 5 Models predicted by I-TASSER

Generated 3D models

Estimated local accuracy of models

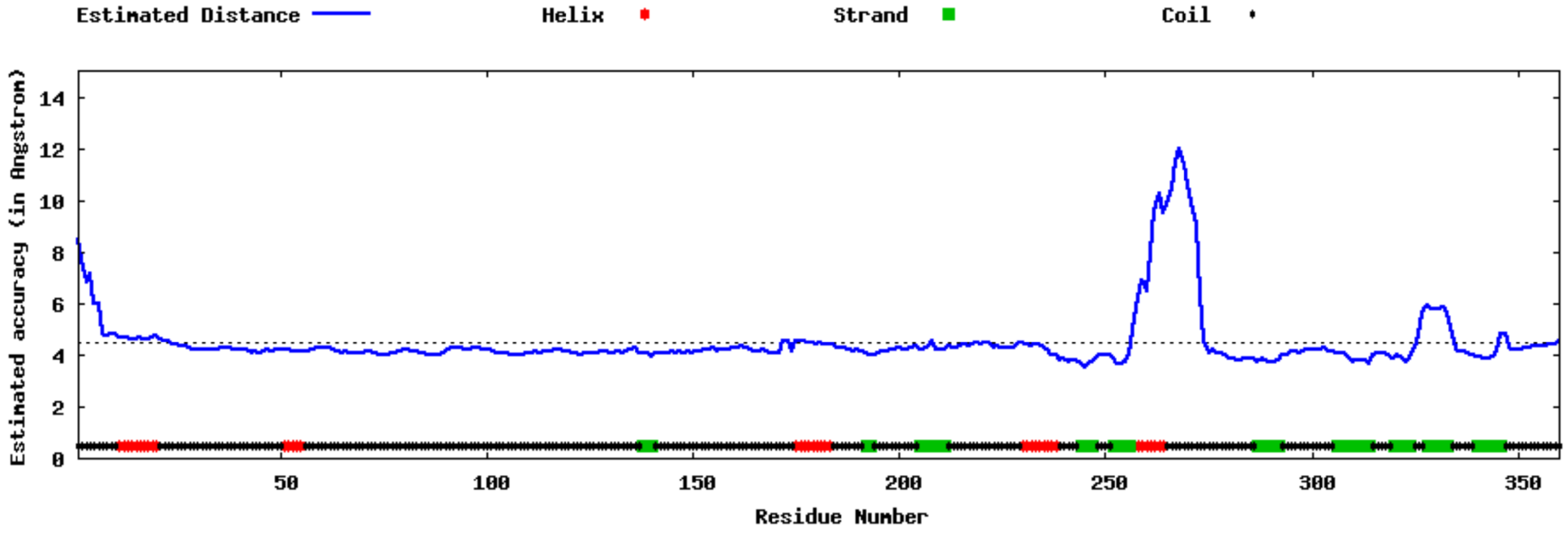

- Download Model 1
- C-score=-1.62 (Read more about C-score)
- Estimated TM-score = 0.52±0.15

- Estimated RMSD = 10.3±4.6Å

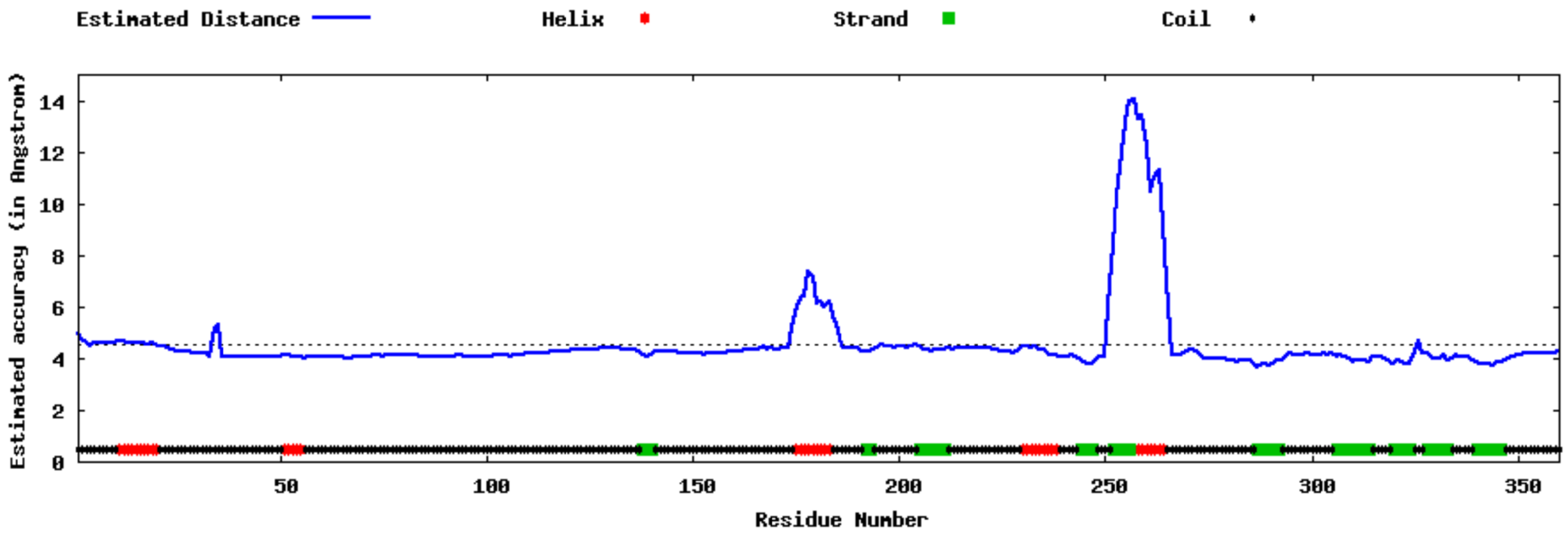

- Download Model 2
- C-score = -1.92

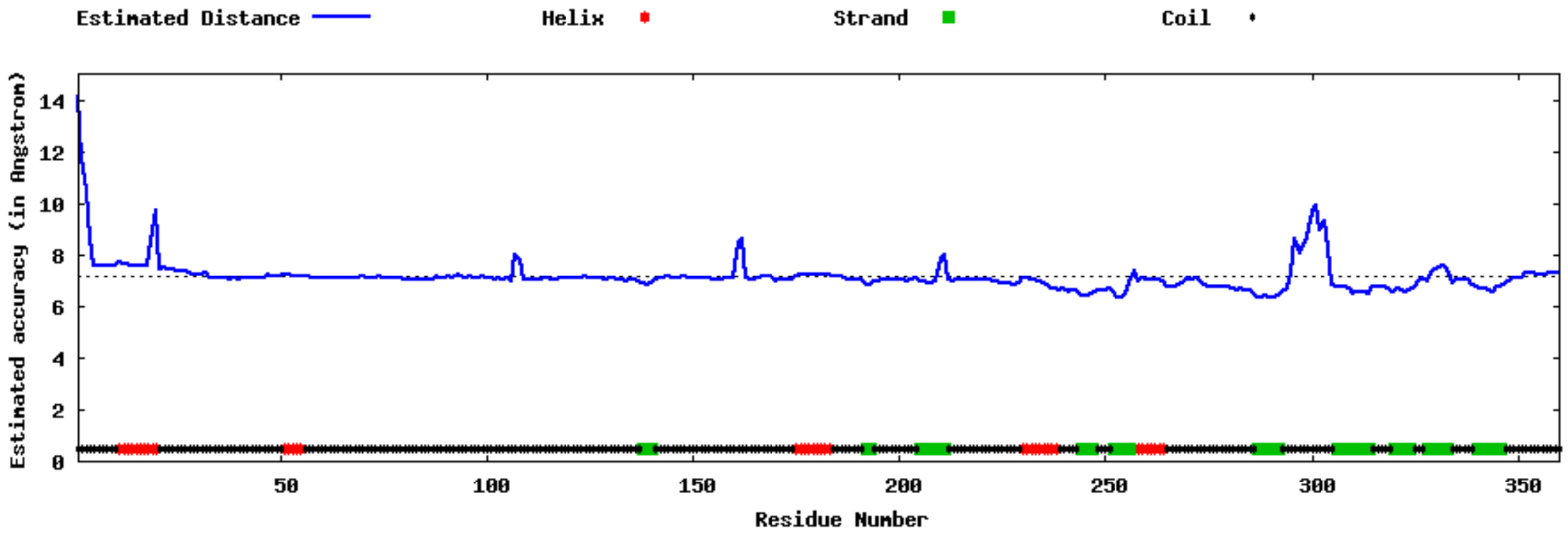

- Download Model 3
- C-score = -1.57

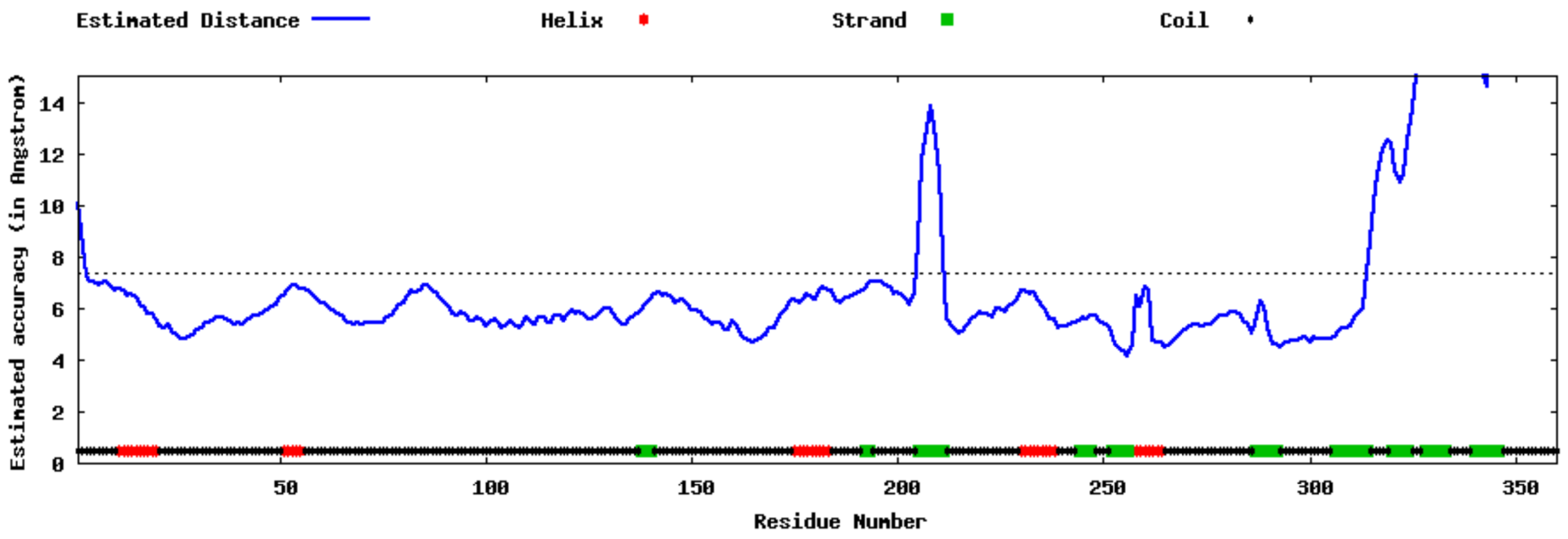

- [Download Model 4]
- C-score = -3.35

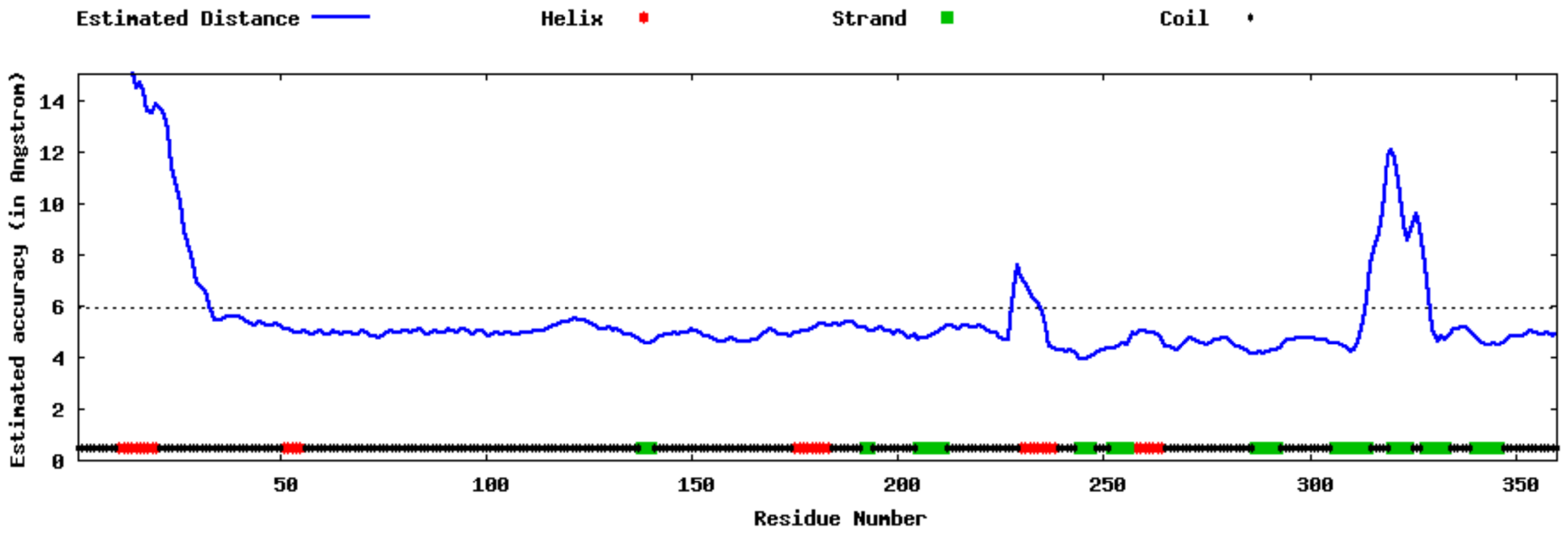

- [Download Model 5]
- C-score = -2.81

Proteins structurally close to the target in the PDB (as identified by TM-align)

Top 10 Identified stuctural analogs in PDB

Click to view	Rank	PDB Hit	TM-score	RMSD[a]	IDEN[a]	Cov	Alignment
○	1	1mg2E	0.899	1.43	0.066	0.928	Download
○	2	1maeH	0.897	1.20	0.070	0.917	Download
○	3	3c75J	0.895	1.34	0.054	0.919	Download
○	4	1mdaH	0.884	1.30	0.049	0.908	Download
○	5	1madH	0.884	1.30	0.049	0.908	Download
○	6	2h47H	0.764	2.56	0.045	0.850	Download
○	7	2vz1A	0.643	4.32	0.072	0.822	Download
○	8	1t2xA2	0.630	4.12	0.069	0.794	Download
○	9	1qniF	0.616	3.88	0.065	0.758	Download
○	10	3sbqA	0.615	4.00	0.068	0.764	Download

(a) Query structure is shown in cartoon, while the structural analog is displayed using backbone trace.

(b) Ranking of proteins is based on TM-score of the structural alignment between the query structure and known structures in the PDB library.

(c) RMSD[a] is the RMSD between residues that are structurally aligned by TM-align.

(d) IDEN[a] is the percentage sequence identity in the structurally aligned region.

(e) Cov represents the coverage of the alignment by TM-align and is equal to the number of structurally aligned residues divided by length of the query protein.

Reset to initial orientation ☐ Spin On/Off

Predicted function using COACH

Ligand binding sites

Click to view	Rank	C-score	Cluster size	PDB Hit	Lig Name	Download Complex	Ligand Binding Site Residues
◉	1	0.05	3	1ux1A	THU	N/A	258,259,260
○	2	0.03	2	2dg1A	CA	Rep, Mult	127,243,311,352,353
○	3	0.03	2	2z2pA	DOL	Rep, Mult	46,308,310,335
○	4	0.03	2	4c1yC	MFB	Rep, Mult	39,58,62,317,322,342
○	5	0.03	2	1eakC	PEPTIDE	N/A	200,203,205

Download the residue-specific ligand binding probability, which is estimated by SVM.
Download the all possible binding ligands and detailed prediction summary.
Download the templates clustering results.

(a) **C-score** is the confidence score of the prediction. C-score ranges [0-1], where a higher score indicates a more reliable prediction.
(b) **Cluster size** is the total number of templates in a cluster.
(c) **Lig Name** is name of possible binding ligand. Click the name to view its information inthe BioLiP database.
(d) **Rep** is a single complex structure with the most representative ligand in the cluster, i.e., the one listed in the**Lig Name** column.
Mult is the complex structures with all potential binding ligands in the cluster.

Reset to initial orientation ☐ Spin On/Off

Enzyme Commission (EC) numbers and active sites

Click to view	Rank	$Cscore^{EC}$	PDB Hit	TM-score	$RMSD^{a}$	$IDEN^{a}$	Cov	EC Number	Active Site Residues
○	1	0.258	2madH	0.896	1.21	0.070	0.917	1.4.99.3	271
○	2	0.257	2gc7A	0.898	1.45	0.066	0.928	1.4.99.3	245,248
○	3	0.254	3c75J	0.895	1.34	0.054	0.919	1.4.99.3	264,267,299
○	4	0.248	1madH	0.884	1.30	0.049	0.908	1.4.99.3	241
○	5	0.216	2h47H	0.764	2.56	0.045	0.850	1.4.99.4	254,261

Click on the radio buttons to visualize predicted active site residues.

(a) $Cscore^{EC}$ is the confidence score for the EC number prediction. $Cscore^{EC}$ values range in between [0-1]; where a higher score indicates a more reliable EC number prediction.
(b) TM-score is a measure of global structural similarity between query and template protein.
(c) $RMSD^{a}$ is the RMSD between residues that are structurally aligned by TM-align.
(d) $IDEN^{a}$ is the percentage sequence identity in the structurally aligned region.
(e) Cov represents the coverage of global structural alignment and is equal to the number of structurally aligned residues divided by length of the query protein.

Reset to initial orientation ☐ Spin On/Off

Gene Ontology (GO) terms

Top 10 homologous GO templates in PDB

Rank	Cscore^{GO}	TM-score	RMSD^a	IDEN^a	Cov	PDB Hit	Associated GO Terms
1	0.26	0.8977	1.18	0.07	0.92	1maeH	GO:0030416 GO:0042597 GO:0005515 GO:0055114 GO:0030058
2	0.26	0.8981	1.45	0.07	0.93	1mg2A	GO:0055114 GO:0006810 GO:0022900 GO:0016491 GO:0042597 GO:0030058 GO:0005515 GO:0030416
3	0.25	0.8953	1.34	0.05	0.92	3c75H	GO:0055114 GO:0042597 GO:0030058 GO:0030416 GO:0005515
4	0.25	0.8844	1.30	0.05	0.91	1mdaH	GO:0005515 GO:0030058 GO:0030416 GO:0042597 GO:0055114
5	0.22	0.7651	2.46	0.05	0.85	2h47F	GO:0022900 GO:0006810 GO:0030058 GO:0030059 GO:0042597 GO:0005515 GO:0055114 GO:0030416 GO:0016491
6	0.17	0.6408	4.42	0.07	0.83	1k3iA	GO:0055114 GO:0046872 GO:0045480 GO:0016491 GO:0005576 GO:0005515 GO:0007155
7	0.17	0.6161	3.88	0.07	0.76	1qniA	GO:0046872 GO:0016020 GO:0005515 GO:0004129 GO:0005507
8	0.10	0.3231	7.06	0.03	0.56	2ocwA	GO:0005515
9	0.07	0.2208	7.48	0.02	0.40	1kvpA	GO:0019012 GO:0030430 GO:0019028 GO:0005198
10	0.06	0.1413	6.73	0.05	0.24	1oqyA	GO:0006289 GO:0031593 GO:0006281 GO:0006974 GO:0005515 GO:0045070 GO:0032434 GO:0003697 GO:0044419 GO:0005634 GO:0000502 GO:0003684 GO:0043161

Consensus prediction of GO terms

**Molecular Function**	GO:0030058	GO:0005515	
**GO-Score**	0.76	0.76	
**Biological Process**	GO:0030416	GO:0006810	GO:0022900
**GO-Score**	0.76	0.42	0.42
**Cellular Component**	GO:0042597		
**GO-Score**	0.76		

(a) Cscore^{GO} is a combined measure for evaluating global and local similarity between query and template protein. It's range is [0-1] and higher values indicate more confident predictions.

(b) TM-score is a measure of global structural similarity between query and template protein.

(c) RMSD^a is the RMSD between residues that are structurally aligned by TM-align.

(d) IDEN^a is the percentage sequence identity in the structurally aligned region.

(e) Cov represents the coverage of global structural alignment and is equal to the number of structurally aligned residues divided by length of the query protein.

(f) The second table shows a consensus GO terms amongst the top scoring templates. The GO-Score associated with each prediction is defined as the average weight of the GO term, where the weights are assigned based on Cscore ^{GO} of the template.

[Click on S206754_results.tar.bz2 to download the tarball file including all modeling results listed on this page]

Please cite the following articles when you use the I-TASSER server:

1. Y Zhang. I-TASSER server for protein 3D structure prediction. BMC Bioinformatics, 9:40, 2008.
2. A Roy, A Kucukural, Y Zhang. I-TASSER: a unified platform for automated protein structure and function prediction. Nature Protocols, 5:725, 2010.
3. J Yang, A Roy, Y Zhang. Protein-ligand binding site recognition using complementary binding-specific substructure comparison and sequence profile alignment, Bioinformatics, 29:2588, 2013.
