# Supplementary material for: Phylogenetic Analysis, Lineage-Specific Expansion and Functional Divergence of seed dormancy 4-Like Genes in Plants
Source: PLoS One. 2016 Jun 14;11(6):e0153717. doi: 10.1371/journal.pone.0153717 (PMC4907471; doi:10.1371/journal.pone.0153717)
Supplement: S1 Table — (DOC) [file pone.0153717.s008.doc]

**S1 Table.** Predicted *Sdr4* and *Sdr4-like* gene models and related information.

| **Species** | **Locus name** | **Location** | **Transcript**  **name** | **Score** | **E-Value** | **Copies** | **Introns** | **ORF length^a^** | **NTP^b^** | **Theoretical**  **pI^c^/Mw (D)^d^** | **Gene name^e^** |
| --- | --- | --- | --- | --- | --- | --- | --- | --- | --- | --- | --- |
| *Oryza sativa* | LOC_Os07g39700.1  /Os07g0585700 | Chr7:23796611..23797642 forward | LOC_Os07g39700.1 (primary) | 1862 | 0.0 | 1 | 0 | 1032 | N | 8.89 / 35989.18 | *OsSdr4** |
| *P.virgatum* | Pavir.Ba01082  Pavirv00068421m  Pavirv00064794m  Pavirv00069224m | Chr02a:13345152..13346489 reverse | Pavir.Ba01082.1 (primary) | 677.6 | 0.0 | 7 | 0 | 984 | Y | 8.48 / 34614.84 | *PvSdr4L1* |
| *P.virgatum* | Pavir.Ba01084  Pavirv00068421m  Pavirv00069224m | Chr02a:13362769..13364172 reverse | Pavir.Ba01084.1 (primary) | 677.6 | 1.5E-18 |  | 2 | 1173 | Y | 9.54 / 41336.45 | *PvSdr4L2* |
| *P.virgatum* | Pavir.Bb01196  Pavirv00068421m  Pavirv00064794m  Pavirv00069224m | Chr02b:21827523..21829976 reverse | Pavir.Bb01196.1 (primary) | 639.7 | 1.5E-18 |  | 3 | 1347 | Y | 8.89 / 47678.66 | *PvSdr4L3* |
| *P.virgatum* | Pavir.Ib02658 | Chr09b:36751791..36752736 forward | Pavir.Ib02658.1 (primary) | 68.0 | 5.7E-9 |  | 1 | 825 | N | 5.15 / 29753.19 | *PvSdr4L4* |
| *P.virgatum* | Pavir.Ib02094 | Chr09b:23186782..23188327 reverse | Pavir.Ib02094.1 (primary) |  |  |  | 2 | 840 | N | 6.00 / 30684.46 | *PvSdr4L5* |
| *P.virgatum* | Pavir.Ia02857 | Chr09a:59116618..59117560 reverse | Pavir.Ia02857.1 (primary) | 84.2 | 7.4E-14 |  | 1 | 876 | N | 5.82 / 32148.97 | *PvSdr4L6* |
| *P.virgatum* | Pavir.Ia03049 | Chr09a:61790391..61791341 forward | Pavir.Ia03049.1 (primary) |  |  |  | 0 | 951 | N | 5.67 / 34841.94 | *PvSdr4L7* |
| *Setaria italica* | Si029889m.g | scaffold_2:44468882..44470507 reverse | Si029889m (primary) | 645.1 | 0.0 | 1 | 0 | 1305 | Y | 10.07 / 46715.74 | *SiSdr4L* |
| *Sorghum bicolor* | Sobic.002G356300 AliasSb02g037770 | Chr02:71882057..71883034 forward | Sobic.002G356300.1 (primary) | 578.4 | 2.4E-162 | 2 | 0 | 978 | N | 8.63 / 34135.02 | *SbSdr4L1* |
| *Sorghum bicolor* | Sobic.001G326000 | Chr01:54093346..54094951 forward | Sobic.001G326000.1 (primary) | 116.7 | 2.3E-23 |  | 0 | 966 | Y | 9.30 / 34074.07 | *SbSdr4L2* |
| *B.distachyon* | Bradi1g23171 | Bd1:18602525..18603377 reverse | Bradi1g23171.1 (primary) | 508.0 | 3.6E-141 | 2 | 1 | 849 | N | 9.29 / 28542.76 | *BdSdr4L1* |
| *B.distachyon* | Bradi1g23180 | Bd1:18607008..18607997 reverse | Bradi1g23180.1 (primary) |  |  |  | 0 | 990 | N | 9.26 / 34598.59 | *BdSdr4L2* |
| *Zea mays* | GRMZM2G396402 | 7:161724076..161725368 forward | GRMZM2G396402_T01 (primary) | 573.0 | 6.8e-161 | 3 | 0 | 984 | N | 8.07 / 34290.97 | *ZmSdr4L3* |
| *Zea mays* | GRMZM2G105302 | 2:208792243..208794560 reverse | GRMZM2G105302_T01 (primary) | 457.5 | 3.8E-126 |  | 0 | 939 | N | 7.99 / 32917.63 | *ZmSdr4L2* |
| *Zea mays* | GRMZM2G038991 | 1:78297267..78298351 reverse | GRMZM2G038991_T01 (primary) | 203.1 | 1.3E-49 |  | 0 | 933 | Y | 9.91 / 32719.45 | *ZmSdr4L1* |
| *Triticum aestivum* | KF021989 | Chromosome A | TaSdr4_1A |  |  |  |  | 993 | N | 6.13 / 34703.49 | *TaSdr4-A1b** |
| *Triticum aestivum* | KF021991 | Chromosome B | TaSdr4_1B |  |  |  |  | 981 | N | 7.56 / 34332.10 | *TaSdr4-B1b** |
| *Triticum aestivum* | KF021992 | Chromosome D | TaSdr4_1D |  |  |  |  | 987 | N | 7.00 / 34503.27 | *TaSdr4-D1** |
| *Aegilops Tauschii* | EG_Scaffold51441 | 34816 to 35597 (+) |  |  |  |  |  |  | N | 6.13 / 34703.49 | *A****e****tSdr4L* |
| *Hordeum vulgare* | 2HS  MLOC_79118.1 | 8398307:8399867 |  |  |  |  |  |  | N | 6.72 / 34882.65 | *Hv Sdr4L* |
| *Aquilegia coerulea Goldsmith* | Aquca_060_00028 | scaffold_60:444416..445498 reverse | Aquca_060_00028.1 (primary) | 51.8 | 8.1E-5 | 1 | 0 | 1083 | N | 8.74 / 39346.66 | *AcSdr4L* |
| *Mimulus guttatus* | Migut.N02788 (No transcript) | scaffold_14:23340690..23341441 reverse |  | 50 | 3E-4 | 1 | 0 |  |  |  |  |
| *Solanum lycopersicum* | Solyc04g077740.1 | SL2.40ch04:60251147..60252229 forward |  | 64.4 | 3.3E-8 | 1 | 0 | 1083 | Y | 6.83 / 39274.85 | *SlSdr4L1* |
| *Solanum tuberosum* | PGSC0003DMG400010622 | chr04:59031244..59032980 forward | PGSC0003DMT400027553 | 69.8 | 7E-10 | 1 | 0 | 1083 | Y | 6.65 / 39193.77 | *StSdr4L* |
| *Vitis vinifera* | GSVIVG01009335001  XM_002283317.1_cds | chr18:7523275..7526375 forward | GSVIVT01009335001 | 140.1 | 3.3E-31 | 1 | 4 | 840 | Y | 6.95 / 30956.95 | *VvSdr4L* |
| *Eucalyptus grandis* | Eucgr.F02242 | scaffold_6:30405532..30406668 reverse | Eucgr.F02242.1 (primary) | 77.0 | 4.6E-12 | 1 | 0 | 1137 | Y | 9.32 / 40332.22 | *EgSdr4L* |
| *Populus trichocarpa* | Potri.014G015300 | Chr14:1502482..1503862 forward | otri.014G015300.1 (primary) | 107.0 | 1.7E-21 | 2 | 0 | 1062 |  | 8.26 / 38986.58 | *PtSdr4L2* |
| *Populus trichocarpa* | Potri.002G117700 | Chr02:8824160..8825251 forward | Potri.002G117700.1 (primary) | 87.8 | 1.6E-15 |  | 0 | 1092 | Y | 8.90 / 40092.98 | *PtSdr4L1* |
| *Linum usitatissimum* | Lus10007752.g | scaffold993:210845..211924 reverse | Lus10007752 (primary) | 111.3 | 1E-22 | 2 | 0 | 1082 | Y | 9.04 / 39748.30 | *LuSdr4L2* |
| *Linum usitatissimum* | Lus10018671.g | scaffold461:144043..145116 forward | Lus10018671 (primary) | 107.7 | 1.3E-21 |  | 0 | 1074 |  | 9.05 / 39592.18 | *LuSdr4L1* |
| *Manihot esculenta* | cassava4.1_031033m.g | scaffold03237:102940..104016 forward | cassava4.1_031033m (primary) | 102.3 | 9E-20 | 1 | 0 | 1077 | Y | 8.64 / 39429.15 | *MeSdr4L* |
| *Ricinus communis* | 30170.t000017 | 30170:684056..685391 reverse | 30170.m013605 (primary) | 86.0 | 4.5E-15 | 1 | 0 | 1092 | Y | 8.26 / 39747.14 | *RcSdr4L* |
| *Carica papaya* | evm.TU.supercontig_142.38 | supercontig_142:439128..440198 reverse | evm.model.supercontig_142.38 (primary) | 116.7 | 2.6E-24 | 1 | 0 | 1071 | Y | 9.14 / 39152.84 | *CpSdr4L* |
| *Gossypium raimondii* | Gorai.009G220700 | Chr09:17106324..17108457 reverse | Gorai.009G220700.1 (primary) | 392.6 | 5.1E-107 | 2 | 0 | 1089 | Y | 9.05 / 39862.12 | *GrSdr4L1* |
| *Gossypium raimondii* | Gorai.010G026200 | Chr10:2122531..2123457 reverse | Gorai.010G026200.1 (primary) | 264.6 | 1.8E-68 |  | 0 | 927 | Y | 9.40 / 33896.17 | *GrSdr4L2* |
| *Theobroma cacao* | Thecc1EG034686 | scaffold_8:4325703..4327237 forward | Thecc1EG034686t1 (primary) | 127.5 | 1.5E-27 | 1 | 0 | 1095 | Y | 8.89 / 39453.23 | *TcSdr4L1* |
| *Arabidopsis lyrata* | 313471 | scaffold_1:12018834..12025360 reverse | 313471 (primary) | 1662.2 | 0.0 | 1 | 0 | 3145 | N |  |  |
| *Arabidopsis lyrata* | 313471 | scaffold_1:12018834..12025360 reverse | 313471 (primary) | 75.2 | 4.7E-12 |  | 0 | 3145 | N |  |  |
| *Arabidopsis Thaliana* | AT1G27461 | Chr1:9537746..9539013 reverse | AT1G27461.1 (primary) | 44.6 | 4.6E-31 | 1 | 0 | 1065 | Y | 9.33 / 39520.08 | *At Sdr4L* |
| *Boechera stricta* | Bostr.15697s0076 | Scaffold15697:372974..374089 reverse | Bostr.15697s0076.1 (primary) | 1476.4 | 0.0 | 1 | 0 | 1116 | Y | 9.17 / 41211.98 | *BsSdr4L1* |
| *Brassica rapa* | Brara.I03014 | A09:29237984..29239060 forward | Brara.I03014.1 (primary) | 1018.4 | 0.0 | 2 | 0 | 1077 | Y | 8.87 / 39785.43 | *BrSdr4L1* |
| *Brassica rapa* | Brara.H02049 | A08:18545387..18546475 forward | Brara.H02049.1 primary) | 1014.8 | 0.0 |  | 0 | 1089 | Y | 9.22 / 40247.04 | *BrSdr4L2* |
| *Capsella grandiflora* | Cagra.16111s0005.1 | Scaffold16111:9648..10736 reverse | Cagra.16111s0005.1 (primary) | 1323.2 | 0.0 | 1 | 0 | 1089 |  | 9.03 / 40267.17 | *CgSdr4L* |
| *Capsella rubella* | Carubv10011167m | scaffold_1:9379587..9380675 reverse | Carubv10011167m (primary) | 1326.8 | 0.0 | 1 | 0 | 1089 | Y | 9.03 / 40223.12 | *CrSdr4L* |
| *Eutrema salsugineum* | Thhalv10009308m.g | scaffold_5:5796557..5797672 forward | Thhalv10009308m (primary) | 1123.0 | 0.0 | 1 | 0 | 1116 | Y | 9.18 / 41065.88 | *EsSdr4L* |
| *Citrus sinensis* | orange1.1g019820m | scaffold00004:3198164..3199289 forward | orange1.1g019820m (primary) | 134.7 | 9.1E-30 | 1 | 0 | 1008 | Y | 8.38 / 36469.52 | *CsSdr4L* |
| *Citrus clementina* | Ciclev10001560m | scaffold_5:34684730..34686032 reverse | Ciclev10001560m (primary) | 138.3 | 7.1E-31 | 1 | 0 | 1110 |  | 7.54 / 40297.93 | *CcSdr4L* |
| *Cucumis sativus* | Cucsa.352870 | scaffold03533:557508..558620 reverse | Cucsa.352870.1 (primary) | 122.1 | 3.7E-26 | 1 | 0 | 1113 | Y | 8.16 / 41115.26 | *C****sa****Sdr4L* |
| *Fragaria vesca* | gene02593-v1.0-hybrid | LG2:17834828..17835958 reverse | mrna02593.1-v1.0-hybrid (primary) | 104.1 | 1E-20 | 1 | 0 | 1137 | Y | 8.36 / 40707.28 | *FvSdr4L* |
| *Glycine max* | Glyma.17G234300.1AliasG lyma17g35260 | Chr17:38981222..38982848 forward | Glyma.17G234300.1 (primary) | 102.3 | 1.6E-19 | 2 | 0 | 1095 | Y | 9.30 / 40337.17 | *GmSdr4L1* |
| *Glycine max* | Glyma.U030500.1  AliasGlyma14g09910 | scaffold_28:290258..291557 forward | Glyma.U030500.1 (primary) | 86.0 | 1.3E-14 | 2 | 0 | 1107 | Y | 8.87 / 40578.36 | *GmSdr4L2* |
| *Malus domestica* | MDP0000900422 | MDC003283.409:4273..5382 forward | MDP0000900422 (primary) | 80.6 | 4.8E-13 | 2 | 0 | 1110 |  | 8.36 / 40351.49 | *MdSdr4L1* |
| *Malus domestica* | MDP0000195679 | MDC012575.238:3112..4413 forward | MDP0000195679 (primary) | 71.6 | 2.5E-10 |  | 0 | 1302 | Y | 9.34 / 47851.73 | *MdSdr4L2* |
| *Medicago truncatula* | Medtr1g017920.1 | chr1:5111821..5113230 reverse | Medtr1g017920.1 (primary) | 69.8 | 4.1E-10 | 1 | 0 | 1101 | Y | 8.97 / 40492.04 | *MtSdr4L* |
| *Phaseolus vulgaris* | Phvul.001G028500.1 | Chr01:2633208..2634434 reverse | Phvul.001G028500.1 (primary) | 86.0 | 6.8E-15 | 1 | 0 | 1095 | Y | 9.21 / 40046.94 | *P****h****vSdr4L* |
| *Prunus persica* | ppa021982m | scaffold_1:35445453..35446586 forward | ppa021982m (primary) | 91.5 | 6.9E-17 | 1 | 0 | 1134 | Y | 6.47 / 41223.72 | *PpSdr4L* |

a Denotes manual ORF length annotation based on conceptual translation of available genomic DNA sequence.

b NTP (N-terminal transit peptide) as predicted by ChloroP 1.1 (left, Y: transit peptide; N: no transit peptide)

c. pI = isoelectric point of the deduced polypeptide.

d. Mw = molecular weight.

e Names pertaining to this paper.

* Denotes already identified Sdr4 genes from previous reports: OsaSdr4 (Sugimoto et al., 2010); TaSdr4-1Ab, 1Bb & 1D (Zhang et al., 2014);
