# Supplementary material for: Phylogenetic Analysis, Lineage-Specific Expansion and Functional Divergence of seed dormancy 4-Like Genes in Plants
Source: PLoS One. 2016 Jun 14;11(6):e0153717. doi: 10.1371/journal.pone.0153717 (PMC4907471; doi:10.1371/journal.pone.0153717)
Supplement: S3 Table — (DOCX) [file pone.0153717.s010.docx]

| **S3 Table.** Amino acid sites of functional divergence between subfamilies of the Sdr4L proteins. | | | |
| --- | --- | --- | --- |
| Group 1 | Group 2 | Type-I (Q_k_>0.9) | Type-II (Q_k_>0.9) |
| Sub I | Sub II | 128 (1M), 132 (5Q), 146 (10A), 147 (11V), 148 (12K), 160 (24A), 163 (27P), 220 (60P), 222 (64R), 225 (65K), 239 (77P), 280 (92P), 414 (173L), 419 (178 V), 429 (18E), 454 (205A), 459 (210E), 460 (211E), 467 (218S), 492 (245Q) 500 (249P), 558 (290S), 584 (302S), 585 (303S) | 130 (3M), 131 (4V), 132 (5Q), 133 (6P), 146 (10A), 147 (11V), 148 (12K), 149 (13A), 150 (14N), 151 (15E), 152 (16I), 153 (17L),217 (57Q), 219 (59R), 220 (60P), 222 (62R), 224 (64R), 225 (65K), 226 (66R), 391 (164R), 393 (166L), 415 (174E), 418 (177A), 423 (182G), 425 (184T), 427 (186H), 436 (195G), 454 (205A), 458 (209A), 459 (210E), 477 (227D), 484 (233R), 492 (241R), 498 (247E), |
| Sub I | Sub VI | - | 128 (1M), 129 (2A), 146 (10A), 149 (13A), 150 (14N), 218 (58S), 223 (63A), 280 (92P), 392 (165K),394 (167L), 417 (176R), 418 (177A), 425 (184T), 427 (186H), 429 (188E), 430 (189S), 434 (193D), 435 (194V), 436 (195G), 452 (203A), 453 (204A), 454 (205A), 455 (206P), 458 (209A), 459 (210E), 463 (214A), 467 (218S), 470 (220S), 473 (223A), 488 (237D), 558 (290S), 584 (302S), 585 (303S), |
| Sub I | Sub VIIIb | 473 (223A) | 128 (1M), 129 (2A),130 (3M), 132 (5Q), 146 (10A), 147 (11V), 149 (13A), 150 (14N), 218 (58S), 223 (63A), 238 (76S), 280 (92P), 281 (93V), 391 (164R), 392 (165K), 394 (167L), 417 (176R), 418 (177A), 427 (186H), 429 (188E), 434 (193D), 435 (194V), 436 (195G), 452 (203A), 453 (204A), 454 (205A), 458 (209A),459 (210E), 463 (214A), 467 (218S), 475 (225V), 481 (230N), 485 (233L), 486 (235V), 488 (237D), 498 (247E), 558 (290S), 559 (291A), 584 (302S), |
| Sub I | Sub IV | - | 129 (2A), 130 (3M), 146 (10A), 149 (13A), 150 (14N), 218 (58S), 223 (63A), 235 (73P), 280 (92P), 281 (93V), 391 (164R), 392 (165K), 394 (167L), 415 (174E), 417 (176R),418 (177A), 427 (186H), 429 (188E), 434 (193D), 435 (194V), 436 (195G), 453 (204A), 458 (209A), 463 (214A), 467 (218S), 473 (223A), 481 (230N), 488 (237D), 558 (290S), 585 (303S), |
| Sub I | Sub VIIIa | - | 129 (2A), 130 (3M), 146 (10A), 147 (11V), 149 (13A), 150 (14N), 218 (58S), 223 (63A), 235 (73P), 280 (92P), 392 (165K), 394 (167L), 415 (174E), 417 (176R), 418 (177A), 427 (186H), 429 (188E), 436 (195G), 453 (204A), 458 (209A), 463 (214A), 467 (218S), 479 (229S), 488 (237D), 558 (290S), 585 (303S), |
| Sub II | Sub VI | 131(4V), 418 (177A), 435 (194V), 436 (195G), 454 (205A), 459 (210E), 460 (211E), 464 (215E), 465 (216L), 471 (221L), 473 (223A), 478 (228S), 478 (228S), 560 (292K), 585 (303S) | 129 (2A), 130 (3M), 131 (4V), 132 (5Q), 133 (6P), 146 (10A), 147 (11V), 148 (12K), 149 (13A), 151 (15E), 152 (16I), 153 (17L), 217 (57Q), 218 (58S), 219 (59R), 220 (60P), 222 (62R), 223 (63A), 224 (64R), 225 (65K), 226 (66R), 280 (92P), 391 (164R), 392 (165K), 415 (174E), 417 (176R), 423 (182G), 425 (184T), 429 (188E), 430 (189S), 434 (193D), 435 (194V), 436 (195G), 452 (203A), 453 (204A), 455 (206P), 459 (210E), 463 (214A), 467 (218S), 470 (220S), 473 (223A), 477 (227D), 484 (233R), 488 (237D), 492 (241R), 498 (247E), 558 (290S), 584 (302S), 585 (303S) |
| Sub II | Sub VIIIb | 128 (1M), 130 (3M), 131(4V), 132(5Q), 146(10A), 147 (11V), 148 (12K), 150 (14N), 160 (24A),163 (27P), 218 (58S), 220 (60P), 222 (62R), 224 (64R), 225 (65K), 238 (76S), 392 (165K), 417 (176R), 419 (178V), 423 (182G), 427 (186H), 429 (188E), 435 (194V), 452 (203A), 459 (210E), 461 (212V), 467 (218S), 469 (219D), 470 (220S), 473 (223A), 478 (228S), 479 (229S), 483 (232V), 584 (302S), | 129 (2A), 130 (3M), 131 (4V), 132 (5Q), 133 (6P), 146 (10A), 147 (11V), 148 (12K), 149 (13A), 151 (15E), 152 (16I), 153 (17L), 217 (57Q), 218 (58S), 219 (59R), 220 (60P), 222 (62R), 223 (63A), 224 (64R), 225 (65K), 226 (66R), 238 (76S), 280 (92P), 391 (164R), 392 (165K), 415 (174E), 417 (176R), 423 (182G), 425 (184T), 429 (188E), 434 (193D), 435 (194V), 452 (203A), 453 (204A), 459 (210E), 463 (214A), 467 (218S), 470 (223A), 477 (227D), 481 (230N), 484 (233R), 488 (237D),  492 (241R), 558 (290S), 584 (302S) |
| Sub II | Sub IV | 128 (1M), 130 (3M), 131 (4V), 146 (10A), 147 (11V), 148 (12K), 150 (14N), 160 (24A), 163 (27P), 218 (58S), 220 (60P), 224 (64R), 225 (65K), 238 (76S), 392 (165K), 415 (174E), 417 (176R), 419 (178V), 421 (180P), 428 (187V), 429 (188E), 435 (194V), 452 (203A),  454 (205A), 461 (212V), 465 (216L), 467 (218S), 470 (220S), 473 (223A), 476 (226S), 478 (228S), 498 (247E), 500 (249P), 560 (292K), 561 (293I), 584 (302S),585 (303S), | 129 (2A), 130 (3M), 131 (4V), 132 (5Q), 133 (6P), 146 (10A), 147 (11V), 148 (12K), 149 (13A), 151 (15E), 152 (16I), 153 (17L), 217 (57Q), 218 (58S), 219 (59R), 220 (60P), 222 (62R), 223 (63A), 224 (64R), 225 (65K), 226 (66R), 235 (73P), 280 (92P), 281 (93V), 392 (165K), 415 (174E), 417 (176R), 423 (182G), 425 (184T), 429 (188E), 434 (193D), 435 (194V), 453 (204A), 454 (205A), 459 (210E), 463 (214A), 467 (218S), 470 (220S), 473 (223A), 477 (227D), 481 (230N), 484 (233R), 488 (237D), 492 (241R), 498 (247E), 558 (290S), 584 (302S), 585 (303S), |
| Sub II | Sub VIIIa | 239 (77P), 421 (180P), 459 (210E), 485 (234L), 585 (303S) | 129 (2A), 130 (3M), 131 (4V), 132 (5Q), 133 (6P), 146 (10A), 147 (11V), 148 (12K), 149 (13A), 151 (15E), 152 (16I), 153 (17L), 217 (57Q), 218 (58S), 219 (59R), 220 (60P), 222 (62R), 223 (63A), 224 (64R), 225 (65K), 226 (66R), 235 (73P), 280 (92P), 391 (164R), 392 (165K), 415 (174E), 417 (176R), 423 (182G), 425 (184T), 429 (188E), 434 (193D), 435 (194V), 453 (204A), 454 (205A), 459 (210E), 463 (214A), 467 (218S), 470 (220S), 477 (227D), 479 (229S), 484 (234R), 488 (238D), 492 (241R), 498 (247E), 558 (290S), 584 (302S), 585 (303S), |
| Sub VI | Sub IV | 427 (186H) | 130 (3M), 146 (10A), 147 (11V), 153 (17L), 235 (73P), 281 (93V), 391 (164R), 425 (184T), 430 (189S), 435 (194V), 436 (195G), 455 (206P), 463 (214A), 469 (219D), 481 (231N),486 (236V),494 (243V), 500 (249P), 584 (302S), 585 (303S), |
| Sub VI | Sub VIIIa | - | 129 (2A), 281 (93V), 435 (194V), 436 (195G) |
| Sub VIIIb | Sub IV | - | 129 (2A), 235 (73P), 429 (188E), 436 (195G), 584 (302S) |
| Sub VIIIb | Sub VIIIa | - | 238 (76S), 281 (93V) |
| Sub IV | Sub VIIIa | - | 147 (11V), 235 (73P), 238 (76S), 391 (164R), 429 (188E), 435 (194V), 454 (205A), 479 (229S), 584 (302S), 585 (303S) |
| Sub VI | Sub IV | - | - |

Note: The alignment (amino acid) positions presented above are based on the rice OsSdr4L protein.
