# Supplementary material for: Phylogenetic Analysis, Lineage-Specific Expansion and Functional Divergence of seed dormancy 4-Like Genes in Plants
Source: PLoS One. 2016 Jun 14;11(6):e0153717. doi: 10.1371/journal.pone.0153717 (PMC4907471; doi:10.1371/journal.pone.0153717)
Supplement: S4 Table — (DOCX) [file pone.0153717.s011.docx]

**S4 Table.** Parameter estimations and likelihood ratio tests for the branch-site models.

| **Cluster** | **Site class** | **Proportion** | **Background ɯ** | **Foreground ɯ** | **Posterior proba-**  **bilities**  **%** | **Positive selected sites^a^** |
| --- | --- | --- | --- | --- | --- | --- |
| Sub-I  (Grass 1) | 0  1  2a  2b | 0.84349  0.12594 0.02659 0.00397 | 0.12823 1.00000 0.12823 1.00000 | 0.12823 1.00000 3.57904 3.57904 | > 90 % | 241 R |
| Sub-II  (Grass 2) | 0  1  2a  2b | 0.53159 0.07336 0.34714 0.04791 | 0.11702 1.00000 0.11702 1.00000 | 0.11702 1.00000 1.00000 1.00000 | > 90 % | 2 A, **3 M**, 4 V, **13 A**, **16 I**, **20 F**, **59 R**, 60 P, 61C, 74 P*, **186 H*** |
| Sub-III  (Pentapetalae) | 0  1  2a  2b | 0.87094 0.12906 0.00000 0.00000 | 0.12910 1.00000 0.12910 1.00000 | 0.12910 1.00000 1.00000 1.00000 | > 50 % | None |
| Sub-IV  (Malvidae) | 0  1  2a  2b | 0.87094 0.12906 0.00000 0.00000 | 0.12910 1.00000 0.12910 1.00000 | 0.12910 1.00000 1.00000 1.00000 | - | None |
| Sub-V  (Brasicales Malvels) | 0  1  2a  2b | 0.87094 0.12906 0.00000 0.00000 | 0.12910 1.00000 0.12910 1.00000 | 0.12910 1.00000 1.00000 1.00000 | - | None |
| Sub-VI  (Brasicaceae) | 0  1  2a  2b | 0.86214 0.12853 0.00812 0.00121 | 0.12824 1.00000 0.12824 1.00000 | 0.12824 1.00000 8.45262 8.45262 | > 95 % | **210 E** |
| Sub-VII  (Citrus) | 0  1  2a  2b | 0.73875 0.10986 0.13179 0.01960 | 0.12767 1.00000 0.12767 1.00000 | 0.12767 1.00000 1.00000 1.00000 | > 50 % | 57 Q, 63 A |
| Sub-VIIIa  (Fabidae F1) | 0  1  2a  2b | 0.87094 0.12906 0.00000 0.00000 | 0.12910 1.00000 0.12910 1.00000 | 0.12910 1.00000 1.00000 1.00000 | - | None |
| Sub-VIIIb  (Fabidae F2) | 0  1  2a  2b | 0.82230 0.12191 0.04858 0.00720 | 0.12625 1.00000 0.12625 1.00000 | 0.12625 1.00000 2.54080 2.54080 | > 70 % | 184 T, 210 E,  218 S |

Note: **^a^**Positive-selection sites are inferred at posterior probabilities between 50-90% with those reaching 99% shown in bold. *Sites were also implicated in site model positive selection. Codon (amino acid) positions presented above are based on rice (OsSdr4) protein.
