# Supplementary material for: Phylogenetic Analysis, Lineage-Specific Expansion and Functional Divergence of seed dormancy 4-Like Genes in Plants
Source: PLoS One. 2016 Jun 14;11(6):e0153717. doi: 10.1371/journal.pone.0153717 (PMC4907471; doi:10.1371/journal.pone.0153717)
Supplement: S5 Table — (DOCX) [file pone.0153717.s012.docx]

**S5 Table.** Functions and number of identified *cis* elements in the 5’ flanking region of *Sdr4*-like genes from different plants species.

| **Species** | **Accession no.** | **Gene id.** | **ABA**  **responsiveness** | **Methyl- jasmonate**  **responsiveness** | **Light responsiveness** | | | | **Seed and endosperm specific expression/regulation** | | | **Flower induction** |
| --- | --- | --- | --- | --- | --- | --- | --- | --- | --- | --- | --- | --- |
|  |  |  | **ABRE** | **CGTCA** | **ACE** | **AE-box** | **CE3** | **G-Box** | **GCN** | **SKN** | **RY** | **Circadian clock** |
| *Oryza sativa* | LOC_Os07g39700.1 | *OsSdr4* | 1 | 1 | 1 | 1 | 1 | 2 |  | 1 | 1 |  |
| *Sorghum bicolor* | Sobic.002G356300 | *SbSdr4L2* | 1 | 1 | 1 | 1 |  | 2 |  | 1 | 1 | 1 |
| *Sorghum bicolor* | Sobic.001G326000 | *SbSdr4L1* | 1 | 1 |  |  |  | 2 | 1 | 1 | 1 | 1 |
| *Setaria italica* | Si029889m.g | *SiSdr4L* | 1 | 1 | 1 |  | 1 | 2 |  | 1 | 1 |  |
| *Panicum virgatum* | Pavir.Ib02658 | *PvSdr4L4* | 1 | 1 |  |  |  | 2 | 1 |  |  | 1 |
| *Panicum virgatum* | Pavir.Ib02094 | *PvSdr4L5* | 1 | 1 |  | 1 |  | 2 | 1 | 1 |  |  |
| *Panicum virgatum* | Pavir.Ba01084 | *PvSdr4L2* | 1 |  |  |  |  |  |  |  |  |  |
| *Panicum virgatum* | Pavir.Ba01082 | *PvSdr4L1* | 1 | 1 | 1 | 1 |  | 2 |  | 1 | 1 |  |
| *Panicum virgatum* | Pavir.Bb01196 | *PvSdr4L3* | 1 | 1 |  |  |  | 2 |  | 1 | 1 |  |
| *Panicum virgatum* | Pavir.Ia03049 | *PvSdr4L7* | 1 | 1 | 1 |  |  | 2 |  | 1 |  | 1 |
| *Zea mays* | Z.Mays2G10530 2 | *ZmSdr4L2* | 1 | 1 | 1 |  | 1 | 2 |  | 1 | 1 | 1 |
| *Zea mays* | Z.Mays2G396402 | *ZmSdr4L3* | 1 | 1 | 1 |  |  | 2 |  | 1 | 1 |  |
| *Zea mays* | Z.Mays2G038991 | *ZmSdr4L1* | 1 | 1 | 1 |  |  | 2 | 1 | 1 | 1 |  |
| *Brachypodium distachyon* | Bradi1g23171 | *BdSdr4L1* | 1 | 1 |  | 1 |  | 2 | 1 | 1 | 1 | 1 |
| *Brachypodium distachyon* | Bradi1g23180 | *BdSdr4L2* | 1 | 1 | 1 | 1 |  | 2 | 1 | 1 | 1 | 1 |
| *Hordeum vulgare* | Barley | *HvSdr4L* | 1 | 1 | 1 |  |  | 2 |  | 1 | 1 | 1 |
| *Arabidopsis thaliana* | AT1G27461 | *AtSdr4L* | 1 | 1 |  | 1 |  | 2 | 1 | 1 |  |  |
| *Cucumis sativus* | Cucsa.352870 | *CsSdr4L* | 1 |  | 1 |  |  | 2 |  |  |  |  |
| *Carica papaya* | evm.TU.supercontig_142.38 | *CpSdr4L* | 1 |  | 1 | 1 |  | 2 |  |  |  |  |
| *Solanum tuberosum* | PGSC0003DMG400010622 | *StSdr4L* | 1 | 1 |  | 1 |  | 1 |  | 1 | 1 |  |
| *Glycine Max* | Glyma14g09910 | *GmSdr4L1* | 1 |  |  |  |  | 2 |  | 1 | 1 | 1 |
| *Glycine Max* | Glyma17g35260 | *GmSdr4L2* | 1 |  | 1 |  |  | 2 | 1 | 1 | 1 |  |
| *Brassica rapa* | Brara.I0301 | *BrSdr4L1* | 1 | 1 | 1 |  |  | 2 |  | 1 | 1 |  |
| *Brassica rapa* | Brara.H02049 | *BrSdr4L2* | 1 | 1 | 1 |  |  | 2 |  |  | 1 |  |
| *Aquilegia coerulea* | Aquca_060_00028 | *AcSdr4L* | 1 |  |  |  |  | 2 |  | 1 | 1 | 1 |
| *Populus trichocarpa* | Potri.014G015300 | *PtSdr4L2* | 1 | 1 |  |  |  | 2 | 1 |  | 1 | 1 |
| *Populus trichocarpa* | Potri.002G117700 | *PtSdr4L1* | 1 |  | 1 |  |  | 2 | 1 |  | 1 | 1 |
| *Eucalyptus grandis* | Eucgr.F02242 | *EgSdr4L* | 1 | 1 | 1 |  |  | 2 | 1 | 1 | 1 | 1 |
| *Solanum lycopersicum* | Solyc04g077740.1 | *SlSdr4L* | 1 | 1 | 1 |  |  | 2 |  | 1 | 1 |  |
| *Citrus clementina* | Ciclev10001560m | *CcSdr4L* | 1 | 1 |  |  |  | 2 |  | 1 |  | 1 |
| *Citrus sinensis* | orange1.1g019820m | *CsDr4L* | 1 |  |  |  |  | 2 |  | 1 | 1 | 1 |
| *Medicago truncatula* | Medtr1g017920.1 | *MtSdr4L* | 1 | 1 |  |  |  | 2 |  | 1 | 1 | 1 |
| *Linum usitatissimum* | Lus10007752.g | *LuSdr4L2* | 1 | 1 |  | 1 |  | 2 |  | 1 | 1 | 1 |
| *Linum usitatissimum* | Lus10018671.g | *LuSdr4L1* | 1 | 1 |  | 1 |  | 2 |  | 1 | 1 |  |
| *Ricinus communis* | 30170.t000017 | *RcSdr4L* | 1 | 1 |  |  |  | 2 | 1 | 1 | 1 | 1 |
| *Vitis vinifera* | GSVIVG01009335001  XM_002283317.1_cds | *VvSdr4L* |  | 1 | 1 |  |  |  |  | 1 |  | 1 |
| *Manihot esculenta* | cassava4.1_031033m.g | *MeSdr4L* | 1 | 1 |  |  |  | 2 |  | 1 | 1 |  |
| *Gossypium raimondii* | Gorai.009G220700 | *GrSdr4L1* |  |  | 1 |  |  |  |  | 1 |  | 1 |
| *Gossypium raimondii* | Gorai.010G026200 | *GrSdr4L2* | 1 | 1 |  |  |  | 2 | 1 | 1 | 1 | 1 |
| *Theobroma cacao* | Thecc1EG034686 | *TcSdr4L* | 1 | 1 | 1 | 1 |  | 2 |  |  |  | 1 |
| *Boechera stricta* | Bostr.15697s0076 | *BsSdr4L* | 1 | 1 | 1 |  |  | 2 | 1 | 1 |  |  |
| *Capsella grandiflora* | Cagra.16111s0005.1 | *CgSdr4L* | 1 | 1 | 1 | 1 |  | 2 |  | 1 |  | 1 |
| *Capsella rubella* | Carubv10011167m | *CrSdr4LEs* | 1 | 1 | 1 | 1 |  | 2 |  | 1 |  | 1 |
| *Eutrema salsugineum* | Thhalv10009308m | *Sdr4L* | 1 | 1 | 1 |  |  | 2 | 1 | 1 |  |  |
| *Fragaria vesca* | gene02593-v1.0-hybrid | *FvSdr4L* | 1 |  | 1 | 1 |  | 2 | 1 | 1 |  |  |
| *Prunus persica* | ppa021982m | *Pp Sdr4L* | 1 | 1 |  |  |  | 2 | 1 | 1 |  |  |
| *Phaseolus vulgaris* | Phvul.001G028500.1 | *Phv Sdr4L* | 1 | 1 |  |  |  | 2 |  | 1 | 1 | 1 |
